# Sex differences in the association of phospholipids with components of the metabolic syndrome in young adults

**DOI:** 10.1186/s13293-017-0131-0

**Published:** 2017-03-28

**Authors:** Sebastian Rauschert, Olaf Uhl, Berthold Koletzko, Trevor A. Mori, Lawrence J. Beilin, Wendy H. Oddy, Christian Hellmuth

**Affiliations:** 1 Ludwig-Maximilian Universität München, Dr. von Hauner Children’s Hospital, University of Munich Medical Center, Lindwurmstr. 4, D-80337 München, Germany; 2 School of Medicine and Pharmacology, Royal Perth Hospital Unit, University of Western Australia, Perth, Western Australia 6000 Australia; 3 Menzies Institute for Medical Research, University of Tasmania, Hobart, Tasmania 7000 Australia; 4 Telethon Kids institute, The University of Western Australia, Perth, Western Australia 6009 Australia

**Keywords:** Metabolic syndrome, Sex differences, Metabolomics, Raine study, Sphingolipids, Hormonal contraceptives

## Abstract

**Background:**

There are differences in the prevalence and severity of diseases between males, females not taking hormonal contraceptives (non-HC females) and females taking hormonal contraceptives (HC females). The aim of this study was to identify sex-specific differences in the metabolome and its relation to components of the metabolic syndrome in a young adult population.

**Methods:**

The subjects analysed are from the 20-year follow-up of the Western Australian Pregnancy Cohort (Raine) Study. Two hundred fifteen plasma metabolites were analysed in 1021 fasted plasma samples by a targeted liquid chromatography coupled to tandem mass spectrometry (LC-MS/MS) metabolomics approach. Principal component analysis between males (*n* = 550), non-HC females (*n* = 199) and HC females (*n* = 269) was applied. Regression analysis with a sex × metabolite concentration interaction was performed on components of the MetS, ﻿namely waist circumference, systolic blood pressure, and plasma HDL-C, triglycerides and glucose concentration, as outcome to select the significant metabolites of the interaction. Those selected metabolites were used as predictors in a sex group stratified analysis to compare the different β coefficients and therefore the sex group-dependent associations.

**Results:**

Principal component analysis between males, non-HC females, and HC females showed a general discriminating trend between males and HC females. One hundred twenty-seven metabolites were significantly different between males and non-HC females, whereas 97 differed between non-HC females and HC females. Males and non-HC females mainly differed in sphingomyelin, lyso-phosphatidylcholine, acyl-carnitine and amino acid species, whilst non-HC females and HC females mainly differed in phosphatidylcholine, lyso-phosphatidylcholine and acyl-carnitine concentrations. Forty-one metabolites (phosphatidylcholines, sphingomyelines, lyso-phosphatidylcholine) were significantly differently associated with the MetS factors in the different groups.

**Conclusions:**

We have shown clear differences between plasma metabolite concentrations in males, and HC or non-HC females, especially in lyso-phosphatidylcholine, sphingomyelin and phosphatidylcholine, which have been shown to associate with obesity in other studies. The association of these metabolites differed between sexes with components of the metabolic syndrome, which means that development of diseases like obesity and diabetes may differ between the sexes. Our findings highlight the importance of considering sex differences when conducting a metabolomics study and the need to account for the effect of HC usage in females in future studies.

**Electronic supplementary material:**

The online version of this article (doi:10.1186/s13293-017-0131-0) contains supplementary material, which is available to authorized users.

## Background

There is increasing evidence from epidemiological studies that sex and oral contraceptive use in females modulate the development and the severity of cardiovascular disease [1–4]. For example, men are more likely to develop coronary heart diseases than women, especially women prior to menopause, but the risk of developing stroke and heart failure is higher in women [1]. Similarly, more females than males are overweight and obese in developing countries, whereas the opposite is found in developed countries [5]. Since males and females differ in the development and severity of obesity and insulin resistance, it is likely that differences in their metabolism, as a result of genetic effects and environmentally induced changes, may play an important role.

Analysis of the metabolome of males and females could provide insights into the sex-related differences of progression and severity of cardiovascular diseases. Most of the metabolomics studies to date have not stratified analyses according to sex, most likely due to their small sample size. Additionally, to our knowledge there are no studies that have examined sex differences in metabolomics and associated these with components of the metabolic syndrome (MetS). In particular, the characterisation of hormonal contraceptive usage in females with regards to its potential effect on metabolomic changes is highly underrepresented in current research, although a large number of women have been using them since their development in the 1960s [3].

This topic is of high relevance in the approach of implementing sex-specific interventional strategies and biomarker tests in future clinical settings. If biomarkers are different between the sexes, the relevance of those general biomarkers is questionable and also critical in terms of false diagnosis.

The aim of this study was to examine the metabolome of males and females at 20 years of age, including females either using (HC) or not using hormonal contraceptives (non-HC), taking part in the Western Australian Pregnancy Cohort (Raine) Study. The study related potential differences in the metabolome to a number of risk factors underlying the MetS [6].

## Methods

Details on the Raine study have been reported previously [7, 8]. Briefly, from 1989 to 1991, 2900 pregnant women were enrolled in this prospective longitudinal cohort study with the purpose to examine the effects of prenatal ultrasound imaging on the offspring [8]. Two thousand eight hundred sixty-eight live births were evaluated and followed serially to 20 years of age. The present study used cross-sectional data from the 20-year follow-up of the cohort, which occurred between March 2010 and April 2012 and included 87% of the active participants. Ethics approval at the 20 year follow-up was obtained from the University of Western Australia Human Research Ethics Committee. Informed and written consent was obtained from participants.

Serum insulin, glucose, lipids and liver function tests were analysed using standardised protocols in the PathWest Laboratory at Royal Perth Hospital, Perth, Western Australia.

MetS components were the ones established by the International Diabetes Federation, including central obesity, raised systolic blood pressure (sysBP), fasting raised triacylglycerol (TG), glucose, and reduced high density lipoprotein cholesterol (HDL-C) [6]. HC use in females was derived from a questionnaire and based on current use of the oral contraceptive pill, implant, injection or any intrauterine HC-device and defined as a binary variable yes/no.

### Metabolomics measurements

Polar lipids (acyl-carnitines (acyl-Carn), diacyl-phosphatidylcholines (PCaa), acyl-alkyl-phosphatidylcholines (PCae), sphingomyelines (SM), lyso-phosphatidylcholines (LPC/LPCa), alkyl-linked lyso-phosphatidylcholines (LPCe)), non-esterified fatty acids (NEFA) and amino acids (AA) were measured as previously reported [7, 9, 10].

The Additional file 1 provides a detailed description of the metabolomics methodology. Briefly, proteins of plasma were precipitated by adding methanol including internal standards. After centrifugation, the supernatant was used for analyses by liquid chromatography (1200, Agilent) coupled to triple quadrupole mass spectrometry (4000QTRAP, Sciex). Metabolites were quantified by comparison to external standards as μmol/L.

### Potential confounding factors

Physical activity was assessed using the short form of the International Physical Activity Questionnaire and asked whether individuals performed more than 10 min of moderate or vigorous physical activity, and time spent walking or sitting in the last 7 days. The number of days, hours and minutes was reported. We created a categorical variable with “less than once a week”, “1 to 3 times” and “4 or more times” more than 10 min of physical activity per week.

Sedentary behaviour assessment was based on hours spent in front of a screen, including TV watching, playing videogames, socializing and non-socializing activities on the Internet.

Smoking cigarettes and drinking alcohol were used as binary variables. Smoking was assessed by asking if the participant was currently smoking. Alcohol consumption was based on any alcohol consumption in the last 7 days.

### Ethnicity was applied as a dichotomous variable of Caucasian versus non-Caucasian.

Diet was based on dietary patterns as reported by Ambrosini et al. and included three variables: a healthy dietary pattern, a western dietary pattern and a dietary misreporting variable of food intake to account for over, under, or plausible dietary reporting [11].

### Statistics

The software R (R Project for Statistical Computing, http://www.r-project.org/, Version 3.0.2) was used for all statistical analyses. To determine if the residuals were normally distributed, we checked the diagnostic plots of all the models. The *R* function crPlots from the car package in *R* [12] was used to check if outliers influence the linearity. We also assessed the boxplots for all variables used, leading to outlier exclusion. Together with our sufficiently large sample size (Central Limit Theorem), the model assumptions were met. Z-scores for each metabolite within each batch have been calculated to account for batch variation. A batch was defined as 81 samples together with standards and 6 quality control samples. All samples were measured in 15 batches.

We defined a three-level sex variable with the categories males, non-HC females and HC females.

The analysis was divided in three parts. First of all, we aimed at general sex differences.

### General sex differences: principal component analysis

To determine whether the variance in the metabolomics data could be explained in part by the sex categories, we applied principal component analysis (PCA), which is used to find components that explain most of the variance in the data. The components explaining most of the variance (component 1 and component 2) were plotted against each other to see if they showed discriminating clusters for the sex categories. To assess which metabolite groups drive that discrimination, the loadings of the metabolite classes were depicted as arrows in the same plot.

### Sex differences in the associations with the components of the MetS: analysis of variance

In the second analysis, we aimed to extract those metabolites that were significantly associated with at least one of the components of the MetS, depending on the sex variable.

Therefore, the metabolites that were significantly different between the three levels of the sex variable in association with the five continuous factors of the MetS needed to be identified. This pre-selection of metabolites was performed by applying a sex and metabolite interaction variable into analysis of variance (Anova) models with one of the five MetS indicators per model as outcome adjusted for the above-mentioned confounders. A significant interaction meant that the association between the metabolite and the single MetS factor was different depending on at least one of the three sex categories. To indicate which sex groups differed, we performed regression analysis to compare the three group differences after the anova.

### Regression models stratified by sex group

The third analysis aimed to determine if the effect size of the metabolite in association with the single component of the MetS was significantly different between the three sex categories.

The metabolites that were significant in the interaction with at least one of the factors associated with the MetS were used to analyse if the effect sizes are significantly different between the sex categories. Therefore, we stratified the data into a male and non-HC and HC female subset. In all analyses, one of the five factors associated with the MetS was the outcome per model and the identified metabolites the predictor, adjusted for confounding variables.

**Fig. 1:**
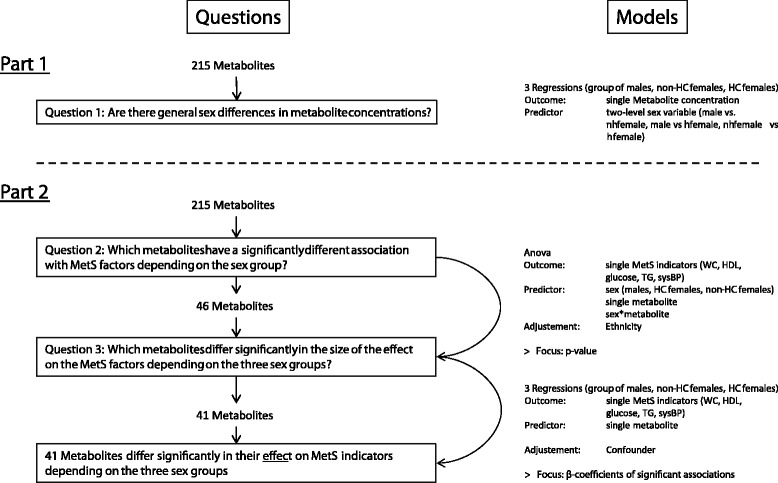
Overview of the analytical strategy

The resulting standardized β coefficients were compared between the three sex categories. Significance for the difference of the β coefficients was tested by applying a regression model with dummy variables (male and non-HC female, HC female, each: yes (1), no (0)), to determine if they were significantly different to the reference category. This difference can be interpreted as the difference in the association between the metabolite and the outcome, if the respective sex category is added. It is a way to perform a significance test for the difference of β coefficients in different categories (for an overview of the analysis strategy, see Fig. 1).

To determine if the metabolites that showed up significantly in the stratified groups were of any significance in the general, unstratified population, we performed analyses with the whole data set, where a single one of the five MetS indicators was the outcome and metabolite concentration was the predictor, adjusted for the confounders stated above (Additional file 1: Table S1).

We used false discovery rate as described by Benjamini and Hochberg to account for multiple testing for the *p* values [13]. This was performed using the *p.adjust* function of the R core package. The confidence intervals were corrected for multiple testing using the formula 1—$\frac{\# of\; selected\; metabolites\times 0.05}{\# of\; all\; metabolites}$, according to Benjamini and Yekutieli [14]. The correction procedure for multiple testing was applied using the number of metabolites. This approach was used, as the main aim of this study was hypothesis generation, and the Bonferroni correction is very restrictive in this regards.

## Results

The study characteristics of the participants are shown in Table 1. Results for all models and metabolites can be found in the Additional file 1: Table S1 which shows that there were significant differences in the concentrations of HDL-C, glucose and triglycerides, sysBP and waist circumference (WC) between males and non-HC and HC females.

**Table 1:** Study participant characteristics stratified by sex and hormonal contraceptive (HC) use

	Females not taking HC	Females taking HC	Males	*p* value
*n* (%)	199 (19.55)	269 (26.42)	550 (54.03)	
Age (years; mean ± sd) Age (years; mean ± sd) Age (years; mean ± sd)	20.1 (0.6)	20.01 (0.61)	20.07 (0.50)	
Waist circumference (cm; mean ± sd)	78.63 (±13.64)	76.09 (±11.96)	82.46 (±11.25)	<0.001a <0.001b <0.05c
Body mass index (BMI, kg/m^2^)	24.88 (6.09)	23.85 (6.09)	24.32 (4.17)	0.0504c
Glucose (mmol/L; mean ± sd)	4.87 (±0.38)	4.83 (±0.36)	5.05 (±0.44)	<0.001a <0.001b
Triglycerides (mmol/L;mean ± sd)	0.85 (±0.38)	1.16 (±0.49)	1.08 (±0.56)	<0.001a <0.05b <0.001c
HDL-C (mmol/L; mean ± sd)	1.41 (±0.32)	1.42 (±0.30)	1.23 (±0.26)	<0.001a <0.001b
LDL-C (mmol/L; mean ± sd)	2.51 (±0.64)	2.59 (±0.62)	2.43 (±0.67)	0.001b
Systolic blood pressure (mm Hg; mean ± sd)	110.92 (±11.69)	111.7 (±10.65)	123.2 (±12.4)	<0.001a <0.001b
Diastolic blood pressure (mm Hg; mean ± sd)	65.16 (±8.16)	65.74 (±7.35)	65.48 (±8.27)	
Western diet (mean ± sd)	-0.25 (±0.79)	-0.4 (±0.68)	0.41 (±0.94)	<0.001a <0.001b <0.05c
Healthy diet (mean ± sd)	0.01 (±0.84)	-0.01 (±0.80)	0.03 (±0.96)	
Misreporting (*n*/%)				<0.01a <0.001b
Underreporting	77 (38.7)	116 (43.12)	131 (23.82)	
Plausible reporting	80 (40.2)	109 (40.52)	222 (40.36)	
Over-reporting	8 (4.02)	11 (4.09)	33 (6)	
NA	34 (17.09)	33 (12.26)	164 (29.82)	
Physical Activity (in the last 7 days, *n*/%)				<0.001a <0.001b
Less than once	39 (20)	55 (20.45)	36 (6.55)	
1 to 3 times	76 (38.19)	122 (45.35)	137 (24.91)	
More than 4	59 (29.65)	73 (27.14)	244 (44.36)	
NA	25 (12.56)	19 (7.06)	133 (24.18)	
Sedentary behaviour (hours per day, *n*/%)				<0.05a <0.05b
0	/	1 (0.37)	/	
1	26 (13.07)	42 (15.61)	37 (6.73)	
2	80 (40.2)	113 (42.01)	204 (37.09)	
3	47 (23.62)	74 (27.51)	142 (25.82)	
4	24 (12.06)	21 (7.81)	41 (7.45)	
NA	22 (11.06)	18 (6.69)	126 (22.91)	
Smoking (currently, *n*/%)				
No	154 (77.39)	217 (80.67)	355 (64.55)	
Yes	23 (11.56)	33 (12.27)	68 (12.36)	
NA	22 (11.06)	19 (7.06)	127 (23.09)	
Alcohol consumption ((in the last 7 days, *n*/%)				<0.001a <0.01c
No	103 (51.76)	110 (40.89)	158 (28.37)	
Yes	70 (35.18)	137 (50.93)	258 (46.91)	
NA	26 (13.07)	22 (8.18)	134 (24.36)	
Ethnicity (*n*/%)				
Caucasian	159 (79.9)	234 (86.99)	452 (82.18)	
Not Caucasian	34 (17.09)	33 (12.27)	84 (15.27)	
NA	6 (3.02)	2 (0.74)	14 (2.55)	

Data expressed as mean ± standard deviation · Superscript letters are statistical significance between groups (*t* test for continuous, Chi^2^ test for categorial variables): a: male vs female, b: male vs hormonal, c: female vs hormonal

### General sex differences: principal component analysis

**Fig. 2:**
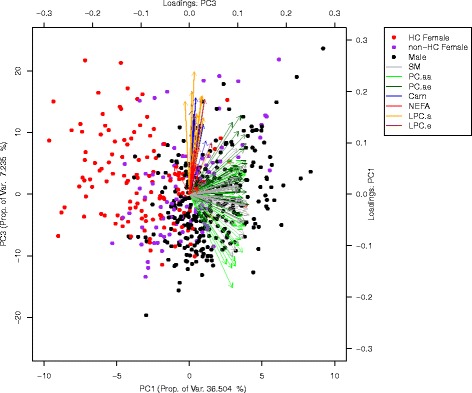
Loadings plot of the first two components of the PCA. *Arrows*: metabolite groups that separate the data set into the different directions. *Points*: loading of the metabolite in the respective component according to male and HC female or non-HC female

PCA (Figure Fig. 2) showed a clustering effect between males and HC females, predominantly due to differences in SM, PC and LPC.

In the regression, there were 127 metabolites significantly different between males and non-HC females and 97 metabolites that differed significantly between HC and non-HC females.

Males and HC females, however, significantly differed in 161 metabolites (Additional file 1: Table S2–Table S4, Table 2).

**Table 2:** Overview of number of significantly sex group between different metabolite groups

	Males vs hfemales	Males vs nhfemales	hfemales vs nhfemales
AA	15 ↑; 1 ↓	15 ↑ ; 2↓	4 ↑; 12 ↓
Carn	12 ↑	11 ↑	8 ↓
LPCa	13 ↑	11 ↑	13 ↓
LPCe	3 ↑	3 ↑	3 ↓
NEFA	2 ↑; 16 ↓	3 ↑; 6↓	6 ↑
PCaa	32 ↓	20 ↓	20 ↑; 2 ↓
PCae	3 ↑; 24 ↓	1 ↑, 20↓	8 ↑; 2 ↓
SM	1 ↑; 39 ↓	1 ↑; 35↓	15 ↑; 4↓

Arrows: ↑ meaning higher and ↓ meaning lower in the firstly mentiones group in the group comparison

### Sex differences in the associations with components of the MetS: analysis of variance

In the Anova, we found that 46 metabolites showed a sex interaction with at least one component of the MetS (Additional file 1: Table S5, group differences Additional file 1: Table S5). These 46 metabolites were included in the stratified analysis to examine the standardized β values. All of the 46 metabolites were significantly associated with the indicators of the MetS in the unstratified analysis of the general Raine study population.

Those metabolites were acyl-Carn C3:0, NEFA C24:5, NEFA C26:1, PCaa C32:3, PCaa C34:1, PCaa C34:2, PCaa C34:3, PCaa C36:0, PCaa C36:1, PCaa C36:2, PCaa C36:3, PCaa C36:4, PCaa C38:0, PCaa C38:3, PCaa C38:4, PCaa C38:5, PCaa C38:6 , PCaa C40:6, PCaa C40:4, PCaa C40:5, PCae C36:1, PCae C38:0, PCae C40:0, PCae C40:6, SM C36:0, SM C38:1, SM C38:2, SM C40:3, SM C40:4, SM C40:5, SM C41:0, SM C42:4, SM C42:6, SM C44:6, LPCa C16:0, LPCa C16:1, LPCa C18:0, LPCa C18:1, LPCa C18:3, LPCa C20:3, LPCa C20:4, LPCa C20:5, LPCa C22:5, LPCa C22:6, LPCe C18:0, and LPCe C18:1 (for sex group differences: Table 3, for *p* values, β-coefficients and confidence interval: Additional file 1: Table S6).

**Table 3:** Results for the group testing after anova

Metabolite	HDL	Triacylglycerol	Waist circumference	Systolic blood pressure	Glucose
Acyl-Carn C3:0	–	males > HC	–	–	–
NEFA C24:5	HC > non-HC HC > males	–	–	–	–
NEFA C26:1	–	males > non-HC	–	–	–
PCaa C32:3	HC > males	–	–	–	–
PCaa C34:1	non-HC > males	HC > non-HC males > non-HC	–	–	–
PCaa C34:2	non-HC > HC	males > non-HC	–	–	males > HC males > non-HC
PCaa C34:3	–	HC > non-HC males > non-HC	–	–	males > HC
PCaa C36:0	HC > males non-HC > males	HC > non-HC HC > males	–	–	–
PCaa C36:1	–	males > non-HC males > HC	–	–	–
PCaa C36:2	–	males > non-HC males > HC	–	–	–
PCaa C36:3	–	males > non-HC	–	–	males > HC
PCaa C36:4	–	males > non-HC	–	–	males > HC
PCaa C38:0	HC > males non-HC > males	HC > non-HC HC > males	–	–	–
PCaa C38:3	HC > non-HC HC > males	males > non-HC males > HC	–	–	–
PCaa C38:4	non-HC > HC HC > males	males > non-HC	–	–	–
PCaa C38:5	HC > males	males > non-HC	–	–	–
PCaa C38:6	HC > males	–	–	–	–
PCaa C40:6	non-HC > HC HC > males	–	–	–	–
PCaa C40:4	HC > males	males > non-HC	–	–	–
PCaa C40:5	HC > males	males > non-HC males > HC	–	–	–
PCae C36:1	–	males > non-HC	–	–	–
PCae C38:0	non-HC > males	HC > non-HC males > non-HC	–	–	–
PCae C40:0	HC > males	–	–	–	–
PCae C40:6	HC > males	–	–	–	–
SM C36:0	HC > non-HC HC > males	males > non-HC males > HC	non-HC > HC	–	–
SM C38:1	non-HC > males	males > non-HC	–	–	–
SM C38:2	–	–	–	–	–
SM C40:3	–	males > non-HC	–	–	–
SM C40:4	–	males > non-HC	–	–	–
SM C40:5	non-HC > HC non-HC > males	HC > non-HC males > non-HC	–	–	–
SM C41:0	HC > non-HC HC > males	–	non-HC > HC non-HC > males	–	–
SM C42:4	non-HC > HC HC > males	males > non-HC	–	–	–
SM C42:6	HC > males non-HC > males	–	–	–	–
SM C44:6	non-HC > HC HC > males	–	–	–	–
LPCa C16:0	non-HC > HC males > HC	males > non-HC males > HC	–	–	–
LPCa C16:1	non-HC > HC males > HC	–	–	–	–
LPCa C18:0	non-HC > HC males > HC	males > non-HC males > HC	–	–	–
LPCa C18:1	non-HC > HC males > HC non-HC > males	males > HC	HC > non-HC males > non-HC	–	–
LPCa C18:3	non-HC > HC males > HC non-HC > males	males > non-HC	HC > non-HC males > non-HC	–	–
LPCa C20:3	non-HC > HC males > HC	–	–	–	–
LPCa C20:4	non-HC > HC males > HC non-HC > males	–	–	–	–
LPCa C20:5	non-HC > HC males > HC	–	HC > non-HC	–	–
LPCa C22:5	non-HC > HC males > HC	–	–	–	–
LPCa C22:6	non-HC > HC non-HC > males	–	males > non-HC	–	–
LPCe C18:0	non-HC > HC	–	–	–	–
LPCe C18:1	non-HC > HC males > HC non-HC > males	males > HC	–	–	–

Direction of the difference in the 46 metabolites found significant in the anova in association with the according MetS factor. Males, HC (hormonal contraceptive-taking females) and non-HC (non-hormonal-taking females) are indicated

In regression models stratified by sex group, we found that 41 of these metabolites had significantly different between-group β coefficients, whereas acyl-Carn C3:0, PCaa C34:2, PCaa C40:4, SMa C38:2 and NEFA C26:1 showed no significant difference (Additional file 1: Table S5). None of the metabolites were significantly different between the sex categories for all MetS factors. For HDL, TAG and WC, LPCa C18:3 was significantly different. LPCa C18:1, LPCa C20:4, LPCa C22:6 and SMa C41:0 shared different associations depending on the sex category for HDL and WC.

HDL and TAG had significant differences in the metabolites LPCa C16:0, LPCa C18:0, LPCa C18:3, LPCe C18:1, PCaa C34:1, PCaa C34:3, PCaa C38:3, PCaa C38:4, SMa C36:0 and SMa C42:4 between the sex categories in common.

For HDL, LPCa C16:1, LPCa C20:3, LPCa C20:5, LPCa C22:5, LPCe C18:0, NEFA C24:5, PCaa C38:0, PCaa C38:6, PCaa C40:5, PCaa C40:6, PCae C40:0, PCae C40:6 and SMa C42:6 were significantly different between males, hormonal contraceptive-taking females and non-hormonal contraceptive-taking females.

**Fig. 3:**
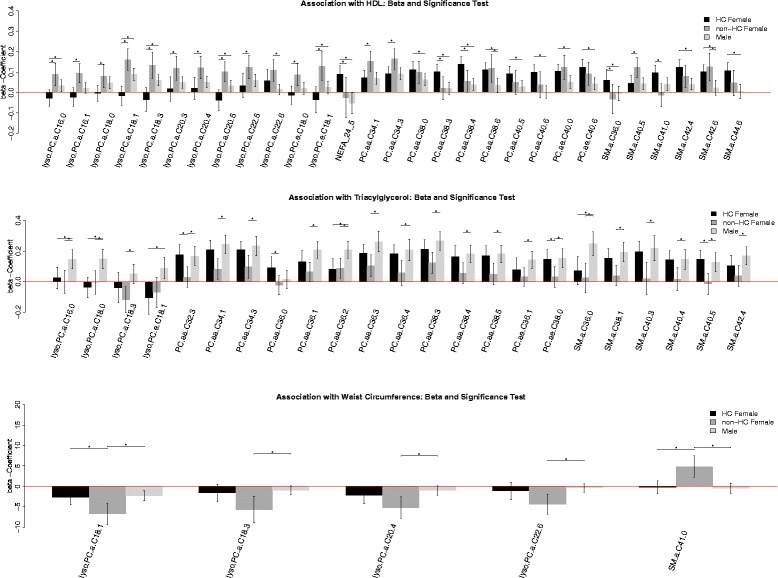
Barplots of the β coefficients from the sex stratified regression models with MetS factors (WC, HDL-C, TG and sysBP) as outcome and single metabolite concentration as predictor, adjusted for ethnicity, physical activity, sedentary behaviour, dietary patterns, smoking, and alcohol consumption. Significance (*): FDR-corrected significant difference of the effect size between the sex groups. Confidence intervals: False coverage rate

TAG was significantly differently associated with PCaa C32:3, PCaa C36:0, PCaa C36:1, PCaa C36:2, PCaa C36:3, PCaa C36:4, PCaa C38:5, PCae C36:1, PCae C38:0, SMa C38:1, SMa C40:3 and SMa C40:4 (Fig. 3).

## Discussion

Our results show significant differences in the concentrations of metabolites in males, HC and non-HC females. In general, males have higher LPC and Carn levels, and non-HC females have higher SM and PC levels. HC females have higher NEFA concentrations than non-HC females and males. The concentrations of HDL-C, glucose and TG, sysBP and WC differed significantly between males, HC and non-HC females and also in their associations with metabolite concentrations.

Numerous epidemiological studies have shown the prevalence of non-communicable diseases differs between males and females, and it is well established that sex hormones have a significant effect on certain diseases [1, 4]. In the field of metabolomics, however, there are few studies addressing sex differences, and how they affect the metabolome underlying diseases risk factors [3, 15, 16]. Most of the metabolomics studies conducted to date have either not taken potential sex differences into account or have used males and females in the same model. There are several studies showing similar results as our study concerning the sex differences in metabolites [3, 15, 17]. However, to our knowledge there is only one other study that has examined sex differences with respect to HC use and metabolomics or that has investigated associations between metabolomics with different components of the MetS [3, 15]. Given a significant number of women of childbearing age are taking HC, this is a large group that is not represented or analysed in many clinical studies.

### General sex differences

In the present study, we found higher WC in males than females which supports data showing obese males have more visceral adiposity than obese females [18]. Our data supports the literature in showing that HDL-C concentrations were higher in women than in men. Higher HDL-C is known to associate with lower cardiovascular risk [17]. Previous reports have shown that lower HDL-C in males associate with lower SM concentrations compared with that in females [19]. Our data are in accordance with these results.

We found LPC and SM concentrations most differentiating between males, non-HC and HC females. This might be due to a suppression of lecithin cholesterol acyltransferase (LCAT) activity by higher SM concentrations. Males, having lower SM concentrations than females, showed higher LPC concentrations, which is in accordance with LPCs being associated with higher LDL and lower HDL. The sex difference may also explain previous findings of decreased LPC concentrations in association with obesity, as LPC are higher in males than in females [20, 21].

Although higher circulating LPC levels in the blood have been predominantly associated with LCAT activity [22], higher levels of LPC have also been associated with phospholipase A_2_ (PLA2) activity. This enzyme mainly associates with LDL and hydrolyses the fatty acid at the sn-2 position from phospholipids, leading to a LPC and a non-esterified fatty acid. This reaction by LPC by PLA2 is thought to play an important role in the early onset of cardiovascular diseases [23]. There is some evidence that PLA2 activity is increased in males compared with females, in accordance with our finding that LPCs were associated with male sex [24].

The difference in PC and SM levels between males and HC and non-HC females is likely related to the different distribution of lipoprotein species and their associated metabolic consequences. This may also explain some of the differences associated with HDL which is enriched in PC containing polyunsaturated fatty acids [25].

Serine was significantly higher in females. Together with palmitoyl-CoA concentrations, serine is the initial and rate-limiting metabolite in the metabolism of sphingolipids. We found that AA and especially branched chain amino acids (BCAA) concentrations were higher in males than in HC or non-HC females. BCAA and associated metabolites have been shown to associate with insulin resistance, cardiovascular disease and female sex hormones [26].

BCAA have been shown to be important in muscle metabolism, which could explain the significantly positive association of leucine with male sex rather than HC or non-HC females, since males have a higher percentage of lean body mass including muscle mass than females [27]. Testosterone is long known as an anabolic hormone linked to AA metabolism [28]. In a study in elderly men, testosterone supply led to increased strength and lean body weight [28]. In general the differences between males and females in the metabolome likely reflect differences in lipid and AA metabolism.

### Hormones and hormonal contraceptives

The majority of HC provide the two hormones estrogen and progesterone, which influence AA and lipid metabolism [28–39]. We found that HC females have decreased free Carn and acyl-Carn levels together with decreased AA levels. Previous reports show female free Carn and acyl-Carn levels decrease significantly upon reaching fertile age [30]. It was suggested that estrogen levels might be the reason.

Furthermore, estrogen is associated with an increased availability of fatty acids by lipolysis and also decreases carbohydrate metabolism [19, 31].

A recent study showed that a decrease in estrogen enhances the accumulation of visceral fat in women through Aldehyde dehydrogenase 1 family, member A1 (aldh1a1), but the effect of the enzyme was not relevant in men, supporting sex differences in lipid metabolism driven by hormones [32].

Our observation of reduced concentrations of some NEFA species in males compared to non-HC females and especially HC females could be due to the higher testosterone levels in males [33]. Higher insulin concentrations are known to associate with lower NEFA levels that associate with a lower β-oxidation in the fasting state and therefore also lower acyl-Carn levels, as carrier of fatty acids.

Estrogen and estradiol have been shown to increase HDL-C blood concentrations, which is a protective marker for cardiovascular diseases. This could, in part, explain the increased risk of cardiovascular diseases in women after menopause [34]. In accordance with our findings, HC use has been associated with higher HDL-C and low-density lipoprotein-cholesterol (LDL-C) (higher than in males in our data), TG (lower in HC females compared to that in males and non-HC females in our data) concentrations [34]. This could explain the higher PC and SM concentrations in HC females compared to those in males and non-HC females, as they are the most abundant phospholipids in lipoproteins [35].

Some estrogen compounds have been shown to not only decrease concentrations of stearic acid but also, amongst others, increase palmitic acid in PC in the serum [38]. The lower levels of LPCa C16:0, LPCa C18:0 and LPCa C18:1 in HC females compared to non-HC females could be a reflection of that, since they potentially occur after cleaving a fatty acid from PCs by LCAT activity.

### MetS dependent differences

Males in our study have a more adverse metabolic profile than non-HC females and HC females, with respect to WC, sysBP and TG, HDL-C and glucose concentrations and also differences in the relationships between these components and different metabolites, for example LPC concentrations between males and either HC or non-HC females.

HC and non-HC females mainly differed in SM and PC concentrations. WC, TG and HDL were associated with increased LPC concentrations that are also differently elevated between males and HC and non-HC females. This is in keeping with the finding that LPC concentrations have been associated to WC in other studies and highlights the importance of our findings.

HDL levels are associated with LCAT, an enzyme that leads to the formation of LPC [36]. In our study, males had higher levels of LPC and lower levels of HDL-C than females.

HDL is known to contain a lower SM/PC ratio than LDL, which could be a reason for HDL being mainly associated with PC and LPC species in our study [37]. The β coefficient for the association between LPC levels and components of the MetS was higher in females than in males; irrespective of whether it was a positive (sysBP, TG, HDL) or negative (TG, WC, HDL) association. This suggests a more negative effect of LPC in females than in males, possibly due to the fact, that males already have higher levels of LPC.

In our study SM had a strong association with high levels of components of the MetS in males and in HC or non-HC females. But in general, SM concentrations were higher in females (HC and non-HC) than in males.

To our knowledge there are no other studies that have performed a comprehensive metabolomics analysis examining sex differences in relation to components of the MetS. Newbern et al. reported sex differences in insulin resistance in adults in their metabolomics study, although these were mainly in BCAAs and their by-products short-chain acyl-Carn [15].

Because of the young age of 20 years of the participants, the analysis of this cohort is valuable for future lifestyle and other prevention strategies in this age group. Additionally, the sample size is exceptionally high with more than 1000 participants. Studies like ours provide some insight into the possible mechanisms for the differences in the prevalence of diseases between sexes. These data are also important in assisting intervention and metabolomics studies conducted in the future.

The limitations of our study are that we do not have blood sex hormone concentrations or any information on the time of the estrous cycle, so the focus of this study is hypothesis generation. It is an observational cohort study with a cross-sectional analysis, which does not allow for interpretations of the directions of associations. Additionally, we had only plasma and no cell samples to measure and interpret metabolic changes, and we did not have separated lipoprotein species.

## Conclusions

This study has shown clear differences between plasma metabolite concentrations in males and HC or non-HC females, especially in LPC, SM and PC, which have been shown to associate with obesity in other studies [7, 21, 38]. Further, the association of these metabolites differed between sexes with components of the MetS, which means that development of diseases like obesity and diabetes may differ between the sexes, potentially mediated by sex hormones. Our findings highlight the importance of considering sex differences when conducting a metabolomics study, and the need to account for the effect of HC usage in females in future studies. The latter finding could be of importance for early programming or pregnancy studies, as the use of HC leads to a hormonal state in the body similar to pregnant women [29, 39]. To our knowledge this is the first comprehensive analysis demonstrating differences in metabolomics between males and females, as well as the effect of HC use. Additionally, we show that the association between metabolomics markers and components of the MetS are significantly different between males and females.

## Additional file

Supplemental information is available on the website of Biology of Sex Differences.

Additional file 1: Table S1. Results for the regression model of the unstratified population with the five MetS. **Table S2–S4.** Results of the Regression model with Metabolite concentration as outcome and two-level sex variable as predictor. **Table S5.** Results for the ANOVA for the five MetS Factors. **Table S6**: Results for the group testing after the Anova. **Table S6.1.** Results for the testing of hfemales versus nhfemales. **Table 6.2.** Results for the testing of males versus hfemales. **Table S6.3.** Results for the testing of males versus nhfemales. **Table 7.** Median, 25% and 75% quartile for every metabolite (215) of the Raine Study metabolomics dataset stratified by males and non-hormonal and hormonal contraceptive-taking females. **Table S7.1.** Non-hormonal contraceptive-taking females. **Table S7.2.** Hormonal contraceptive-taking females. **Table S7.3.** Male subset. (DOCX 530 kb)

## Abbreviations

AA: Amino acids
acyl-Carn: Acyl-Carnitine
aldh1a1: Aldehyde dehydrogenase 1 family, member A1
Anova: Analysis of variance
BCAA: Branched chain amino acids
Carn: Free carnitine
CoA: Coenzyme A
HC females: Hormonal contraceptive-taking females
HDL-C: High-density lipoprotein cholesterol
LCAT: Lecithin cholesterol acyltransferase
LC-MS/MS: Liquid chromatography-tandem mass spectrometry
LDL-C: Low-density lipoprotein cholesterol
LPC/LPCa: Lyso-phosphatidylcholines
LPCe: alkyl-linked lyso-phosphatidylcholines
MetS: Metabolic syndrome
NEFA: Non-esterified fatty acids
Non-HC females: Non-hormonal contraceptive-taking females
PCA: Principal component analysis
PCaa: Diacyl-phosphatidylcholines
PCae: Acyl-alkyl-phosphatidylcholines
SM: Sphingomyelines
sysBP: Systolic blood pressure
TG: Triacylglycerol
WC: Waist circumference
