# Supplementary material for: Sex differences in the association of phospholipids with components of the metabolic syndrome in young adults
Source: Biol Sex Differ. 2017 Mar 28;8:10. doi: 10.1186/s13293-017-0131-0 (PMC5371176; doi:10.1186/s13293-017-0131-0)
Supplement: Additional file 1: Table S1. — Results for the regression model of the unstratified population with the five MetS. Table S2–S4. Results of the Regression model with Metabolite concentration as outcome and two-level sex variable as predictor. Table S5. Results for the ANOVA for the five MetS Factors. Table S6: Results for the group testing after the Anova. Table S6.1. Results for the testing of hfemales versus nhfemales. Table 6.2. Results for the testing of males versus hfemales. Table S6.3. Results for the testing of males versus nhfemales. Table 7. Median, 25% and 75% quartile for every metabolite (215) of the Raine Study metabolomics dataset stratified by males and non-hormonal and hormonal contraceptive-taking females. Table S7.1. Non-hormonal contraceptive-taking females. Table S7.2. Hormonal contraceptive-taking females. Table S7.3. Male subset. (DOCX 530 kb) [file 13293_2017_131_MOESM1_ESM.docx]

**Supplemental Material**

**Sex Differences in the Association of Phospholipids with Components of the Metabolic Syndrome in Young Adults**

Sebastian Rauschert 1, Olaf Uhl 1, Berthold Koletzko 1,*, Trevor A. Mori 2, Lawrence J. Beilin 2, Wendy H. Oddy 3,4, Christian Hellmuth 1

1 Ludwig-Maximilians-Universität München, Division of Metabolic and Nutritional Medicine, Dr. von Hauner Children´s Hospital, Munich, Germany

2 School of Medicine and Pharmacology, Royal Perth Hospital Unit, University of Western Australia, Perth, Western Australia, Australia 6000

3 Menzies Institute for Medical Research, University of Tasmania, Hobart, Tasmania, Australia 7000

4 Telethon Kids institute, The University of Western Australia, Perth, Western Australia, Australia 6009

*Corresponding author:

Berthold Koletzko, MD PhD (Dr med Dr med habil), Professor of Pediatrics

Ludwig-Maximilian Universität München

Dr. von Hauner Children’s Hospital

University of Munich Medical Center

Lindwurmstr. 4, D-80337 München, Germany

Phone: +49 89 44005 2826

Fax: +49 89 44005 7742

E-Mail: [office.koletzko@med.lmu.de](mailto:office.koletzko@med.lmu.de)

Inhalt

[**Metabolomics measurements** 4](#_Toc475008004)

[**Tables** 7](#_Toc475008005)

[**Supplemental Table 1**: Results for the regression model of the unstratified population with the five MetS Factors (waist circumference, HDL, triglyceride, glucose and systolic blood pressure) as outcome and single metabolite concentration as predictor. Adjusted for ethnicity, dietary patterns, dietary misreporting, physical activity, sedentary behaviour smoking and alcohol consumption. P-value, beta coefficient, confidence intervals (CI), number of observations per metabolite and FDR corrected P-values reported. 8](#_Toc475008006)

[Waist circumference 8](#_Toc475008007)

[HDL 13](#_Toc475008008)

[Triglyceride 18](#_Toc475008009)

[Glucose 23](#_Toc475008010)

[Systolic Blood Pressure 28](#_Toc475008011)

[**Supplemental Table 2**: Results of the Regression model with Metabolite concentration as outcome and two level sex variable as predictor (males versus *non-HC* females), adjusted for ethnicity. P-values, beta coefficient, Confidence interval (CI), number of observations per metabolite and FDR corrected p-values reported for all metabolites. 33](#_Toc475008012)

[**Supplemental Table 3**: Results of the Regression model with Metabolite concentration as outcome and two level sex variable as predictor (*HC* females versus *non-HC* females), adjusted for ethnicity P-values, beta coefficient, Confidence interval (CI), number of observations per metabolite and FDR corrected p-values reported for all metabolites. 38](#_Toc475008013)

[**Supplemental Table 4**: Results of the Regression model with Metabolite concentration as outcome and two level sex variable as predictor (*HC* females versus males), adjusted for ethnicity. ethnicity P-values, beta coefficient, Confidence interval (CI), number of observations per metabolite and FDR corrected p-values reported for all metabolites. 43](#_Toc475008014)

[**Supplemental Table 5**: Results for the ANOVA for the five MetS Factors: waist circumference, HDL, triglyceride, glucose and systolic blood pressure as outcome and three level sex variable (males, *nh*females and *h*females) * single metabolite concentration interaction as predictor. Adjusted for ethnicity. P-value, FDR corrected P-values and number of observations per metabolite reported. 48](#_Toc475008015)

[Waist circumference 48](#_Toc475008016)

[HDL 53](#_Toc475008017)

[Triglycerides 58](#_Toc475008018)

[Glucose 63](#_Toc475008019)

[Systolic Blood Pressure 68](#_Toc475008020)

[**Supplemental Table 6**: Results for the group testing after the Anova. MetS Factors as outcome and sex dummy (male versus *nh*female, male versus *h*female, *nh*female vs *h*female) and metabolite interaction as predictor. Adding two dummies at once allows to test for differences between two, rather than one category versus all the others. Metabolites are the 46 significant ones from the anova Models adjusted for ethnicity. P-Value, β-coefficient, FDR p-value and confidence interval reported. 73](#_Toc475008021)

[**Supplemental Table 6.1**: Results for the testing of *h*females versus *nh*females 73](#_Toc475008022)

[**Supplemental Table 6.2**: Results for the testing of males versus *h*females 79](#_Toc475008023)

[**Supplemental Table 6.3**: Results for the testing of males versus *nh*females 86](#_Toc475008024)

[**Supplemental Table 7**: Median, 25% and 75 % quartile for every metabolite (215) of the Raine Study metabolomics data set stratified by males, non-hormonal and hormonal contraceptive taking females. 92](#_Toc475008025)

[**Supplemental Table 7.1**: Non-hormonal taking females. 92](#_Toc475008026)

[**Supplemental Table 7.2**: Hormonal taking females. 97](#_Toc475008027)

[**Supplemental Table 7.3**: Male subset. 102](#_Toc475008028)

# Metabolomics measurements

A phlebotomist visited the home of each participant early in the morning and venous blood samples were taken from an antecubital vein after an overnight fast. Samples were processed and stored at -80˚C. After labelling and packing 1176 plasma EDTA samples in 100µl tubes, they were transported on dry ice from the Royal Perth Hospital Research Unit in Perth, Western Australia, to the Division of Metabolic and Nutritional Medicine of the Dr. von Hauner Children´s Hospital in Munich, Germany, and stored at –80°C until analysis by liquid chromatography coupled to tandem mass spectrometry (LC-MS/MS).

Amino Acids

AA were prepared by derivatization to AA butylester, and determined by LC-MS/MS equipped with 150 x 2.1 mm, 3.5 µm particle size C18 HPLC column (X-Bridge, Waters, Milford, USA) and 0.1% heptafluorobutyric acid as ion pair reagent in water as mobile phase A and in methanol as mobile phase B (1). Mass spectrometry detection was performed with an atmospheric pressure chemical ionization source operating in positive ionization mode.

Non-esterified fatty acids

NEFA were analysed by LC-MS/MS operating in negative electrospray ionization mode as described previously (2).

Isopropanol (200 µl, containing 2 mg/100 ml ^13^C-labelled palmitic acid) and 20 µl plasma was mixed in a 96-deepwell plate. After centrifugation the supernatant was transferred into another 96-well plate for LC-MS/MS analysis. For chromatographic separation an UPLC diphenyl column (Pursuit UPS Diphenyl, 1.9 μm, 100 mm, 3.0 mm; Varian, Darmstadt, Germany) was used with 5 mM ammonium acetate and 2.1 mM acetic acid in water as mobile phase A and acetonitrile/ isopropanol (80:20) as mobile phase B at 40°C with an Agilent 1200 SL series HPLC system (Waldbronn, Germany). The injection volume was set to 10 µL with an eluent flow rate of 700 µL/min. A hybrid triple quadrupole MS (4000 QTRAP, AB Sciex, Darmstadt, Germany) operating in negative electrospray ionization mode was coupled to the HPLC system for identification of NEFA. Fatty acids are separated according to chain length and number of double bonds, but not according to position of double bonds.

Quantification of NEFA has been carried out by comparison of signal-to-internal standard-ratios between samples and dilutions of a commercial available standard mixture. The lipid species are described using the nomenclature CX:Y, where X is the length of the carbon chain (C), Y is the number of double bonds. Metabolite concentrations are reported in µmol/L plasma.

Polar lipids

Polar lipids were analyzed by flow-injection analysis with a LC-MS/MS with electrospray ionization source (3).

Proteins of 10 µL plasma were precipitated by adding 500 µL methanol, including 1-tridecanoyl-2-hydroxy-sn-glycero-3-phosphocholine and 1,2-dimyristoyl-sn-glycero-3-phosphocholine as internal standards with a concentration of approximately 2 µmol/L and ammonium acetate with a concentration of 0.4 g/l. Samples were prepared using a 1.2 mL 96-deepwell plate. The plate was shaken for 30 minutes with 800 rpm and afterwards cooled for 20 minutes at -20°C. After centrifugation at 4000 rpm for 10 minutes, 100 µL of the supernatant was transferred to a 1.0 mL 96 deepwell-plate, prefilled with 350 µL methanol.

A liquid chromatographic system (1200, Agilent, Waldbronn, Germany) was used as flow-injection analyses coupled to a triple quadrupole mass spectrometer (QTRAP4000, Sciex, Darmstadt, Germany) with an electrospray ionization source. Analyses were split in two periods per sample each with 30 µL injection volume to cover the full range of analytes. Samples were injected in 40 µL/min mobile phase with isocratic elution (76% isopropanol, 19% methanol and 5% water) and the flow was diluted by adding a further 200 µL/min mobile phase “post-column” via a T-piece adapter to stabilize the electro spray for ionization. Mass spectrometric analysis was carried out in Multiple Reaction Monitoring mode. Period one was operated with positive and negative ionisation and period two with positive ionisation.

Quantification of metabolites was performed by comparison of signal-to-internal standard-ratios between samples and commercially available lyophilized aliquots of control plasma (Recipe, Germany). The concentrations of the control plasma were determined by AbsoluteIDQ p150 Kit from Biocrates®, a previous published LC-MS/MS method [4] and by in-house quantification with various standards. The entire analytical process was post-processed by Analyst 1.5.1 and the isotopomer correction for up to M+4 was applied by R (programming language, version 3.0.1).

Quality Control

The quality control (QC) criterion was defined as inter- and intra-batch coefficient of variance of 30% and values above 1.5 times the interquartile range were defined as outlier. Further, z-scores for metabolites per batch were used.

1. Harder U, Koletzko B, Peissner W. Quantification of 22 plasma amino acids combining derivatization and ion-pair LC-MS/MS. Journal of chromatography B, Analytical technologies in the biomedical and life sciences. 2011 Mar 1;879(7-8):495-504. PubMed PMID: 21292569. Epub 2011/02/05. eng.

2. Hellmuth C, Weber M, Koletzko B, Peissner W. Nonesterified fatty acid determination for functional lipidomics: comprehensive ultrahigh performance liquid chromatography-tandem mass spectrometry quantitation, qualification, and parameter prediction. Analytical chemistry. 2012 Feb 7;84(3):1483-90. PubMed PMID: 22224852. Epub 2012/01/10. eng.

3. Rauschert S, Uhl O, Koletzko B, Kirchberg F, Mori TA, Huang RC, et al. Lipidomics reveals associations of phospholipids with obesity and insulin resistance in young adults. The Journal of clinical endocrinology and metabolism. 2015 Dec 28:jc20153525. PubMed PMID: 26709969. Epub 2015/12/29. Eng.

4. Uhl O, Glaser C, Demmelmair H, Koletzko B. Reversed phase LC/MS/MS method for targeted quantification of glycerophospholipid molecular species in plasma. Journal of chromatography B, Analytical technologies in the biomedical and life sciences. 2011 Nov 15;879(30):3556-64. PubMed PMID: 22014895. Epub 2011/10/22. eng.

Tables

## Supplemental Table 1: Results for the regression model of the unstratified population with the five MetS Factors (waist circumference, HDL, triglyceride, glucose and systolic blood pressure) as outcome and single metabolite concentration as predictor. Adjusted for ethnicity, dietary patterns, dietary misreporting, physical activity, sedentary behaviour smoking and alcohol consumption. P-value, beta coefficient, confidence intervals (CI), number of observations per metabolite and FDR corrected P-values reported.

### Waist circumference

| Analytes | P-Value | BETA | FDR P-Value | CI lower | CI upper | Number of observations |
| --- | --- | --- | --- | --- | --- | --- |
| Carn | 0.00036183 | 1.58088007 | 0.00228806 | 0.71469855 | 2.4470616 | 757 |
| Carn.a.C10.0 | 0.66588078 | 0.22371168 | 0.72672268 | -0.79370052 | 1.24112388 | 480 |
| Carn.a.C10.1 | 0.37514533 | 0.4983321 | 0.4744485 | -0.60482466 | 1.60148885 | 467 |
| Carn.a.C12.0 | 0.19050003 | -0.65947435 | 0.2989599 | -1.6479726 | 0.32902389 | 474 |
| Carn.a.C12.1 | 0.07088087 | 1.11424383 | 0.14795521 | -0.09527556 | 2.32376321 | 408 |
| Carn.a.C14.0 | 0.07743779 | 0.80104334 | 0.15706721 | -0.0883679 | 1.69045458 | 630 |
| Carn.a.C14.1 | 0.10574007 | 0.72957349 | 0.19104298 | -0.15479316 | 1.61394014 | 689 |
| Carn.a.C14.2 | 0.2695129 | 0.49413102 | 0.37488662 | -0.38382846 | 1.3720905 | 683 |
| Carn.a.C16.0 | 0.04049564 | 0.91238205 | 0.10035271 | 0.03960509 | 1.78515901 | 754 |
| Carn.a.C16.1 | 0.045927 | 0.90452202 | 0.10944375 | 0.01640582 | 1.79263822 | 687 |
| Carn.a.C18.0 | 0.05786634 | -0.93071987 | 0.12695167 | -1.89257833 | 0.03113859 | 606 |
| Carn.a.C18.1 | 0.0562722 | 0.82490344 | 0.12602628 | -0.02211339 | 1.67192027 | 756 |
| Carn.a.C2.0 | 0.03129602 | 0.94386456 | 0.08410806 | 0.08498827 | 1.80274085 | 706 |
| Carn.a.C3.0 | 6.95E-06 | 2.00711941 | 9.34E-05 | 1.1367953 | 2.87744352 | 756 |
| Carn.a.C4.0 | 0.15842469 | 0.66308807 | 0.25804022 | -0.25904465 | 1.58522079 | 656 |
| Carn.a.C4.0.DC | 0.10200087 | 0.93233223 | 0.18584904 | -0.18595786 | 2.05062231 | 417 |
| Carn.a.C5.0 | 0.06901166 | 0.93094902 | 0.14546575 | -0.07278094 | 1.93467899 | 503 |
| Carn.a.C6.0 | 0.00180767 | 1.43751107 | 0.00883291 | 0.53664385 | 2.33837829 | 640 |
| Carn.a.C8.1 | 0.10793109 | 0.76306158 | 0.19337653 | -0.16779586 | 1.69391901 | 576 |
| lyso.PC.a.C14.0 | 0.67965645 | 0.17581925 | 0.7362138 | -0.65973595 | 1.01137445 | 754 |
| lyso.PC.a.C16.0 | 0.0830574 | -0.76100589 | 0.16534576 | -1.62183148 | 0.09981969 | 706 |
| lyso.PC.a.C16.1 | 0.01495541 | 1.06852715 | 0.04728549 | 0.20851321 | 1.92854108 | 757 |
| lyso.PC.a.C18.0 | 0.58056149 | -0.23119537 | 0.66749049 | -1.05222972 | 0.58983897 | 757 |
| lyso.PC.a.C18.1 | 4.50E-06 | -2.02800999 | 6.45E-05 | -2.88921907 | -1.16680091 | 706 |
| lyso.PC.a.C18.2 | 2.08E-06 | -2.05996676 | 3.44E-05 | -2.90507296 | -1.21486056 | 706 |
| lyso.PC.a.C18.3 | 0.00996003 | -1.26663425 | 0.03569012 | -2.22876313 | -0.30450536 | 605 |
| lyso.PC.a.C18.6 | 0.62260907 | -0.24901276 | 0.70084267 | -1.24235693 | 0.74433142 | 548 |
| lyso.PC.a.C20.2 | 0.00683885 | -1.46529388 | 0.02514551 | -2.52526266 | -0.4053251 | 499 |
| lyso.PC.a.C20.3 | 0.09298055 | -0.80299958 | 0.17233465 | -1.74026028 | 0.13426112 | 646 |
| lyso.PC.a.C20.4 | 0.0463227 | -0.86113669 | 0.10944375 | -1.70820015 | -0.01407323 | 757 |
| lyso.PC.a.C20.5 | 0.37439658 | 0.38901964 | 0.4744485 | -0.47031033 | 1.2483496 | 698 |
| lyso.PC.a.C22.5 | 0.22093947 | -0.56552746 | 0.33218172 | -1.47188279 | 0.34082786 | 666 |
| lyso.PC.a.C22.6 | 0.2702671 | -0.46678049 | 0.37488662 | -1.29741289 | 0.3638519 | 717 |
| lyso.PC.e.C16.0 | 0.45389071 | -0.31616098 | 0.54517599 | -1.14446031 | 0.51213835 | 756 |
| lyso.PC.e.C18.0 | 0.04764896 | -0.87089278 | 0.11135355 | -1.7327373 | -0.00904825 | 740 |
| lyso.PC.e.C18.1 | 0.24602805 | -0.59728802 | 0.3526402 | -1.60761758 | 0.41304155 | 548 |
| PC.aa.C30.0 | 0.39979 | -0.355774 | 0.49399339 | -1.18480889 | 0.4732609 | 753 |
| PC.aa.C30.1 | 0.20686477 | -0.64889327 | 0.31997069 | -1.65755925 | 0.3597727 | 538 |
| PC.aa.C30.2 | 0.12700512 | -0.83025723 | 0.2238205 | -1.89743804 | 0.23692358 | 501 |
| PC.aa.C32.0 | 0.02747963 | -0.95245345 | 0.07574514 | -1.79891217 | -0.10599474 | 757 |
| PC.aa.C32.1 | 0.08604688 | 0.76025907 | 0.16725214 | -0.10806643 | 1.62858456 | 665 |
| PC.aa.C32.2 | 0.3889363 | -0.39743258 | 0.48608559 | -1.30267206 | 0.50780691 | 648 |
| PC.aa.C32.3 | 0.13866685 | 0.66047265 | 0.23313763 | -0.21428781 | 1.53523312 | 666 |
| PC.aa.C34.0 | 0.17264647 | -0.65811486 | 0.27293375 | -1.60483055 | 0.28860083 | 547 |
| PC.aa.C34.1 | 0.40628223 | -0.36238661 | 0.49631068 | -1.21862118 | 0.49384795 | 716 |
| PC.aa.C34.2 | 0.00419193 | -1.27298828 | 0.01669011 | -2.14297717 | -0.40299939 | 706 |
| PC.aa.C34.3 | 0.86032556 | -0.07736999 | 0.89357486 | -0.94036005 | 0.78562007 | 706 |
| PC.aa.C34.4 | 0.70717396 | -0.1663548 | 0.75268516 | -1.03554136 | 0.70283175 | 665 |
| PC.aa.C34.5 | 0.07242832 | 0.77830654 | 0.14842342 | -0.07105577 | 1.62766884 | 674 |
| PC.aa.C36.0 | 0.95749141 | -0.02394423 | 0.96196567 | -0.90562312 | 0.85773467 | 705 |
| PC.aa.C36.1 | 0.88355671 | 0.06683623 | 0.91329179 | -0.82880898 | 0.96248143 | 705 |
| PC.aa.C36.2 | 0.22678755 | -0.53473464 | 0.33396796 | -1.40259662 | 0.33312735 | 706 |
| PC.aa.C36.3 | 0.14156174 | -0.63882779 | 0.23412134 | -1.49109309 | 0.2134375 | 716 |
| PC.aa.C36.4 | 0.5644323 | -0.25873223 | 0.65243519 | -1.13989909 | 0.62243463 | 665 |
| PC.aa.C36.5 | 0.01757331 | 1.0704991 | 0.05321496 | 0.18753199 | 1.95346622 | 625 |
| PC.aa.C36.6 | 0.32910692 | 0.42134587 | 0.43409809 | -0.42568999 | 1.26838173 | 750 |
| PC.aa.C38.0 | 0.16658191 | -0.6494705 | 0.26727695 | -1.57039149 | 0.27145049 | 665 |
| PC.aa.C38.1 | 0.00414973 | -1.37491523 | 0.01669011 | -2.31325491 | -0.43657554 | 605 |
| PC.aa.C38.2 | 0.21850264 | -0.54734569 | 0.33083147 | -1.4198794 | 0.32518801 | 715 |
| PC.aa.C38.3 | 4.12E-07 | 2.33589443 | 1.27E-05 | 1.43942678 | 3.23236209 | 648 |
| PC.aa.C38.4 | 0.00206409 | 1.40604322 | 0.00986175 | 0.51354803 | 2.2985384 | 648 |
| PC.aa.C38.5 | 0.03608309 | 0.93896377 | 0.091269 | 0.06112834 | 1.81679921 | 648 |
| PC.aa.C38.6 | 0.69328292 | -0.19156882 | 0.74157128 | -1.14494385 | 0.7618062 | 648 |
| PC.aa.C40.0 | 0.24571313 | -0.58028038 | 0.3526402 | -1.5610733 | 0.40051254 | 605 |
| PC.aa.C40.1 | 0.42638524 | -0.45392472 | 0.51792557 | -1.57457417 | 0.66672473 | 439 |
| PC.aa.C40.3 | 0.01426608 | -1.36045672 | 0.04728549 | -2.44720175 | -0.27371168 | 440 |
| PC.aa.C40.4 | 0.02570967 | 0.94847391 | 0.07469702 | 0.11540766 | 1.78154016 | 706 |
| PC.aa.C40.5 | 1.81E-06 | 2.16894902 | 3.25E-05 | 1.28498162 | 3.05291643 | 648 |
| PC.aa.C40.6 | 0.00016069 | 1.76264029 | 0.00127956 | 0.85072143 | 2.67455916 | 696 |
| PC.aa.C42.0 | 0.34246675 | -0.46397681 | 0.44355633 | -1.42311174 | 0.49515811 | 606 |
| PC.aa.C42.1 | 0.09145537 | -0.9331362 | 0.17098177 | -2.01731911 | 0.1510467 | 498 |
| PC.aa.C42.2 | 0.33530731 | -0.52712105 | 0.4395797 | -1.60110839 | 0.54686629 | 477 |
| PC.aa.C42.4 | 0.01175327 | -1.47621049 | 0.04075731 | -2.62284557 | -0.32957541 | 425 |
| PC.aa.C42.5 | 0.01305095 | -1.27263048 | 0.04453897 | -2.27636049 | -0.26890047 | 555 |
| PC.aa.C42.6 | 0.02646147 | -1.1470398 | 0.07514057 | -2.15948588 | -0.13459372 | 538 |
| PC.aa.C43.4 | 0.48580444 | -0.38748241 | 0.57388986 | -1.47905365 | 0.70408883 | 479 |
| PC.aa.C43.6 | 0.08929256 | -0.76300695 | 0.16840264 | -1.64344143 | 0.11742753 | 706 |
| PC.aa.C44.12 | 0.49093017 | -0.3004267 | 0.57677588 | -1.1562879 | 0.5554345 | 706 |
| PC.ae.C30.0 | 0.0963481 | -0.78099444 | 0.17704992 | -1.70194707 | 0.1399582 | 639 |
| PC.ae.C30.1 | 0.83574092 | -0.10355261 | 0.87225387 | -1.08404713 | 0.87694192 | 581 |
| PC.ae.C32.0 | 0.28227004 | -0.45172894 | 0.38444179 | -1.27589726 | 0.37243939 | 757 |
| PC.ae.C32.1 | 0.26445505 | -0.47575018 | 0.37161984 | -1.31207038 | 0.36057002 | 757 |
| PC.ae.C32.2 | 0.46695003 | -0.30796327 | 0.55774587 | -1.13863371 | 0.52270716 | 756 |
| PC.ae.C34.0 | 0.64778796 | -0.19912559 | 0.71790933 | -1.05453892 | 0.65628775 | 716 |
| PC.ae.C34.1 | 0.01681857 | -1.04359641 | 0.05165703 | -1.89861259 | -0.18858023 | 716 |
| PC.ae.C34.2 | 1.76E-07 | -2.31799639 | 7.24E-06 | -3.18061119 | -1.45538159 | 716 |
| PC.ae.C34.3 | 2.54E-08 | -2.50481382 | 1.36E-06 | -3.37753921 | -1.63208844 | 716 |
| PC.ae.C34.4 | 0.20330646 | -0.5761846 | 0.31674557 | -1.46462927 | 0.31226007 | 663 |
| PC.ae.C36.0 | 0.00176361 | -1.36377324 | 0.00881803 | -2.21662408 | -0.5109224 | 705 |
| PC.ae.C36.1 | 0.14423487 | -0.65150311 | 0.23672136 | -1.52652822 | 0.223522 | 706 |
| PC.ae.C36.2 | 0.00024883 | -1.64897045 | 0.00167185 | -2.52811997 | -0.76982093 | 706 |
| PC.ae.C36.3 | 1.88E-05 | -1.84304742 | 0.0002249 | -2.68325889 | -1.00283594 | 757 |
| PC.ae.C36.4 | 0.68485005 | -0.18280004 | 0.7362138 | -1.0668335 | 0.70123343 | 665 |
| PC.ae.C36.5 | 0.93556426 | -0.03676078 | 0.94880338 | -0.92927149 | 0.85574994 | 665 |
| PC.ae.C36.6 | 0.48200962 | 0.34680682 | 0.57255286 | -0.6214629 | 1.31507653 | 564 |
| PC.ae.C38.0 | 0.9863534 | -0.00763487 | 0.9863534 | -0.88372286 | 0.86845312 | 706 |
| PC.ae.C38.2 | 0.0069004 | -1.22277294 | 0.02514551 | -2.10875969 | -0.33678619 | 706 |
| PC.ae.C38.3 | 0.95471604 | -0.02512792 | 0.96196567 | -0.89362778 | 0.84337195 | 706 |
| PC.ae.C38.4 | 0.63029979 | -0.21699112 | 0.70580445 | -1.10184687 | 0.66786463 | 665 |
| PC.ae.C38.5 | 0.6393673 | -0.21219208 | 0.71224855 | -1.10097341 | 0.67658926 | 665 |
| PC.ae.C38.6 | 0.88963262 | -0.06167162 | 0.91517231 | -0.93390461 | 0.81056136 | 716 |
| PC.ae.C40.0 | 0.04909169 | -0.89788699 | 0.11228419 | -1.79219105 | -0.00358293 | 706 |
| PC.ae.C40.1 | 0.00078646 | -1.46985855 | 0.00469692 | -2.32558838 | -0.61412871 | 706 |
| PC.ae.C40.2 | 0.11579223 | -0.72150694 | 0.20574652 | -1.62116175 | 0.17814787 | 665 |
| PC.ae.C40.3 | 0.21164104 | -0.56385235 | 0.32433732 | -1.44939317 | 0.32168848 | 666 |
| PC.ae.C40.4 | 0.13906785 | -0.64709771 | 0.23313763 | -1.50498431 | 0.2107889 | 706 |
| PC.ae.C40.5 | 0.23483543 | -0.52812695 | 0.34346678 | -1.40020148 | 0.34394759 | 706 |
| PC.ae.C40.6 | 0.26344367 | -0.53522201 | 0.37161984 | -1.47423664 | 0.40379262 | 648 |
| PC.ae.C42.0 | 0.0002197 | -1.72911076 | 0.00157454 | -2.64278794 | -0.81543358 | 665 |
| PC.ae.C42.1 | 0.00631209 | -1.30823968 | 0.0242339 | -2.2456425 | -0.37083686 | 601 |
| PC.ae.C42.2 | 0.66400385 | 0.19381374 | 0.72672268 | -0.68189536 | 1.06952284 | 666 |
| PC.ae.C42.3 | 4.07E-05 | -1.81121551 | 0.00043737 | -2.67228048 | -0.95015054 | 714 |
| PC.ae.C42.4 | 0.08835262 | -0.7460528 | 0.16840264 | -1.60438716 | 0.11228157 | 706 |
| PC.ae.C42.5 | 0.16002603 | -0.6374958 | 0.25868869 | -1.52744545 | 0.25245386 | 666 |
| PC.ae.C42.6 | 0.32677928 | -0.42854927 | 0.43368855 | -1.28592101 | 0.42882247 | 756 |
| SM.a.C30.1 | 0.02907678 | 1.05537651 | 0.07913301 | 0.10792658 | 2.00282645 | 655 |
| SM.a.C32.0 | 0.0068799 | 1.54456736 | 0.02514551 | 0.42668586 | 2.66244885 | 443 |
| SM.a.C32.1 | 0.01483631 | 1.08617714 | 0.04728549 | 0.21304529 | 1.95930899 | 706 |
| SM.a.C32.2 | 1.03E-13 | 3.2731181 | 7.35E-12 | 2.42635939 | 4.11987681 | 716 |
| SM.a.C33.1 | 0.28414618 | 0.46936916 | 0.38444179 | -0.3903873 | 1.32912562 | 706 |
| SM.a.C33.2 | 8.08E-07 | 2.31055894 | 1.74E-05 | 1.40041082 | 3.22070705 | 615 |
| SM.a.C34.0 | 0.36523858 | -0.48954968 | 0.46741843 | -1.55094088 | 0.57184151 | 493 |
| SM.a.C34.1 | 0.22372199 | -0.53309622 | 0.33303898 | -1.39258197 | 0.32638953 | 717 |
| SM.a.C34.2 | 5.57E-07 | 2.16598377 | 1.33E-05 | 1.32393054 | 3.00803701 | 757 |
| SM.a.C34.3 | 0.00307422 | 1.54567481 | 0.01348892 | 0.52488212 | 2.5664675 | 498 |
| SM.a.C35.0 | 0.68363321 | 0.19806801 | 0.7362138 | -0.75615601 | 1.15229203 | 564 |
| SM.a.C35.1 | 0.01484711 | 1.06584253 | 0.04728549 | 0.20896387 | 1.92272118 | 706 |
| SM.a.C36.0 | 4.76E-07 | 2.73585707 | 1.28E-05 | 1.68284266 | 3.78887149 | 498 |
| SM.a.C36.1 | 0.00010692 | 1.71481854 | 0.00095781 | 0.85076684 | 2.57887023 | 717 |
| SM.a.C36.2 | 2.02E-07 | 2.20602349 | 7.24E-06 | 1.38060735 | 3.03143963 | 757 |
| SM.a.C36.3 | 5.65E-05 | 1.75634598 | 0.00057881 | 0.90525506 | 2.60743691 | 716 |
| SM.a.C37.1 | 0.04478623 | 0.90340775 | 0.10819146 | 0.02108407 | 1.78573142 | 666 |
| SM.a.C38.1 | 0.08237314 | -0.75036138 | 0.16534576 | -1.59723726 | 0.09651451 | 755 |
| SM.a.C38.2 | 0.35521282 | -0.4050729 | 0.45730992 | -1.26467871 | 0.45453291 | 757 |
| SM.a.C39.1 | 0.13867518 | 0.68722018 | 0.23313763 | -0.22298518 | 1.59742553 | 666 |
| SM.a.C39.2 | 0.22460769 | 0.55165805 | 0.33303898 | -0.33945989 | 1.442776 | 715 |
| SM.a.C40.1 | 0.00133877 | 1.42929129 | 0.00702037 | 0.55810182 | 2.30048077 | 661 |
| SM.a.C40.2 | 0.30554209 | 0.47520313 | 0.41057218 | -0.43483774 | 1.38524401 | 625 |
| SM.a.C40.3 | 0.78579582 | 0.14034279 | 0.82680606 | -0.87360029 | 1.15428586 | 536 |
| SM.a.C40.4 | 0.78834997 | -0.11518284 | 0.82680606 | -0.9571934 | 0.72682773 | 757 |
| SM.a.C40.5 | 0.0144783 | 1.07438021 | 0.04728549 | 0.21389205 | 1.93486837 | 675 |
| SM.a.C41.1 | 0.00340845 | 1.29638177 | 0.01436897 | 0.43016916 | 2.16259438 | 706 |
| SM.a.C41.2 | 0.24256396 | 0.52322662 | 0.35237331 | -0.35510834 | 1.40156158 | 706 |
| SM.a.C42.1 | 0.00262423 | 1.29931237 | 0.01175434 | 0.45447598 | 2.14414877 | 706 |
| SM.a.C42.2 | 0.08634878 | 0.76045962 | 0.16725214 | -0.10891405 | 1.62983329 | 706 |
| SM.a.C42.3 | 0.07248585 | 0.81026348 | 0.14842342 | -0.07414233 | 1.69466928 | 706 |
| SM.a.C42.4 | 0.05245473 | 0.87083667 | 0.11871333 | -0.00926863 | 1.75094197 | 665 |
| SM.a.C42.5 | 0.92844144 | -0.04906232 | 0.94604223 | -1.12194101 | 1.02381638 | 498 |
| SM.a.C42.6 | 0.53826247 | -0.28818099 | 0.62554827 | -1.20715673 | 0.63079476 | 665 |
| SM.a.C43.1 | 0.00018524 | 1.65855847 | 0.00140805 | 0.79218503 | 2.52493191 | 706 |
| SM.a.C43.2 | 0.00032772 | 1.58412137 | 0.00213511 | 0.72266615 | 2.44557659 | 706 |
| SM.a.C44.2 | 0.13205083 | 0.74791575 | 0.2289591 | -0.22611473 | 1.72194622 | 556 |
| SM.a.C44.6 | 0.05755283 | 0.9291211 | 0.12695167 | -0.02987536 | 1.88811756 | 624 |
| SM.a.C31.1 | 0.13723996 | 0.68481731 | 0.23313763 | -0.21890175 | 1.58853637 | 665 |
| SM.a.C33.3 | 0.33799435 | 0.4224555 | 0.44041688 | -0.44262271 | 1.28753371 | 715 |
| SM.a.C35.2 | 0.00012215 | 1.66350566 | 0.00105046 | 0.81813087 | 2.50888045 | 717 |
| SM.a.C37.3 | 7.25E-05 | -1.73916062 | 0.00070835 | -2.59456932 | -0.88375193 | 717 |
| SM.a.C39.5 | 0.66066264 | 0.21376831 | 0.72672268 | -0.74211848 | 1.1696551 | 598 |
| SM.a.C41.0 | 0.5948787 | 0.24986657 | 0.6803134 | -0.67244836 | 1.17218151 | 605 |
| SM.a.C41.3 | 0.12950752 | 0.7628841 | 0.22637493 | -0.22409184 | 1.74986004 | 550 |
| SM.a.C43.0 | 0.44881343 | -0.40454675 | 0.54210611 | -1.45305072 | 0.64395722 | 539 |
| SM.a.C43.3 | 0.00222239 | 1.47524034 | 0.01016627 | 0.53212023 | 2.41836044 | 616 |
| Ala | 0.03345487 | 0.91071316 | 0.08666022 | 0.07154765 | 1.74987866 | 754 |
| Arg | 0.37956035 | 0.38736796 | 0.477225 | -0.47755996 | 1.25229588 | 752 |
| Asn | 0.00482713 | -1.22966836 | 0.0188697 | -2.08354212 | -0.37579459 | 703 |
| Asp | 0.17223186 | 0.5787043 | 0.27293375 | -0.25275552 | 1.41016411 | 754 |
| Cit | 0.71109963 | -0.16256817 | 0.75313508 | -1.02391991 | 0.69878357 | 754 |
| Gln | 0.92548567 | 0.04156364 | 0.94604223 | -0.83067179 | 0.91379906 | 703 |
| Glu | 1.78E-20 | 4.02274291 | 3.83E-18 | 3.19615278 | 4.84933303 | 754 |
| Gly | 0.31420423 | -0.43477226 | 0.4195895 | -1.28226841 | 0.41272389 | 754 |
| His | 0.28430812 | -0.45207532 | 0.38444179 | -1.28039188 | 0.37624123 | 743 |
| Ile | 3.71E-05 | 1.98958082 | 0.00041945 | 1.05014568 | 2.92901596 | 543 |
| Leu | 9.54E-07 | 2.29777762 | 1.86E-05 | 1.38557902 | 3.20997623 | 703 |
| Lys | 0.04260208 | 0.90882968 | 0.10408463 | 0.03039833 | 1.78726104 | 703 |
| Met | 0.40358507 | 0.39685262 | 0.49583309 | -0.53546594 | 1.32917118 | 703 |
| Orn | 0.00022727 | 1.70952195 | 0.00157624 | 0.80388025 | 2.61516366 | 703 |
| Phe | 8.55E-05 | 1.7980518 | 0.00079897 | 0.90476754 | 2.69133606 | 703 |
| Pro | 0.00113642 | 1.44342607 | 0.00610825 | 0.57609983 | 2.31075231 | 754 |
| Trp | 0.28355377 | 0.46267472 | 0.38444179 | -0.38372293 | 1.30907237 | 754 |
| Ser | 0.5193868 | -0.27248477 | 0.60689219 | -1.10235763 | 0.55738808 | 754 |
| Thr | 0.06493008 | -0.80070613 | 0.1382175 | -1.65109668 | 0.04968443 | 714 |
| Tyr | 1.39E-15 | 3.55111481 | 1.50E-13 | 2.69849035 | 4.40373927 | 703 |
| Val | 2.52E-06 | 2.15716039 | 3.87E-05 | 1.26482649 | 3.04949428 | 703 |
| NEFA_12_0 | 0.60656679 | 0.25520838 | 0.68637821 | -0.71756683 | 1.22798358 | 602 |
| NEFA_14_0 | 0.39112934 | 0.38278556 | 0.48608559 | -0.49301327 | 1.25858438 | 731 |
| NEFA_14_1 | 0.01167826 | 1.09503991 | 0.04075731 | 0.24466754 | 1.94541228 | 750 |
| NEFA_15_0 | 0.01627607 | 1.05145907 | 0.05071528 | 0.19430165 | 1.90861649 | 747 |
| NEFA_15_1 | 0.08577699 | 0.96069591 | 0.16725214 | -0.13573173 | 2.05712355 | 506 |
| NEFA_16_0 | 0.00399577 | 1.36257009 | 0.01652098 | 0.43644412 | 2.28869607 | 641 |
| NEFA_16_1 | 0.03421307 | 0.90693833 | 0.08756918 | 0.06767752 | 1.74619914 | 750 |
| NEFA_17_0 | 0.00013544 | 1.75399874 | 0.00111999 | 0.85676175 | 2.65123572 | 692 |
| NEFA_17_1 | 0.00018992 | 1.71566292 | 0.00140805 | 0.81806831 | 2.61325753 | 679 |
| NEFA_18_0 | 0.0328573 | 1.03816343 | 0.08666022 | 0.08486028 | 1.99146657 | 636 |
| NEFA_18_1 | 0.04060784 | 0.92882237 | 0.10035271 | 0.03982968 | 1.81781506 | 694 |
| NEFA_18_2 | 0.03310715 | 1.00369299 | 0.08666022 | 0.0807195 | 1.92666649 | 643 |
| NEFA_18_3 | 0.08891759 | 0.72676762 | 0.16840264 | -0.11084803 | 1.56438327 | 750 |
| NEFA_20_1 | 0.25520245 | 0.510136 | 0.36336773 | -0.3694778 | 1.3897498 | 665 |
| NEFA_20_2 | 0.13988258 | 0.67399673 | 0.23313763 | -0.22145937 | 1.56945282 | 633 |
| NEFA_20_3 | 0.00111696 | 1.51492644 | 0.00610825 | 0.60638533 | 2.42346755 | 643 |
| NEFA_20_4 | 0.00112209 | 1.52441169 | 0.00610825 | 0.60981411 | 2.43900927 | 643 |
| NEFA_22_6 | 0.0190538 | 1.09980592 | 0.05689678 | 0.18093712 | 2.01867472 | 694 |
| NEFA_24_1 | 0.60633368 | 0.22192588 | 0.68637821 | -0.62318357 | 1.06703533 | 749 |
| NEFA_12_1 | 0.21270494 | 0.56095218 | 0.32433732 | -0.32206381 | 1.44396816 | 688 |
| NEFA_13_1 | 0.02130267 | 1.50059195 | 0.06274073 | 0.2245116 | 2.77667231 | 393 |
| NEFA_14_2 | 0.04841729 | 0.9628656 | 0.11193244 | 0.00671694 | 1.91901427 | 603 |
| NEFA_16_2 | 0.00151743 | 1.45385575 | 0.00776778 | 0.55749939 | 2.35021211 | 659 |
| NEFA_18_4 | 0.00104059 | 1.70271511 | 0.00604664 | 0.68847253 | 2.7169577 | 527 |
| NEFA_19_0 | 0.0627718 | 0.96592172 | 0.1352872 | -0.05164406 | 1.98348749 | 577 |
| NEFA_19_1 | 0.00326016 | 1.38382304 | 0.0140187 | 0.46368209 | 2.303964 | 653 |
| NEFA_20_5 | 7.80E-06 | 2.05907629 | 9.86E-05 | 1.16206055 | 2.95609203 | 653 |
| NEFA_22_4 | 0.00221257 | 1.52495677 | 0.01016627 | 0.55063841 | 2.49927512 | 577 |
| NEFA_22_5 | 0.00069967 | 1.56520895 | 0.00429799 | 0.66290017 | 2.46751772 | 653 |
| NEFA_24_4 | 0.02656132 | -1.2025572 | 0.07514057 | -2.26451216 | -0.14060223 | 399 |
| NEFA_24_5 | 0.02694027 | -1.21643461 | 0.07522283 | -2.2934401 | -0.13942912 | 456 |
| NEFA_26_1 | 0.06292428 | 1.03111843 | 0.1352872 | -0.05577096 | 2.11800782 | 549 |

### HDL

| Analytes | P-Value | BETA | FDR P-Value | CI lower | CI upper | Number of observations |
| --- | --- | --- | --- | --- | --- | --- |
| Carn | 0.00151085 | -0.03405916 | 0.00253776 | -0.05505613 | -0.0130622 | 757 |
| Carn.a.C10.0 | 0.74124478 | -0.00418459 | 0.78506221 | -0.02907238 | 0.02070319 | 480 |
| Carn.a.C10.1 | 0.47284715 | 0.00952595 | 0.53010134 | -0.0165307 | 0.0355826 | 467 |
| Carn.a.C12.0 | 0.74987634 | 0.00407254 | 0.79031085 | -0.02101603 | 0.0291611 | 474 |
| Carn.a.C12.1 | 0.32396329 | -0.01414031 | 0.38061261 | -0.04228975 | 0.01400913 | 408 |
| Carn.a.C14.0 | 0.01580481 | -0.02680479 | 0.02311588 | -0.04855595 | -0.00505363 | 630 |
| Carn.a.C14.1 | 0.68562798 | 0.00441653 | 0.73338316 | -0.01699688 | 0.02582993 | 689 |
| Carn.a.C14.2 | 0.79967215 | -0.00274622 | 0.83057736 | -0.02398619 | 0.01849376 | 683 |
| Carn.a.C16.0 | 0.00870769 | -0.02814559 | 0.01309198 | -0.04915239 | -0.00713879 | 754 |
| Carn.a.C16.1 | 0.40592344 | -0.00908977 | 0.46670343 | -0.03055137 | 0.01237184 | 687 |
| Carn.a.C18.0 | 0.00246703 | -0.03650356 | 0.00408009 | -0.06008341 | -0.01292371 | 606 |
| Carn.a.C18.1 | 0.0689578 | -0.0190248 | 0.09443265 | -0.03953105 | 0.00148145 | 756 |
| Carn.a.C2.0 | 0.06746212 | -0.01949336 | 0.09297664 | -0.04039106 | 0.00140433 | 706 |
| Carn.a.C3.0 | 1.55E-05 | -0.04675822 | 2.83E-05 | -0.06786194 | -0.02565451 | 756 |
| Carn.a.C4.0 | 0.36462328 | -0.01029311 | 0.42375138 | -0.03257201 | 0.0119858 | 656 |
| Carn.a.C4.0.DC | 0.08592424 | -0.0232983 | 0.1154607 | -0.04990349 | 0.00330689 | 417 |
| Carn.a.C5.0 | 0.00121667 | -0.04322413 | 0.00207606 | -0.06932381 | -0.01712445 | 503 |
| Carn.a.C6.0 | 0.64457198 | -0.0051398 | 0.69735603 | -0.02700867 | 0.01672906 | 640 |
| Carn.a.C8.1 | 0.81754417 | 0.00260221 | 0.84505767 | -0.01954233 | 0.02474675 | 576 |
| lyso.PC.a.C14.0 | 0.08525905 | 0.01769284 | 0.11528739 | -0.00246321 | 0.03784889 | 754 |
| lyso.PC.a.C16.0 | 0.82623437 | -0.00234565 | 0.84590662 | -0.02331597 | 0.01862468 | 706 |
| lyso.PC.a.C16.1 | 0.12342427 | 0.01638815 | 0.1618062 | -0.00447187 | 0.03724816 | 757 |
| lyso.PC.a.C18.0 | 0.97100729 | 0.00036801 | 0.97554471 | -0.01950308 | 0.02023909 | 757 |
| lyso.PC.a.C18.1 | 1.36E-02 | 0.02666437 | 2.02E-02 | 0.00550198 | 0.04782677 | 706 |
| lyso.PC.a.C18.2 | 4.83E-03 | 0.02989826 | 7.47E-03 | 0.0091372 | 0.05065932 | 706 |
| lyso.PC.a.C18.3 | 0.02197951 | 0.02740467 | 0.0317154 | 0.00397094 | 0.05083841 | 605 |
| lyso.PC.a.C18.6 | 0.24837738 | -0.01449268 | 0.30170134 | -0.03912976 | 0.0101444 | 548 |
| lyso.PC.a.C20.2 | 2.0928E-05 | 0.05662353 | 3.7812E-05 | 0.03073125 | 0.08251581 | 499 |
| lyso.PC.a.C20.3 | 0.22493163 | 0.0139581 | 0.27793276 | -0.00860691 | 0.0365231 | 646 |
| lyso.PC.a.C20.4 | 0.47339282 | 0.00750696 | 0.53010134 | -0.01303766 | 0.02805157 | 757 |
| lyso.PC.a.C20.5 | 0.87219335 | -0.00169634 | 0.88453571 | -0.02239212 | 0.01899945 | 698 |
| lyso.PC.a.C22.5 | 0.31249521 | 0.01144916 | 0.36915643 | -0.01079264 | 0.03369096 | 666 |
| lyso.PC.a.C22.6 | 0.54828078 | 0.00627586 | 0.60763076 | -0.01423876 | 0.02679049 | 717 |
| lyso.PC.e.C16.0 | 0.06174951 | -0.01906289 | 0.08565254 | -0.03906568 | 0.0009399 | 756 |
| lyso.PC.e.C18.0 | 0.46459033 | -0.00774921 | 0.52572064 | -0.02854138 | 0.01304295 | 740 |
| lyso.PC.e.C18.1 | 0.84582444 | -0.0024872 | 0.86185903 | -0.02760194 | 0.02262753 | 548 |
| PC.aa.C30.0 | 7.7936E-15 | 0.07773781 | 3.5651E-14 | 0.05850525 | 0.09697037 | 753 |
| PC.aa.C30.1 | 6.8248E-11 | 0.08004428 | 1.9307E-10 | 0.05644169 | 0.10364686 | 538 |
| PC.aa.C30.2 | 2.2589E-14 | 0.09751814 | 1.0118E-13 | 0.0731808 | 0.12185548 | 501 |
| PC.aa.C32.0 | 1.6572E-25 | 0.10540948 | 2.2268E-24 | 0.08631387 | 0.12450509 | 757 |
| PC.aa.C32.1 | 2.3619E-15 | 0.08426537 | 1.2695E-14 | 0.06388456 | 0.10464618 | 665 |
| PC.aa.C32.2 | 8.6981E-24 | 0.10903024 | 8.9052E-23 | 0.08858944 | 0.12947104 | 648 |
| PC.aa.C32.3 | 2.7121E-19 | 0.09661952 | 2.1597E-18 | 0.07614985 | 0.11708918 | 666 |
| PC.aa.C34.0 | 2.4744E-09 | 0.07031135 | 6.1148E-09 | 0.04754504 | 0.09307767 | 547 |
| PC.aa.C34.1 | 6.8638E-20 | 0.09453571 | 5.6758E-19 | 0.07481369 | 0.11425773 | 716 |
| PC.aa.C34.2 | 6.9849E-36 | 0.12823256 | 3.7544E-34 | 0.10923302 | 0.1472321 | 706 |
| PC.aa.C34.3 | 7.3904E-30 | 0.11588239 | 1.7655E-28 | 0.09676967 | 0.1349951 | 706 |
| PC.aa.C34.4 | 8.504E-21 | 0.09846654 | 7.6182E-20 | 0.078493 | 0.11844009 | 665 |
| PC.aa.C34.5 | 8.1812E-08 | 0.05750677 | 1.7415E-07 | 0.03668905 | 0.07832449 | 674 |
| PC.aa.C36.0 | 5.2786E-17 | 0.08927331 | 3.661E-16 | 0.06889452 | 0.10965211 | 705 |
| PC.aa.C36.1 | 2.5153E-15 | 0.08584789 | 1.319E-14 | 0.06503512 | 0.10666065 | 705 |
| PC.aa.C36.2 | 5.9554E-28 | 0.11298939 | 9.8494E-27 | 0.09362735 | 0.13235144 | 706 |
| PC.aa.C36.3 | 1.5906E-32 | 0.12009608 | 5.6995E-31 | 0.10123625 | 0.13895591 | 716 |
| PC.aa.C36.4 | 4.8222E-24 | 0.10735479 | 5.1838E-23 | 0.087332 | 0.12737759 | 665 |
| PC.aa.C36.5 | 1.29E-08 | 0.06384472 | 2.9822E-08 | 0.04209936 | 0.08559007 | 625 |
| PC.aa.C36.6 | 1.7641E-13 | 0.07549354 | 6.7728E-13 | 0.05574829 | 0.09523879 | 750 |
| PC.aa.C38.0 | 4.5189E-15 | 0.0894737 | 2.2001E-14 | 0.0675985 | 0.1113489 | 665 |
| PC.aa.C38.1 | 1.0689E-12 | 0.08302578 | 3.5909E-12 | 0.06062662 | 0.10542493 | 605 |
| PC.aa.C38.2 | 1.7956E-13 | 0.07942968 | 6.7729E-13 | 0.05866603 | 0.10019334 | 715 |
| PC.aa.C38.3 | 4.14E-08 | 0.06174215 | 9.08E-08 | 0.03990743 | 0.08357687 | 648 |
| PC.aa.C38.4 | 7.3488E-12 | 0.07530619 | 2.2898E-11 | 0.05412696 | 0.09648542 | 648 |
| PC.aa.C38.5 | 2.287E-15 | 0.08481194 | 1.2608E-14 | 0.06432249 | 0.1053014 | 648 |
| PC.aa.C38.6 | 4.9173E-15 | 0.09074725 | 2.2983E-14 | 0.06854253 | 0.11295197 | 648 |
| PC.aa.C40.0 | 5.3607E-10 | 0.07422263 | 1.3677E-09 | 0.05113312 | 0.09731214 | 605 |
| PC.aa.C40.1 | 7.9107E-08 | 0.07367954 | 1.7008E-07 | 0.04717782 | 0.10018126 | 439 |
| PC.aa.C40.3 | 1.6718E-07 | 0.0714466 | 3.4897E-07 | 0.04505362 | 0.09783957 | 440 |
| PC.aa.C40.4 | 4.2387E-10 | 0.06376576 | 1.098E-09 | 0.04400712 | 0.08352439 | 706 |
| PC.aa.C40.5 | 1.05E-05 | 0.04901424 | 1.96E-05 | 0.0273509 | 0.07067758 | 648 |
| PC.aa.C40.6 | 1.2668E-06 | 0.05490515 | 2.5218E-06 | 0.03285362 | 0.07695669 | 696 |
| PC.aa.C42.0 | 2.8463E-10 | 0.07469423 | 7.7463E-10 | 0.05183395 | 0.09755451 | 606 |
| PC.aa.C42.1 | 1.3892E-10 | 0.08575038 | 3.879E-10 | 0.06006317 | 0.1114376 | 498 |
| PC.aa.C42.2 | 2.4958E-10 | 0.08010739 | 6.8795E-10 | 0.05577623 | 0.10443856 | 477 |
| PC.aa.C42.4 | 5.6839E-07 | 0.07061977 | 1.1638E-06 | 0.04330222 | 0.09793731 | 425 |
| PC.aa.C42.5 | 3.8764E-13 | 0.08901779 | 1.4126E-12 | 0.06552586 | 0.11250972 | 555 |
| PC.aa.C42.6 | 7.9546E-17 | 0.10141312 | 5.1825E-16 | 0.07829958 | 0.12452667 | 538 |
| PC.aa.C43.4 | 3.0257E-08 | 0.07376969 | 6.8477E-08 | 0.04804674 | 0.09949265 | 479 |
| PC.aa.C43.6 | 9.6715E-13 | 0.07655418 | 3.3006E-12 | 0.05588236 | 0.097226 | 706 |
| PC.aa.C44.12 | 4.1322E-10 | 0.06533815 | 1.0834E-09 | 0.04510554 | 0.08557075 | 706 |
| PC.ae.C30.0 | 5.2224E-12 | 0.07538044 | 1.6512E-11 | 0.05434029 | 0.0964206 | 639 |
| PC.ae.C30.1 | 8.2581E-07 | 0.05983334 | 1.6593E-06 | 0.03625671 | 0.08340997 | 581 |
| PC.ae.C32.0 | 4.3591E-15 | 0.07814925 | 2.1795E-14 | 0.05900093 | 0.09729757 | 757 |
| PC.ae.C32.1 | 9.5896E-31 | 0.11383862 | 2.9454E-29 | 0.09531839 | 0.13235885 | 757 |
| PC.ae.C32.2 | 6.1613E-34 | 0.11839212 | 2.6494E-32 | 0.10020281 | 0.13658143 | 756 |
| PC.ae.C34.0 | 2.9155E-12 | 0.07308135 | 9.4973E-12 | 0.05289311 | 0.0932696 | 716 |
| PC.ae.C34.1 | 3.0116E-30 | 0.11666319 | 8.0937E-29 | 0.09755321 | 0.13577317 | 716 |
| PC.ae.C34.2 | 2.14E-42 | 0.1400153 | 2.30E-40 | 0.12119751 | 0.1588331 | 716 |
| PC.ae.C34.3 | 1.52E-47 | 0.14956073 | 3.28E-45 | 0.13078956 | 0.16833191 | 716 |
| PC.ae.C34.4 | 4.0021E-14 | 0.08267849 | 1.7209E-13 | 0.06168509 | 0.10367189 | 663 |
| PC.ae.C36.0 | 1.5811E-24 | 0.10373357 | 1.8885E-23 | 0.08456338 | 0.12290375 | 705 |
| PC.ae.C36.1 | 1.117E-11 | 0.07249452 | 3.4308E-11 | 0.05188955 | 0.09309948 | 706 |
| PC.ae.C36.2 | 4.2951E-20 | 0.0979375 | 3.6938E-19 | 0.07763388 | 0.11824111 | 706 |
| PC.ae.C36.3 | 2.20E-41 | 0.1331108 | 1.5771E-39 | 0.11489648 | 0.15132511 | 757 |
| PC.ae.C36.4 | 4.6049E-15 | 0.08474687 | 2.2001E-14 | 0.06402076 | 0.10547297 | 665 |
| PC.ae.C36.5 | 6.4917E-14 | 0.08200473 | 2.6841E-13 | 0.06099853 | 0.10301094 | 665 |
| PC.ae.C36.6 | 6.112E-06 | 0.05520604 | 1.1629E-05 | 0.03146147 | 0.07895062 | 564 |
| PC.ae.C38.0 | 7.7474E-21 | 0.09845874 | 7.2421E-20 | 0.07847023 | 0.11844725 | 706 |
| PC.ae.C38.2 | 1.3373E-24 | 0.10879283 | 1.6913E-23 | 0.08872201 | 0.12886366 | 706 |
| PC.ae.C38.3 | 8.2764E-14 | 0.07871662 | 3.3574E-13 | 0.05843842 | 0.09899482 | 706 |
| PC.ae.C38.4 | 4.1205E-10 | 0.06821958 | 1.0834E-09 | 0.04711336 | 0.0893258 | 665 |
| PC.ae.C38.5 | 3.3622E-13 | 0.07938997 | 1.2463E-12 | 0.05841608 | 0.10036387 | 665 |
| PC.ae.C38.6 | 2.7956E-18 | 0.09216562 | 2.1466E-17 | 0.07197959 | 0.11235164 | 716 |
| PC.ae.C40.0 | 5.9708E-17 | 0.09061951 | 4.0117E-16 | 0.06989493 | 0.11134408 | 706 |
| PC.ae.C40.1 | 8.8525E-30 | 0.11569865 | 1.9033E-28 | 0.0965871 | 0.1348102 | 706 |
| PC.ae.C40.2 | 5.023E-11 | 0.07372312 | 1.4399E-10 | 0.05206269 | 0.09538355 | 665 |
| PC.ae.C40.3 | 3.7309E-15 | 0.08621972 | 1.9099E-14 | 0.06520685 | 0.1072326 | 666 |
| PC.ae.C40.4 | 3.9969E-12 | 0.07256111 | 1.2826E-11 | 0.05238831 | 0.09273391 | 706 |
| PC.ae.C40.5 | 5.4072E-10 | 0.06618414 | 1.3677E-09 | 0.04554645 | 0.08682183 | 706 |
| PC.ae.C40.6 | 2.5143E-11 | 0.07674247 | 7.508E-11 | 0.05456165 | 0.0989233 | 648 |
| PC.ae.C42.0 | 4.0599E-11 | 0.07499215 | 1.1796E-10 | 0.05306743 | 0.09691687 | 665 |
| PC.ae.C42.1 | 1.5776E-15 | 0.09149543 | 9.1672E-15 | 0.06956661 | 0.11342426 | 601 |
| PC.ae.C42.2 | 6.9712E-13 | 0.07807107 | 2.4571E-12 | 0.05714887 | 0.09899327 | 666 |
| PC.ae.C42.3 | 2.17E-24 | 0.10774744 | 2.455E-23 | 0.08776237 | 0.1277325 | 714 |
| PC.ae.C42.4 | 1.5698E-15 | 0.08300572 | 9.1672E-15 | 0.06303578 | 0.10297565 | 706 |
| PC.ae.C42.5 | 1.7984E-09 | 0.0669481 | 4.496E-09 | 0.04540385 | 0.08849236 | 666 |
| PC.ae.C42.6 | 8.8435E-13 | 0.07440327 | 3.0667E-12 | 0.05432472 | 0.09448182 | 756 |
| SM.a.C30.1 | 1.3598E-12 | 0.08121458 | 4.4976E-12 | 0.0591627 | 0.10326646 | 655 |
| SM.a.C32.0 | 0.00397813 | 0.04077502 | 0.00633554 | 0.01309679 | 0.06845326 | 443 |
| SM.a.C32.1 | 2.0051E-16 | 0.08715483 | 1.2679E-15 | 0.06685565 | 0.10745401 | 706 |
| SM.a.C32.2 | 1.12E-13 | 0.08062424 | 4.46E-13 | 0.059733 | 0.10151547 | 716 |
| SM.a.C33.1 | 8.8093E-09 | 0.06057655 | 2.0813E-08 | 0.04015413 | 0.08099896 | 706 |
| SM.a.C33.2 | 2.01E-06 | 0.05491175 | 3.90E-06 | 0.03244035 | 0.07738314 | 615 |
| SM.a.C34.0 | 0.00277829 | 0.03995007 | 0.00452525 | 0.01384331 | 0.06605683 | 493 |
| SM.a.C34.1 | 1.4368E-17 | 0.08998022 | 1.0297E-16 | 0.06981585 | 0.11014459 | 717 |
| SM.a.C34.2 | 4.04E-18 | 0.08935221 | 3.00E-17 | 0.06965444 | 0.10904998 | 757 |
| SM.a.C34.3 | 2.6202E-09 | 0.07558669 | 6.4016E-09 | 0.05110895 | 0.10006443 | 498 |
| SM.a.C35.0 | 8.2188E-05 | 0.04752269 | 0.00014604 | 0.02399552 | 0.07104986 | 564 |
| SM.a.C35.1 | 0.01003914 | 0.02737427 | 0.01498899 | 0.0065551 | 0.04819344 | 706 |
| SM.a.C36.0 | 4.61E-03 | 0.03827423 | 7.24E-03 | 0.01185036 | 0.0646981 | 498 |
| SM.a.C36.1 | 7.6097E-05 | 0.04321257 | 0.00013634 | 0.0218953 | 0.06452984 | 717 |
| SM.a.C36.2 | 6.88E-06 | 0.04629524 | 1.30E-05 | 0.0262307 | 0.06635978 | 757 |
| SM.a.C36.3 | 6.68E-07 | 0.05280526 | 1.3552E-06 | 0.03213743 | 0.07347308 | 716 |
| SM.a.C37.1 | 0.00210257 | 0.0343375 | 0.00350428 | 0.0125009 | 0.05617409 | 666 |
| SM.a.C38.1 | 4.9136E-27 | 0.10840784 | 7.0428E-26 | 0.08941681 | 0.12739886 | 755 |
| SM.a.C38.2 | 1.1855E-28 | 0.11304359 | 2.124E-27 | 0.09388979 | 0.13219739 | 757 |
| SM.a.C39.1 | 3.7811E-09 | 0.06713646 | 9.1341E-09 | 0.04507365 | 0.08919926 | 666 |
| SM.a.C39.2 | 5.3339E-08 | 0.06046483 | 1.1584E-07 | 0.03887939 | 0.08205028 | 715 |
| SM.a.C40.1 | 0.00586239 | 0.03057157 | 0.00900296 | 0.00885688 | 0.05228625 | 661 |
| SM.a.C40.2 | 9.8833E-28 | 0.12149699 | 1.5178E-26 | 0.10070055 | 0.14229342 | 625 |
| SM.a.C40.3 | 1.5316E-13 | 0.0888104 | 5.9871E-13 | 0.06580824 | 0.11181256 | 536 |
| SM.a.C40.4 | 2.0587E-22 | 0.09796815 | 2.0119E-21 | 0.07885324 | 0.11708306 | 757 |
| SM.a.C40.5 | 1.2057E-08 | 0.06189992 | 2.8177E-08 | 0.04084189 | 0.08295795 | 675 |
| SM.a.C41.1 | 3.1388E-08 | 0.05907968 | 7.0295E-08 | 0.03835615 | 0.07980321 | 706 |
| SM.a.C41.2 | 9.3947E-16 | 0.08548723 | 5.771E-15 | 0.0650896 | 0.10588485 | 706 |
| SM.a.C42.1 | 1.7E-08 | 0.05872628 | 3.8883E-08 | 0.03852459 | 0.07892798 | 706 |
| SM.a.C42.2 | 3.7192E-11 | 0.07026286 | 1.0954E-10 | 0.04974408 | 0.09078163 | 706 |
| SM.a.C42.3 | 2.187E-15 | 0.08512582 | 1.2374E-14 | 0.0645348 | 0.10571684 | 706 |
| SM.a.C42.4 | 3.2151E-14 | 0.08204637 | 1.4107E-13 | 0.06129433 | 0.10279841 | 665 |
| SM.a.C42.5 | 6.054E-13 | 0.09440896 | 2.1693E-12 | 0.06934103 | 0.1194769 | 498 |
| SM.a.C42.6 | 6.445E-14 | 0.08447074 | 2.6841E-13 | 0.06283571 | 0.10610578 | 665 |
| SM.a.C43.1 | 0.02116409 | 0.0249373 | 0.03074513 | 0.00374457 | 0.04613004 | 706 |
| SM.a.C43.2 | 0.18792966 | 0.01417162 | 0.23628583 | -0.00693907 | 0.03528231 | 706 |
| SM.a.C44.2 | 0.00080642 | 0.04050345 | 0.0014096 | 0.01689112 | 0.06411577 | 556 |
| SM.a.C44.6 | 1.1955E-07 | 0.06360416 | 2.52E-07 | 0.04029023 | 0.08691808 | 624 |
| SM.a.C31.1 | 2.1139E-11 | 0.07535463 | 6.4012E-11 | 0.05365119 | 0.09705808 | 665 |
| SM.a.C33.3 | 1.7495E-06 | 0.05152169 | 3.4195E-06 | 0.03054105 | 0.07250233 | 715 |
| SM.a.C35.2 | 0.00089378 | 0.03555121 | 0.00154971 | 0.01462982 | 0.05647261 | 717 |
| SM.a.C37.3 | 1.07E-29 | 0.11771816 | 2.1E-28 | 0.09822695 | 0.13720938 | 717 |
| SM.a.C39.5 | 1.4628E-06 | 0.05703989 | 2.8854E-06 | 0.03401967 | 0.08006011 | 598 |
| SM.a.C41.0 | 3.8582E-08 | 0.06372241 | 8.5517E-08 | 0.04125648 | 0.08618833 | 605 |
| SM.a.C41.3 | 5.2046E-07 | 0.06064684 | 1.0759E-06 | 0.03719731 | 0.08409637 | 550 |
| SM.a.C43.0 | 5.0288E-09 | 0.07464474 | 1.2013E-08 | 0.04998049 | 0.09930898 | 539 |
| SM.a.C43.3 | 0.00357981 | 0.03468632 | 0.00578691 | 0.01139316 | 0.05797949 | 616 |
| Ala | 0.7585496 | 0.00319064 | 0.79555202 | -0.01717944 | 0.02356073 | 754 |
| Arg | 0.29575525 | -0.01117605 | 0.35326321 | -0.03214536 | 0.00979326 | 752 |
| Asn | 0.09259662 | 0.01778358 | 0.12365388 | -0.00294827 | 0.03851543 | 703 |
| Asp | 0.64545977 | -0.00472305 | 0.69735603 | -0.02486823 | 0.01542214 | 754 |
| Cit | 0.12232704 | 0.01640038 | 0.16135162 | -0.00441406 | 0.03721483 | 754 |
| Gln | 0.09536319 | -0.01791055 | 0.12656226 | -0.03896727 | 0.00314616 | 703 |
| Glu | 3.26E-10 | -0.06701455 | 8.76E-10 | -0.08765842 | -0.04637068 | 754 |
| Gly | 0.13873112 | -0.01547263 | 0.17649225 | -0.03596709 | 0.00502183 | 754 |
| His | 0.40552354 | 0.00852305 | 0.46670343 | -0.01158154 | 0.02862763 | 743 |
| Ile | 1.33E-03 | -0.03931936 | 0.0022516 | -0.06325791 | -0.0153808 | 543 |
| Leu | 3.03E-06 | -0.05299485 | 5.81E-06 | -0.07509629 | -0.0308934 | 703 |
| Lys | 0.2002747 | -0.01389933 | 0.2488963 | -0.03518653 | 0.00738786 | 703 |
| Met | 0.98066056 | -0.00027868 | 0.98066056 | -0.02284265 | 0.02228529 | 703 |
| Orn | 0.00275338 | -0.03364038 | 0.00451891 | -0.05562136 | -0.0116594 | 703 |
| Phe | 3.80E-02 | -0.02306067 | 0.05379497 | -0.04484435 | -0.001277 | 703 |
| Pro | 0.00011714 | -0.04128949 | 0.00020644 | -0.06222002 | -0.02035895 | 754 |
| Trp | 0.68430373 | 0.00424703 | 0.73338316 | -0.01625084 | 0.02474489 | 754 |
| Ser | 0.55427715 | -0.00605298 | 0.61112609 | -0.02613816 | 0.0140322 | 754 |
| Thr | 0.00102366 | 0.03507755 | 0.0017607 | 0.01419408 | 0.05596103 | 714 |
| Tyr | 1.37E-02 | -0.02706107 | 2.02E-02 | -0.04856849 | -0.00555364 | 703 |
| Val | 1.18E-05 | -0.04861644 | 2.20E-05 | -0.07024829 | -0.02698459 | 703 |
| NEFA_12_0 | 0.13216628 | 0.01765392 | 0.1701542 | -0.00534282 | 0.04065067 | 602 |
| NEFA_14_0 | 0.00733345 | 0.02874162 | 0.01118221 | 0.00775684 | 0.04972641 | 731 |
| NEFA_14_1 | 0.00801268 | 0.02788893 | 0.01213188 | 0.00729633 | 0.04848154 | 750 |
| NEFA_15_0 | 0.30218056 | 0.01096461 | 0.35894376 | -0.0098837 | 0.03181292 | 747 |
| NEFA_15_1 | 0.02744645 | 0.02791847 | 0.03933991 | 0.00311661 | 0.05272033 | 506 |
| NEFA_16_0 | 0.24565865 | 0.01296593 | 0.30009437 | -0.00894533 | 0.0348772 | 641 |
| NEFA_16_1 | 0.0046821 | 0.02931064 | 0.00729458 | 0.00902634 | 0.04959493 | 750 |
| NEFA_17_0 | 0.70149603 | -0.00429253 | 0.74664182 | -0.02627178 | 0.01768672 | 692 |
| NEFA_17_1 | 0.40934804 | 0.00924724 | 0.46813739 | -0.01274661 | 0.03124109 | 679 |
| NEFA_18_0 | 0.82346515 | 0.00263414 | 0.84590662 | -0.02054345 | 0.02581172 | 636 |
| NEFA_18_1 | 0.29122301 | 0.01160922 | 0.349793 | -0.00997084 | 0.03318929 | 694 |
| NEFA_18_2 | 0.1289579 | 0.01743422 | 0.16702379 | -0.0050864 | 0.03995484 | 643 |
| NEFA_18_3 | 0.00450678 | 0.0293469 | 0.00712469 | 0.00912519 | 0.0495686 | 750 |
| NEFA_20_1 | 0.19611456 | 0.01442789 | 0.2451432 | -0.00746562 | 0.03632139 | 665 |
| NEFA_20_2 | 0.13682361 | 0.01700516 | 0.17510165 | -0.00541261 | 0.03942293 | 633 |
| NEFA_20_3 | 0.33344869 | 0.01099189 | 0.38962755 | -0.0113084 | 0.03329217 | 643 |
| NEFA_20_4 | 0.60237182 | -0.00596196 | 0.65741087 | -0.02842261 | 0.01649869 | 643 |
| NEFA_22_6 | 0.05483709 | 0.02183073 | 0.07705865 | -0.00045375 | 0.04411521 | 694 |
| NEFA_24_1 | 0.14087254 | -0.01536221 | 0.17816233 | -0.03582097 | 0.00509655 | 749 |
| NEFA_12_1 | 0.03617041 | 0.02293424 | 0.05150091 | 0.00148269 | 0.0443858 | 688 |
| NEFA_13_1 | 0.92373386 | 0.00158415 | 0.93240742 | -0.03093102 | 0.03409931 | 393 |
| NEFA_14_2 | 0.50195197 | 0.00797852 | 0.55916929 | -0.01534531 | 0.03130234 | 603 |
| NEFA_16_2 | 0.12518925 | 0.01715073 | 0.16312539 | -0.00478438 | 0.03908585 | 659 |
| NEFA_18_4 | 0.05897082 | 0.0236231 | 0.0823294 | -0.00089839 | 0.04814459 | 527 |
| NEFA_19_0 | 0.59520783 | 0.00668121 | 0.65290654 | -0.01800459 | 0.03136701 | 577 |
| NEFA_19_1 | 0.79216913 | 0.00304392 | 0.82677846 | -0.01963125 | 0.02571909 | 653 |
| NEFA_20_5 | 4.29E-01 | 0.00898333 | 4.88E-01 | -0.01330926 | 0.03127593 | 653 |
| NEFA_22_4 | 0.26639282 | 0.01344677 | 0.32176661 | -0.01029406 | 0.0371876 | 577 |
| NEFA_22_5 | 0.23099678 | 0.01359177 | 0.28379605 | -0.00866956 | 0.03585309 | 653 |
| NEFA_24_4 | 1.3687E-05 | 0.06176958 | 2.5152E-05 | 0.03420344 | 0.08933573 | 399 |
| NEFA_24_5 | 0.00393245 | 0.03958202 | 0.00630952 | 0.01274578 | 0.06641826 | 456 |
| NEFA_26_1 | 0.08193273 | 0.02386886 | 0.11149074 | -0.00303414 | 0.05077186 | 549 |

### Triglyceride

| Analytes | P-Value | BETA | FDR P-Value | CI lower | CI upper | | Number of observations | |  |
| --- | --- | --- | --- | --- | --- | --- | --- | --- | --- |
| Carn | 0.31617954 | -0.01980458 | 0.39753568 | -0.05856725 | | 0.01895809 | | 757 | |
| Carn.a.C10.0 | 0.28252234 | -0.02634555 | 0.36156133 | -0.07446337 | | 0.02177226 | | 480 | |
| Carn.a.C10.1 | 0.13641072 | -0.03537259 | 0.19816423 | -0.08196609 | | 0.0112209 | | 467 | |
| Carn.a.C12.0 | 0.03097746 | -0.0490153 | 0.0569789 | -0.093526 | | -0.0045046 | | 474 | |
| Carn.a.C12.1 | 0.09945133 | -0.04292089 | 0.15164565 | -0.09401816 | | 0.00817637 | | 408 | |
| Carn.a.C14.0 | 0.41424507 | -0.01601513 | 0.49479272 | -0.05451074 | | 0.02248049 | | 630 | |
| Carn.a.C14.1 | 0.14745166 | -0.02917258 | 0.20994773 | -0.068669 | | 0.01032384 | | 689 | |
| Carn.a.C14.2 | 0.00552579 | -0.05545513 | 0.01277467 | -0.09457085 | | -0.01633941 | | 683 | |
| Carn.a.C16.0 | 0.18273806 | -0.02637741 | 0.25024638 | -0.06520659 | | 0.01245178 | | 754 | |
| Carn.a.C16.1 | 0.00717562 | -0.05425787 | 0.0160704 | -0.09376178 | | -0.01475397 | | 687 | |
| Carn.a.C18.0 | 0.02640667 | -0.04971779 | 0.05024278 | -0.09358825 | | -0.00584733 | | 606 | |
| Carn.a.C18.1 | 0.00390388 | -0.0552732 | 0.00932593 | -0.09275624 | | -0.01779016 | | 756 | |
| Carn.a.C2.0 | 0.00124677 | -0.06315799 | 0.00322958 | -0.10141525 | | -0.02490073 | | 706 | |
| Carn.a.C3.0 | 1.22E-02 | 0.0499972 | 2.54E-02 | 0.01094658 | | 0.08904782 | | 756 | |
| Carn.a.C4.0 | 0.04777711 | 0.04196399 | 0.07841281 | 0.00041214 | | 0.08351584 | | 656 | |
| Carn.a.C4.0.DC | 0.6454637 | 0.01237723 | 0.6933273 | -0.04046996 | | 0.06522442 | | 417 | |
| Carn.a.C5.0 | 0.77068389 | 0.00727168 | 0.80046877 | -0.04171825 | | 0.0562616 | | 503 | |
| Carn.a.C6.0 | 0.42154462 | 0.01669731 | 0.5007298 | -0.02407183 | | 0.05746645 | | 640 | |
| Carn.a.C8.1 | 0.07630435 | -0.03601864 | 0.11887996 | -0.07585813 | | 0.00382086 | | 576 | |
| lyso.PC.a.C14.0 | 4.9608E-09 | 0.10926971 | 2.5062E-08 | 0.07302638 | | 0.14551304 | | 754 | |
| lyso.PC.a.C16.0 | 0.0100318 | 0.05050078 | 0.02178623 | 0.01209682 | | 0.08890474 | | 706 | |
| lyso.PC.a.C16.1 | 2.205E-05 | 0.08238672 | 7.0756E-05 | 0.04451188 | | 0.12026156 | | 757 | |
| lyso.PC.a.C18.0 | 0.68281891 | -0.00759126 | 0.7267627 | -0.04404822 | | 0.0288657 | | 757 | |
| lyso.PC.a.C18.1 | 8.63E-05 | -0.07780585 | 2.73E-04 | -0.11648471 | | -0.03912698 | | 706 | |
| lyso.PC.a.C18.2 | 3.23E-09 | -0.11442464 | 1.70E-08 | -0.15188559 | | -0.0769637 | | 706 | |
| lyso.PC.a.C18.3 | 0.5348258 | -0.01390729 | 0.60519762 | -0.0578893 | | 0.03007473 | | 605 | |
| lyso.PC.a.C18.6 | 0.00011062 | -0.09029789 | 0.0003447 | -0.13583807 | | -0.0447577 | | 548 | |
| lyso.PC.a.C20.2 | 0.18012456 | -0.03252199 | 0.24824859 | -0.08012754 | | 0.01508356 | | 499 | |
| lyso.PC.a.C20.3 | 0.95917103 | -0.00109629 | 0.96365314 | -0.04313185 | | 0.04093926 | | 646 | |
| lyso.PC.a.C20.4 | 8.7718E-06 | -0.0848546 | 2.9468E-05 | -0.12206602 | | -0.04764318 | | 757 | |
| lyso.PC.a.C20.5 | 0.02095566 | -0.04528959 | 0.04095878 | -0.08371575 | | -0.00686342 | | 698 | |
| lyso.PC.a.C22.5 | 0.0118487 | -0.05295627 | 0.02497519 | -0.09415966 | | -0.01175288 | | 666 | |
| lyso.PC.a.C22.6 | 0.00307643 | -0.05646359 | 0.00760268 | -0.09378551 | | -0.01914166 | | 717 | |
| lyso.PC.e.C16.0 | 0.99804864 | 4.5858E-05 | 0.99804864 | -0.03675224 | | 0.03684395 | | 756 | |
| lyso.PC.e.C18.0 | 0.03492516 | -0.04109784 | 0.06199939 | -0.07927936 | | -0.00291632 | | 740 | |
| lyso.PC.e.C18.1 | 0.5907928 | -0.01287454 | 0.64806353 | -0.05988267 | | 0.0341336 | | 548 | |
| PC.aa.C30.0 | 4.5448E-19 | 0.16318667 | 8.1428E-18 | 0.12825771 | | 0.19811563 | | 753 | |
| PC.aa.C30.1 | 9.5141E-09 | 0.13428961 | 4.649E-08 | 0.08906404 | | 0.17951518 | | 538 | |
| PC.aa.C30.2 | 0.03589061 | 0.05360488 | 0.06218577 | 0.00354523 | | 0.10366452 | | 501 | |
| PC.aa.C32.0 | 7.8617E-14 | 0.14091379 | 6.501E-13 | 0.10459968 | | 0.17722789 | | 757 | |
| PC.aa.C32.1 | 1.4615E-28 | 0.21285381 | 6.2846E-27 | 0.17693278 | | 0.24877484 | | 665 | |
| PC.aa.C32.2 | 1.5182E-23 | 0.2010066 | 4.0802E-22 | 0.163098 | | 0.23891521 | | 648 | |
| PC.aa.C32.3 | 1.087E-14 | 0.15375792 | 9.7373E-14 | 0.11559924 | | 0.1919166 | | 666 | |
| PC.aa.C34.0 | 2.0805E-10 | 0.13999076 | 1.278E-09 | 0.0975571 | | 0.18242441 | | 547 | |
| PC.aa.C34.1 | 1.3686E-31 | 0.21883097 | 1.9073E-29 | 0.18387799 | | 0.25378394 | | 716 | |
| PC.aa.C34.2 | 7.7628E-23 | 0.18953991 | 1.8544E-21 | 0.15303949 | | 0.22604033 | | 706 | |
| PC.aa.C34.3 | 9.4669E-29 | 0.20937408 | 5.0885E-27 | 0.17407617 | | 0.24467199 | | 706 | |
| PC.aa.C34.4 | 3.508E-25 | 0.19995657 | 1.257E-23 | 0.16364919 | | 0.23626395 | | 665 | |
| PC.aa.C34.5 | 1.9755E-07 | 0.10275625 | 7.7223E-07 | 0.06437842 | | 0.14113408 | | 674 | |
| PC.aa.C36.0 | 0.15905307 | 0.02828893 | 0.22205462 | -0.01110897 | | 0.06768683 | | 705 | |
| PC.aa.C36.1 | 2.6177E-14 | 0.15212989 | 2.2512E-13 | 0.11374501 | | 0.19051477 | | 705 | |
| PC.aa.C36.2 | 1.1621E-11 | 0.13213228 | 8.3281E-11 | 0.09454397 | | 0.1697206 | | 706 | |
| PC.aa.C36.3 | 1.7743E-31 | 0.2176724 | 1.9073E-29 | 0.18283216 | | 0.25251264 | | 716 | |
| PC.aa.C36.4 | 4.3207E-24 | 0.19836265 | 1.3271E-22 | 0.16140838 | | 0.23531692 | | 665 | |
| PC.aa.C36.5 | 4.7475E-10 | 0.12836248 | 2.6861E-09 | 0.08853868 | | 0.16818628 | | 625 | |
| PC.aa.C36.6 | 1.7795E-18 | 0.16420806 | 2.943E-17 | 0.12842023 | | 0.19999588 | | 750 | |
| PC.aa.C38.0 | 0.43288344 | 0.01680507 | 0.51137329 | -0.02524466 | | 0.0588548 | | 665 | |
| PC.aa.C38.1 | 0.01569873 | 0.05349859 | 0.03214503 | 0.01013206 | | 0.09686512 | | 605 | |
| PC.aa.C38.2 | 8.6966E-17 | 0.16354849 | 1.0388E-15 | 0.12592347 | | 0.20117351 | | 715 | |
| PC.aa.C38.3 | 4.65E-31 | 0.23220399 | 3.33E-29 | 0.19494081 | | 0.26946718 | | 648 | |
| PC.aa.C38.4 | 6.29E-16 | 0.1634748 | 6.4398E-15 | 0.12479709 | | 0.20215251 | | 648 | |
| PC.aa.C38.5 | 9.9573E-16 | 0.15908674 | 9.731E-15 | 0.12117004 | | 0.19700345 | | 648 | |
| PC.aa.C38.6 | 3.0434E-18 | 0.1860141 | 4.6738E-17 | 0.14534041 | | 0.22668779 | | 648 | |
| PC.aa.C40.0 | 0.47851845 | 0.01614595 | 0.55312617 | -0.0285708 | | 0.0608627 | | 605 | |
| PC.aa.C40.1 | 0.1472221 | 0.03694371 | 0.20994773 | -0.01306445 | | 0.08695187 | | 439 | |
| PC.aa.C40.3 | 0.03582985 | 0.05239037 | 0.06218577 | 0.00348306 | | 0.10129769 | | 440 | |
| PC.aa.C40.4 | 4.857E-17 | 0.15588237 | 6.5267E-16 | 0.12034085 | | 0.1914239 | | 706 | |
| PC.aa.C40.5 | 7.89E-17 | 0.16839978 | 9.98E-16 | 0.12981252 | | 0.20698704 | | 648 | |
| PC.aa.C40.6 | 1.0631E-20 | 0.19130991 | 2.2857E-19 | 0.1523405 | | 0.23027932 | | 696 | |
| PC.aa.C42.0 | 0.33460273 | -0.02153334 | 0.41108336 | -0.06532774 | | 0.02226107 | | 606 | |
| PC.aa.C42.1 | 0.9275611 | 0.00237242 | 0.93627059 | -0.04887476 | | 0.05361961 | | 498 | |
| PC.aa.C42.2 | 0.15353038 | 0.03583751 | 0.21716469 | -0.01342684 | | 0.08510186 | | 477 | |
| PC.aa.C42.4 | 0.12091977 | 0.04299297 | 0.17685544 | -0.0113864 | | 0.09737234 | | 425 | |
| PC.aa.C42.5 | 2.9268E-08 | 0.1296639 | 1.3388E-07 | 0.08440733 | | 0.17492048 | | 555 | |
| PC.aa.C42.6 | 5.0125E-09 | 0.1372969 | 2.5062E-08 | 0.09193663 | | 0.18265718 | | 538 | |
| PC.aa.C43.4 | 0.20029175 | -0.03307723 | 0.26914203 | -0.08375722 | | 0.01760277 | | 479 | |
| PC.aa.C43.6 | 0.01985329 | -0.04674014 | 0.03952275 | -0.08604981 | | -0.00743047 | | 706 | |
| PC.aa.C44.12 | 0.07944496 | -0.03418424 | 0.1228825 | -0.072395 | | 0.00402651 | | 706 | |
| PC.ae.C30.0 | 0.03644375 | 0.04397306 | 0.06218577 | 0.00278301 | | 0.08516312 | | 639 | |
| PC.ae.C30.1 | 0.48474856 | 0.01600185 | 0.55733123 | -0.0289533 | | 0.06095699 | | 581 | |
| PC.ae.C32.0 | 0.00623963 | 0.05090655 | 0.01427149 | 0.01446934 | | 0.08734377 | | 757 | |
| PC.ae.C32.1 | 0.7663952 | 0.00562582 | 0.79987848 | -0.03153538 | | 0.04278701 | | 757 | |
| PC.ae.C32.2 | 0.19349459 | -0.0244315 | 0.2632996 | -0.06128394 | | 0.01242094 | | 756 | |
| PC.ae.C34.0 | 2.2746E-08 | 0.1083866 | 1.0631E-07 | 0.07075294 | | 0.14602026 | | 716 | |
| PC.ae.C34.1 | 1.0755E-06 | 0.09515133 | 3.9193E-06 | 0.05718597 | | 0.13311669 | | 716 | |
| PC.ae.C34.2 | 7.43E-01 | -0.00661393 | 7.79E-01 | -0.04617198 | | 0.03294411 | | 716 | |
| PC.ae.C34.3 | 3.52E-02 | -0.04300076 | 6.20E-02 | -0.08300614 | | -0.00299538 | | 716 | |
| PC.ae.C34.4 | 0.26571754 | 0.02308967 | 0.34209144 | -0.01761219 | | 0.06379153 | | 663 | |
| PC.ae.C36.0 | 0.92651552 | 0.00180865 | 0.93627059 | -0.03668005 | | 0.04029734 | | 705 | |
| PC.ae.C36.1 | 3.2595E-08 | 0.10917923 | 1.46E-07 | 0.07083526 | | 0.14752319 | | 706 | |
| PC.ae.C36.2 | 0.00035676 | 0.07189766 | 0.00100925 | 0.03255408 | | 0.11124124 | | 706 | |
| PC.ae.C36.3 | 5.54E-01 | 0.01137402 | 0.61447796 | -0.02638479 | | 0.04913283 | | 757 | |
| PC.ae.C36.4 | 0.23123695 | 0.02444918 | 0.30130875 | -0.01561494 | | 0.06451329 | | 665 | |
| PC.ae.C36.5 | 0.80674963 | 0.00504569 | 0.83389985 | -0.03544041 | | 0.04553179 | | 665 | |
| PC.ae.C36.6 | 0.72314523 | 0.00813165 | 0.76213835 | -0.03693355 | | 0.05319686 | | 564 | |
| PC.ae.C38.0 | 1.4934E-12 | 0.13874436 | 1.1467E-11 | 0.10094991 | | 0.17653881 | | 706 | |
| PC.ae.C38.2 | 2.1588E-05 | 0.085665 | 7.0326E-05 | 0.04634148 | | 0.12498851 | | 706 | |
| PC.ae.C38.3 | 1.1614E-11 | 0.13209172 | 8.3281E-11 | 0.09451544 | | 0.16966801 | | 706 | |
| PC.ae.C38.4 | 0.01556245 | 0.04936666 | 0.03214503 | 0.00939918 | | 0.08933413 | | 665 | |
| PC.ae.C38.5 | 0.64603801 | -0.00943463 | 0.6933273 | -0.04975348 | | 0.03088422 | | 665 | |
| PC.ae.C38.6 | 0.47393336 | 0.01431178 | 0.55078742 | -0.02490638 | | 0.05352995 | | 716 | |
| PC.ae.C40.0 | 1.6426E-16 | 0.16446929 | 1.8588E-15 | 0.12627927 | | 0.20265931 | | 706 | |
| PC.ae.C40.1 | 1.3838E-07 | 0.10253621 | 5.6135E-07 | 0.06471258 | | 0.14035984 | | 706 | |
| PC.ae.C40.2 | 1.0558E-07 | 0.11011502 | 4.3654E-07 | 0.06990381 | | 0.15032623 | | 665 | |
| PC.ae.C40.3 | 0.01125775 | 0.0520668 | 0.02396451 | 0.01184426 | | 0.09228935 | | 666 | |
| PC.ae.C40.4 | 0.57231856 | 0.01105571 | 0.6310179 | -0.0273695 | | 0.04948091 | | 706 | |
| PC.ae.C40.5 | 0.82971364 | -0.00427873 | 0.85353317 | -0.04332518 | | 0.03476772 | | 706 | |
| PC.ae.C40.6 | 0.04989998 | 0.04245931 | 0.08066538 | 1.8601E-05 | | 0.08490001 | | 648 | |
| PC.ae.C42.0 | 2.8244E-10 | 0.13334895 | 1.6868E-09 | 0.09248752 | | 0.17421039 | | 665 | |
| PC.ae.C42.1 | 7.6478E-08 | 0.11713474 | 3.2241E-07 | 0.07487908 | | 0.1593904 | | 601 | |
| PC.ae.C42.2 | 0.00024881 | 0.07414427 | 0.00072289 | 0.03462294 | | 0.11366561 | | 666 | |
| PC.ae.C42.3 | 4.06E-01 | 0.01665539 | 0.48809439 | -0.02270463 | | 0.05601541 | | 714 | |
| PC.ae.C42.4 | 0.11567903 | -0.03081087 | 0.17152409 | -0.06921611 | | 0.00759438 | | 706 | |
| PC.ae.C42.5 | 0.32959045 | -0.02017605 | 0.40896292 | -0.06078191 | | 0.02042981 | | 666 | |
| PC.ae.C42.6 | 0.31613877 | -0.01948642 | 0.39753568 | -0.0576232 | | 0.01865037 | | 756 | |
| SM.a.C30.1 | 8.3694E-06 | 0.09666471 | 2.8562E-05 | 0.05440748 | | 0.13892195 | | 655 | |
| SM.a.C32.0 | 0.03630745 | 0.05778142 | 0.06218577 | 0.00370186 | | 0.11186098 | | 443 | |
| SM.a.C32.1 | 6.2601E-08 | 0.107005 | 2.6918E-07 | 0.0686035 | | 0.14540649 | | 706 | |
| SM.a.C32.2 | 3.45E-08 | 0.11064935 | 1.51E-07 | 0.07171303 | | 0.14958567 | | 716 | |
| SM.a.C33.1 | 0.00664102 | 0.05308722 | 0.01502968 | 0.01480287 | | 0.09137158 | | 706 | |
| SM.a.C33.2 | 3.78E-04 | 0.07698709 | 1.06E-03 | 0.03469319 | | 0.11928099 | | 615 | |
| SM.a.C34.0 | 0.63400268 | 0.01218863 | 0.68843725 | -0.03808303 | | 0.06246029 | | 493 | |
| SM.a.C34.1 | 0.34278813 | 0.01878098 | 0.41874687 | -0.02006139 | | 0.05762336 | | 717 | |
| SM.a.C34.2 | 4.33E-04 | 0.06789319 | 1.18E-03 | 0.03018596 | | 0.10560042 | | 757 | |
| SM.a.C34.3 | 0.01744197 | 0.05859501 | 0.03537759 | 0.01033065 | | 0.10685937 | | 498 | |
| SM.a.C35.0 | 0.52390373 | -0.01442989 | 0.59597514 | -0.05887453 | | 0.03001475 | | 564 | |
| SM.a.C35.1 | 0.90907984 | -0.00223971 | 0.92631358 | -0.04073238 | | 0.03625296 | | 706 | |
| SM.a.C36.0 | 3.26E-10 | 0.15978868 | 1.90E-09 | 0.11087998 | | 0.20869737 | | 498 | |
| SM.a.C36.1 | 0.00019822 | 0.07443551 | 0.00058575 | 0.03537061 | | 0.11350042 | | 717 | |
| SM.a.C36.2 | 3.10E-02 | 0.0409551 | 5.70E-02 | 0.00375098 | | 0.07815922 | | 757 | |
| SM.a.C36.3 | 1.71E-01 | 0.02699296 | 0.23722384 | -0.01168137 | | 0.0656673 | | 716 | |
| SM.a.C37.1 | 0.00355304 | 0.05973619 | 0.00868072 | 0.01964773 | | 0.09982465 | | 666 | |
| SM.a.C38.1 | 1.2356E-19 | 0.1696041 | 2.4151E-18 | 0.13390353 | | 0.20530467 | | 755 | |
| SM.a.C38.2 | 1.7546E-13 | 0.14075988 | 1.3972E-12 | 0.10394199 | | 0.17757777 | | 757 | |
| SM.a.C39.1 | 7.7979E-06 | 0.09395343 | 2.7041E-05 | 0.05301882 | | 0.13488804 | | 666 | |
| SM.a.C39.2 | 0.02623788 | 0.04554231 | 0.05024278 | 0.00539863 | | 0.08568599 | | 715 | |
| SM.a.C40.1 | 7.4806E-06 | 0.09046734 | 2.6366E-05 | 0.05113279 | | 0.12980188 | | 661 | |
| SM.a.C40.2 | 1.0121E-08 | 0.1215436 | 4.8358E-08 | 0.08045256 | | 0.16263465 | | 625 | |
| SM.a.C40.3 | 1.1847E-17 | 0.19411156 | 1.698E-16 | 0.15110579 | | 0.23711732 | | 536 | |
| SM.a.C40.4 | 2.9571E-16 | 0.15229936 | 3.1789E-15 | 0.11655791 | | 0.18804081 | | 757 | |
| SM.a.C40.5 | 2.3371E-09 | 0.11921737 | 1.2562E-08 | 0.08056487 | | 0.15786986 | | 675 | |
| SM.a.C41.1 | 1.5845E-10 | 0.12521243 | 1.002E-09 | 0.08736241 | | 0.16306245 | | 706 | |
| SM.a.C41.2 | 0.00150929 | 0.0633451 | 0.00386307 | 0.02430356 | | 0.10238664 | | 706 | |
| SM.a.C42.1 | 1.4473E-10 | 0.12242087 | 9.4293E-10 | 0.08549689 | | 0.15934484 | | 706 | |
| SM.a.C42.2 | 5.5668E-07 | 0.0984957 | 2.0636E-06 | 0.06022548 | | 0.13676592 | | 706 | |
| SM.a.C42.3 | 0.00049253 | 0.07009461 | 0.00132366 | 0.03078937 | | 0.10939985 | | 706 | |
| SM.a.C42.4 | 3.4386E-11 | 0.13292392 | 2.3849E-10 | 0.09421217 | | 0.17163566 | | 665 | |
| SM.a.C42.5 | 1.0505E-10 | 0.16279957 | 7.0582E-10 | 0.11436582 | | 0.21123333 | | 498 | |
| SM.a.C42.6 | 9.653E-15 | 0.16079378 | 9.0235E-14 | 0.12097175 | | 0.20061582 | | 665 | |
| SM.a.C43.1 | 0.00183798 | 0.06191705 | 0.004649 | 0.02304429 | | 0.10078982 | | 706 | |
| SM.a.C43.2 | 0.00251025 | 0.0596911 | 0.00627564 | 0.02105294 | | 0.09832926 | | 706 | |
| SM.a.C44.2 | 0.03379673 | 0.04858449 | 0.06106132 | 0.00373398 | | 0.09343501 | | 556 | |
| SM.a.C44.6 | 1.1573E-09 | 0.13622487 | 6.3799E-09 | 0.09295032 | | 0.17949941 | | 624 | |
| SM.a.C31.1 | 0.00019888 | 0.0778214 | 0.00058575 | 0.03698184 | | 0.11866095 | | 665 | |
| SM.a.C33.3 | 0.23084259 | 0.0238913 | 0.30130875 | -0.01522293 | | 0.06300553 | | 715 | |
| SM.a.C35.2 | 0.2345307 | 0.0233634 | 0.30375964 | -0.01518997 | | 0.06191678 | | 717 | |
| SM.a.C37.3 | 4.47E-01 | -0.01512271 | 0.52568529 | -0.0541843 | | 0.02393887 | | 717 | |
| SM.a.C39.5 | 0.55440399 | 0.01323446 | 0.61447796 | -0.03070838 | | 0.0571773 | | 598 | |
| SM.a.C41.0 | 0.00014718 | 0.08214257 | 0.00044568 | 0.0399178 | | 0.12436733 | | 605 | |
| SM.a.C41.3 | 0.15833544 | 0.03287291 | 0.22205462 | -0.01283896 | | 0.07858477 | | 550 | |
| SM.a.C43.0 | 0.00371834 | 0.07115112 | 0.00898251 | 0.02318688 | | 0.11911536 | | 539 | |
| SM.a.C43.3 | 0.116966 | 0.03489534 | 0.17224446 | -0.00875823 | | 0.0785489 | | 616 | |
| Ala | 0.00070607 | 0.06422851 | 0.00187415 | 0.02715945 | | 0.10129757 | | 754 | |
| Arg | 0.00495665 | -0.05494955 | 0.01158347 | -0.09322617 | | -0.01667294 | | 752 | |
| Asn | 0.03374767 | -0.04150764 | 0.06106132 | -0.07981709 | | -0.00319819 | | 703 | |
| Asp | 0.01828253 | 0.0443453 | 0.03673593 | 0.00753595 | | 0.08115466 | | 754 | |
| Cit | 0.00082994 | -0.06487252 | 0.00217607 | -0.10281668 | | -0.02692835 | | 754 | |
| Gln | 4.7648E-07 | -0.09923642 | 1.8293E-06 | -0.13756015 | | -0.06091268 | | 703 | |
| Glu | 4.95E-07 | 0.09877358 | 1.87E-06 | 0.06055015 | | 0.13699702 | | 754 | |
| Gly | 1.4492E-07 | -0.09992512 | 5.7699E-07 | -0.13686702 | | -0.06298321 | | 754 | |
| His | 0.04093673 | 0.03843819 | 0.06876091 | 0.00158767 | | 0.07528871 | | 743 | |
| Ile | 3.31E-01 | 0.02116828 | 0.40896292 | -0.0215678 | | 0.06390437 | | 543 | |
| Leu | 3.91E-01 | 0.01816265 | 4.72E-01 | -0.0233577 | | 0.059683 | | 703 | |
| Lys | 0.88291895 | 0.00295862 | 0.90394083 | -0.03647133 | | 0.04238858 | | 703 | |
| Met | 0.10438147 | -0.03450818 | 0.15804237 | -0.07617413 | | 0.00715777 | | 703 | |
| Orn | 0.22320615 | -0.02538911 | 0.29441301 | -0.06627785 | | 0.01549962 | | 703 | |
| Phe | 6.11E-01 | -0.01046467 | 0.66725869 | -0.05088543 | | 0.02995608 | | 703 | |
| Pro | 0.3190935 | 0.01967611 | 0.39886687 | -0.01906802 | | 0.05842025 | | 754 | |
| Trp | 0.36633586 | 0.01729996 | 0.44498424 | -0.02027319 | | 0.0548731 | | 754 | |
| Ser | 1.5538E-06 | -0.08948815 | 5.568E-06 | -0.12575929 | | -0.05321701 | | 754 | |
| Thr | 0.03076227 | 0.04229766 | 0.0569789 | 0.00393086 | | 0.08066445 | | 714 | |
| Tyr | 1.14E-01 | 0.03214219 | 1.70E-01 | -0.00775235 | | 0.07203672 | | 703 | |
| Val | 5.52E-02 | 0.03959145 | 8.86E-02 | -0.00088321 | | 0.08006611 | | 703 | |
| NEFA_12_0 | 0.04024638 | 0.04585372 | 0.06813363 | 0.0020466 | | 0.08966084 | | 602 | |
| NEFA_14_0 | 0.04936508 | 0.03908993 | 0.08040525 | 0.0001089 | | 0.07807096 | | 731 | |
| NEFA_14_1 | 0.19526945 | 0.02507236 | 0.2640436 | -0.01289792 | | 0.06304263 | | 750 | |
| NEFA_15_0 | 0.00406057 | 0.05588686 | 0.00959366 | 0.01782379 | | 0.09394994 | | 747 | |
| NEFA_15_1 | 0.01014269 | 0.06100148 | 0.02180677 | 0.01456198 | | 0.10744098 | | 506 | |
| NEFA_16_0 | 0.00764537 | 0.05548301 | 0.01694591 | 0.01476737 | | 0.09619865 | | 641 | |
| NEFA_16_1 | 0.52272121 | 0.01220126 | 0.59597514 | -0.02525743 | | 0.04965995 | | 750 | |
| NEFA_17_0 | 0.00013904 | 0.07750914 | 0.00042704 | 0.03779218 | | 0.1172261 | | 692 | |
| NEFA_17_1 | 0.0348287 | 0.04327532 | 0.06199939 | 0.00309301 | | 0.08345764 | | 679 | |
| NEFA_18_0 | 0.00038635 | 0.07719958 | 0.00106494 | 0.03471852 | | 0.11968063 | | 636 | |
| NEFA_18_1 | 0.29014716 | 0.02126326 | 0.36912213 | -0.01817431 | | 0.06070084 | | 694 | |
| NEFA_18_2 | 0.46692239 | 0.0153262 | 0.54558866 | -0.02601896 | | 0.05667137 | | 643 | |
| NEFA_18_3 | 0.04557544 | 0.03800364 | 0.07537477 | 0.00074977 | | 0.07525752 | | 750 | |
| NEFA_20_1 | 0.07218355 | 0.03616729 | 0.11328076 | -0.00326809 | | 0.07560266 | | 665 | |
| NEFA_20_2 | 0.00932671 | 0.05359368 | 0.02046166 | 0.01323824 | | 0.09394912 | | 633 | |
| NEFA_20_3 | 0.00025288 | 0.07586556 | 0.00072491 | 0.03538579 | | 0.11634533 | | 643 | |
| NEFA_20_4 | 0.06137566 | 0.03919704 | 0.09774643 | -0.0018742 | | 0.08026829 | | 643 | |
| NEFA_22_6 | 0.09715776 | 0.03447705 | 0.14920657 | -0.00627604 | | 0.07523014 | | 694 | |
| NEFA_24_1 | 0.22107169 | 0.02345992 | 0.29441301 | -0.01414565 | | 0.06106549 | | 749 | |
| NEFA_12_1 | 0.54018866 | -0.01241064 | 0.6059387 | -0.05217331 | | 0.02735203 | | 688 | |
| NEFA_13_1 | 0.64818041 | 0.01257385 | 0.6933273 | -0.04156563 | | 0.06671332 | | 393 | |
| NEFA_14_2 | 0.69263643 | 0.00875535 | 0.73358046 | -0.03472532 | | 0.05223602 | | 603 | |
| NEFA_16_2 | 0.02431892 | 0.0471045 | 0.04710422 | 0.00612896 | | 0.08808004 | | 659 | |
| NEFA_18_4 | 0.04512646 | 0.04504846 | 0.07521077 | 0.00098203 | | 0.08911489 | | 527 | |
| NEFA_19_0 | 0.02048311 | 0.05140807 | 0.04040246 | 0.00795836 | | 0.09485779 | | 577 | |
| NEFA_19_1 | 0.10898027 | 0.03391434 | 0.16385146 | -0.00757824 | | 0.07540693 | | 653 | |
| NEFA_20_5 | 1.40E-01 | 0.03071972 | 2.02E-01 | -0.01010305 | | 0.07154249 | | 653 | |
| NEFA_22_4 | 0.22184598 | 0.02613003 | 0.29441301 | -0.01583626 | | 0.06809633 | | 577 | |
| NEFA_22_5 | 0.54111735 | 0.0127196 | 0.6059387 | -0.02812945 | | 0.05356864 | | 653 | |
| NEFA_24_4 | 0.02819443 | 0.05065237 | 0.05317371 | 0.0054436 | | 0.09586113 | | 399 | |
| NEFA_24_5 | 2.0929E-05 | 0.10333826 | 6.9226E-05 | 0.05611861 | | 0.15055792 | | 456 | |
| NEFA_26_1 | 0.06897046 | 0.04658618 | 0.10903419 | -0.00363342 | | 0.09680578 | | 549 | |

### Glucose

| Analytes | P-Value | BETA | FDR P-Value | CI lower | CI upper | Number of observations |
| --- | --- | --- | --- | --- | --- | --- |
| Carn | 0.00112874 | 0.05100486 | 0.01516739 | 0.02037502 | 0.0816347 | 757 |
| Carn.a.C10.0 | 0.3858932 | -0.01567185 | 0.55682576 | -0.05115517 | 0.01981147 | 480 |
| Carn.a.C10.1 | 0.73498835 | -0.00606857 | 0.83609786 | -0.04127923 | 0.02914208 | 467 |
| Carn.a.C12.0 | 0.81503317 | 0.00457793 | 0.88244232 | -0.03385573 | 0.04301159 | 474 |
| Carn.a.C12.1 | 0.83276111 | 0.00416125 | 0.88635465 | -0.03455603 | 0.04287853 | 408 |
| Carn.a.C14.0 | 0.16292848 | 0.02236235 | 0.36489191 | -0.00907425 | 0.05379896 | 630 |
| Carn.a.C14.1 | 0.54653921 | -0.00934089 | 0.68716918 | -0.03974359 | 0.02106181 | 689 |
| Carn.a.C14.2 | 0.39539379 | -0.01307855 | 0.56453819 | -0.04327519 | 0.01711809 | 683 |
| Carn.a.C16.0 | 0.19470705 | 0.02039978 | 0.39868586 | -0.01045516 | 0.05125473 | 754 |
| Carn.a.C16.1 | 0.10411694 | -0.02531405 | 0.26335463 | -0.05585574 | 0.00522764 | 687 |
| Carn.a.C18.0 | 0.00165549 | 0.05405224 | 0.01763704 | 0.02046328 | 0.08764119 | 606 |
| Carn.a.C18.1 | 0.82498096 | 0.0033792 | 0.88244232 | -0.02660856 | 0.03336696 | 756 |
| Carn.a.C2.0 | 0.61781494 | -0.00753045 | 0.74623715 | -0.0371499 | 0.02208899 | 706 |
| Carn.a.C3.0 | 1.41E-02 | 0.03895913 | 6.57E-02 | 0.00789095 | 0.07002731 | 756 |
| Carn.a.C4.0 | 0.05944911 | 0.02887041 | 0.17503636 | -0.00115379 | 0.05889462 | 656 |
| Carn.a.C4.0.DC | 0.44582067 | 0.01374624 | 0.61839642 | -0.02166379 | 0.04915626 | 417 |
| Carn.a.C5.0 | 0.17936231 | 0.02461953 | 0.3769847 | -0.01135495 | 0.06059401 | 503 |
| Carn.a.C6.0 | 0.59145411 | -0.00857958 | 0.71996623 | -0.03995396 | 0.0227948 | 640 |
| Carn.a.C8.1 | 0.85130377 | 0.00326593 | 0.88849665 | -0.03093941 | 0.03747127 | 576 |
| lyso.PC.a.C14.0 | 0.05620851 | 0.02863279 | 0.17503636 | -0.00075995 | 0.05802554 | 754 |
| lyso.PC.a.C16.0 | 0.35174653 | 0.01406649 | 0.52464828 | -0.01557191 | 0.04370488 | 706 |
| lyso.PC.a.C16.1 | 0.22798032 | 0.01872091 | 0.43913793 | -0.01173904 | 0.04918086 | 757 |
| lyso.PC.a.C18.0 | 0.0053098 | 0.04108228 | 0.03805355 | 0.01223566 | 0.0699289 | 757 |
| lyso.PC.a.C18.1 | 3.49E-03 | 0.04459592 | 2.75E-02 | 0.01472115 | 0.07447069 | 706 |
| lyso.PC.a.C18.2 | 8.76E-04 | 0.04986532 | 1.45E-02 | 0.02057167 | 0.07915897 | 706 |
| lyso.PC.a.C18.3 | 2.596E-05 | 0.07215581 | 0.00279069 | 0.03873122 | 0.1055804 | 605 |
| lyso.PC.a.C18.6 | 0.1484022 | 0.0261391 | 0.34268724 | -0.00933965 | 0.06161785 | 548 |
| lyso.PC.a.C20.2 | 0.7537001 | 0.00559961 | 0.84840587 | -0.02944652 | 0.04064574 | 499 |
| lyso.PC.a.C20.3 | 0.00584194 | 0.04533733 | 0.03806114 | 0.01314922 | 0.07752545 | 646 |
| lyso.PC.a.C20.4 | 0.00168539 | 0.04783775 | 0.01763704 | 0.01804511 | 0.0776304 | 757 |
| lyso.PC.a.C20.5 | 0.0034683 | 0.04335911 | 0.02750754 | 0.01433404 | 0.07238418 | 698 |
| lyso.PC.a.C22.5 | 0.05408169 | 0.03057543 | 0.17354573 | -0.00053781 | 0.06168867 | 666 |
| lyso.PC.a.C22.6 | 0.12307501 | 0.02344924 | 0.29731604 | -0.00637164 | 0.05327012 | 717 |
| lyso.PC.e.C16.0 | 0.02889532 | 0.03247419 | 0.11504619 | 0.00335255 | 0.06159584 | 756 |
| lyso.PC.e.C18.0 | 0.00030095 | 0.05587569 | 0.01078401 | 0.02567324 | 0.08607814 | 740 |
| lyso.PC.e.C18.1 | 0.00042738 | 0.06455648 | 0.01084978 | 0.02878143 | 0.10033153 | 548 |
| PC.aa.C30.0 | 0.24935078 | -0.01720398 | 0.44675349 | -0.04650088 | 0.01209292 | 753 |
| PC.aa.C30.1 | 0.80040875 | 0.00428402 | 0.88244232 | -0.02898761 | 0.03755566 | 538 |
| PC.aa.C30.2 | 0.24791492 | -0.02223593 | 0.44675349 | -0.06000347 | 0.01553162 | 501 |
| PC.aa.C32.0 | 0.0004069 | -0.0537982 | 0.01084978 | -0.0835346 | -0.02406179 | 757 |
| PC.aa.C32.1 | 0.01128017 | -0.03909766 | 0.05774373 | -0.06930962 | -0.00888571 | 665 |
| PC.aa.C32.2 | 0.04547639 | -0.03210388 | 0.16028563 | -0.06355965 | -0.00064812 | 648 |
| PC.aa.C32.3 | 0.8436305 | 0.0030776 | 0.88849665 | -0.02754719 | 0.03370238 | 666 |
| PC.aa.C34.0 | 0.52486735 | -0.01098619 | 0.66457774 | -0.04490452 | 0.02293214 | 547 |
| PC.aa.C34.1 | 0.00168286 | -0.04891794 | 0.01763704 | -0.07937538 | -0.01846049 | 716 |
| PC.aa.C34.2 | 0.0002909 | -0.05527745 | 0.01078401 | -0.08507869 | -0.02547622 | 706 |
| PC.aa.C34.3 | 0.15309126 | -0.02158004 | 0.3464697 | -0.051204 | 0.00804391 | 706 |
| PC.aa.C34.4 | 0.52072234 | -0.00992087 | 0.66457774 | -0.04023732 | 0.02039558 | 665 |
| PC.aa.C34.5 | 0.43945759 | 0.01226731 | 0.61352846 | -0.01887069 | 0.04340531 | 674 |
| PC.aa.C36.0 | 0.94883708 | -0.00099118 | 0.9532709 | -0.0313084 | 0.02932604 | 705 |
| PC.aa.C36.1 | 0.27636032 | 0.01707347 | 0.46785408 | -0.01369788 | 0.04784482 | 705 |
| PC.aa.C36.2 | 0.82449739 | 0.00337451 | 0.88244232 | -0.02649049 | 0.0332395 | 706 |
| PC.aa.C36.3 | 0.01636213 | -0.03730742 | 0.07179303 | -0.06774437 | -0.00687047 | 716 |
| PC.aa.C36.4 | 0.00848886 | -0.04112092 | 0.04932716 | -0.0717062 | -0.01053564 | 665 |
| PC.aa.C36.5 | 0.5790737 | 0.00890835 | 0.7155221 | -0.02261163 | 0.04042833 | 625 |
| PC.aa.C36.6 | 0.75292926 | -0.00479659 | 0.84840587 | -0.03470042 | 0.02510725 | 750 |
| PC.aa.C38.0 | 0.52548008 | -0.01042948 | 0.66457774 | -0.04266728 | 0.02180832 | 665 |
| PC.aa.C38.1 | 0.56426428 | -0.00960271 | 0.70533035 | -0.04229706 | 0.02309164 | 605 |
| PC.aa.C38.2 | 0.01022123 | -0.04100028 | 0.05615233 | -0.07225889 | -0.00974168 | 715 |
| PC.aa.C38.3 | 3.56E-01 | -0.01497217 | 5.25E-01 | -0.04682028 | 0.01687594 | 648 |
| PC.aa.C38.4 | 0.29345513 | -0.01675881 | 0.48826917 | -0.04805812 | 0.0145405 | 648 |
| PC.aa.C38.5 | 0.70329262 | -0.00595427 | 0.80859847 | -0.03663905 | 0.0247305 | 648 |
| PC.aa.C38.6 | 0.00101268 | -0.0553875 | 0.01451513 | -0.08832324 | -0.02245176 | 648 |
| PC.aa.C40.0 | 0.26046823 | -0.01928466 | 0.46219332 | -0.05291029 | 0.01434097 | 605 |
| PC.aa.C40.1 | 0.26871705 | -0.02071446 | 0.46219332 | -0.05747878 | 0.01604986 | 439 |
| PC.aa.C40.3 | 0.11567601 | -0.02965474 | 0.28469867 | -0.06662995 | 0.00732047 | 440 |
| PC.aa.C40.4 | 0.19771833 | -0.01885129 | 0.40103247 | -0.0475584 | 0.00985581 | 706 |
| PC.aa.C40.5 | 4.16E-01 | -0.01298047 | 5.85E-01 | -0.04431858 | 0.01835765 | 648 |
| PC.aa.C40.6 | 0.04878319 | -0.03314584 | 0.16516457 | -0.06611425 | -0.00017742 | 696 |
| PC.aa.C42.0 | 0.05847836 | -0.03242955 | 0.17503636 | -0.06602607 | 0.00116696 | 606 |
| PC.aa.C42.1 | 0.76020056 | -0.00566398 | 0.84952408 | -0.04210588 | 0.03077791 | 498 |
| PC.aa.C42.2 | 0.71220636 | -0.00601473 | 0.81449132 | -0.03803541 | 0.02600595 | 477 |
| PC.aa.C42.4 | 0.01070812 | -0.04846537 | 0.05615233 | -0.08562562 | -0.01130511 | 425 |
| PC.aa.C42.5 | 0.00019547 | -0.06251303 | 0.01050642 | -0.09525454 | -0.02977153 | 555 |
| PC.aa.C42.6 | 0.14982605 | -0.02443157 | 0.34268724 | -0.05770972 | 0.00884657 | 538 |
| PC.aa.C43.4 | 0.00551522 | -0.05049574 | 0.03806114 | -0.08608279 | -0.01490869 | 479 |
| PC.aa.C43.6 | 0.05142756 | -0.03006012 | 0.16752916 | -0.060307 | 0.00018677 | 706 |
| PC.aa.C44.12 | 0.06156094 | -0.02799842 | 0.17503636 | -0.05735621 | 0.00135937 | 706 |
| PC.ae.C30.0 | 0.30848938 | -0.01623748 | 0.50246376 | -0.04752251 | 0.01504756 | 639 |
| PC.ae.C30.1 | 0.67158211 | 0.00743292 | 0.78901723 | -0.02698359 | 0.04184944 | 581 |
| PC.ae.C32.0 | 0.35003407 | -0.01386511 | 0.52464828 | -0.04297332 | 0.0152431 | 757 |
| PC.ae.C32.1 | 0.21552061 | -0.01864339 | 0.42904566 | -0.04816943 | 0.01088264 | 757 |
| PC.ae.C32.2 | 0.06333045 | -0.02773221 | 0.17621649 | -0.05700811 | 0.00154368 | 756 |
| PC.ae.C34.0 | 0.89908931 | -0.00197923 | 0.9204962 | -0.03261136 | 0.0286529 | 716 |
| PC.ae.C34.1 | 0.16488752 | -0.0217365 | 0.36547234 | -0.05243293 | 0.00895994 | 716 |
| PC.ae.C34.2 | 2.29E-01 | -0.019302 | 4.39E-01 | -0.05076164 | 0.01215764 | 716 |
| PC.ae.C34.3 | 2.21E-01 | -0.01989326 | 4.35E-01 | -0.0518059 | 0.01201939 | 716 |
| PC.ae.C34.4 | 0.81314745 | 0.00370091 | 0.88244232 | -0.02703186 | 0.03443368 | 663 |
| PC.ae.C36.0 | 0.30286767 | -0.01523783 | 0.4970729 | -0.04425418 | 0.01377852 | 705 |
| PC.ae.C36.1 | 0.90537241 | 0.00182475 | 0.92253587 | -0.02830212 | 0.03195163 | 706 |
| PC.ae.C36.2 | 0.66128328 | -0.00681207 | 0.78550224 | -0.03732511 | 0.02370097 | 706 |
| PC.ae.C36.3 | 3.34E-01 | -0.01478658 | 0.52047514 | -0.04480484 | 0.01523168 | 757 |
| PC.ae.C36.4 | 0.23204424 | 0.01876979 | 0.44150011 | -0.012041 | 0.04958058 | 665 |
| PC.ae.C36.5 | 0.34659368 | 0.01492533 | 0.52464828 | -0.0161901 | 0.04604076 | 665 |
| PC.ae.C36.6 | 0.29523252 | 0.01847402 | 0.48826917 | -0.01616178 | 0.05310981 | 564 |
| PC.ae.C38.0 | 0.91750423 | -0.00158939 | 0.92905117 | -0.03170648 | 0.02852769 | 706 |
| PC.ae.C38.2 | 0.26643476 | -0.01732917 | 0.46219332 | -0.0479206 | 0.01326227 | 706 |
| PC.ae.C38.3 | 0.34913071 | -0.01423818 | 0.52464828 | -0.04407578 | 0.01559943 | 706 |
| PC.ae.C38.4 | 0.45508784 | 0.01174682 | 0.6272044 | -0.01911485 | 0.04260849 | 665 |
| PC.ae.C38.5 | 0.397312 | 0.01336925 | 0.56453819 | -0.01762528 | 0.04436377 | 665 |
| PC.ae.C38.6 | 0.92040884 | -0.00158995 | 0.92905117 | -0.0328203 | 0.02964041 | 716 |
| PC.ae.C40.0 | 0.01474432 | -0.03822306 | 0.06604225 | -0.06892051 | -0.00752562 | 706 |
| PC.ae.C40.1 | 0.87764157 | 0.00232647 | 0.91156008 | -0.02733125 | 0.03198418 | 706 |
| PC.ae.C40.2 | 0.00342793 | -0.04681003 | 0.02750754 | -0.0781031 | -0.01551696 | 665 |
| PC.ae.C40.3 | 0.34902918 | -0.01477936 | 0.52464828 | -0.04574659 | 0.01618787 | 666 |
| PC.ae.C40.4 | 0.50821868 | -0.00995566 | 0.66457774 | -0.03948461 | 0.0195733 | 706 |
| PC.ae.C40.5 | 0.62416075 | 0.00749098 | 0.74969029 | -0.02251376 | 0.03749572 | 706 |
| PC.ae.C40.6 | 0.66824704 | -0.00714864 | 0.78901723 | -0.03988963 | 0.02559235 | 648 |
| PC.ae.C42.0 | 0.00045418 | -0.05633921 | 0.01084978 | -0.08772864 | -0.02494979 | 665 |
| PC.ae.C42.1 | 0.00403371 | -0.0477145 | 0.02990513 | -0.08017564 | -0.01525337 | 601 |
| PC.ae.C42.2 | 0.81851226 | -0.00357846 | 0.88244232 | -0.03418902 | 0.0270321 | 666 |
| PC.ae.C42.3 | 6.30E-03 | -0.04362522 | 0.03982118 | -0.07488317 | -0.01236728 | 714 |
| PC.ae.C42.4 | 0.0639297 | -0.02787692 | 0.17621649 | -0.05737265 | 0.00161881 | 706 |
| PC.ae.C42.5 | 0.26777089 | -0.01757999 | 0.46219332 | -0.04870289 | 0.01354292 | 666 |
| PC.ae.C42.6 | 0.26277769 | -0.0172951 | 0.46219332 | -0.04759146 | 0.01300125 | 756 |
| SM.a.C30.1 | 0.50822149 | -0.0104464 | 0.66457774 | -0.0414341 | 0.0205413 | 655 |
| SM.a.C32.0 | 0.57678122 | 0.01068638 | 0.7155221 | -0.02692033 | 0.04829308 | 443 |
| SM.a.C32.1 | 0.39911537 | -0.01294735 | 0.56453819 | -0.04307665 | 0.01718195 | 706 |
| SM.a.C32.2 | 4.79E-01 | -0.01139446 | 6.50E-01 | -0.04297096 | 0.02018205 | 716 |
| SM.a.C33.1 | 0.33622555 | -0.01448853 | 0.52047514 | -0.04404917 | 0.0150721 | 706 |
| SM.a.C33.2 | 4.64E-01 | -0.01141796 | 6.36E-01 | -0.04204571 | 0.01920979 | 615 |
| SM.a.C34.0 | 0.01299778 | -0.04164853 | 0.06210049 | -0.07447302 | -0.00882405 | 493 |
| SM.a.C34.1 | 0.11080604 | -0.02509451 | 0.27701511 | -0.05595347 | 0.00576444 | 717 |
| SM.a.C34.2 | 4.92E-02 | -0.03027242 | 1.65E-01 | -0.06043388 | -0.00011096 | 757 |
| SM.a.C34.3 | 0.68494218 | 0.0071321 | 0.80034005 | -0.02738661 | 0.04165082 | 498 |
| SM.a.C35.0 | 0.33649323 | -0.01672299 | 0.52047514 | -0.05087078 | 0.01742479 | 564 |
| SM.a.C35.1 | 0.7646131 | -0.00451314 | 0.84952408 | -0.03409485 | 0.02506857 | 706 |
| SM.a.C36.0 | 6.73E-02 | -0.03369245 | 1.83E-01 | -0.06979709 | 0.00241219 | 498 |
| SM.a.C36.1 | 0.05052823 | -0.0312225 | 0.16713185 | -0.06251703 | 7.2034E-05 | 717 |
| SM.a.C36.2 | 3.98E-02 | -0.0310457 | 1.46E-01 | -0.06064314 | -0.00144826 | 757 |
| SM.a.C36.3 | 2.80E-01 | -0.01695429 | 0.47065843 | -0.04775608 | 0.01384749 | 716 |
| SM.a.C37.1 | 0.24305365 | -0.01838805 | 0.44663704 | -0.04928972 | 0.01251361 | 666 |
| SM.a.C38.1 | 0.00172269 | -0.04778857 | 0.01763704 | -0.07761177 | -0.01796538 | 755 |
| SM.a.C38.2 | 0.00093577 | -0.05102398 | 0.01446312 | -0.08117234 | -0.02087562 | 757 |
| SM.a.C39.1 | 0.35435138 | -0.01503146 | 0.52464828 | -0.04687712 | 0.01681421 | 666 |
| SM.a.C39.2 | 0.32612949 | -0.0160572 | 0.51557236 | -0.0481405 | 0.01602611 | 715 |
| SM.a.C40.1 | 0.07306692 | -0.02794555 | 0.19636734 | -0.05851083 | 0.00261974 | 661 |
| SM.a.C40.2 | 0.06015506 | -0.03095269 | 0.17503636 | -0.06323191 | 0.00132654 | 625 |
| SM.a.C40.3 | 0.14201455 | -0.02462959 | 0.33188183 | -0.05753233 | 0.00827315 | 536 |
| SM.a.C40.4 | 0.06187332 | -0.02825708 | 0.17503636 | -0.0579215 | 0.00140734 | 757 |
| SM.a.C40.5 | 0.35954917 | 0.01475825 | 0.52587124 | -0.01684813 | 0.04636463 | 675 |
| SM.a.C41.1 | 0.23586389 | -0.01808732 | 0.44400131 | -0.04801992 | 0.01184528 | 706 |
| SM.a.C41.2 | 0.01454483 | -0.03754782 | 0.06604225 | -0.067642 | -0.00745364 | 706 |
| SM.a.C42.1 | 0.11652783 | -0.02335668 | 0.28469867 | -0.05253834 | 0.00582498 | 706 |
| SM.a.C42.2 | 0.00246339 | -0.04605074 | 0.02302737 | -0.07580299 | -0.0162985 | 706 |
| SM.a.C42.3 | 0.00203504 | -0.04773686 | 0.01988787 | -0.07800211 | -0.01747161 | 706 |
| SM.a.C42.4 | 0.04735451 | -0.03106336 | 0.16421322 | -0.061763 | -0.00036372 | 665 |
| SM.a.C42.5 | 0.08893059 | -0.0311015 | 0.22761996 | -0.06695411 | 0.00475112 | 498 |
| SM.a.C42.6 | 0.00082238 | -0.05441859 | 0.01446312 | -0.08621302 | -0.02262417 | 665 |
| SM.a.C43.1 | 0.52482897 | 0.00974651 | 0.66457774 | -0.02033053 | 0.03982356 | 706 |
| SM.a.C43.2 | 0.97566484 | -0.00046458 | 0.97566484 | -0.03035647 | 0.02942731 | 706 |
| SM.a.C44.2 | 0.76654731 | -0.00486271 | 0.84952408 | -0.0370198 | 0.02729437 | 556 |
| SM.a.C44.6 | 0.02853886 | -0.03739679 | 0.11504619 | -0.07085544 | -0.00393813 | 624 |
| SM.a.C31.1 | 0.70258454 | -0.00611933 | 0.80859847 | -0.03757465 | 0.025336 | 665 |
| SM.a.C33.3 | 0.18021721 | -0.02118795 | 0.3769847 | -0.05219935 | 0.00982345 | 715 |
| SM.a.C35.2 | 0.83918455 | -0.00317391 | 0.88849665 | -0.03386905 | 0.02752123 | 717 |
| SM.a.C37.3 | 1.81E-01 | -0.02119053 | 0.3769847 | -0.05223307 | 0.00985202 | 717 |
| SM.a.C39.5 | 0.37676946 | 0.01417284 | 0.54733401 | -0.01729653 | 0.04564221 | 598 |
| SM.a.C41.0 | 0.59271638 | -0.00884136 | 0.71996623 | -0.04128618 | 0.02360346 | 605 |
| SM.a.C41.3 | 0.18462461 | -0.02216606 | 0.38167587 | -0.05494552 | 0.01061341 | 550 |
| SM.a.C43.0 | 0.01282356 | -0.04358746 | 0.06210049 | -0.07787706 | -0.00929786 | 539 |
| SM.a.C43.3 | 0.0408256 | -0.03262172 | 0.14629173 | -0.06387797 | -0.00136547 | 616 |
| Ala | 0.07774719 | 0.02670098 | 0.20384934 | -0.00297508 | 0.05637704 | 754 |
| Arg | 0.0016906 | 0.04879212 | 0.01763704 | 0.01839672 | 0.07918752 | 752 |
| Asn | 0.52410416 | 0.00984553 | 0.66457774 | -0.02048417 | 0.04017524 | 703 |
| Asp | 0.84792642 | -0.00287398 | 0.88849665 | -0.0322858 | 0.02653784 | 754 |
| Cit | 0.01212612 | 0.0388182 | 0.0606306 | 0.00851299 | 0.06912342 | 754 |
| Gln | 0.03685347 | 0.03271863 | 0.13900872 | 0.00200299 | 0.06343428 | 703 |
| Glu | 5.38E-04 | 0.05436862 | 1.16E-02 | 0.02366604 | 0.0850712 | 754 |
| Gly | 0.21521914 | 0.01891179 | 0.42904566 | -0.01101971 | 0.04884328 | 754 |
| His | 0.50854299 | -0.00992545 | 0.66457774 | -0.03938551 | 0.0195346 | 743 |
| Ile | 5.68E-02 | 0.03659249 | 0.17503636 | -0.00105962 | 0.0742446 | 543 |
| Leu | 1.73E-02 | 0.03968019 | 7.45E-02 | 0.00702324 | 0.07233714 | 703 |
| Lys | 0.27521162 | 0.01729578 | 0.46785408 | -0.01380178 | 0.04839334 | 703 |
| Met | 0.31965188 | 0.01670207 | 0.50907522 | -0.01622623 | 0.04963037 | 703 |
| Orn | 0.00568177 | 0.04540156 | 0.03806114 | 0.01327029 | 0.07753284 | 703 |
| Phe | 3.13E-02 | 0.03496018 | 0.12218347 | 0.00315547 | 0.06676489 | 703 |
| Pro | 0.0001398 | 0.05960106 | 0.01001919 | 0.02903936 | 0.09016276 | 754 |
| Trp | 0.23955419 | 0.01792611 | 0.44400131 | -0.01197245 | 0.04782466 | 754 |
| Ser | 0.03332565 | 0.03175415 | 0.12794669 | 0.00251614 | 0.06099217 | 754 |
| Thr | 0.89895195 | -0.00198274 | 0.9204962 | -0.03262741 | 0.02866192 | 714 |
| Tyr | 8.55E-06 | 0.07103014 | 1.84E-03 | 0.03993294 | 0.10212735 | 703 |
| Val | 2.65E-03 | 0.04889033 | 2.38E-02 | 0.01706575 | 0.08071491 | 703 |
| NEFA_12_0 | 0.48037704 | -0.01197181 | 0.64956644 | -0.04526898 | 0.02132536 | 602 |
| NEFA_14_0 | 0.06063717 | -0.02953543 | 0.17503636 | -0.06039407 | 0.00132321 | 731 |
| NEFA_14_1 | 0.01055953 | -0.03922189 | 0.05615233 | -0.06925888 | -0.0091849 | 750 |
| NEFA_15_0 | 0.52330141 | -0.00987893 | 0.66457774 | -0.04025046 | 0.02049259 | 747 |
| NEFA_15_1 | 0.17804868 | -0.02472054 | 0.3769847 | -0.06073313 | 0.01129205 | 506 |
| NEFA_16_0 | 0.17795559 | -0.02283863 | 0.3769847 | -0.0560952 | 0.01041793 | 641 |
| NEFA_16_1 | 0.00094178 | -0.0499315 | 0.01446312 | -0.0794498 | -0.02041319 | 750 |
| NEFA_17_0 | 0.69487864 | 0.0064503 | 0.80756166 | -0.02582453 | 0.03872513 | 692 |
| NEFA_17_1 | 0.17239644 | -0.02250487 | 0.3769847 | -0.05485386 | 0.00984412 | 679 |
| NEFA_18_0 | 0.88812743 | -0.00244323 | 0.91801633 | -0.03653625 | 0.0316498 | 636 |
| NEFA_18_1 | 0.04007118 | -0.03318354 | 0.14602209 | -0.06485872 | -0.00150836 | 694 |
| NEFA_18_2 | 0.00670552 | -0.04421779 | 0.04119106 | -0.07613968 | -0.01229591 | 643 |
| NEFA_18_3 | 0.12563211 | -0.02311908 | 0.30012116 | -0.0527201 | 0.00648195 | 750 |
| NEFA_20_1 | 0.08322791 | -0.02871247 | 0.21559037 | -0.06120975 | 0.0037848 | 665 |
| NEFA_20_2 | 0.07657873 | -0.03007674 | 0.20326454 | -0.06337418 | 0.0032207 | 633 |
| NEFA_20_3 | 0.06108417 | -0.03026067 | 0.17503636 | -0.06193249 | 0.00141115 | 643 |
| NEFA_20_4 | 0.31600565 | -0.01632499 | 0.50907522 | -0.04827125 | 0.01562128 | 643 |
| NEFA_22_6 | 0.12885271 | -0.02541341 | 0.30443224 | -0.05823058 | 0.00740376 | 694 |
| NEFA_24_1 | 0.81614427 | -0.00353959 | 0.88244232 | -0.03341576 | 0.02633658 | 749 |
| NEFA_12_1 | 0.00694101 | -0.04325247 | 0.04145327 | -0.07461373 | -0.01189122 | 688 |
| NEFA_13_1 | 0.02322737 | 0.05356561 | 0.09603623 | 0.0073493 | 0.09978191 | 393 |
| NEFA_14_2 | 0.31884009 | -0.01680812 | 0.50907522 | -0.04989567 | 0.01627943 | 603 |
| NEFA_16_2 | 0.00943614 | -0.04084698 | 0.05338868 | -0.07165354 | -0.01004043 | 659 |
| NEFA_18_4 | 0.50148389 | -0.01190055 | 0.66457774 | -0.04665908 | 0.02285799 | 527 |
| NEFA_19_0 | 0.59063203 | 0.00999879 | 0.71996623 | -0.02649035 | 0.04648794 | 577 |
| NEFA_19_1 | 0.48721172 | -0.0118633 | 0.65469074 | -0.04537499 | 0.02164838 | 653 |
| NEFA_20_5 | 6.61E-01 | 0.00737676 | 7.86E-01 | -0.02559134 | 0.04034487 | 653 |
| NEFA_22_4 | 0.22241637 | -0.02181827 | 0.43472291 | -0.05690293 | 0.0132664 | 577 |
| NEFA_22_5 | 0.23951314 | -0.01973201 | 0.44400131 | -0.05264404 | 0.01318002 | 653 |
| NEFA_24_4 | 0.24829672 | -0.02650359 | 0.44675349 | -0.07157169 | 0.01856451 | 399 |
| NEFA_24_5 | 0.00358238 | -0.05290223 | 0.02750754 | -0.08840598 | -0.01739848 | 456 |
| NEFA_26_1 | 0.01774046 | 0.04596038 | 0.07478821 | 0.00799914 | 0.08392163 | 549 |

### Systolic Blood Pressure

| Analytes | P-Value | BETA | FDR P-Value | CI lower | CI upper | Number of observations |
| --- | --- | --- | --- | --- | --- | --- |
| Carn | 5.7026E-06 | 2.21258792 | 0.00036583 | 1.26217353 | 3.16300232 | 757 |
| Carn.a.C10.0 | 0.34664531 | 0.55201832 | 0.53234815 | -0.599436 | 1.70347264 | 480 |
| Carn.a.C10.1 | 0.13102304 | 0.90271907 | 0.31651633 | -0.26995066 | 2.0753888 | 467 |
| Carn.a.C12.0 | 0.04560953 | 1.17742309 | 0.14008641 | 0.02308334 | 2.33176284 | 474 |
| Carn.a.C12.1 | 0.1419973 | 0.94982089 | 0.33342572 | -0.31932857 | 2.21897034 | 408 |
| Carn.a.C14.0 | 0.00208886 | 1.57865146 | 0.0154864 | 0.57549124 | 2.58181168 | 630 |
| Carn.a.C14.1 | 0.17578764 | 0.68036105 | 0.38579273 | -0.30533702 | 1.66605912 | 689 |
| Carn.a.C14.2 | 0.0445267 | 0.99855484 | 0.1387426 | 0.02449173 | 1.97261796 | 683 |
| Carn.a.C16.0 | 0.00128697 | 1.57724458 | 0.01064227 | 0.61898058 | 2.53550859 | 754 |
| Carn.a.C16.1 | 0.35053944 | 0.47216848 | 0.53373802 | -0.52023951 | 1.46457648 | 687 |
| Carn.a.C18.0 | 0.25442995 | 0.62964056 | 0.44473528 | -0.4543719 | 1.71365301 | 606 |
| Carn.a.C18.1 | 0.01111888 | 1.20923282 | 0.04980329 | 0.27657206 | 2.14189358 | 756 |
| Carn.a.C2.0 | 0.80030261 | 0.12402796 | 0.87788296 | -0.83828621 | 1.08634212 | 706 |
| Carn.a.C3.0 | 8.04E-06 | 2.1969757 | 3.66E-04 | 1.23763386 | 3.15631754 | 756 |
| Carn.a.C4.0 | 0.00580509 | 1.43831928 | 0.03284458 | 0.41790112 | 2.45873745 | 656 |
| Carn.a.C4.0.DC | 0.00133943 | 2.02249066 | 0.01066587 | 0.79153288 | 3.25344844 | 417 |
| Carn.a.C5.0 | 0.00084477 | 2.01001171 | 0.00789676 | 0.83409245 | 3.18593097 | 503 |
| Carn.a.C6.0 | 0.00733899 | 1.37410474 | 0.03735667 | 0.37092181 | 2.37728768 | 640 |
| Carn.a.C8.1 | 0.20916346 | -0.65056685 | 0.3971382 | -1.6668946 | 0.3657609 | 576 |
| lyso.PC.a.C14.0 | 0.02993016 | 1.01683404 | 0.09899975 | 0.09911965 | 1.93454843 | 754 |
| lyso.PC.a.C16.0 | 0.0024289 | 1.48338306 | 0.01740711 | 0.52636109 | 2.44040503 | 706 |
| lyso.PC.a.C16.1 | 3.3922E-05 | 2.00027743 | 0.00072932 | 1.05878643 | 2.94176843 | 757 |
| lyso.PC.a.C18.0 | 0.00461278 | 1.30407853 | 0.02858585 | 0.40311322 | 2.20504384 | 757 |
| lyso.PC.a.C18.1 | 7.41E-03 | 1.32887236 | 3.74E-02 | 0.35743806 | 2.30030666 | 706 |
| lyso.PC.a.C18.2 | 1.96E-02 | 1.13884765 | 7.33E-02 | 0.18336954 | 2.09432577 | 706 |
| lyso.PC.a.C18.3 | 0.00019736 | 2.02970064 | 0.00265198 | 0.96552302 | 3.09387826 | 605 |
| lyso.PC.a.C18.6 | 0.92881529 | -0.05104452 | 0.96471153 | -1.17294344 | 1.0708544 | 548 |
| lyso.PC.a.C20.2 | 0.21370852 | 0.7409079 | 0.3971382 | -0.4283325 | 1.91014831 | 499 |
| lyso.PC.a.C20.3 | 3.5104E-10 | 3.27427324 | 7.5473E-08 | 2.26583237 | 4.28271411 | 646 |
| lyso.PC.a.C20.4 | 8.5077E-06 | 2.11122134 | 0.00036583 | 1.18676816 | 3.03567452 | 757 |
| lyso.PC.a.C20.5 | 2.3528E-05 | 2.02796936 | 0.00063231 | 1.09279355 | 2.96314517 | 698 |
| lyso.PC.a.C22.5 | 0.00010985 | 1.98136967 | 0.00196811 | 0.98159541 | 2.98114393 | 666 |
| lyso.PC.a.C22.6 | 0.00571547 | 1.30493678 | 0.03284458 | 0.3807423 | 2.22913126 | 717 |
| lyso.PC.e.C16.0 | 0.01532139 | 1.12727608 | 0.06334807 | 0.21668538 | 2.03786679 | 756 |
| lyso.PC.e.C18.0 | 0.02241174 | 1.10414147 | 0.07899218 | 0.1568117 | 2.05147124 | 740 |
| lyso.PC.e.C18.1 | 0.0074601 | 1.55131867 | 0.03735667 | 0.41668495 | 2.68595238 | 548 |
| PC.aa.C30.0 | 0.69271025 | 0.18436602 | 0.81633185 | -0.73117183 | 1.09990386 | 753 |
| PC.aa.C30.1 | 0.35311556 | -0.53421973 | 0.53373802 | -1.66342702 | 0.59498757 | 538 |
| PC.aa.C30.2 | 0.51397201 | -0.39107864 | 0.67380477 | -1.56755363 | 0.78539635 | 501 |
| PC.aa.C32.0 | 0.82147428 | 0.10770802 | 0.88752246 | -0.82902714 | 1.04444319 | 757 |
| PC.aa.C32.1 | 0.18333616 | 0.66141873 | 0.38579273 | -0.31365691 | 1.63649437 | 665 |
| PC.aa.C32.2 | 0.50170441 | -0.34704133 | 0.66997794 | -1.36086831 | 0.66678566 | 648 |
| PC.aa.C32.3 | 0.81924615 | 0.11561533 | 0.88752246 | -0.87745757 | 1.10868822 | 666 |
| PC.aa.C34.0 | 0.31709442 | 0.5453412 | 0.50500222 | -0.52445734 | 1.61513974 | 547 |
| PC.aa.C34.1 | 0.17621381 | 0.65306669 | 0.38579273 | -0.29399514 | 1.60012852 | 716 |
| PC.aa.C34.2 | 0.21077409 | -0.62280379 | 0.3971382 | -1.59902188 | 0.3534143 | 706 |
| PC.aa.C34.3 | 0.62085178 | 0.24286689 | 0.754142 | -0.72071589 | 1.20644966 | 706 |
| PC.aa.C34.4 | 0.35499784 | 0.45941351 | 0.53373802 | -0.51521152 | 1.43403855 | 665 |
| PC.aa.C34.5 | 0.00261238 | 1.4673154 | 0.01811811 | 0.51376647 | 2.42086432 | 674 |
| PC.aa.C36.0 | 0.53253945 | 0.3131698 | 0.68973483 | -0.67150534 | 1.29784494 | 705 |
| PC.aa.C36.1 | 0.0395263 | 1.04782032 | 0.12497287 | 0.05038045 | 2.04526019 | 705 |
| PC.aa.C36.2 | 0.58688981 | 0.26855949 | 0.73361227 | -0.70143006 | 1.23854903 | 706 |
| PC.aa.C36.3 | 0.71709362 | 0.17443842 | 0.82332948 | -0.77036489 | 1.11924172 | 716 |
| PC.aa.C36.4 | 0.47012708 | 0.36379476 | 0.64793155 | -0.62466252 | 1.35225204 | 665 |
| PC.aa.C36.5 | 0.00423413 | 1.46862358 | 0.02796198 | 0.4640222 | 2.47322496 | 625 |
| PC.aa.C36.6 | 0.08757141 | 0.81578902 | 0.23244262 | -0.12044716 | 1.7520252 | 750 |
| PC.aa.C38.0 | 0.38627405 | -0.46147941 | 0.56495864 | -1.50668333 | 0.58372451 | 665 |
| PC.aa.C38.1 | 0.79702926 | 0.13898946 | 0.87788296 | -0.9218824 | 1.19986131 | 605 |
| PC.aa.C38.2 | 0.86670325 | -0.08364732 | 0.92248118 | -1.06173205 | 0.89443741 | 715 |
| PC.aa.C38.3 | 3.60E-04 | 1.85235751 | 4.52E-03 | 0.83830764 | 2.86640737 | 648 |
| PC.aa.C38.4 | 0.01258157 | 1.2768113 | 0.05410076 | 0.2749086 | 2.27871399 | 648 |
| PC.aa.C38.5 | 0.00107686 | 1.63599687 | 0.00964685 | 0.65796011 | 2.61403363 | 648 |
| PC.aa.C38.6 | 0.75391166 | -0.17049512 | 0.85053326 | -1.23803483 | 0.89704459 | 648 |
| PC.aa.C40.0 | 0.97388429 | 0.01803497 | 0.98302874 | -1.06347182 | 1.09954175 | 605 |
| PC.aa.C40.1 | 0.94189036 | 0.04513659 | 0.97358859 | -1.17122764 | 1.26150083 | 439 |
| PC.aa.C40.3 | 0.53653902 | 0.396874 | 0.69075383 | -0.86424123 | 1.65798923 | 440 |
| PC.aa.C40.4 | 0.00037827 | 1.68335057 | 0.0045182 | 0.75817511 | 2.60852602 | 706 |
| PC.aa.C40.5 | 3.92E-05 | 2.09711852 | 7.66E-04 | 1.10274436 | 3.09149269 | 648 |
| PC.aa.C40.6 | 0.05328467 | 1.00507942 | 0.161355 | -0.01426743 | 2.02442626 | 696 |
| PC.aa.C42.0 | 0.33747619 | -0.52730297 | 0.52577811 | -1.60613051 | 0.55152457 | 606 |
| PC.aa.C42.1 | 0.35953075 | -0.57099691 | 0.53679938 | -1.79430668 | 0.65231286 | 498 |
| PC.aa.C42.2 | 0.69483129 | 0.23630039 | 0.81633185 | -0.94660656 | 1.41920734 | 477 |
| PC.aa.C42.4 | 0.18426729 | -0.87388232 | 0.38579273 | -2.16551847 | 0.41775383 | 425 |
| PC.aa.C42.5 | 0.07284473 | -1.02843022 | 0.20607392 | -2.15244891 | 0.09558846 | 555 |
| PC.aa.C42.6 | 0.91177049 | -0.06391066 | 0.9562471 | -1.19644828 | 1.06862696 | 538 |
| PC.aa.C43.4 | 0.13342025 | 0.93491432 | 0.31872615 | -0.28711766 | 2.15694631 | 479 |
| PC.aa.C43.6 | 0.42894683 | -0.39698868 | 0.60536159 | -1.38181017 | 0.58783282 | 706 |
| PC.aa.C44.12 | 0.9639442 | 0.02202084 | 0.98153972 | -0.93407634 | 0.97811802 | 706 |
| PC.ae.C30.0 | 0.65175909 | 0.23134944 | 0.78283913 | -0.7748032 | 1.23750208 | 639 |
| PC.ae.C30.1 | 0.99045356 | 0.00672163 | 0.99045356 | -1.09620688 | 1.10965014 | 581 |
| PC.ae.C32.0 | 0.76640873 | 0.13772181 | 0.85377139 | -0.77205049 | 1.0474941 | 757 |
| PC.ae.C32.1 | 0.76360209 | 0.14149121 | 0.85377139 | -0.78174921 | 1.06473163 | 757 |
| PC.ae.C32.2 | 0.50508464 | -0.31127587 | 0.67032839 | -1.22767348 | 0.60512175 | 756 |
| PC.ae.C34.0 | 0.25972357 | 0.54360154 | 0.44952625 | -0.40260461 | 1.48980769 | 716 |
| PC.ae.C34.1 | 0.75559001 | -0.15072006 | 0.85053326 | -1.10100054 | 0.79956041 | 716 |
| PC.ae.C34.2 | 2.82E-02 | -1.08648265 | 9.49E-02 | -2.05678946 | -0.11617585 | 716 |
| PC.ae.C34.3 | 5.13E-01 | -0.32890034 | 6.74E-01 | -1.31629372 | 0.65849303 | 716 |
| PC.ae.C34.4 | 0.22728868 | 0.61097635 | 0.41412768 | -0.38175072 | 1.60370343 | 663 |
| PC.ae.C36.0 | 0.95130315 | 0.02927984 | 0.97861329 | -0.91171871 | 0.97027839 | 705 |
| PC.ae.C36.1 | 0.15834337 | 0.70291124 | 0.36216834 | -0.27436089 | 1.68018338 | 706 |
| PC.ae.C36.2 | 0.29075437 | -0.53340526 | 0.48086299 | -1.52395679 | 0.45714627 | 706 |
| PC.ae.C36.3 | 2.46E-01 | -0.55472559 | 0.4347573 | -1.49218443 | 0.38273325 | 757 |
| PC.ae.C36.4 | 0.09721806 | 0.83724355 | 0.254901 | -0.15260451 | 1.82709161 | 665 |
| PC.ae.C36.5 | 0.00746407 | 1.36108029 | 0.03735667 | 0.36524093 | 2.35691965 | 665 |
| PC.ae.C36.6 | 0.00197849 | 1.71945926 | 0.01519196 | 0.63287346 | 2.80604506 | 564 |
| PC.ae.C38.0 | 0.07698002 | 0.88055586 | 0.21152857 | -0.09559297 | 1.8567047 | 706 |
| PC.ae.C38.2 | 0.41856909 | -0.40983923 | 0.59994902 | -1.40401703 | 0.58433857 | 706 |
| PC.ae.C38.3 | 0.21426991 | 0.61336289 | 0.3971382 | -0.35544301 | 1.5821688 | 706 |
| PC.ae.C38.4 | 0.4069766 | 0.41935462 | 0.58724811 | -0.5730375 | 1.41174674 | 665 |
| PC.ae.C38.5 | 0.19362722 | 0.66005102 | 0.38906405 | -0.33596695 | 1.656069 | 665 |
| PC.ae.C38.6 | 0.20479072 | 0.6234639 | 0.3971382 | -0.34098472 | 1.58791253 | 716 |
| PC.ae.C40.0 | 0.61694631 | -0.25520525 | 0.75365601 | -1.25652538 | 0.74611488 | 706 |
| PC.ae.C40.1 | 0.26135247 | 0.55110534 | 0.44952625 | -0.4114613 | 1.51367199 | 706 |
| PC.ae.C40.2 | 0.30133606 | -0.53763353 | 0.4871222 | -1.55823593 | 0.48296887 | 665 |
| PC.ae.C40.3 | 0.98181056 | 0.01167173 | 0.98639846 | -0.99319155 | 1.01653502 | 666 |
| PC.ae.C40.4 | 0.79124442 | 0.12940489 | 0.87689459 | -0.83009601 | 1.0889058 | 706 |
| PC.ae.C40.5 | 0.66475412 | 0.21523546 | 0.79401187 | -0.75950314 | 1.18997406 | 706 |
| PC.ae.C40.6 | 0.54108859 | -0.32763575 | 0.69246457 | -1.37977597 | 0.72450446 | 648 |
| PC.ae.C42.0 | 0.07012034 | -0.94787746 | 0.20101163 | -1.97386871 | 0.0781138 | 665 |
| PC.ae.C42.1 | 0.33422044 | 0.51859273 | 0.52450653 | -0.53529328 | 1.57247874 | 601 |
| PC.ae.C42.2 | 0.00075 | 1.69719318 | 0.00732957 | 0.71314891 | 2.68123745 | 666 |
| PC.ae.C42.3 | 2.10E-01 | -0.62389363 | 0.3971382 | -1.60055825 | 0.352771 | 714 |
| PC.ae.C42.4 | 0.89222604 | -0.06630791 | 0.94033627 | -1.02684258 | 0.89422676 | 706 |
| PC.ae.C42.5 | 0.23461606 | -0.61135878 | 0.42388615 | -1.62045152 | 0.39773397 | 666 |
| PC.ae.C42.6 | 0.30677264 | -0.49358884 | 0.49220983 | -1.44105744 | 0.45387976 | 756 |
| SM.a.C30.1 | 0.64676111 | -0.24676467 | 0.78120021 | -1.30365029 | 0.81012095 | 655 |
| SM.a.C32.0 | 0.96784381 | -0.02610382 | 0.98153972 | -1.29808672 | 1.24587908 | 443 |
| SM.a.C32.1 | 0.74017499 | -0.16545057 | 0.841998 | -1.144624 | 0.81372285 | 706 |
| SM.a.C32.2 | 9.94E-02 | -0.82525727 | 2.57E-01 | -1.80731743 | 0.1568029 | 716 |
| SM.a.C33.1 | 0.95626721 | 0.02684843 | 0.97903548 | -0.9340668 | 0.98776365 | 706 |
| SM.a.C33.2 | 4.34E-01 | -0.41699714 | 6.06E-01 | -1.46380592 | 0.62981164 | 615 |
| SM.a.C34.0 | 0.01680983 | -1.47113934 | 0.06692801 | -2.67597017 | -0.2663085 | 493 |
| SM.a.C34.1 | 0.21964351 | -0.60113826 | 0.40361842 | -1.5617995 | 0.35952298 | 717 |
| SM.a.C34.2 | 3.44E-02 | -1.01697093 | 1.10E-01 | -1.95877867 | -0.07516318 | 757 |
| SM.a.C34.3 | 0.20484054 | -0.74818205 | 0.3971382 | -1.90610046 | 0.40973636 | 498 |
| SM.a.C35.0 | 0.06620612 | -1.00862345 | 0.19235562 | -2.08497533 | 0.06772842 | 564 |
| SM.a.C35.1 | 0.28654212 | -0.52161959 | 0.48086299 | -1.48185248 | 0.4386133 | 706 |
| SM.a.C36.0 | 1.87E-01 | 0.81775411 | 3.86E-01 | -0.39717634 | 2.03268457 | 498 |
| SM.a.C36.1 | 0.40394504 | -0.41499807 | 0.58681206 | -1.39067036 | 0.56067422 | 717 |
| SM.a.C36.2 | 2.76E-02 | -1.03925869 | 9.49E-02 | -1.96344129 | -0.11507609 | 757 |
| SM.a.C36.3 | 7.39E-02 | -0.86701737 | 0.20625064 | -1.81793898 | 0.08390424 | 716 |
| SM.a.C37.1 | 0.18381513 | -0.67877092 | 0.38579273 | -1.68052197 | 0.32298013 | 666 |
| SM.a.C38.1 | 0.45645199 | -0.35584114 | 0.63314309 | -1.29341167 | 0.58172938 | 755 |
| SM.a.C38.2 | 0.01930858 | -1.128939 | 0.0732979 | -2.07420295 | -0.18367504 | 757 |
| SM.a.C39.1 | 0.70379565 | 0.20014755 | 0.82236991 | -0.83309091 | 1.233386 | 666 |
| SM.a.C39.2 | 0.07772445 | -0.89625375 | 0.21152857 | -1.89230721 | 0.09979972 | 715 |
| SM.a.C40.1 | 0.10756446 | 0.81528206 | 0.27011783 | -0.17816851 | 1.80873262 | 661 |
| SM.a.C40.2 | 0.24669949 | -0.61245963 | 0.4347573 | -1.64977438 | 0.42485513 | 625 |
| SM.a.C40.3 | 0.50021849 | 0.38492463 | 0.66997794 | -0.73599378 | 1.50584304 | 536 |
| SM.a.C40.4 | 0.55348488 | 0.28041678 | 0.70413756 | -0.64820832 | 1.20904188 | 757 |
| SM.a.C40.5 | 0.02124338 | 1.13954754 | 0.07612211 | 0.17054132 | 2.10855376 | 675 |
| SM.a.C41.1 | 0.16769157 | 0.68373704 | 0.37951249 | -0.28827088 | 1.65574496 | 706 |
| SM.a.C41.2 | 0.03410783 | -1.05821075 | 0.11023 | -2.03686572 | -0.07955577 | 706 |
| SM.a.C42.1 | 0.00429184 | 1.37776055 | 0.02796198 | 0.43369037 | 2.32183074 | 706 |
| SM.a.C42.2 | 0.4910439 | -0.3413129 | 0.6676299 | -1.3139067 | 0.6312809 | 706 |
| SM.a.C42.3 | 0.00551323 | -1.39598899 | 0.03284458 | -2.38044433 | -0.41153365 | 706 |
| SM.a.C42.4 | 0.29453101 | 0.52861368 | 0.48339059 | -0.46081941 | 1.51804677 | 665 |
| SM.a.C42.5 | 0.60371521 | -0.31925664 | 0.74170726 | -1.52697855 | 0.88846526 | 498 |
| SM.a.C42.6 | 0.28784068 | -0.55820771 | 0.48086299 | -1.58863279 | 0.47221736 | 665 |
| SM.a.C43.1 | 0.18993005 | 0.65231089 | 0.38890439 | -0.32381194 | 1.62843372 | 706 |
| SM.a.C43.2 | 0.59139827 | 0.26555843 | 0.73497473 | -0.70527564 | 1.23639249 | 706 |
| SM.a.C44.2 | 0.83524353 | 0.11575178 | 0.89341969 | -0.97697571 | 1.20847928 | 556 |
| SM.a.C44.6 | 0.17917535 | 0.7357175 | 0.38579273 | -0.33864084 | 1.81007584 | 624 |
| SM.a.C31.1 | 0.3672153 | -0.47098547 | 0.54076226 | -1.4959253 | 0.55395436 | 665 |
| SM.a.C33.3 | 0.34494793 | -0.46632422 | 0.53234815 | -1.43509676 | 0.50244833 | 715 |
| SM.a.C35.2 | 0.4937356 | -0.3329153 | 0.6676299 | -1.28748232 | 0.62165172 | 717 |
| SM.a.C37.3 | 1.22E-01 | -0.76044835 | 0.29897344 | -1.72570432 | 0.20480762 | 717 |
| SM.a.C39.5 | 0.00966771 | 1.41384012 | 0.04544578 | 0.34419827 | 2.48348197 | 598 |
| SM.a.C41.0 | 0.24045504 | -0.62745272 | 0.43081527 | -1.67619713 | 0.42129168 | 605 |
| SM.a.C41.3 | 0.19261166 | -0.72810109 | 0.38906405 | -1.82449234 | 0.36829016 | 550 |
| SM.a.C43.0 | 0.42236489 | -0.47831074 | 0.60138047 | -1.64853437 | 0.69191288 | 539 |
| SM.a.C43.3 | 0.36326761 | -0.49618405 | 0.53863818 | -1.56719989 | 0.5748318 | 616 |
| Ala | 0.15154026 | 0.67585744 | 0.350335 | -0.24837723 | 1.6000921 | 754 |
| Arg | 0.71819935 | 0.17535667 | 0.82332948 | -0.77825274 | 1.12896608 | 752 |
| Asn | 0.68109641 | -0.19672152 | 0.80903717 | -1.13616771 | 0.74272466 | 703 |
| Asp | 0.7169262 | 0.16910607 | 0.82332948 | -0.74618756 | 1.08439971 | 754 |
| Cit | 0.4307922 | 0.3801581 | 0.60536159 | -0.56662209 | 1.3269383 | 754 |
| Gln | 0.58268469 | 0.26711796 | 0.73261525 | -0.68692423 | 1.22116015 | 703 |
| Glu | 1.60E-04 | 1.84410504 | 2.65E-03 | 0.89004829 | 2.7981618 | 754 |
| Gly | 0.14267519 | -0.69602167 | 0.33342572 | -1.62716124 | 0.2351179 | 754 |
| His | 0.71993462 | 0.16737111 | 0.82332948 | -0.74871656 | 1.08345877 | 743 |
| Ile | 1.02E-02 | 1.4170657 | 0.04683518 | 0.33679555 | 2.49733584 | 543 |
| Leu | 1.21E-05 | 2.24818285 | 4.35E-04 | 1.24667718 | 3.24968852 | 703 |
| Lys | 0.82757321 | -0.10696879 | 0.8896412 | -1.07083289 | 0.8568953 | 703 |
| Met | 0.17386688 | 0.70658859 | 0.38579273 | -0.31253635 | 1.72571352 | 703 |
| Orn | 0.10804713 | 0.81852652 | 0.27011783 | -0.18021868 | 1.81727172 | 703 |
| Phe | 1.99E-02 | 1.17018518 | 0.0732979 | 0.18578097 | 2.15458939 | 703 |
| Pro | 0.00747133 | 1.30609932 | 0.03735667 | 0.35022293 | 2.26197571 | 754 |
| Trp | 0.02805673 | 1.04071569 | 0.09488424 | 0.11237786 | 1.96905351 | 754 |
| Ser | 1.8235E-05 | -1.98079504 | 0.00056007 | -2.88226503 | -1.07932505 | 754 |
| Thr | 0.00937316 | -1.25425395 | 0.04544578 | -2.19944549 | -0.30906241 | 714 |
| Tyr | 6.35E-02 | 0.92247799 | 1.89E-01 | -0.05209077 | 1.89704675 | 703 |
| Val | 5.28E-04 | 1.74433749 | 5.98E-03 | 0.76088975 | 2.72778524 | 703 |
| NEFA_12_0 | 0.56035284 | 0.31402746 | 0.70868153 | -0.74450048 | 1.37255541 | 602 |
| NEFA_14_0 | 0.331686 | 0.47666016 | 0.52435654 | -0.48672716 | 1.44004747 | 731 |
| NEFA_14_1 | 0.48067738 | 0.33867654 | 0.65825246 | -0.60366086 | 1.28101395 | 750 |
| NEFA_15_0 | 0.00972328 | 1.24645927 | 0.04544578 | 0.30249247 | 2.19042607 | 747 |
| NEFA_15_1 | 0.9191503 | -0.05899804 | 0.95930735 | -1.20042217 | 1.08242609 | 506 |
| NEFA_16_0 | 0.01180602 | 1.30282152 | 0.05180193 | 0.28969709 | 2.31594594 | 641 |
| NEFA_16_1 | 0.89063238 | 0.06510059 | 0.94033627 | -0.864041 | 0.99424217 | 750 |
| NEFA_17_0 | 0.00070361 | 1.71857546 | 0.00720366 | 0.72722858 | 2.70992234 | 692 |
| NEFA_17_1 | 0.02011431 | 1.18704509 | 0.0732979 | 0.18661916 | 2.18747102 | 679 |
| NEFA_18_0 | 0.00125791 | 1.72837287 | 0.01064227 | 0.68087815 | 2.77586758 | 636 |
| NEFA_18_1 | 0.20247565 | 0.63920407 | 0.3971382 | -0.34456237 | 1.6229705 | 694 |
| NEFA_18_2 | 0.2113166 | 0.66029171 | 0.3971382 | -0.3760049 | 1.69658832 | 643 |
| NEFA_18_3 | 0.29049015 | 0.49874649 | 0.48086299 | -0.4268804 | 1.42437337 | 750 |
| NEFA_20_1 | 0.18609718 | 0.66713793 | 0.38579273 | -0.32258088 | 1.65685673 | 665 |
| NEFA_20_2 | 0.27526854 | 0.56073154 | 0.46970426 | -0.44768519 | 1.56914827 | 633 |
| NEFA_20_3 | 0.00019448 | 1.93743292 | 0.00265198 | 0.92239099 | 2.95247486 | 643 |
| NEFA_20_4 | 0.00017549 | 1.96371003 | 0.00265198 | 0.94206563 | 2.98535443 | 643 |
| NEFA_22_6 | 0.06430537 | 0.95931102 | 0.18939254 | -0.05714699 | 1.97576903 | 694 |
| NEFA_24_1 | 0.01588222 | 1.14319716 | 0.06442787 | 0.21472296 | 2.07167136 | 749 |
| NEFA_12_1 | 0.5984068 | 0.26162555 | 0.7394107 | -0.71324169 | 1.23649279 | 688 |
| NEFA_13_1 | 0.10055058 | 1.19188817 | 0.2573616 | -0.23171193 | 2.61548827 | 393 |
| NEFA_14_2 | 0.29833994 | 0.56067192 | 0.48593248 | -0.4971978 | 1.61854165 | 603 |
| NEFA_16_2 | 0.0810752 | 0.8848408 | 0.21788959 | -0.10960536 | 1.87928696 | 659 |
| NEFA_18_4 | 0.11715628 | 0.87134261 | 0.28952415 | -0.21937111 | 1.96205634 | 527 |
| NEFA_19_0 | 0.00465351 | 1.57740236 | 0.02858585 | 0.48700509 | 2.66779962 | 577 |
| NEFA_19_1 | 0.01756341 | 1.23992596 | 0.06865696 | 0.21726944 | 2.26258249 | 653 |
| NEFA_20_5 | 2.99E-05 | 2.1336946 | 7.15E-04 | 1.13709241 | 3.13029679 | 653 |
| NEFA_22_4 | 0.01518172 | 1.30382038 | 0.06334807 | 0.25230031 | 2.35534044 | 577 |
| NEFA_22_5 | 0.00063243 | 1.74949359 | 0.00679861 | 0.74916939 | 2.7498178 | 653 |
| NEFA_24_4 | 0.80667395 | -0.15449859 | 0.8803802 | -1.39493143 | 1.08593425 | 399 |
| NEFA_24_5 | 0.53107318 | -0.38053029 | 0.68973483 | -1.57356786 | 0.81250728 | 456 |
| NEFA_26_1 | 5.7666E-06 | 2.78385994 | 0.00036583 | 1.59004471 | 3.97767516 | 549 |

## Supplemental Table 2: Results of the Regression model with Metabolite concentration as outcome and two level sex variable as predictor (males versus *non-HC* females), adjusted for ethnicity. P-values, beta coefficient, Confidence interval (CI), number of observations per metabolite and FDR corrected p-values reported for all metabolites.

| Analytes | P-Value | BETA | FDR P-Value | CI lower | CI upper | Number of observations |
| --- | --- | --- | --- | --- | --- | --- |
| Carn | 1.6204E-11 | 0.51486761 | 1.8336E-10 | 0.36721336 | 0.66252186 | 729 |
| Carn.a.C10.0 | 0.41465796 | 0.0818381 | 0.48569664 | -0.11513698 | 0.27881319 | 463 |
| Carn.a.C10.1 | 0.67508678 | 0.04402226 | 0.72936511 | -0.16223299 | 0.25027751 | 455 |
| Carn.a.C12.0 | 0.00018506 | 0.39132833 | 0.00054503 | 0.18732518 | 0.59533149 | 455 |
| Carn.a.C12.1 | 0.1605976 | 0.15643801 | 0.21992665 | -0.06234493 | 0.37522094 | 410 |
| Carn.a.C14.0 | 0.00010919 | 0.34570584 | 0.00035038 | 0.17139747 | 0.52001421 | 605 |
| Carn.a.C14.1 | 0.62256791 | 0.04366527 | 0.68291888 | -0.13044692 | 0.21777746 | 656 |
| Carn.a.C14.2 | 0.00753527 | 0.2367752 | 0.0152838 | 0.06333122 | 0.41021917 | 651 |
| Carn.a.C16.0 | 2.2591E-07 | 0.4243559 | 1.3127E-06 | 0.2649633 | 0.58374849 | 726 |
| Carn.a.C16.1 | 0.69319375 | -0.0342714 | 0.74268608 | -0.20477019 | 0.13622738 | 654 |
| Carn.a.C18.0 | 3.4979E-05 | 0.37630792 | 0.0001213 | 0.19909279 | 0.55352306 | 591 |
| Carn.a.C18.1 | 0.01429501 | 0.19930348 | 0.0262686 | 0.03996699 | 0.35863996 | 729 |
| Carn.a.C2.0 | 0.97854091 | -0.00236796 | 0.97854091 | -0.17515767 | 0.17042175 | 677 |
| Carn.a.C3.0 | 7.12E-12 | 0.56523561 | 9.00E-11 | 0.40603322 | 0.72443799 | 728 |
| Carn.a.C4.0 | 5.7536E-07 | 0.44225488 | 2.8768E-06 | 0.27032362 | 0.61418614 | 634 |
| Carn.a.C4.0.DC | 9.0029E-07 | 0.53090569 | 4.3014E-06 | 0.32175834 | 0.74005304 | 404 |
| Carn.a.C5.0 | 1.9794E-08 | 0.56056177 | 1.3728E-07 | 0.36760213 | 0.75352141 | 495 |
| Carn.a.C6.0 | 0.81177217 | 0.02078841 | 0.84723794 | -0.15056569 | 0.19214252 | 628 |
| Carn.a.C8.1 | 0.12965276 | -0.14784304 | 0.18339042 | -0.33918207 | 0.043496 | 558 |
| lyso.PC.a.C14.0 | 0.02085729 | 0.19722468 | 0.03706047 | 0.03001298 | 0.36443638 | 724 |
| lyso.PC.a.C16.0 | 8.8787E-09 | 0.45802507 | 7.342E-08 | 0.3036167 | 0.61243345 | 677 |
| lyso.PC.a.C16.1 | 0.01230301 | 0.21154712 | 0.02320305 | 0.04605647 | 0.37703777 | 729 |
| lyso.PC.a.C18.0 | 0.19965924 | 0.08878915 | 0.26174839 | -0.04700208 | 0.22458037 | 729 |
| lyso.PC.a.C18.1 | 1.27E-06 | 0.37503013 | 5.88E-06 | 0.22441359 | 0.52564667 | 677 |
| lyso.PC.a.C18.2 | 2.70E-07 | 0.40307232 | 1.49E-06 | 0.25075373 | 0.5553909 | 677 |
| lyso.PC.a.C18.3 | 0.00012675 | 0.36056744 | 0.00039764 | 0.17703909 | 0.54409578 | 586 |
| lyso.PC.a.C18.6 | 0.35220332 | -0.08733358 | 0.42471371 | -0.2715873 | 0.09692013 | 524 |
| lyso.PC.a.C20.2 | 0.03933678 | 0.21838272 | 0.06456036 | 0.01071474 | 0.4260507 | 475 |
| lyso.PC.a.C20.3 | 1.0055E-18 | 0.74212995 | 3.0884E-17 | 0.58232517 | 0.90193473 | 632 |
| lyso.PC.a.C20.4 | 4.5443E-18 | 0.65903702 | 1.2213E-16 | 0.51360126 | 0.80447279 | 729 |
| lyso.PC.a.C20.5 | 4.3059E-07 | 0.41588448 | 2.258E-06 | 0.25592093 | 0.57584804 | 682 |
| lyso.PC.a.C22.5 | 3.8013E-12 | 0.54388878 | 5.108E-11 | 0.39303258 | 0.69474498 | 645 |
| lyso.PC.a.C22.6 | 0.00239835 | 0.26177957 | 0.00560484 | 0.09310663 | 0.43045252 | 688 |
| lyso.PC.e.C16.0 | 0.01087397 | 0.20447917 | 0.02068942 | 0.0472552 | 0.36170314 | 728 |
| lyso.PC.e.C18.0 | 5.4501E-06 | 0.3555589 | 2.2976E-05 | 0.20320093 | 0.50791687 | 708 |
| lyso.PC.e.C18.1 | 2.5182E-06 | 0.4502694 | 1.1279E-05 | 0.26441475 | 0.63612406 | 524 |
| PC.aa.C30.0 | 0.69432512 | -0.02743206 | 0.74268608 | -0.1644192 | 0.10955507 | 722 |
| PC.aa.C30.1 | 0.00304598 | -0.2825186 | 0.00696687 | -0.4689533 | -0.09608389 | 526 |
| PC.aa.C30.2 | 0.00050146 | -0.33729719 | 0.00134768 | -0.52643908 | -0.1481553 | 478 |
| PC.aa.C32.0 | 0.0959692 | -0.12118355 | 0.14428935 | -0.26391234 | 0.02154523 | 729 |
| PC.aa.C32.1 | 0.47097521 | 0.05581522 | 0.53141885 | -0.09613654 | 0.20776698 | 630 |
| PC.aa.C32.2 | 0.02151993 | -0.16983956 | 0.03792448 | -0.31456314 | -0.02511598 | 610 |
| PC.aa.C32.3 | 0.02519722 | -0.19184909 | 0.04299526 | -0.35975886 | -0.02393932 | 636 |
| PC.aa.C34.0 | 0.41566597 | -0.08296139 | 0.48569664 | -0.28303234 | 0.11710956 | 523 |
| PC.aa.C34.1 | 0.24218451 | 0.09603368 | 0.30810455 | -0.06504984 | 0.25711719 | 682 |
| PC.aa.C34.2 | 0.00518204 | -0.20967746 | 0.01114139 | -0.35646861 | -0.06288631 | 677 |
| PC.aa.C34.3 | 0.01704651 | -0.18555695 | 0.03054166 | -0.3378957 | -0.03321821 | 677 |
| PC.aa.C34.4 | 0.53041809 | 0.04639658 | 0.59088025 | -0.09875059 | 0.19154375 | 631 |
| PC.aa.C34.5 | 0.12420133 | 0.13688735 | 0.17921669 | -0.03772866 | 0.31150336 | 639 |
| PC.aa.C36.0 | 0.35002188 | -0.0816972 | 0.42471371 | -0.25322463 | 0.08983023 | 676 |
| PC.aa.C36.1 | 0.08330375 | -0.1564101 | 0.12978483 | -0.33347952 | 0.02065932 | 676 |
| PC.aa.C36.2 | 5.564E-07 | -0.43267175 | 2.8482E-06 | -0.60074704 | -0.26459647 | 677 |
| PC.aa.C36.3 | 0.39634553 | -0.06173581 | 0.47079718 | -0.20456054 | 0.08108893 | 683 |
| PC.aa.C36.4 | 0.0590595 | 0.14099062 | 0.09475964 | -0.00540966 | 0.28739089 | 631 |
| PC.aa.C36.5 | 0.4016055 | 0.07635169 | 0.47442408 | -0.10230184 | 0.25500522 | 590 |
| PC.aa.C36.6 | 0.10599907 | -0.11342146 | 0.15609452 | -0.25100622 | 0.02416331 | 718 |
| PC.aa.C38.0 | 0.00078772 | -0.28760871 | 0.00204047 | -0.45502957 | -0.12018786 | 635 |
| PC.aa.C38.1 | 0.00923782 | -0.23703514 | 0.01822138 | -0.41527844 | -0.05879184 | 581 |
| PC.aa.C38.2 | 0.00084879 | -0.25387491 | 0.00217251 | -0.40261624 | -0.10513358 | 686 |
| PC.aa.C38.3 | 2.04E-01 | -0.11096327 | 2.66E-01 | -0.28236799 | 0.06044146 | 610 |
| PC.aa.C38.4 | 0.18515446 | -0.12125664 | 0.24656309 | -0.30076921 | 0.05825593 | 610 |
| PC.aa.C38.5 | 0.5181322 | -0.05916493 | 0.58020012 | -0.23886173 | 0.12053188 | 610 |
| PC.aa.C38.6 | 0.00204826 | -0.22772466 | 0.00494804 | -0.37215172 | -0.0832976 | 610 |
| PC.aa.C40.0 | 6.9274E-05 | -0.34918432 | 0.00022566 | -0.52032727 | -0.17804137 | 598 |
| PC.aa.C40.1 | 0.07004762 | -0.18617754 | 0.10992875 | -0.38766826 | 0.01531318 | 430 |
| PC.aa.C40.3 | 0.18578242 | -0.13630429 | 0.24656309 | -0.3384535 | 0.06584491 | 435 |
| PC.aa.C40.4 | 0.42775723 | 0.06669274 | 0.48660214 | -0.09833321 | 0.2317187 | 677 |
| PC.aa.C40.5 | 4.27E-01 | -0.07225017 | 4.87E-01 | -0.25081546 | 0.10631512 | 610 |
| PC.aa.C40.6 | 3.1886E-05 | -0.3345521 | 0.00011619 | -0.49138075 | -0.17772346 | 658 |
| PC.aa.C42.0 | 2.5201E-07 | -0.45087552 | 1.4258E-06 | -0.62060359 | -0.28114745 | 591 |
| PC.aa.C42.1 | 0.01639478 | -0.23203877 | 0.02962083 | -0.42134257 | -0.04273496 | 481 |
| PC.aa.C42.2 | 0.05518348 | -0.19305496 | 0.08920638 | -0.39041004 | 0.00430013 | 469 |
| PC.aa.C42.4 | 0.08843204 | -0.17135001 | 0.13484319 | -0.36858665 | 0.02588662 | 403 |
| PC.aa.C42.5 | 0.00051949 | -0.28958109 | 0.0013789 | -0.45250472 | -0.12665746 | 547 |
| PC.aa.C42.6 | 0.00640882 | -0.24323847 | 0.01350879 | -0.41781741 | -0.06865953 | 526 |
| PC.aa.C43.4 | 0.00117374 | -0.31972389 | 0.00293434 | -0.51212423 | -0.12732355 | 465 |
| PC.aa.C43.6 | 0.00023236 | -0.30320551 | 0.0006658 | -0.46406837 | -0.14234265 | 677 |
| PC.aa.C44.12 | 0.00696233 | -0.22755659 | 0.01453302 | -0.39261475 | -0.06249843 | 677 |
| PC.ae.C30.0 | 0.26705841 | -0.09530203 | 0.33495341 | -0.26377579 | 0.07317172 | 632 |
| PC.ae.C30.1 | 0.00293161 | -0.28577639 | 0.00677737 | -0.47363894 | -0.09791384 | 563 |
| PC.ae.C32.0 | 0.04768224 | -0.154197 | 0.07766425 | -0.30681484 | -0.00157915 | 729 |
| PC.ae.C32.1 | 0.0003551 | -0.29355356 | 0.00097881 | -0.45415999 | -0.13294714 | 729 |
| PC.ae.C32.2 | 2.0205E-07 | -0.41365037 | 1.2411E-06 | -0.56839199 | -0.25890875 | 728 |
| PC.ae.C34.0 | 0.93782274 | 0.00659284 | 0.95109381 | -0.1592906 | 0.17247628 | 682 |
| PC.ae.C34.1 | 0.00330055 | -0.24968616 | 0.00746966 | -0.41594505 | -0.08342727 | 683 |
| PC.ae.C34.2 | 8.42E-07 | -0.41860614 | 4.12E-06 | -0.58393424 | -0.25327804 | 683 |
| PC.ae.C34.3 | 1.16E-01 | -0.13714074 | 1.69E-01 | -0.30809536 | 0.03381388 | 683 |
| PC.ae.C34.4 | 0.76947552 | -0.02669058 | 0.81515968 | -0.20545781 | 0.15207665 | 641 |
| PC.ae.C36.0 | 0.12618186 | -0.13572948 | 0.18086067 | -0.30977401 | 0.03831506 | 683 |
| PC.ae.C36.1 | 0.30724848 | 0.09268287 | 0.37964611 | -0.08541877 | 0.27078451 | 677 |
| PC.ae.C36.2 | 0.01340784 | -0.20648479 | 0.02506684 | -0.37001176 | -0.04295783 | 677 |
| PC.ae.C36.3 | 3.59E-03 | -0.2430993 | 0.00795097 | -0.40643932 | -0.07975928 | 729 |
| PC.ae.C36.4 | 0.0848855 | 0.1597354 | 0.13035988 | -0.02202986 | 0.34150067 | 631 |
| PC.ae.C36.5 | 0.00862864 | 0.24721842 | 0.01733792 | 0.06295719 | 0.43147964 | 631 |
| PC.ae.C36.6 | 0.1016493 | 0.16351584 | 0.15072137 | -0.03237638 | 0.35940805 | 543 |
| PC.ae.C38.0 | 0.00175147 | -0.25651742 | 0.00432835 | -0.41681945 | -0.09621539 | 677 |
| PC.ae.C38.2 | 3.3318E-05 | -0.34996048 | 0.00011939 | -0.51443845 | -0.18548251 | 677 |
| PC.ae.C38.3 | 0.31581809 | -0.08408315 | 0.38800508 | -0.24854872 | 0.08038243 | 677 |
| PC.ae.C38.4 | 0.42237303 | 0.07320862 | 0.48660214 | -0.10586066 | 0.2522779 | 631 |
| PC.ae.C38.5 | 0.66152707 | 0.04025444 | 0.71832485 | -0.1402185 | 0.22072737 | 631 |
| PC.ae.C38.6 | 0.16983607 | -0.11866622 | 0.22965255 | -0.28821834 | 0.05088589 | 682 |
| PC.ae.C40.0 | 1.5769E-05 | -0.32254933 | 6.1641E-05 | -0.46816315 | -0.17693551 | 677 |
| PC.ae.C40.1 | 0.35359886 | -0.07886957 | 0.42471371 | -0.24569526 | 0.08795612 | 677 |
| PC.ae.C40.2 | 3.9732E-05 | -0.31971248 | 0.00013347 | -0.47142445 | -0.16800051 | 635 |
| PC.ae.C40.3 | 9.2018E-06 | -0.38283891 | 3.6637E-05 | -0.55096416 | -0.21471366 | 636 |
| PC.ae.C40.4 | 0.014083 | -0.20997866 | 0.02610211 | -0.37747074 | -0.04248658 | 677 |
| PC.ae.C40.5 | 0.08431679 | -0.14978541 | 0.13035988 | -0.31991231 | 0.0203415 | 677 |
| PC.ae.C40.6 | 0.00748821 | -0.23855522 | 0.0152838 | -0.41314991 | -0.06396052 | 610 |
| PC.ae.C42.0 | 0.00019958 | -0.24828239 | 0.00057987 | -0.37859866 | -0.11796611 | 641 |
| PC.ae.C42.1 | 0.84317135 | -0.01769917 | 0.87154731 | -0.19332494 | 0.1579266 | 595 |
| PC.ae.C42.2 | 0.00559658 | -0.24829825 | 0.01191351 | -0.42368644 | -0.07291005 | 636 |
| PC.ae.C42.3 | 6.39E-10 | -0.49610012 | 5.7227E-09 | -0.65144237 | -0.34075787 | 685 |
| PC.ae.C42.4 | 7.7141E-08 | -0.44743747 | 4.8781E-07 | -0.60911741 | -0.28575753 | 677 |
| PC.ae.C42.5 | 5.738E-06 | -0.38998801 | 2.3724E-05 | -0.55739071 | -0.22258531 | 636 |
| PC.ae.C42.6 | 1.2855E-06 | -0.38546483 | 5.8806E-06 | -0.54043899 | -0.23049067 | 727 |
| SM.a.C30.1 | 2.106E-10 | -0.53994475 | 2.0581E-09 | -0.70409635 | -0.37579315 | 632 |
| SM.a.C32.0 | 0.0043747 | -0.30723929 | 0.00950062 | -0.51799752 | -0.09648106 | 417 |
| SM.a.C32.1 | 1.9095E-05 | -0.36146314 | 7.2024E-05 | -0.52628701 | -0.19663927 | 677 |
| SM.a.C32.2 | 6.08E-47 | -1.07588652 | 1.31E-44 | -1.2116507 | -0.94012234 | 685 |
| SM.a.C33.1 | 0.00015081 | -0.33145334 | 0.00046322 | -0.50220437 | -0.1607023 | 677 |
| SM.a.C33.2 | 6.53E-19 | -0.79690495 | 2.34E-17 | -0.96716187 | -0.62664804 | 592 |
| SM.a.C34.0 | 0.0001789 | -0.38961121 | 0.00053421 | -0.59225117 | -0.18697125 | 455 |
| SM.a.C34.1 | 1.9489E-08 | -0.48152309 | 1.3728E-07 | -0.64785985 | -0.31518632 | 688 |
| SM.a.C34.2 | 3.82E-36 | -0.98463219 | 4.11E-34 | -1.13029229 | -0.8389721 | 729 |
| SM.a.C34.3 | 3.1335E-11 | -0.68027448 | 3.3685E-10 | -0.87684892 | -0.48370003 | 481 |
| SM.a.C35.0 | 7.5252E-06 | -0.42756756 | 3.0527E-05 | -0.61329702 | -0.24183811 | 545 |
| SM.a.C35.1 | 3.4092E-05 | -0.35847217 | 0.00012016 | -0.52716714 | -0.1897772 | 677 |
| SM.a.C36.0 | 3.22E-02 | -0.21120004 | 5.37E-02 | -0.40443049 | -0.01796959 | 481 |
| SM.a.C36.1 | 6.3109E-14 | -0.63082239 | 1.2335E-12 | -0.79249373 | -0.46915106 | 688 |
| SM.a.C36.2 | 2.84E-33 | -0.9592641 | 2.04E-31 | -1.10818067 | -0.81034754 | 729 |
| SM.a.C36.3 | 3.29E-20 | -0.77252776 | 1.4155E-18 | -0.93206222 | -0.6129933 | 682 |
| SM.a.C37.1 | 2.5532E-08 | -0.48929131 | 1.7154E-07 | -0.65962674 | -0.31895589 | 636 |
| SM.a.C38.1 | 0.02455595 | -0.16645439 | 0.04223623 | -0.31149969 | -0.02140909 | 728 |
| SM.a.C38.2 | 2.26E-12 | -0.51270391 | 3.4708E-11 | -0.6536659 | -0.37174193 | 729 |
| SM.a.C39.1 | 0.00056436 | -0.30997957 | 0.00147972 | -0.48561243 | -0.13434671 | 636 |
| SM.a.C39.2 | 1.4557E-13 | -0.61802788 | 2.6081E-12 | -0.77886418 | -0.45719157 | 684 |
| SM.a.C40.1 | 0.47209767 | -0.06327228 | 0.53141885 | -0.2359617 | 0.10941715 | 631 |
| SM.a.C40.2 | 2.0517E-10 | -0.55660132 | 2.0581E-09 | -0.72552817 | -0.38767447 | 590 |
| SM.a.C40.3 | 0.95648615 | -0.00437606 | 0.96546725 | -0.1618559 | 0.15310378 | 527 |
| SM.a.C40.4 | 0.23812878 | 0.08592209 | 0.30474814 | -0.05695322 | 0.2287974 | 728 |
| SM.a.C40.5 | 0.82621686 | 0.01904813 | 0.85814795 | -0.15124658 | 0.18934284 | 641 |
| SM.a.C41.1 | 0.26685284 | -0.09359704 | 0.33495341 | -0.25897409 | 0.07178001 | 677 |
| SM.a.C41.2 | 1.0562E-12 | -0.60193619 | 1.7468E-11 | -0.76468841 | -0.43918396 | 677 |
| SM.a.C42.1 | 0.15486829 | 0.11860865 | 0.21518774 | -0.04491925 | 0.28213656 | 677 |
| SM.a.C42.2 | 0.00023535 | -0.28688108 | 0.0006658 | -0.43921978 | -0.13454239 | 677 |
| SM.a.C42.3 | 2.9292E-17 | -0.65798966 | 6.9975E-16 | -0.8068072 | -0.50917212 | 677 |
| SM.a.C42.4 | 0.0021795 | -0.27220008 | 0.00518158 | -0.44589951 | -0.09850066 | 631 |
| SM.a.C42.5 | 0.03635016 | -0.18609124 | 0.06011758 | -0.36030578 | -0.0118767 | 481 |
| SM.a.C42.6 | 0.00346855 | -0.20921891 | 0.00776811 | -0.34925158 | -0.06918625 | 631 |
| SM.a.C43.1 | 0.26796273 | -0.09702099 | 0.33495341 | -0.26884635 | 0.07480438 | 677 |
| SM.a.C43.2 | 0.13238256 | -0.12406133 | 0.18602778 | -0.28574571 | 0.03762305 | 677 |
| SM.a.C44.2 | 0.27512524 | -0.10514852 | 0.34191865 | -0.29421815 | 0.08392111 | 548 |
| SM.a.C44.6 | 0.00024874 | -0.32032558 | 0.00069454 | -0.49100248 | -0.14964868 | 595 |
| SM.a.C31.1 | 1.2719E-08 | -0.49941967 | 1.0128E-07 | -0.66951336 | -0.32932598 | 635 |
| SM.a.C33.3 | 0.02371688 | -0.19613451 | 0.041122 | -0.36602438 | -0.02624465 | 687 |
| SM.a.C35.2 | 8.755E-12 | -0.59766112 | 1.0457E-10 | -0.76660408 | -0.42871816 | 688 |
| SM.a.C37.3 | 1.13E-03 | -0.27511233 | 0.00286829 | -0.4403596 | -0.10986507 | 687 |
| SM.a.C39.5 | 0.00416069 | 0.27914793 | 0.00912805 | 0.08859725 | 0.46969861 | 570 |
| SM.a.C41.0 | 0.02725751 | -0.19659471 | 0.0461446 | -0.37103988 | -0.02214955 | 567 |
| SM.a.C41.3 | 1.4727E-08 | -0.529182 | 1.1308E-07 | -0.70985591 | -0.34850809 | 537 |
| SM.a.C43.0 | 2.3327E-05 | -0.39303935 | 8.6469E-05 | -0.5739137 | -0.21216499 | 527 |
| SM.a.C43.3 | 6.3176E-08 | -0.4814201 | 4.116E-07 | -0.65396654 | -0.30887366 | 593 |
| Ala | 0.89769934 | 0.01048302 | 0.91471733 | -0.14953727 | 0.1705033 | 727 |
| Arg | 0.0236808 | 0.1882706 | 0.041122 | 0.02523052 | 0.35131069 | 726 |
| Asn | 0.01058107 | 0.20760179 | 0.02031187 | 0.0485873 | 0.36661628 | 667 |
| Asp | 0.11942417 | 0.12158818 | 0.17348781 | -0.03152379 | 0.27470015 | 727 |
| Cit | 0.01451697 | 0.19387798 | 0.02645041 | 0.03852569 | 0.34923026 | 727 |
| Gln | 0.00017439 | 0.30332249 | 0.00052809 | 0.14554762 | 0.46109735 | 667 |
| Glu | 1.61E-05 | 0.3569722 | 6.18E-05 | 0.19557964 | 0.51836475 | 727 |
| Gly | 0.00012761 | -0.29191078 | 0.00039764 | -0.44069429 | -0.14312727 | 727 |
| His | 4.8465E-05 | 0.31031205 | 0.00016031 | 0.16127787 | 0.45934622 | 720 |
| Ile | 1.97E-08 | 0.53678334 | 1.3728E-07 | 0.35186299 | 0.72170368 | 530 |
| Leu | 3.12E-27 | 0.85605618 | 1.68E-25 | 0.70739359 | 1.00471877 | 667 |
| Lys | 0.00219313 | 0.26396676 | 0.00518158 | 0.09539764 | 0.43253589 | 667 |
| Met | 3.0259E-09 | 0.5042704 | 2.6022E-08 | 0.33957079 | 0.66897001 | 667 |
| Orn | 0.00947631 | 0.20849545 | 0.01852189 | 0.05115565 | 0.36583525 | 667 |
| Phe | 2.09E-07 | 0.43511709 | 1.2464E-06 | 0.27227277 | 0.59796141 | 667 |
| Pro | 3.617E-05 | 0.33857064 | 0.00012344 | 0.17865625 | 0.49848504 | 727 |
| Trp | 2.4907E-10 | 0.53073706 | 2.3283E-09 | 0.36839439 | 0.69307974 | 727 |
| Ser | 3.2158E-12 | -0.56508323 | 4.6094E-11 | -0.7215802 | -0.40858627 | 727 |
| Thr | 0.22254267 | -0.09472602 | 0.28650703 | -0.24706296 | 0.05761092 | 686 |
| Tyr | 1.80E-01 | 0.10592403 | 2.42E-01 | -0.04915976 | 0.26100782 | 667 |
| Val | 1.43E-16 | 0.67806802 | 3.08E-15 | 0.52110497 | 0.83503107 | 667 |
| NEFA_12_0 | 0.12872382 | -0.14181924 | 0.18328226 | -0.32491112 | 0.04127264 | 583 |
| NEFA_14_0 | 0.16018635 | -0.11813737 | 0.21992665 | -0.28311278 | 0.04683805 | 707 |
| NEFA_14_1 | 4.0344E-07 | -0.3928251 | 2.1685E-06 | -0.54361632 | -0.24203388 | 726 |
| NEFA_15_0 | 0.64606011 | -0.03914263 | 0.70509099 | -0.20640668 | 0.12812143 | 725 |
| NEFA_15_1 | 0.09778682 | -0.15398842 | 0.14600115 | -0.33637613 | 0.02839929 | 511 |
| NEFA_16_0 | 0.61457996 | -0.04537687 | 0.6776138 | -0.22225003 | 0.1314963 | 633 |
| NEFA_16_1 | 2.756E-06 | -0.36260487 | 1.2093E-05 | -0.51324512 | -0.21196462 | 726 |
| NEFA_17_0 | 0.21784852 | 0.11245562 | 0.2821532 | -0.06656259 | 0.29147384 | 679 |
| NEFA_17_1 | 0.06842475 | -0.15948663 | 0.10817148 | -0.33106466 | 0.01209141 | 668 |
| NEFA_18_0 | 0.77509864 | 0.02736771 | 0.81689318 | -0.16066028 | 0.2153957 | 618 |
| NEFA_18_1 | 0.03003947 | -0.18801509 | 0.05045692 | -0.35781153 | -0.01821865 | 682 |
| NEFA_18_2 | 0.09147474 | -0.14761028 | 0.13850049 | -0.31910456 | 0.023884 | 630 |
| NEFA_18_3 | 0.06311443 | -0.15013727 | 0.10051558 | -0.30850235 | 0.00822782 | 725 |
| NEFA_20_1 | 0.61442569 | -0.04408619 | 0.6776138 | -0.21584603 | 0.12767365 | 653 |
| NEFA_20_2 | 0.41961533 | -0.07015856 | 0.48660214 | -0.24075375 | 0.10043663 | 623 |
| NEFA_20_3 | 0.34099702 | 0.08127689 | 0.41655885 | -0.0862163 | 0.24877007 | 630 |
| NEFA_20_4 | 0.00705337 | 0.24170065 | 0.01458149 | 0.06611678 | 0.41728453 | 630 |
| NEFA_22_6 | 0.42382792 | -0.06488363 | 0.48660214 | -0.22407355 | 0.09430629 | 682 |
| NEFA_24_1 | 0.0019585 | 0.2662345 | 0.00478496 | 0.09805168 | 0.43441732 | 725 |
| NEFA_12_1 | 0.00043549 | -0.3002665 | 0.0011852 | -0.46702407 | -0.13350892 | 655 |
| NEFA_13_1 | 0.87295633 | 0.01804671 | 0.89801728 | -0.20370044 | 0.23979385 | 390 |
| NEFA_14_2 | 0.76966239 | -0.02638902 | 0.81515968 | -0.20330533 | 0.15052728 | 593 |
| NEFA_16_2 | 0.01029817 | -0.21420624 | 0.01994691 | -0.37766637 | -0.0507461 | 637 |
| NEFA_18_4 | 0.00886 | -0.25027169 | 0.01763795 | -0.43740701 | -0.06313637 | 521 |
| NEFA_19_0 | 0.15513535 | 0.14210216 | 0.21518774 | -0.0539704 | 0.33817472 | 564 |
| NEFA_19_1 | 0.39012711 | -0.07736206 | 0.46598516 | -0.25401478 | 0.09929065 | 636 |
| NEFA_20_5 | 1.99E-01 | 0.11677439 | 2.62E-01 | -0.0616783 | 0.29522707 | 636 |
| NEFA_22_4 | 0.96821588 | -0.00369174 | 0.97274026 | -0.18559427 | 0.17821079 | 564 |
| NEFA_22_5 | 0.88150882 | -0.01342194 | 0.90249713 | -0.19017529 | 0.1633314 | 636 |
| NEFA_24_4 | 0.16241188 | 0.12720676 | 0.22100351 | -0.05147742 | 0.30589095 | 394 |
| NEFA_24_5 | 0.80941033 | -0.01855343 | 0.84723794 | -0.16964537 | 0.13253851 | 449 |
| NEFA_26_1 | 4.1678E-06 | 0.46983262 | 1.7921E-05 | 0.27141406 | 0.66825117 | 531 |

## Supplemental Table 3: Results of the Regression model with Metabolite concentration as outcome and two level sex variable as predictor (*HC* females versus *non-HC* females), adjusted for ethnicity P-values, beta coefficient, Confidence interval (CI), number of observations per metabolite and FDR corrected p-values reported for all metabolites.

| Analytes | P-Value | BETA | FDR P-Value | CI lower | CI upper | Number of observations |
| --- | --- | --- | --- | --- | --- | --- |
| Carn | 1.6431E-13 | -0.59971081 | 2.5234E-12 | -0.75469173 | -0.4447299 | 460 |
| Carn.a.C10.0 | 0.73911453 | -0.04323946 | 0.84079167 | -0.29858104 | 0.21210211 | 279 |
| Carn.a.C10.1 | 0.29023533 | -0.13444234 | 0.40967023 | -0.38420696 | 0.11532229 | 280 |
| Carn.a.C12.0 | 0.95177317 | 0.00701418 | 0.97443443 | -0.22108869 | 0.23511704 | 278 |
| Carn.a.C12.1 | 0.41440139 | -0.10498396 | 0.53997757 | -0.35793488 | 0.14796697 | 242 |
| Carn.a.C14.0 | 0.34303802 | -0.09827177 | 0.46385644 | -0.30181043 | 0.10526689 | 375 |
| Carn.a.C14.1 | 0.30815556 | -0.09724181 | 0.42744158 | -0.28458236 | 0.09009873 | 411 |
| Carn.a.C14.2 | 0.0860842 | -0.16414299 | 0.15553028 | -0.35168065 | 0.02339467 | 404 |
| Carn.a.C16.0 | 0.00021211 | -0.30911317 | 0.00080005 | -0.47178041 | -0.14644593 | 456 |
| Carn.a.C16.1 | 0.01195185 | -0.25612421 | 0.02920054 | -0.45553391 | -0.05671451 | 410 |
| Carn.a.C18.0 | 2.6082E-07 | -0.48094426 | 1.3677E-06 | -0.6610643 | -0.30082423 | 358 |
| Carn.a.C18.1 | 2.1641E-08 | -0.47850833 | 1.2575E-07 | -0.64350761 | -0.31350904 | 459 |
| Carn.a.C2.0 | 0.00591225 | -0.27226306 | 0.0162966 | -0.46569499 | -0.07883112 | 425 |
| Carn.a.C3.0 | 1.58E-05 | -0.32276194 | 6.52E-05 | -0.46808432 | -0.17743955 | 459 |
| Carn.a.C4.0 | 0.0816398 | 0.16793197 | 0.15002185 | -0.02119189 | 0.35705582 | 393 |
| Carn.a.C4.0.DC | 0.442284 | -0.09542507 | 0.56940754 | -0.33966309 | 0.14881296 | 246 |
| Carn.a.C5.0 | 0.01441672 | -0.25140445 | 0.03406147 | -0.45242505 | -0.05038386 | 296 |
| Carn.a.C6.0 | 0.08412088 | -0.18451251 | 0.1532711 | -0.39400245 | 0.02497742 | 381 |
| Carn.a.C8.1 | 0.03368159 | -0.22875525 | 0.07151714 | -0.43975257 | -0.01775794 | 341 |
| lyso.PC.a.C14.0 | 0.09139777 | 0.15236859 | 0.162401 | -0.02463855 | 0.32937572 | 457 |
| lyso.PC.a.C16.0 | 0.00030309 | -0.34434189 | 0.00112353 | -0.53013646 | -0.15854732 | 425 |
| lyso.PC.a.C16.1 | 0.00601924 | -0.23720214 | 0.01638148 | -0.4061171 | -0.06828718 | 460 |
| lyso.PC.a.C18.0 | 8.3976E-25 | -0.97527479 | 1.8055E-22 | -1.15087906 | -0.79967052 | 460 |
| lyso.PC.a.C18.1 | 2.13E-16 | -0.73058288 | 6.77E-15 | -0.89835929 | -0.56280647 | 425 |
| lyso.PC.a.C18.2 | 2.64E-14 | -0.67367587 | 5.17E-13 | -0.84153088 | -0.50582085 | 425 |
| lyso.PC.a.C18.3 | 6.069E-07 | -0.43524129 | 3.1067E-06 | -0.60367857 | -0.26680401 | 356 |
| lyso.PC.a.C18.6 | 1.329E-09 | -0.70137774 | 9.2172E-09 | -0.92208413 | -0.48067134 | 314 |
| lyso.PC.a.C20.2 | 0.00676923 | -0.29809328 | 0.01802125 | -0.51319533 | -0.08299124 | 294 |
| lyso.PC.a.C20.3 | 6.5002E-05 | -0.32638429 | 0.00025881 | -0.48528709 | -0.16748148 | 386 |
| lyso.PC.a.C20.4 | 2.5587E-11 | -0.51209966 | 2.3918E-10 | -0.65923888 | -0.36496045 | 460 |
| lyso.PC.a.C20.5 | 4.051E-13 | -0.62147198 | 5.1233E-12 | -0.78447005 | -0.45847392 | 418 |
| lyso.PC.a.C22.5 | 1.4207E-16 | -0.70962573 | 6.109E-15 | -0.87113097 | -0.54812049 | 399 |
| lyso.PC.a.C22.6 | 3.1142E-09 | -0.49210035 | 2.0289E-08 | -0.65190814 | -0.33229256 | 432 |
| lyso.PC.e.C16.0 | 6.9595E-05 | -0.36261767 | 0.00027205 | -0.5401197 | -0.18511565 | 459 |
| lyso.PC.e.C18.0 | 8.1931E-10 | -0.54858678 | 6.0742E-09 | -0.72032623 | -0.37684734 | 446 |
| lyso.PC.e.C18.1 | 4.3245E-09 | -0.55355601 | 2.7346E-08 | -0.73379943 | -0.37331259 | 314 |
| PC.aa.C30.0 | 4.4076E-11 | 0.69779766 | 3.9484E-10 | 0.49476972 | 0.9008256 | 457 |
| PC.aa.C30.1 | 0.00571399 | 0.32193251 | 0.01595465 | 0.09433448 | 0.54953054 | 311 |
| PC.aa.C30.2 | 0.15713321 | 0.16783795 | 0.24480898 | -0.06505842 | 0.40073432 | 284 |
| PC.aa.C32.0 | 1.0358E-12 | 0.71081736 | 1.1458E-11 | 0.52031123 | 0.90132349 | 460 |
| PC.aa.C32.1 | 3.4466E-12 | 0.76801781 | 3.5286E-11 | 0.55782099 | 0.97821464 | 395 |
| PC.aa.C32.2 | 3.9077E-14 | 0.84945943 | 6.4627E-13 | 0.63709412 | 1.06182474 | 381 |
| PC.aa.C32.3 | 0.00413455 | 0.30392561 | 0.01201253 | 0.09678359 | 0.51106763 | 397 |
| PC.aa.C34.0 | 0.24091089 | 0.13092207 | 0.35700875 | -0.08832436 | 0.35016849 | 314 |
| PC.aa.C34.1 | 1.0658E-12 | 0.70203293 | 1.1458E-11 | 0.51411775 | 0.8899481 | 430 |
| PC.aa.C34.2 | 3.2411E-14 | 0.75127356 | 5.807E-13 | 0.56338814 | 0.93915897 | 425 |
| PC.aa.C34.3 | 1.2119E-09 | 0.60219245 | 8.6852E-09 | 0.41183058 | 0.79255432 | 425 |
| PC.aa.C34.4 | 8.6049E-13 | 0.81063225 | 1.0278E-11 | 0.5951417 | 1.02612279 | 395 |
| PC.aa.C34.5 | 0.51602448 | 0.06653458 | 0.6413021 | -0.13468163 | 0.2677508 | 400 |
| PC.aa.C36.0 | 0.94952819 | 0.00624117 | 0.97443443 | -0.18744761 | 0.19992995 | 425 |
| PC.aa.C36.1 | 0.13518943 | -0.1405112 | 0.21530168 | -0.32502924 | 0.04400683 | 425 |
| PC.aa.C36.2 | 0.0067894 | -0.26480674 | 0.01802125 | -0.45613926 | -0.07347421 | 425 |
| PC.aa.C36.3 | 3.7284E-16 | 0.84609506 | 1.002E-14 | 0.64997065 | 1.04221946 | 430 |
| PC.aa.C36.4 | 2.2561E-19 | 0.99296538 | 1.6169E-17 | 0.78730171 | 1.19862906 | 395 |
| PC.aa.C36.5 | 0.28393175 | 0.12058696 | 0.40427368 | -0.10039142 | 0.34156534 | 367 |
| PC.aa.C36.6 | 1.2574E-06 | 0.52037661 | 6.1441E-06 | 0.31221953 | 0.72853369 | 454 |
| PC.aa.C38.0 | 0.59837295 | 0.05362524 | 0.71472325 | -0.14636262 | 0.25361311 | 397 |
| PC.aa.C38.1 | 0.9849656 | 0.00200629 | 0.98956824 | -0.20721803 | 0.21123061 | 369 |
| PC.aa.C38.2 | 1.7049E-07 | 0.53205608 | 9.3986E-07 | 0.33536966 | 0.7287425 | 430 |
| PC.aa.C38.3 | 4.87E-03 | 0.30571859 | 1.40E-02 | 0.09347655 | 0.51796063 | 381 |
| PC.aa.C38.4 | 0.09309386 | 0.17231427 | 0.16272503 | -0.02893557 | 0.3735641 | 381 |
| PC.aa.C38.5 | 0.93163989 | -0.00900284 | 0.97443443 | -0.21522538 | 0.1972197 | 381 |
| PC.aa.C38.6 | 1.595E-10 | 0.70760191 | 1.2701E-09 | 0.49607529 | 0.91912853 | 381 |
| PC.aa.C40.0 | 0.67411938 | 0.04672714 | 0.79199818 | -0.17163136 | 0.26508564 | 359 |
| PC.aa.C40.1 | 0.31157646 | 0.13476261 | 0.42941627 | -0.12698898 | 0.39651419 | 255 |
| PC.aa.C40.3 | 0.06042287 | 0.25385763 | 0.11918272 | -0.01120352 | 0.51891879 | 262 |
| PC.aa.C40.4 | 0.05038007 | 0.19793386 | 0.10123098 | -0.00032936 | 0.39619709 | 425 |
| PC.aa.C40.5 | 2.21E-02 | -0.24044791 | 4.90E-02 | -0.44621024 | -0.03468558 | 381 |
| PC.aa.C40.6 | 0.02995516 | 0.22148963 | 0.06571796 | 0.02160774 | 0.42137152 | 412 |
| PC.aa.C42.0 | 0.59407516 | 0.05726372 | 0.71355396 | -0.15386131 | 0.26838874 | 358 |
| PC.aa.C42.1 | 0.01692359 | 0.30106447 | 0.03954968 | 0.05441564 | 0.54771331 | 282 |
| PC.aa.C42.2 | 0.50115277 | 0.08194299 | 0.62644096 | -0.15754324 | 0.32142921 | 281 |
| PC.aa.C42.4 | 0.00259616 | 0.41110365 | 0.0078616 | 0.14496943 | 0.67723788 | 256 |
| PC.aa.C42.5 | 7.2053E-09 | 0.67317147 | 4.3031E-08 | 0.45036951 | 0.89597343 | 326 |
| PC.aa.C42.6 | 0.0015092 | 0.38695229 | 0.00506057 | 0.14913189 | 0.62477269 | 312 |
| PC.aa.C43.4 | 0.62989152 | 0.06192617 | 0.74410262 | -0.19076918 | 0.31462152 | 281 |
| PC.aa.C43.6 | 0.72146765 | -0.03631406 | 0.82949489 | -0.23640302 | 0.1637749 | 425 |
| PC.aa.C44.12 | 0.98137106 | 0.00234573 | 0.98956824 | -0.19500029 | 0.19969175 | 425 |
| PC.ae.C30.0 | 0.00221133 | 0.32120179 | 0.00689038 | 0.11623428 | 0.5261693 | 384 |
| PC.ae.C30.1 | 0.85601819 | -0.01965648 | 0.93899954 | -0.2325889 | 0.19327595 | 339 |
| PC.ae.C32.0 | 0.00383486 | 0.29292083 | 0.01129445 | 0.09485579 | 0.49098586 | 460 |
| PC.ae.C32.1 | 0.27875966 | 0.10500804 | 0.40223709 | -0.08528952 | 0.2953056 | 460 |
| PC.ae.C32.2 | 0.24243385 | 0.11431719 | 0.35700875 | -0.07762189 | 0.30625628 | 459 |
| PC.ae.C34.0 | 0.00252079 | 0.30490038 | 0.00774244 | 0.10768949 | 0.50211126 | 430 |
| PC.ae.C34.1 | 0.00199691 | 0.30457652 | 0.00631377 | 0.11207613 | 0.49707692 | 430 |
| PC.ae.C34.2 | 9.28E-01 | 0.00888595 | 9.74E-01 | -0.18557704 | 0.20334894 | 430 |
| PC.ae.C34.3 | 6.86E-01 | -0.0388682 | 7.98E-01 | -0.22792392 | 0.15018752 | 430 |
| PC.ae.C34.4 | 0.76078399 | -0.03101717 | 0.86088714 | -0.23117497 | 0.16914063 | 395 |
| PC.ae.C36.0 | 0.16316678 | -0.13706501 | 0.25238026 | -0.3299254 | 0.05579538 | 427 |
| PC.ae.C36.1 | 0.29153277 | 0.0988744 | 0.40967023 | -0.08515181 | 0.28290061 | 425 |
| PC.ae.C36.2 | 0.55465454 | 0.05916068 | 0.68534901 | -0.13751066 | 0.25583202 | 425 |
| PC.ae.C36.3 | 9.71E-01 | 0.00340971 | 0.98483328 | -0.18138851 | 0.18820793 | 460 |
| PC.ae.C36.4 | 0.92289842 | 0.0096937 | 0.97443443 | -0.18709504 | 0.20648244 | 395 |
| PC.ae.C36.5 | 0.25017281 | -0.11105842 | 0.36589901 | -0.30065369 | 0.07853686 | 395 |
| PC.ae.C36.6 | 0.00163268 | -0.3323651 | 0.00523919 | -0.53819619 | -0.126534 | 328 |
| PC.ae.C38.0 | 0.10042796 | 0.16908901 | 0.1727361 | -0.03278393 | 0.37096195 | 425 |
| PC.ae.C38.2 | 0.98977107 | 0.00126013 | 0.98977107 | -0.19182671 | 0.19434698 | 425 |
| PC.ae.C38.3 | 7.9295E-05 | 0.39136178 | 0.00030443 | 0.19835234 | 0.58437123 | 425 |
| PC.ae.C38.4 | 0.11315168 | 0.15999178 | 0.18430008 | -0.03811972 | 0.35810328 | 395 |
| PC.ae.C38.5 | 0.00901771 | -0.25431217 | 0.02254428 | -0.44482196 | -0.06380238 | 395 |
| PC.ae.C38.6 | 0.37675448 | -0.0878546 | 0.49826733 | -0.28301555 | 0.10730636 | 430 |
| PC.ae.C40.0 | 5.4256E-06 | 0.46442876 | 2.4819E-05 | 0.26626451 | 0.66259302 | 425 |
| PC.ae.C40.1 | 0.79624768 | 0.02603489 | 0.88701167 | -0.1720311 | 0.22410089 | 425 |
| PC.ae.C40.2 | 5.6641E-06 | 0.48226543 | 2.537E-05 | 0.27621665 | 0.68831421 | 397 |
| PC.ae.C40.3 | 0.09258156 | 0.17688714 | 0.16272503 | -0.02937286 | 0.38314714 | 397 |
| PC.ae.C40.4 | 0.68209045 | 0.04117452 | 0.79700787 | -0.15627251 | 0.23862154 | 425 |
| PC.ae.C40.5 | 0.03123393 | -0.21193171 | 0.06783125 | -0.40467366 | -0.01918976 | 425 |
| PC.ae.C40.6 | 0.72985542 | 0.03589601 | 0.83467508 | -0.16834667 | 0.24013869 | 381 |
| PC.ae.C42.0 | 1.6071E-14 | 0.84657407 | 3.8392E-13 | 0.63803952 | 1.05510862 | 396 |
| PC.ae.C42.1 | 0.03392906 | 0.22941487 | 0.07151714 | 0.01750823 | 0.44132151 | 357 |
| PC.ae.C42.2 | 0.03942273 | -0.21124631 | 0.08149892 | -0.41220764 | -0.01028498 | 397 |
| PC.ae.C42.3 | 9.69E-01 | 0.00401931 | 0.98483328 | -0.19773687 | 0.20577548 | 427 |
| PC.ae.C42.4 | 0.47752414 | -0.0723218 | 0.60750112 | -0.27228108 | 0.12763748 | 425 |
| PC.ae.C42.5 | 0.90863556 | 0.01170282 | 0.97443443 | -0.18865486 | 0.2120605 | 397 |
| PC.ae.C42.6 | 0.37775616 | -0.08559382 | 0.49826733 | -0.27611085 | 0.10492321 | 459 |
| SM.a.C30.1 | 0.14329377 | 0.14970268 | 0.22653059 | -0.05098483 | 0.35039018 | 392 |
| SM.a.C32.0 | 0.11000755 | 0.19533026 | 0.18054675 | -0.04452538 | 0.4351859 | 257 |
| SM.a.C32.1 | 0.06904539 | 0.17416492 | 0.12933315 | -0.01364728 | 0.36197713 | 425 |
| SM.a.C32.2 | 4.64E-01 | -0.07246321 | 5.94E-01 | -0.26685753 | 0.1219311 | 432 |
| SM.a.C33.1 | 0.17686876 | -0.13200513 | 0.26969349 | -0.32381796 | 0.05980771 | 425 |
| SM.a.C33.2 | 1.33E-02 | -0.27371323 | 3.21E-02 | -0.490007 | -0.05741946 | 364 |
| SM.a.C34.0 | 0.06216968 | 0.24292483 | 0.12010721 | -0.01243962 | 0.49828928 | 288 |
| SM.a.C34.1 | 0.5781177 | -0.05144257 | 0.70622332 | -0.23311334 | 0.1302282 | 432 |
| SM.a.C34.2 | 4.58E-02 | -0.18521545 | 9.31E-02 | -0.36698073 | -0.00345018 | 460 |
| SM.a.C34.3 | 0.56874383 | -0.06759535 | 0.69874242 | -0.3007997 | 0.16560899 | 282 |
| SM.a.C35.0 | 0.12038674 | -0.18211872 | 0.19315784 | -0.41219347 | 0.04795602 | 327 |
| SM.a.C35.1 | 0.00148438 | -0.31633427 | 0.00506057 | -0.5107194 | -0.12194914 | 425 |
| SM.a.C36.0 | 1.10E-05 | 0.52349765 | 4.85E-05 | 0.29331414 | 0.75368115 | 282 |
| SM.a.C36.1 | 0.86460299 | 0.01612633 | 0.94360225 | -0.16964585 | 0.20189852 | 432 |
| SM.a.C36.2 | 1.62E-03 | -0.30013612 | 5.24E-03 | -0.48612846 | -0.11414378 | 460 |
| SM.a.C36.3 | 4.59E-02 | -0.19809485 | 0.09305846 | -0.39254848 | -0.00364123 | 430 |
| SM.a.C37.1 | 0.93649389 | 0.00826593 | 0.97443443 | -0.195562 | 0.21209385 | 397 |
| SM.a.C38.1 | 1.7842E-13 | 0.70868783 | 2.5574E-12 | 0.52527735 | 0.89209831 | 458 |
| SM.a.C38.2 | 4.8766E-09 | 0.53671481 | 2.9956E-08 | 0.35992439 | 0.71350523 | 460 |
| SM.a.C39.1 | 0.36451958 | 0.08708403 | 0.4867808 | -0.10150444 | 0.27567251 | 397 |
| SM.a.C39.2 | 0.28188568 | -0.10230252 | 0.40403614 | -0.28892618 | 0.08432114 | 431 |
| SM.a.C40.1 | 0.00070114 | 0.3374036 | 0.00247124 | 0.14323575 | 0.53157145 | 394 |
| SM.a.C40.2 | 0.03273975 | 0.22805849 | 0.07039047 | 0.01882619 | 0.43729078 | 367 |
| SM.a.C40.3 | 2.2044E-16 | 0.97748121 | 6.7705E-15 | 0.75566446 | 1.19929797 | 323 |
| SM.a.C40.4 | 2.6181E-17 | 0.83747431 | 1.4072E-15 | 0.65067046 | 1.02427817 | 460 |
| SM.a.C40.5 | 0.08746144 | 0.17953447 | 0.15670175 | -0.02649063 | 0.38555956 | 401 |
| SM.a.C41.1 | 0.00265235 | 0.29168698 | 0.00792022 | 0.10204994 | 0.48132402 | 425 |
| SM.a.C41.2 | 0.34802358 | 0.08963036 | 0.46765669 | -0.09789701 | 0.27715772 | 425 |
| SM.a.C42.1 | 1.4483E-05 | 0.43086925 | 6.1058E-05 | 0.23785322 | 0.62388529 | 425 |
| SM.a.C42.2 | 5.7405E-08 | 0.53421762 | 3.2479E-07 | 0.3442008 | 0.72423444 | 425 |
| SM.a.C42.3 | 0.00152994 | 0.30331033 | 0.00506057 | 0.11640596 | 0.49021471 | 425 |
| SM.a.C42.4 | 0.1069006 | 0.16750305 | 0.18054675 | -0.0362831 | 0.37128919 | 395 |
| SM.a.C42.5 | 6.4221E-07 | 0.64158446 | 3.211E-06 | 0.39372244 | 0.88944648 | 282 |
| SM.a.C42.6 | 5.1518E-12 | 0.75553399 | 5.0347E-11 | 0.54694293 | 0.96412505 | 395 |
| SM.a.C43.1 | 0.85174981 | 0.01799847 | 0.93899954 | -0.17118683 | 0.20718378 | 425 |
| SM.a.C43.2 | 0.00778991 | 0.27396347 | 0.0199337 | 0.07256656 | 0.47536037 | 425 |
| SM.a.C44.2 | 0.30467741 | 0.11829817 | 0.42536132 | -0.10807551 | 0.34467186 | 326 |
| SM.a.C44.6 | 0.05570498 | 0.21181547 | 0.11089418 | -0.00518887 | 0.42881981 | 367 |
| SM.a.C31.1 | 0.76761145 | 0.02977384 | 0.86406524 | -0.16817863 | 0.2277263 | 397 |
| SM.a.C33.3 | 0.78377881 | 0.02592394 | 0.87766898 | -0.15965545 | 0.21150333 | 429 |
| SM.a.C35.2 | 2.357E-05 | -0.40875431 | 9.5615E-05 | -0.59668489 | -0.22082373 | 432 |
| SM.a.C37.3 | 5.89E-01 | -0.05314783 | 0.71355396 | -0.24650108 | 0.14020542 | 432 |
| SM.a.C39.5 | 0.948018 | -0.00674996 | 0.97443443 | -0.21023307 | 0.19673316 | 351 |
| SM.a.C41.0 | 0.00039186 | 0.4023508 | 0.00142798 | 0.18132773 | 0.62337387 | 351 |
| SM.a.C41.3 | 0.33699322 | 0.11336813 | 0.45856672 | -0.11859102 | 0.34532727 | 321 |
| SM.a.C43.0 | 0.00762973 | 0.30153275 | 0.01976376 | 0.08061257 | 0.52245293 | 312 |
| SM.a.C43.3 | 0.25839949 | 0.1212249 | 0.37537763 | -0.08937702 | 0.33182681 | 365 |
| Ala | 0.2139074 | -0.12562591 | 0.32160903 | -0.3239802 | 0.07272837 | 459 |
| Arg | 2.2128E-06 | -0.41941842 | 1.0572E-05 | -0.59133182 | -0.24750501 | 458 |
| Asn | 0.01810903 | 0.24697814 | 0.04186496 | 0.04236513 | 0.45159115 | 435 |
| Asp | 0.00788077 | 0.27061857 | 0.0199337 | 0.07135713 | 0.46988001 | 459 |
| Cit | 1.1142E-10 | -0.58656999 | 9.2135E-10 | -0.76110923 | -0.41203075 | 459 |
| Gln | 3.4176E-10 | -0.58207105 | 2.6242E-09 | -0.76003827 | -0.40410383 | 435 |
| Glu | 1.10E-01 | -0.14739377 | 1.81E-01 | -0.32828148 | 0.03349394 | 459 |
| Gly | 9.7984E-21 | -0.94181783 | 1.0533E-18 | -1.13051047 | -0.75312519 | 459 |
| His | 3.521E-06 | 0.48000191 | 1.6457E-05 | 0.27914331 | 0.6808605 | 453 |
| Ile | 9.75E-03 | -0.27244294 | 0.0240871 | -0.47858247 | -0.06630342 | 327 |
| Leu | 7.42E-03 | -0.21546191 | 1.94E-02 | -0.37287521 | -0.05804861 | 435 |
| Lys | 0.15599938 | -0.13601802 | 0.24480898 | -0.32413505 | 0.05209901 | 435 |
| Met | 0.01866668 | -0.20943614 | 0.04269507 | -0.38378287 | -0.03508941 | 435 |
| Orn | 2.1575E-14 | -0.72744147 | 4.6386E-13 | -0.90816607 | -0.54671687 | 435 |
| Phe | 1.98E-02 | -0.21539626 | 0.04484496 | -0.39643121 | -0.03436131 | 435 |
| Pro | 1.0893E-10 | -0.54846583 | 9.2135E-10 | -0.71157622 | -0.38535544 | 459 |
| Trp | 0.92021203 | 0.00854758 | 0.97443443 | -0.15905553 | 0.1761507 | 459 |
| Ser | 2.3042E-07 | -0.5349294 | 1.2385E-06 | -0.73506005 | -0.33479874 | 459 |
| Thr | 0.01429038 | 0.27423388 | 0.03406147 | 0.05511756 | 0.4933502 | 431 |
| Tyr | 2.56E-09 | -0.60172185 | 1.72E-08 | -0.79606028 | -0.40738342 | 435 |
| Val | 1.10E-01 | -0.1395368 | 1.81E-01 | -0.31055566 | 0.03148207 | 435 |
| NEFA_12_0 | 0.32190329 | 0.10796853 | 0.44082298 | -0.10609802 | 0.32203509 | 356 |
| NEFA_14_0 | 0.06247953 | 0.17948683 | 0.12010721 | -0.00939433 | 0.368368 | 441 |
| NEFA_14_1 | 0.20704474 | 0.12884212 | 0.31348323 | -0.07154959 | 0.32923383 | 455 |
| NEFA_15_0 | 0.22491911 | 0.11054057 | 0.33581673 | -0.06822568 | 0.28930682 | 452 |
| NEFA_15_1 | 0.10562747 | 0.19413247 | 0.18023735 | -0.04124274 | 0.42950769 | 308 |
| NEFA_16_0 | 0.16807064 | 0.14610507 | 0.25810848 | -0.06190731 | 0.35411745 | 380 |
| NEFA_16_1 | 0.07398219 | 0.18518877 | 0.13712216 | -0.01802899 | 0.38840652 | 455 |
| NEFA_17_0 | 0.89833755 | -0.0117474 | 0.97443443 | -0.19238427 | 0.16888947 | 412 |
| NEFA_17_1 | 0.61807872 | 0.05109236 | 0.7341819 | -0.15020964 | 0.25239437 | 404 |
| NEFA_18_0 | 0.43062291 | -0.07659655 | 0.55773449 | -0.26748994 | 0.11429685 | 380 |
| NEFA_18_1 | 0.48094369 | 0.07138609 | 0.60815663 | -0.12753918 | 0.27031136 | 414 |
| NEFA_18_2 | 0.10853032 | 0.17421316 | 0.18054675 | -0.03873083 | 0.38715716 | 379 |
| NEFA_18_3 | 0.06710034 | 0.18103728 | 0.1276688 | -0.0128035 | 0.37487806 | 455 |
| NEFA_20_1 | 0.06256748 | 0.19084905 | 0.12010721 | -0.01006158 | 0.39175969 | 396 |
| NEFA_20_2 | 0.00503559 | 0.30560415 | 0.01424543 | 0.09261715 | 0.51859114 | 381 |
| NEFA_20_3 | 0.00069211 | 0.37605395 | 0.00247124 | 0.15991654 | 0.59219136 | 379 |
| NEFA_20_4 | 0.02103224 | 0.24315966 | 0.04710345 | 0.03681831 | 0.449501 | 379 |
| NEFA_22_6 | 0.00130334 | 0.33375779 | 0.00451965 | 0.13110954 | 0.53640604 | 414 |
| NEFA_24_1 | 0.59287208 | -0.04685965 | 0.71355396 | -0.21897114 | 0.12525183 | 455 |
| NEFA_12_1 | 0.69284312 | 0.04178228 | 0.80086705 | -0.16601069 | 0.24957525 | 409 |
| NEFA_13_1 | 0.92733806 | 0.01242856 | 0.97443443 | -0.25578731 | 0.28064443 | 237 |
| NEFA_14_2 | 0.11633447 | 0.17811014 | 0.18805948 | -0.04441047 | 0.40063076 | 352 |
| NEFA_16_2 | 0.09994012 | 0.1842588 | 0.1727361 | -0.03542051 | 0.4039381 | 393 |
| NEFA_18_4 | 0.81689142 | -0.02763587 | 0.90531781 | -0.26228791 | 0.20701616 | 311 |
| NEFA_19_0 | 0.93886035 | -0.00792797 | 0.97443443 | -0.21108372 | 0.19522778 | 343 |
| NEFA_19_1 | 0.48369666 | 0.07342064 | 0.60815663 | -0.13249816 | 0.27933945 | 384 |
| NEFA_20_5 | 3.83E-01 | -0.08934692 | 5.02E-01 | -0.29056693 | 0.11187308 | 384 |
| NEFA_22_4 | 0.0691782 | 0.21132179 | 0.12933315 | -0.01668582 | 0.4393294 | 343 |
| NEFA_22_5 | 0.94469198 | -0.00739849 | 0.97443443 | -0.21694972 | 0.20215274 | 384 |
| NEFA_24_4 | 1.286E-05 | 0.69670479 | 5.5297E-05 | 0.38877924 | 1.00463034 | 237 |
| NEFA_24_5 | 2.0579E-13 | 1.04529056 | 2.7653E-12 | 0.77963771 | 1.31094341 | 265 |
| NEFA_26_1 | 0.03821412 | -0.2010012 | 0.07976734 | -0.39101363 | -0.01098877 | 313 |

## Supplemental Table 4: Results of the Regression model with Metabolite concentration as outcome and two level sex variable as predictor (*HC* females versus males), adjusted for ethnicity. ethnicity P-values, beta coefficient, Confidence interval (CI), number of observations per metabolite and FDR corrected p-values reported for all metabolites.

| Analytes | P-Value | BETA | FDR P-Value | CI lower | CI upper | Number of observations |
| --- | --- | --- | --- | --- | --- | --- |
| Carn | 1.7197E-56 | 1.11821164 | 1.2324E-54 | 0.99037235 | 1.24605092 | 803 |
| Carn.a.C10.0 | 0.21466905 | 0.11928261 | 0.23790642 | -0.06934927 | 0.30791449 | 502 |
| Carn.a.C10.1 | 0.05654552 | 0.17954105 | 0.06607221 | -0.00502403 | 0.36410614 | 505 |
| Carn.a.C12.0 | 9.1134E-05 | 0.38031595 | 0.00014261 | 0.19092678 | 0.56970512 | 499 |
| Carn.a.C12.1 | 0.01542059 | 0.25198316 | 0.0189453 | 0.04833281 | 0.4556335 | 446 |
| Carn.a.C14.0 | 8.1043E-08 | 0.44132664 | 1.6134E-07 | 0.28160933 | 0.60104396 | 666 |
| Carn.a.C14.1 | 0.08344544 | 0.13692624 | 0.09593995 | -0.01815385 | 0.29200633 | 725 |
| Carn.a.C14.2 | 6.4182E-07 | 0.39603272 | 1.1499E-06 | 0.24125095 | 0.55081448 | 719 |
| Carn.a.C16.0 | 6.126E-23 | 0.72337079 | 2.9934E-22 | 0.58379499 | 0.86294659 | 800 |
| Carn.a.C16.1 | 0.0045534 | 0.21587838 | 0.00600602 | 0.066957 | 0.36479976 | 724 |
| Carn.a.C18.0 | 8.1421E-25 | 0.8430312 | 4.6067E-24 | 0.68875965 | 0.99730275 | 643 |
| Carn.a.C18.1 | 4.0508E-20 | 0.6701725 | 1.6128E-19 | 0.53078542 | 0.80955958 | 802 |
| Carn.a.C2.0 | 0.00033662 | 0.27075073 | 0.00050611 | 0.12319052 | 0.41831094 | 748 |
| Carn.a.C3.0 | 1.84E-33 | 0.88290796 | 1.89E-32 | 0.74567122 | 1.0201447 | 801 |
| Carn.a.C4.0 | 0.00186733 | 0.260081 | 0.00259017 | 0.09654976 | 0.42361223 | 695 |
| Carn.a.C4.0.DC | 6.4862E-10 | 0.62663076 | 1.4679E-09 | 0.43171973 | 0.82154178 | 442 |
| Carn.a.C5.0 | 2.1899E-18 | 0.80301093 | 7.98E-18 | 0.62913156 | 0.9768903 | 541 |
| Carn.a.C6.0 | 0.01300024 | 0.20249261 | 0.01634533 | 0.04284044 | 0.36214478 | 683 |
| Carn.a.C8.1 | 0.30174474 | 0.08725418 | 0.32765212 | -0.07853969 | 0.25304805 | 597 |
| lyso.PC.a.C14.0 | 0.53365424 | 0.04690767 | 0.56520031 | -0.10095775 | 0.19477309 | 801 |
| lyso.PC.a.C16.0 | 1.8898E-25 | 0.7925615 | 1.1286E-24 | 0.64878511 | 0.93633789 | 748 |
| lyso.PC.a.C16.1 | 2.0844E-09 | 0.44773272 | 4.6681E-09 | 0.30272512 | 0.59274033 | 803 |
| lyso.PC.a.C18.0 | 2.6918E-48 | 1.05676389 | 8.2677E-47 | 0.924113 | 1.18941478 | 803 |
| lyso.PC.a.C18.1 | 8.82E-49 | 1.10336405 | 3.16E-47 | 0.96638248 | 1.24034561 | 748 |
| lyso.PC.a.C18.2 | 8.76E-46 | 1.07302326 | 2.09E-44 | 0.93468152 | 1.211365 | 748 |
| lyso.PC.a.C18.3 | 1.2737E-20 | 0.79315097 | 5.3695E-20 | 0.63155518 | 0.95474676 | 644 |
| lyso.PC.a.C18.6 | 8.7589E-12 | 0.59889707 | 2.1159E-11 | 0.4301262 | 0.76766793 | 576 |
| lyso.PC.a.C20.2 | 1.7126E-08 | 0.51528173 | 3.5747E-08 | 0.33859264 | 0.69197082 | 521 |
| lyso.PC.a.C20.3 | 1.7041E-43 | 1.06794706 | 3.3307E-42 | 0.9268294 | 1.20906472 | 688 |
| lyso.PC.a.C20.4 | 1.1525E-60 | 1.17094149 | 1.2389E-58 | 1.04272039 | 1.2991626 | 803 |
| lyso.PC.a.C20.5 | 4.1755E-42 | 1.03083341 | 7.4811E-41 | 0.89127971 | 1.17038712 | 740 |
| lyso.PC.a.C22.5 | 1.7227E-61 | 1.24327595 | 3.7037E-59 | 1.10994708 | 1.37660482 | 704 |
| lyso.PC.a.C22.6 | 8.1557E-23 | 0.74079789 | 3.8966E-22 | 0.59765256 | 0.88394322 | 758 |
| lyso.PC.e.C16.0 | 3.6712E-14 | 0.56493189 | 1.1439E-13 | 0.42114639 | 0.70871739 | 803 |
| lyso.PC.e.C18.0 | 1.7671E-33 | 0.90067921 | 1.8883E-32 | 0.76089806 | 1.04046036 | 780 |
| lyso.PC.e.C18.1 | 9.2416E-30 | 1.00417967 | 7.6421E-29 | 0.83984436 | 1.16851498 | 576 |
| PC.aa.C30.0 | 1.0235E-21 | -0.7202644 | 4.7839E-21 | -0.86364394 | -0.57688485 | 797 |
| PC.aa.C30.1 | 3.3291E-12 | -0.61276178 | 8.4208E-12 | -0.78182159 | -0.44370197 | 565 |
| PC.aa.C30.2 | 3.5451E-08 | -0.5239015 | 7.1905E-08 | -0.70776786 | -0.34003514 | 514 |
| PC.aa.C32.0 | 1.9557E-29 | -0.83026535 | 1.5017E-28 | -0.96919679 | -0.6913339 | 803 |
| PC.aa.C32.1 | 2.1224E-18 | -0.70451337 | 7.8677E-18 | -0.85822414 | -0.55080259 | 703 |
| PC.aa.C32.2 | 1.6383E-37 | -1.01169964 | 2.3482E-36 | -1.15755476 | -0.86584452 | 681 |
| PC.aa.C32.3 | 2.2648E-10 | -0.49644644 | 5.2926E-10 | -0.64787335 | -0.34501953 | 703 |
| PC.aa.C34.0 | 0.01590565 | -0.21605409 | 0.0194302 | -0.39153305 | -0.04057512 | 575 |
| PC.aa.C34.1 | 3.8235E-15 | -0.60220211 | 1.2461E-14 | -0.74947995 | -0.45492427 | 758 |
| PC.aa.C34.2 | 1.1461E-36 | -0.95619066 | 1.5401E-35 | -1.09666056 | -0.81572077 | 748 |
| PC.aa.C34.3 | 7.0364E-24 | -0.77961525 | 3.6898E-23 | -0.92631081 | -0.63291969 | 748 |
| PC.aa.C34.4 | 4.4771E-21 | -0.75746381 | 1.9644E-20 | -0.91029741 | -0.60463022 | 704 |
| PC.aa.C34.5 | 0.35616517 | 0.07316526 | 0.38480157 | -0.08241447 | 0.22874499 | 711 |
| PC.aa.C36.0 | 0.21691758 | -0.09221918 | 0.23916554 | -0.23871429 | 0.05427594 | 747 |
| PC.aa.C36.1 | 0.82264253 | -0.01731509 | 0.84625907 | -0.1689128 | 0.13428263 | 747 |
| PC.aa.C36.2 | 0.03174219 | -0.16387333 | 0.03834029 | -0.31338736 | -0.01435931 | 748 |
| PC.aa.C36.3 | 5.9268E-33 | -0.90558126 | 5.6074E-32 | -1.0472307 | -0.76393181 | 759 |
| PC.aa.C36.4 | 2.4338E-27 | -0.84920743 | 1.6352E-26 | -0.9966834 | -0.70173146 | 704 |
| PC.aa.C36.5 | 0.55279374 | -0.04871113 | 0.58260125 | -0.20976676 | 0.1123445 | 659 |
| PC.aa.C36.6 | 4.5511E-17 | -0.6325701 | 1.6041E-16 | -0.77710226 | -0.48803794 | 794 |
| PC.aa.C38.0 | 8.1425E-06 | -0.34766804 | 1.3466E-05 | -0.49952616 | -0.19580992 | 702 |
| PC.aa.C38.1 | 0.00318356 | -0.23907648 | 0.00430482 | -0.39764571 | -0.08050724 | 642 |
| PC.aa.C38.2 | 1.1808E-25 | -0.78751093 | 7.2536E-25 | -0.92977192 | -0.64524994 | 756 |
| PC.aa.C38.3 | 2.30E-07 | -0.41953622 | 4.26E-07 | -0.57713455 | -0.26193788 | 681 |
| PC.aa.C38.4 | 0.00023901 | -0.29629555 | 0.00036188 | -0.45380719 | -0.13878391 | 681 |
| PC.aa.C38.5 | 0.50533866 | -0.05368613 | 0.53786046 | -0.21184981 | 0.10447756 | 681 |
| PC.aa.C38.6 | 1.1157E-32 | -0.94266512 | 9.9952E-32 | -1.09002852 | -0.79530171 | 681 |
| PC.aa.C40.0 | 1.1082E-06 | -0.40641499 | 1.9215E-06 | -0.56866218 | -0.2441678 | 643 |
| PC.aa.C40.1 | 0.0009838 | -0.33182419 | 0.00139156 | -0.52843884 | -0.13520954 | 461 |
| PC.aa.C40.3 | 6.7535E-05 | -0.39229636 | 0.00010676 | -0.58400871 | -0.200584 | 471 |
| PC.aa.C40.4 | 0.09184418 | -0.13219182 | 0.10447883 | -0.28593847 | 0.02155484 | 748 |
| PC.aa.C40.5 | 4.54E-02 | 0.16271897 | 5.37E-02 | 0.00331571 | 0.32212222 | 681 |
| PC.aa.C40.6 | 3.0511E-13 | -0.56733791 | 8.9862E-13 | -0.71724991 | -0.41742592 | 732 |
| PC.aa.C42.0 | 4.4152E-10 | -0.5207031 | 1.0207E-09 | -0.68205103 | -0.35935517 | 643 |
| PC.aa.C42.1 | 9.7797E-09 | -0.53835006 | 2.0818E-08 | -0.71978135 | -0.35691877 | 523 |
| PC.aa.C42.2 | 0.00409303 | -0.27532258 | 0.00543211 | -0.46287509 | -0.08777007 | 508 |
| PC.aa.C42.4 | 7.0965E-09 | -0.58049196 | 1.5569E-08 | -0.77369829 | -0.38728562 | 439 |
| PC.aa.C42.5 | 1.4436E-29 | -0.97424536 | 1.1495E-28 | -1.13449773 | -0.81399299 | 589 |
| PC.aa.C42.6 | 2.7819E-12 | -0.63105866 | 7.294E-12 | -0.80451627 | -0.45760105 | 566 |
| PC.aa.C43.4 | 3.6655E-05 | -0.39635791 | 6.0159E-05 | -0.58332972 | -0.20938611 | 506 |
| PC.aa.C43.6 | 0.0004252 | -0.27102689 | 0.00063009 | -0.42133437 | -0.12071941 | 748 |
| PC.aa.C44.12 | 0.00234146 | -0.23365663 | 0.00320646 | -0.38387297 | -0.0834403 | 748 |
| PC.ae.C30.0 | 7.5441E-07 | -0.41100615 | 1.3405E-06 | -0.57261285 | -0.24939944 | 682 |
| PC.ae.C30.1 | 0.00147485 | -0.26858844 | 0.0020725 | -0.43372678 | -0.10345009 | 614 |
| PC.ae.C32.0 | 3.4232E-09 | -0.44208454 | 7.5875E-09 | -0.5872741 | -0.29689497 | 803 |
| PC.ae.C32.1 | 1.0631E-07 | -0.38955734 | 2.0969E-07 | -0.53209882 | -0.24701585 | 803 |
| PC.ae.C32.2 | 9.6015E-13 | -0.51335249 | 2.6466E-12 | -0.65227513 | -0.37442985 | 803 |
| PC.ae.C34.0 | 0.00014367 | -0.2932575 | 0.00022223 | -0.44391212 | -0.14260287 | 758 |
| PC.ae.C34.1 | 4.4513E-13 | -0.55016065 | 1.276E-12 | -0.69668458 | -0.40363672 | 759 |
| PC.ae.C34.2 | 8.75E-09 | -0.42002531 | 1.88E-08 | -0.56172969 | -0.27832093 | 759 |
| PC.ae.C34.3 | 1.94E-01 | -0.09605148 | 2.16E-01 | -0.24105956 | 0.0489566 | 759 |
| PC.ae.C34.4 | 0.90571099 | 0.00918878 | 0.91852766 | -0.14306409 | 0.16144166 | 702 |
| PC.ae.C36.0 | 0.99313774 | -0.00065797 | 0.99313774 | -0.15079198 | 0.14947605 | 748 |
| PC.ae.C36.1 | 0.97972539 | -0.00195603 | 0.98430355 | -0.15300738 | 0.14909532 | 748 |
| PC.ae.C36.2 | 0.00080065 | -0.25981699 | 0.0011553 | -0.41133252 | -0.10830147 | 748 |
| PC.ae.C36.3 | 8.28E-04 | -0.24151727 | 0.00118619 | -0.38277416 | -0.10026038 | 803 |
| PC.ae.C36.4 | 0.05342619 | 0.15042534 | 0.06276848 | -0.00222599 | 0.30307666 | 704 |
| PC.ae.C36.5 | 3.6534E-06 | 0.35513532 | 6.2339E-06 | 0.2057499 | 0.50452073 | 704 |
| PC.ae.C36.6 | 1.0658E-08 | 0.48235753 | 2.2465E-08 | 0.31908277 | 0.6456323 | 597 |
| PC.ae.C38.0 | 1.7668E-08 | -0.42669797 | 3.6526E-08 | -0.57376452 | -0.27963143 | 748 |
| PC.ae.C38.2 | 7.6235E-06 | -0.34430475 | 1.2706E-05 | -0.49426742 | -0.19434207 | 748 |
| PC.ae.C38.3 | 6.0603E-10 | -0.4765883 | 1.3861E-09 | -0.62577585 | -0.32740074 | 748 |
| PC.ae.C38.4 | 0.25701839 | -0.0879311 | 0.28050231 | -0.24011838 | 0.06425619 | 704 |
| PC.ae.C38.5 | 0.00019393 | 0.29146024 | 0.00029575 | 0.13872792 | 0.44419256 | 704 |
| PC.ae.C38.6 | 0.6140228 | -0.03733831 | 0.64084904 | -0.18261507 | 0.10793844 | 758 |
| PC.ae.C40.0 | 3.081E-25 | -0.79490377 | 1.7903E-24 | -0.93980022 | -0.65000731 | 748 |
| PC.ae.C40.1 | 0.15091741 | -0.10923547 | 0.16899606 | -0.25838662 | 0.03991567 | 748 |
| PC.ae.C40.2 | 1.6401E-24 | -0.80161844 | 8.8157E-24 | -0.94987012 | -0.65336676 | 702 |
| PC.ae.C40.3 | 3.512E-13 | -0.55956756 | 1.0204E-12 | -0.70772148 | -0.41141364 | 703 |
| PC.ae.C40.4 | 0.00084365 | -0.2505344 | 0.00120122 | -0.39727649 | -0.10379232 | 748 |
| PC.ae.C40.5 | 0.44244222 | 0.0581532 | 0.47562538 | -0.09040344 | 0.20670985 | 748 |
| PC.ae.C40.6 | 0.00042787 | -0.2847924 | 0.00063009 | -0.44276403 | -0.12682077 | 681 |
| PC.ae.C42.0 | 7.3311E-45 | -1.09297986 | 1.5762E-43 | -1.23498368 | -0.95097604 | 703 |
| PC.ae.C42.1 | 0.00363294 | -0.25102883 | 0.00485144 | -0.41989013 | -0.08216753 | 640 |
| PC.ae.C42.2 | 0.59638746 | -0.04112992 | 0.62547953 | -0.19353825 | 0.11127841 | 703 |
| PC.ae.C42.3 | 9.25E-12 | -0.50556097 | 2.2101E-11 | -0.64883979 | -0.36228216 | 754 |
| PC.ae.C42.4 | 8.0832E-07 | -0.37733684 | 1.4245E-06 | -0.52621717 | -0.22845651 | 748 |
| PC.ae.C42.5 | 1.9652E-07 | -0.40819159 | 3.7392E-07 | -0.56069313 | -0.25569005 | 703 |
| PC.ae.C42.6 | 4.4964E-05 | -0.30274643 | 7.2687E-05 | -0.44758446 | -0.15790839 | 802 |
| SM.a.C30.1 | 3.6847E-19 | -0.69701321 | 1.4146E-18 | -0.84552417 | -0.54850225 | 694 |
| SM.a.C32.0 | 2.0517E-07 | -0.51783762 | 3.8695E-07 | -0.71071545 | -0.32495979 | 454 |
| SM.a.C32.1 | 1.5516E-12 | -0.54128247 | 4.2226E-12 | -0.68902185 | -0.39354308 | 748 |
| SM.a.C32.2 | 7.83E-52 | -1.00856617 | 4.21E-50 | -1.12939425 | -0.88773809 | 755 |
| SM.a.C33.1 | 0.00562113 | -0.2093568 | 0.00728039 | -0.35734865 | -0.06136494 | 748 |
| SM.a.C33.2 | 3.06E-12 | -0.53264705 | 7.83E-12 | -0.67973244 | -0.38556166 | 650 |
| SM.a.C34.0 | 1.0094E-13 | -0.63602275 | 3.014E-13 | -0.79936048 | -0.47268503 | 513 |
| SM.a.C34.1 | 8.0112E-09 | -0.4391508 | 1.7398E-08 | -0.58691451 | -0.29138709 | 758 |
| SM.a.C34.2 | 6.00E-33 | -0.80314363 | 5.61E-32 | -0.92911017 | -0.67717708 | 803 |
| SM.a.C34.3 | 2.958E-12 | -0.62337188 | 7.6622E-12 | -0.79465003 | -0.45209373 | 523 |
| SM.a.C35.0 | 0.00203161 | -0.2655004 | 0.00279997 | -0.43374399 | -0.09725682 | 598 |
| SM.a.C35.1 | 0.45410072 | -0.05625062 | 0.48572962 | -0.20368764 | 0.0911864 | 748 |
| SM.a.C36.0 | 3.83E-15 | -0.73688537 | 1.25E-14 | -0.91551779 | -0.55825295 | 523 |
| SM.a.C36.1 | 1.7307E-19 | -0.66260572 | 6.7653E-19 | -0.80274996 | -0.52246148 | 758 |
| SM.a.C36.2 | 2.06E-23 | -0.66808788 | 1.03E-22 | -0.79551776 | -0.540658 | 803 |
| SM.a.C36.3 | 7.19E-16 | -0.57535461 | 2.4168E-15 | -0.71231144 | -0.43839779 | 758 |
| SM.a.C37.1 | 5.8885E-11 | -0.50300114 | 1.3912E-10 | -0.65149768 | -0.35450459 | 703 |
| SM.a.C38.1 | 8.0511E-34 | -0.88421957 | 9.1104E-33 | -1.02080733 | -0.7476318 | 800 |
| SM.a.C38.2 | 6.1698E-50 | -1.0560113 | 2.653E-48 | -1.18597172 | -0.92605088 | 803 |
| SM.a.C39.1 | 2.6427E-07 | -0.40802163 | 4.8562E-07 | -0.56212912 | -0.25391414 | 703 |
| SM.a.C39.2 | 3.8101E-12 | -0.51723149 | 9.5253E-12 | -0.66106904 | -0.37339395 | 755 |
| SM.a.C40.1 | 1.6152E-07 | -0.42353559 | 3.1285E-07 | -0.58063558 | -0.26643561 | 695 |
| SM.a.C40.2 | 1.082E-23 | -0.79565068 | 5.539E-23 | -0.94536113 | -0.64594024 | 659 |
| SM.a.C40.3 | 2.6844E-28 | -0.97708766 | 1.9901E-27 | -1.14178918 | -0.81238614 | 574 |
| SM.a.C40.4 | 1.3063E-24 | -0.75517977 | 7.2017E-24 | -0.89516711 | -0.61519244 | 802 |
| SM.a.C40.5 | 0.04243758 | -0.1609679 | 0.05097251 | -0.31642817 | -0.00550762 | 712 |
| SM.a.C41.1 | 5.9602E-07 | -0.39188312 | 1.0768E-06 | -0.54464046 | -0.23912578 | 748 |
| SM.a.C41.2 | 2.6503E-21 | -0.69942644 | 1.1871E-20 | -0.83993926 | -0.55891362 | 748 |
| SM.a.C42.1 | 3.768E-05 | -0.32322233 | 6.1372E-05 | -0.4762598 | -0.17018486 | 748 |
| SM.a.C42.2 | 9.0653E-28 | -0.83255676 | 6.4968E-27 | -0.97620519 | -0.68890833 | 748 |
| SM.a.C42.3 | 1.024E-39 | -0.96768137 | 1.6935E-38 | -1.10335472 | -0.83200802 | 748 |
| SM.a.C42.4 | 2.2779E-08 | -0.44034735 | 4.6643E-08 | -0.59324837 | -0.28744633 | 704 |
| SM.a.C42.5 | 4.5113E-19 | -0.82937905 | 1.7016E-18 | -1.00493237 | -0.65382574 | 523 |
| SM.a.C42.6 | 1.6839E-35 | -0.97487981 | 2.0114E-34 | -1.12033807 | -0.82942154 | 704 |
| SM.a.C43.1 | 0.11435646 | -0.12244128 | 0.12940336 | -0.27450131 | 0.02961875 | 748 |
| SM.a.C43.2 | 1.3515E-07 | -0.40270678 | 2.6415E-07 | -0.551225 | -0.25418855 | 748 |
| SM.a.C44.2 | 0.00482521 | -0.24428212 | 0.00629857 | -0.41385825 | -0.07470599 | 590 |
| SM.a.C44.6 | 5.8063E-12 | -0.55615156 | 1.4349E-11 | -0.71185889 | -0.40044422 | 658 |
| SM.a.C31.1 | 6.0192E-12 | -0.53234549 | 1.4706E-11 | -0.681661 | -0.38302997 | 702 |
| SM.a.C33.3 | 0.00483379 | -0.21836452 | 0.00629857 | -0.37003806 | -0.06669097 | 754 |
| SM.a.C35.2 | 0.00768958 | -0.19547306 | 0.00984084 | -0.33905569 | -0.05189044 | 758 |
| SM.a.C37.3 | 2.51E-03 | -0.22726257 | 0.00341471 | -0.3743883 | -0.08013683 | 757 |
| SM.a.C39.5 | 0.00048953 | 0.28764314 | 0.00071598 | 0.12647944 | 0.44880683 | 625 |
| SM.a.C41.0 | 5.7211E-13 | -0.59878733 | 1.6185E-12 | -0.75853162 | -0.43904304 | 634 |
| SM.a.C41.3 | 2.2123E-14 | -0.6428205 | 7.0993E-14 | -0.80393429 | -0.48170671 | 586 |
| SM.a.C43.0 | 7.1606E-16 | -0.70400492 | 2.4168E-15 | -0.87040442 | -0.53760543 | 567 |
| SM.a.C43.3 | 5.1423E-14 | -0.60434399 | 1.5794E-13 | -0.75847755 | -0.45021043 | 650 |
| Ala | 0.06673713 | 0.13431169 | 0.07714238 | -0.00929035 | 0.27791374 | 800 |
| Arg | 2.3014E-16 | 0.60628679 | 7.9805E-16 | 0.46434977 | 0.74822382 | 798 |
| Asn | 0.64648637 | -0.0351624 | 0.67147135 | -0.18560982 | 0.11528502 | 736 |
| Asp | 0.04413234 | -0.14876988 | 0.05242239 | -0.29362271 | -0.00391705 | 800 |
| Cit | 2.2069E-26 | 0.77693074 | 1.3955E-25 | 0.63853484 | 0.91532663 | 800 |
| Gln | 6.2421E-32 | 0.88141753 | 5.3682E-31 | 0.74121491 | 1.02162014 | 736 |
| Glu | 1.93E-12 | 0.50411869 | 5.18E-12 | 0.36577434 | 0.64246303 | 800 |
| Gly | 2.248E-21 | 0.64067093 | 1.0283E-20 | 0.51196102 | 0.76938085 | 800 |
| His | 0.01969992 | -0.17733271 | 0.02392929 | -0.32629789 | -0.02836753 | 789 |
| Ile | 1.75E-20 | 0.79095177 | 7.1844E-20 | 0.62979976 | 0.95210378 | 577 |
| Leu | 3.97E-47 | 1.06545948 | 1.07E-45 | 0.93059428 | 1.20032468 | 736 |
| Lys | 2.1362E-07 | 0.39528675 | 3.9938E-07 | 0.24710067 | 0.54347282 | 736 |
| Met | 1.0866E-20 | 0.71410333 | 4.6726E-20 | 0.56828756 | 0.8599191 | 736 |
| Orn | 1.4182E-38 | 0.9378877 | 2.1779E-37 | 0.80425274 | 1.07152266 | 736 |
| Phe | 3.00E-17 | 0.64665659 | 1.0735E-16 | 0.50005359 | 0.79325958 | 736 |
| Pro | 1.2505E-35 | 0.88885076 | 1.5815E-34 | 0.75561524 | 1.02208628 | 800 |
| Trp | 2.5064E-12 | 0.52197152 | 6.6529E-12 | 0.37795257 | 0.66599048 | 800 |
| Ser | 0.68724488 | -0.02746728 | 0.71037332 | -0.16134097 | 0.1064064 | 800 |
| Thr | 9.8726E-07 | -0.36281366 | 1.7257E-06 | -0.50713791 | -0.21848941 | 755 |
| Tyr | 1.77E-20 | 0.70372633 | 7.18E-20 | 0.55917638 | 0.84827628 | 736 |
| Val | 5.58E-27 | 0.81181427 | 3.64E-26 | 0.66949194 | 0.95413661 | 736 |
| NEFA_12_0 | 0.00151499 | -0.26299656 | 0.00211508 | -0.42511229 | -0.10088084 | 637 |
| NEFA_14_0 | 9.1534E-05 | -0.30080598 | 0.00014261 | -0.45095749 | -0.15065446 | 780 |
| NEFA_14_1 | 9.5859E-13 | -0.52860527 | 2.6466E-12 | -0.67163487 | -0.38557566 | 797 |
| NEFA_15_0 | 0.0432231 | -0.15305257 | 0.05162759 | -0.30143109 | -0.00467404 | 795 |
| NEFA_15_1 | 0.00019396 | -0.35293253 | 0.00029575 | -0.53768099 | -0.16818406 | 543 |
| NEFA_16_0 | 0.01334658 | -0.20050326 | 0.01658679 | -0.35918998 | -0.04181654 | 689 |
| NEFA_16_1 | 3.4903E-14 | -0.55525313 | 1.1036E-13 | -0.69643033 | -0.41407592 | 797 |
| NEFA_17_0 | 0.11591144 | 0.12364807 | 0.13047622 | -0.0305708 | 0.27786694 | 743 |
| NEFA_17_1 | 0.00675345 | -0.21406449 | 0.00869456 | -0.36876793 | -0.05936104 | 732 |
| NEFA_18_0 | 0.23332995 | 0.09607024 | 0.25594867 | -0.06205941 | 0.25419989 | 680 |
| NEFA_18_1 | 0.00065049 | -0.26338283 | 0.00094497 | -0.41438633 | -0.11237933 | 746 |
| NEFA_18_2 | 4.9032E-05 | -0.32953459 | 7.8088E-05 | -0.48787831 | -0.17119087 | 691 |
| NEFA_18_3 | 5.9862E-06 | -0.3387941 | 1.0134E-05 | -0.48470866 | -0.19287955 | 796 |
| NEFA_20_1 | 0.0033917 | -0.23556539 | 0.00455759 | -0.39289105 | -0.07823972 | 719 |
| NEFA_20_2 | 2.7308E-06 | -0.37874033 | 4.6969E-06 | -0.5359645 | -0.22151616 | 688 |
| NEFA_20_3 | 0.00035212 | -0.29431193 | 0.00052573 | -0.45520537 | -0.13341848 | 691 |
| NEFA_20_4 | 0.87825468 | -0.01257945 | 0.89490405 | -0.17375717 | 0.14859826 | 691 |
| NEFA_22_6 | 1.854E-07 | -0.40588124 | 3.559E-07 | -0.5572725 | -0.25448997 | 746 |
| NEFA_24_1 | 4.5783E-05 | 0.30512779 | 7.3458E-05 | 0.15900309 | 0.45125248 | 796 |
| NEFA_12_1 | 7.0499E-06 | -0.35305992 | 1.1842E-05 | -0.50623139 | -0.19988845 | 726 |
| NEFA_13_1 | 0.83727316 | 0.02175302 | 0.85720823 | -0.18629989 | 0.22980592 | 429 |
| NEFA_14_2 | 0.01401852 | -0.21096239 | 0.01732173 | -0.37911973 | -0.04280504 | 649 |
| NEFA_16_2 | 4.6118E-07 | -0.4048169 | 8.4029E-07 | -0.56096849 | -0.2486653 | 700 |
| NEFA_18_4 | 0.01079396 | -0.22827449 | 0.01365119 | -0.40356934 | -0.05297964 | 564 |
| NEFA_19_0 | 0.09124277 | 0.14425938 | 0.10434679 | -0.02322295 | 0.31174171 | 617 |
| NEFA_19_1 | 0.06606061 | -0.14969838 | 0.07677315 | -0.30935572 | 0.00995895 | 702 |
| NEFA_20_5 | 1.31E-02 | 0.19949817 | 1.64E-02 | 0.04197567 | 0.35702068 | 702 |
| NEFA_22_4 | 0.01044799 | -0.21772434 | 0.01329182 | -0.38419035 | -0.05125833 | 617 |
| NEFA_22_5 | 0.93802931 | -0.00623756 | 0.9468371 | -0.16369956 | 0.15122444 | 702 |
| NEFA_24_4 | 6.851E-08 | -0.55534149 | 1.3766E-07 | -0.75416915 | -0.35651384 | 439 |
| NEFA_24_5 | 1.0436E-27 | -1.05692264 | 7.2377E-27 | -1.23568146 | -0.87816382 | 490 |
| NEFA_26_1 | 7.9944E-14 | 0.65967971 | 2.4209E-13 | 0.49046599 | 0.82889343 | 584 |

## Supplemental Table 5: Results for the ANOVA for the five MetS Factors: waist circumference, HDL, triglyceride, glucose and systolic blood pressure as outcome and three level sex variable (males, *nh*females and *h*females) * single metabolite concentration interaction as predictor. Adjusted for ethnicity. P-value, FDR corrected P-values and number of observations per metabolite reported.

### Waist circumference

| Analytes | P-VALUE | FDR P-Value | Number of observations |
| --- | --- | --- | --- |
| Carn | 0.77398611 | 0.8667032 | 996 |
| Carn.a.C10.0 | 0.03523295 | 0.23127167 | 622 |
| Carn.a.C10.1 | 0.00242669 | 0.06521739 | 620 |
| Carn.a.C12.0 | 0.47176932 | 0.69472879 | 616 |
| Carn.a.C12.1 | 0.00222942 | 0.06521739 | 549 |
| Carn.a.C14.0 | 0.51226552 | 0.71595464 | 823 |
| Carn.a.C14.1 | 0.09132989 | 0.32726545 | 896 |
| Carn.a.C14.2 | 0.01781328 | 0.18237404 | 887 |
| Carn.a.C16.0 | 0.83065243 | 0.89295136 | 991 |
| Carn.a.C16.1 | 0.02039473 | 0.19931214 | 894 |
| Carn.a.C18.0 | 0.20680037 | 0.47576811 | 796 |
| Carn.a.C18.1 | 0.52507409 | 0.71905051 | 995 |
| Carn.a.C2.0 | 0.48857185 | 0.70028632 | 925 |
| Carn.a.C3.0 | 0.22476303 | 0.49818609 | 994 |
| Carn.a.C4.0 | 0.28477498 | 0.56083487 | 861 |
| Carn.a.C4.0.DC | 0.04530252 | 0.25365456 | 546 |
| Carn.a.C5.0 | 0.84041154 | 0.89449743 | 666 |
| Carn.a.C6.0 | 0.00184859 | 0.06521739 | 846 |
| Carn.a.C8.1 | 0.14478298 | 0.40426415 | 748 |
| lyso.PC.a.C14.0 | 0.32363704 | 0.59217599 | 991 |
| lyso.PC.a.C16.0 | 0.05315145 | 0.25537361 | 925 |
| lyso.PC.a.C16.1 | 0.02971777 | 0.2206473 | 996 |
| lyso.PC.a.C18.0 | 0.13834298 | 0.39658322 | 996 |
| lyso.PC.a.C18.1 | 0.00060259 | 0.04925788 | 925 |
| lyso.PC.a.C18.2 | 0.00114824 | 0.0617177 | 925 |
| lyso.PC.a.C18.3 | 0.0002935 | 0.04925788 | 793 |
| lyso.PC.a.C18.6 | 0.15949197 | 0.40822348 | 707 |
| lyso.PC.a.C20.2 | 0.35719494 | 0.62269079 | 645 |
| lyso.PC.a.C20.3 | 0.03757021 | 0.23485644 | 853 |
| lyso.PC.a.C20.4 | 0.02143166 | 0.20033944 | 996 |
| lyso.PC.a.C20.5 | 0.00526096 | 0.10282794 | 920 |
| lyso.PC.a.C22.5 | 0.0538154 | 0.25537361 | 874 |
| lyso.PC.a.C22.6 | 0.0131902 | 0.15941669 | 939 |
| lyso.PC.e.C16.0 | 0.86108617 | 0.91198781 | 995 |
| lyso.PC.e.C18.0 | 0.17982862 | 0.43718715 | 967 |
| lyso.PC.e.C18.1 | 0.06414035 | 0.25537361 | 707 |
| PC.aa.C30.0 | 0.8370785 | 0.89449743 | 988 |
| PC.aa.C30.1 | 0.61504077 | 0.77329687 | 701 |
| PC.aa.C30.2 | 0.37197764 | 0.63980155 | 638 |
| PC.aa.C32.0 | 0.28816244 | 0.56083487 | 996 |
| PC.aa.C32.1 | 0.5915076 | 0.77212557 | 864 |
| PC.aa.C32.2 | 0.64118373 | 0.78774001 | 836 |
| PC.aa.C32.3 | 0.89375697 | 0.9282983 | 868 |
| PC.aa.C34.0 | 0.93710018 | 0.95414791 | 706 |
| PC.aa.C34.1 | 0.59726536 | 0.77212557 | 935 |
| PC.aa.C34.2 | 0.11201829 | 0.35298249 | 925 |
| PC.aa.C34.3 | 0.95121941 | 0.95566436 | 925 |
| PC.aa.C34.4 | 0.60780788 | 0.77212557 | 865 |
| PC.aa.C34.5 | 0.72411614 | 0.84332531 | 875 |
| PC.aa.C36.0 | 0.46930562 | 0.69472879 | 924 |
| PC.aa.C36.1 | 0.2120075 | 0.47576811 | 924 |
| PC.aa.C36.2 | 0.5977104 | 0.77212557 | 925 |
| PC.aa.C36.3 | 0.20982862 | 0.47576811 | 936 |
| PC.aa.C36.4 | 0.0313645 | 0.22477892 | 865 |
| PC.aa.C36.5 | 0.65949576 | 0.80563403 | 808 |
| PC.aa.C36.6 | 0.29589153 | 0.56297946 | 983 |
| PC.aa.C38.0 | 0.44453232 | 0.69264427 | 867 |
| PC.aa.C38.1 | 0.04601176 | 0.25365456 | 796 |
| PC.aa.C38.2 | 0.13012886 | 0.37807711 | 936 |
| PC.aa.C38.3 | 0.28715347 | 0.56083487 | 836 |
| PC.aa.C38.4 | 0.2779238 | 0.56083487 | 836 |
| PC.aa.C38.5 | 0.94083422 | 0.95414791 | 836 |
| PC.aa.C38.6 | 0.09973281 | 0.32988546 | 836 |
| PC.aa.C40.0 | 0.60674132 | 0.77212557 | 800 |
| PC.aa.C40.1 | 0.73936926 | 0.85136572 | 573 |
| PC.aa.C40.3 | 0.80747237 | 0.87549435 | 584 |
| PC.aa.C40.4 | 0.38695398 | 0.64513119 | 925 |
| PC.aa.C40.5 | 0.5945548 | 0.77212557 | 836 |
| PC.aa.C40.6 | 0.29369305 | 0.56297946 | 901 |
| PC.aa.C42.0 | 0.61920578 | 0.77400723 | 796 |
| PC.aa.C42.1 | 0.20006089 | 0.47576811 | 643 |
| PC.aa.C42.2 | 0.06281939 | 0.25537361 | 629 |
| PC.aa.C42.4 | 0.68243904 | 0.81824972 | 549 |
| PC.aa.C42.5 | 0.05787381 | 0.25537361 | 731 |
| PC.aa.C42.6 | 0.44401015 | 0.69264427 | 702 |
| PC.aa.C43.4 | 0.88359119 | 0.92219469 | 626 |
| PC.aa.C43.6 | 0.60609525 | 0.77212557 | 925 |
| PC.aa.C44.12 | 0.31678888 | 0.59217599 | 925 |
| PC.ae.C30.0 | 0.38125164 | 0.64513119 | 849 |
| PC.ae.C30.1 | 0.56589347 | 0.76520186 | 758 |
| PC.ae.C32.0 | 0.61051789 | 0.77212557 | 996 |
| PC.ae.C32.1 | 0.02846963 | 0.2206473 | 996 |
| PC.ae.C32.2 | 0.01539496 | 0.17420607 | 995 |
| PC.ae.C34.0 | 0.87135207 | 0.9143225 | 935 |
| PC.ae.C34.1 | 0.41841128 | 0.67637914 | 936 |
| PC.ae.C34.2 | 0.27072025 | 0.56083487 | 936 |
| PC.ae.C34.3 | 0.05207227 | 0.25537361 | 936 |
| PC.ae.C34.4 | 0.74445003 | 0.85136572 | 869 |
| PC.ae.C36.0 | 0.28691523 | 0.56083487 | 929 |
| PC.ae.C36.1 | 0.60215585 | 0.77212557 | 925 |
| PC.ae.C36.2 | 0.3591333 | 0.62269079 | 925 |
| PC.ae.C36.3 | 0.4458454 | 0.69264427 | 996 |
| PC.ae.C36.4 | 0.27864875 | 0.56083487 | 865 |
| PC.ae.C36.5 | 0.11492453 | 0.35298249 | 865 |
| PC.ae.C36.6 | 0.2895473 | 0.56083487 | 734 |
| PC.ae.C38.0 | 0.67910953 | 0.81824972 | 925 |
| PC.ae.C38.2 | 0.75680504 | 0.85703577 | 925 |
| PC.ae.C38.3 | 0.48150009 | 0.69478201 | 925 |
| PC.ae.C38.4 | 0.09010539 | 0.32726545 | 865 |
| PC.ae.C38.5 | 0.32818325 | 0.59293613 | 865 |
| PC.ae.C38.6 | 0.27094996 | 0.56083487 | 935 |
| PC.ae.C40.0 | 0.23832678 | 0.52285978 | 925 |
| PC.ae.C40.1 | 0.72565201 | 0.84332531 | 925 |
| PC.ae.C40.2 | 0.63283724 | 0.78302605 | 867 |
| PC.ae.C40.3 | 0.91196767 | 0.93814856 | 868 |
| PC.ae.C40.4 | 0.09378981 | 0.32937578 | 925 |
| PC.ae.C40.5 | 0.44786083 | 0.69264427 | 925 |
| PC.ae.C40.6 | 0.34552708 | 0.61395307 | 836 |
| PC.ae.C42.0 | 0.06068161 | 0.25537361 | 870 |
| PC.ae.C42.1 | 0.45489275 | 0.69264427 | 796 |
| PC.ae.C42.2 | 0.66521515 | 0.8080297 | 868 |
| PC.ae.C42.3 | 0.87179587 | 0.9143225 | 933 |
| PC.ae.C42.4 | 0.63370481 | 0.78302605 | 925 |
| PC.ae.C42.5 | 0.43650716 | 0.69264427 | 868 |
| PC.ae.C42.6 | 0.53674249 | 0.73037744 | 994 |
| SM.a.C30.1 | 0.34352729 | 0.61395307 | 859 |
| SM.a.C32.0 | 0.17582849 | 0.43451868 | 564 |
| SM.a.C32.1 | 0.47825161 | 0.6947574 | 925 |
| SM.a.C32.2 | 0.10395123 | 0.338629 | 936 |
| SM.a.C33.1 | 0.25492286 | 0.54808415 | 925 |
| SM.a.C33.2 | 0.0991646 | 0.32988546 | 803 |
| SM.a.C34.0 | 0.00737644 | 0.12199494 | 628 |
| SM.a.C34.1 | 0.74079852 | 0.85136572 | 939 |
| SM.a.C34.2 | 0.16350566 | 0.41357314 | 996 |
| SM.a.C34.3 | 0.30388884 | 0.57312368 | 643 |
| SM.a.C35.0 | 0.94829666 | 0.95566436 | 735 |
| SM.a.C35.1 | 0.1292237 | 0.37807711 | 925 |
| SM.a.C36.0 | 0.01104045 | 0.14835599 | 643 |
| SM.a.C36.1 | 0.46391058 | 0.69264427 | 939 |
| SM.a.C36.2 | 0.69301687 | 0.82319683 | 996 |
| SM.a.C36.3 | 0.02891279 | 0.2206473 | 935 |
| SM.a.C37.1 | 0.16933278 | 0.42333196 | 868 |
| SM.a.C38.1 | 0.5147577 | 0.71595464 | 993 |
| SM.a.C38.2 | 0.08540733 | 0.31659613 | 996 |
| SM.a.C39.1 | 0.07829457 | 0.30606061 | 868 |
| SM.a.C39.2 | 0.15770475 | 0.40822348 | 935 |
| SM.a.C40.1 | 0.1431269 | 0.40426415 | 860 |
| SM.a.C40.2 | 0.18097514 | 0.43718715 | 808 |
| SM.a.C40.3 | 0.80479537 | 0.87549435 | 712 |
| SM.a.C40.4 | 0.01764229 | 0.18237404 | 995 |
| SM.a.C40.5 | 0.09763053 | 0.32988546 | 877 |
| SM.a.C41.1 | 0.05701775 | 0.25537361 | 925 |
| SM.a.C41.2 | 0.15924515 | 0.40822348 | 925 |
| SM.a.C42.1 | 0.0347592 | 0.23127167 | 925 |
| SM.a.C42.2 | 0.00968891 | 0.14835599 | 925 |
| SM.a.C42.3 | 0.03549751 | 0.23127167 | 925 |
| SM.a.C42.4 | 0.40248977 | 0.65777907 | 865 |
| SM.a.C42.5 | 0.21091277 | 0.47576811 | 643 |
| SM.a.C42.6 | 0.0449503 | 0.25365456 | 865 |
| SM.a.C43.1 | 0.15267651 | 0.40822348 | 925 |
| SM.a.C43.2 | 0.00325711 | 0.07780884 | 925 |
| SM.a.C44.2 | 0.51446839 | 0.71595464 | 732 |
| SM.a.C44.6 | 0.72019223 | 0.84332531 | 810 |
| SM.a.C31.1 | 0.05074338 | 0.25537361 | 867 |
| SM.a.C33.3 | 0.50242837 | 0.71537815 | 935 |
| SM.a.C35.2 | 0.08008315 | 0.3074621 | 939 |
| SM.a.C37.3 | 0.35329482 | 0.62260972 | 938 |
| SM.a.C39.5 | 0.51615335 | 0.71595464 | 773 |
| SM.a.C41.0 | 0.00068732 | 0.04925788 | 776 |
| SM.a.C41.3 | 0.11383027 | 0.35298249 | 722 |
| SM.a.C43.0 | 0.26792022 | 0.56083487 | 703 |
| SM.a.C43.3 | 0.02660774 | 0.2206473 | 804 |
| Ala | 0.58929707 | 0.77212557 | 993 |
| Arg | 0.75804756 | 0.85703577 | 991 |
| Asn | 0.39007932 | 0.64513119 | 919 |
| Asp | 0.00228689 | 0.06521739 | 993 |
| Cit | 0.05693872 | 0.25537361 | 993 |
| Gln | 0.46169429 | 0.69264427 | 919 |
| Glu | 0.58957647 | 0.77212557 | 993 |
| Gly | 0.28082589 | 0.56083487 | 993 |
| His | 0.14901793 | 0.40822348 | 981 |
| Ile | 0.45358272 | 0.69264427 | 717 |
| Leu | 0.1202809 | 0.35917214 | 919 |
| Lys | 0.52101904 | 0.71807111 | 919 |
| Met | 0.78422708 | 0.87197092 | 919 |
| Orn | 0.01056954 | 0.14835599 | 919 |
| Phe | 0.90815454 | 0.93814856 | 919 |
| Pro | 0.04801358 | 0.25537361 | 993 |
| Trp | 0.81034128 | 0.87549435 | 993 |
| Ser | 0.38752911 | 0.64513119 | 993 |
| Thr | 0.15053725 | 0.40822348 | 936 |
| Tyr | 0.68504628 | 0.81824972 | 919 |
| Val | 0.04919648 | 0.25537361 | 919 |
| NEFA_12_0 | 0.11211635 | 0.35298249 | 788 |
| NEFA_14_0 | 0.4766036 | 0.6947574 | 964 |
| NEFA_14_1 | 0.06352635 | 0.25537361 | 989 |
| NEFA_15_0 | 0.24301687 | 0.52776392 | 986 |
| NEFA_15_1 | 0.78837464 | 0.87197092 | 681 |
| NEFA_16_0 | 0.03823244 | 0.23485644 | 851 |
| NEFA_16_1 | 0.04129889 | 0.24664618 | 989 |
| NEFA_17_0 | 0.32500822 | 0.59217599 | 917 |
| NEFA_17_1 | 0.06312138 | 0.25537361 | 902 |
| NEFA_18_0 | 0.32409617 | 0.59217599 | 839 |
| NEFA_18_1 | 0.157475 | 0.40822348 | 921 |
| NEFA_18_2 | 0.0045128 | 0.09702515 | 850 |
| NEFA_18_3 | 0.21243599 | 0.47576811 | 988 |
| NEFA_20_1 | 0.40384576 | 0.65777907 | 884 |
| NEFA_20_2 | 0.43232109 | 0.69264427 | 846 |
| NEFA_20_3 | 0.01334651 | 0.15941669 | 850 |
| NEFA_20_4 | 0.00736963 | 0.12199494 | 850 |
| NEFA_22_6 | 0.45796742 | 0.69264427 | 921 |
| NEFA_24_1 | 0.20196243 | 0.47576811 | 988 |
| NEFA_12_1 | 0.11786807 | 0.35692442 | 895 |
| NEFA_13_1 | 0.72060871 | 0.84332531 | 528 |
| NEFA_14_2 | 0.79579993 | 0.8729438 | 797 |
| NEFA_16_2 | 0.02690606 | 0.2206473 | 865 |
| NEFA_18_4 | 0.93671918 | 0.95414791 | 698 |
| NEFA_19_0 | 0.98692902 | 0.98692902 | 762 |
| NEFA_19_1 | 0.09498278 | 0.32937578 | 861 |
| NEFA_20_5 | 0.76136666 | 0.85703577 | 861 |
| NEFA_22_4 | 0.02976173 | 0.2206473 | 762 |
| NEFA_22_5 | 0.06086855 | 0.25537361 | 861 |
| NEFA_24_4 | 0.79085734 | 0.87197092 | 535 |
| NEFA_24_5 | 0.08518219 | 0.31659613 | 602 |
| NEFA_26_1 | 0.38858143 | 0.64513119 | 714 |

### HDL

| Analytes | P-VALUE | FDR P-Value | Number of observations |
| --- | --- | --- | --- |
| Carn | 0.12195434 | 0.30006152 | 996 |
| Carn.a.C10.0 | 0.43709287 | 0.63928549 | 622 |
| Carn.a.C10.1 | 0.67915471 | 0.81574448 | 620 |
| Carn.a.C12.0 | 0.48202167 | 0.67295233 | 616 |
| Carn.a.C12.1 | 0.71287955 | 0.83975885 | 549 |
| Carn.a.C14.0 | 0.87503648 | 0.92221982 | 823 |
| Carn.a.C14.1 | 0.71774772 | 0.83975885 | 896 |
| Carn.a.C14.2 | 0.82256132 | 0.88425342 | 887 |
| Carn.a.C16.0 | 0.58141078 | 0.74476503 | 991 |
| Carn.a.C16.1 | 0.99341285 | 0.99803328 | 894 |
| Carn.a.C18.0 | 0.00971136 | 0.0699686 | 796 |
| Carn.a.C18.1 | 0.31346057 | 0.49192718 | 995 |
| Carn.a.C2.0 | 0.73011038 | 0.83975885 | 925 |
| Carn.a.C3.0 | 0.42245797 | 0.62211276 | 994 |
| Carn.a.C4.0 | 0.38355672 | 0.58073729 | 861 |
| Carn.a.C4.0.DC | 0.53488739 | 0.72221702 | 546 |
| Carn.a.C5.0 | 0.12845926 | 0.30687491 | 666 |
| Carn.a.C6.0 | 0.09878997 | 0.25902248 | 846 |
| Carn.a.C8.1 | 0.31726141 | 0.49428407 | 748 |
| lyso.PC.a.C14.0 | 0.65448851 | 0.79500016 | 991 |
| lyso.PC.a.C16.0 | 0.000261 | 0.00431655 | 925 |
| lyso.PC.a.C16.1 | 0.00254676 | 0.02488882 | 996 |
| lyso.PC.a.C18.0 | 0.00195285 | 0.01999345 | 996 |
| lyso.PC.a.C18.1 | 4.1348E-11 | 8.8898E-09 | 925 |
| lyso.PC.a.C18.2 | 1.1866E-10 | 1.2756E-08 | 925 |
| lyso.PC.a.C18.3 | 1.403E-07 | 1.0055E-05 | 793 |
| lyso.PC.a.C18.6 | 0.02648707 | 0.11713616 | 707 |
| lyso.PC.a.C20.2 | 0.0905876 | 0.24044856 | 645 |
| lyso.PC.a.C20.3 | 0.00140696 | 0.01512483 | 853 |
| lyso.PC.a.C20.4 | 4.38E-05 | 0.00104633 | 996 |
| lyso.PC.a.C20.5 | 9.2092E-06 | 0.00037877 | 920 |
| lyso.PC.a.C22.5 | 0.00300005 | 0.02804393 | 874 |
| lyso.PC.a.C22.6 | 0.00042079 | 0.00532178 | 939 |
| lyso.PC.e.C16.0 | 0.16256523 | 0.33603926 | 995 |
| lyso.PC.e.C18.0 | 0.00527726 | 0.04727541 | 967 |
| lyso.PC.e.C18.1 | 3.5189E-05 | 0.00104633 | 707 |
| PC.aa.C30.0 | 0.83924477 | 0.89769963 | 988 |
| PC.aa.C30.1 | 0.00994152 | 0.0699686 | 701 |
| PC.aa.C30.2 | 0.48933823 | 0.67626216 | 638 |
| PC.aa.C32.0 | 0.14386364 | 0.3221946 | 996 |
| PC.aa.C32.1 | 0.36401363 | 0.55902093 | 864 |
| PC.aa.C32.2 | 0.1344658 | 0.31424072 | 836 |
| PC.aa.C32.3 | 0.04249277 | 0.15602973 | 868 |
| PC.aa.C34.0 | 0.06037754 | 0.19374882 | 706 |
| PC.aa.C34.1 | 0.03656497 | 0.14558275 | 935 |
| PC.aa.C34.2 | 0.04991124 | 0.1652193 | 925 |
| PC.aa.C34.3 | 0.07354386 | 0.21562249 | 925 |
| PC.aa.C34.4 | 0.07930068 | 0.22548008 | 865 |
| PC.aa.C34.5 | 0.15703909 | 0.3326802 | 875 |
| PC.aa.C36.0 | 0.00584575 | 0.04833987 | 924 |
| PC.aa.C36.1 | 0.44855869 | 0.64724912 | 924 |
| PC.aa.C36.2 | 0.31984359 | 0.4947221 | 925 |
| PC.aa.C36.3 | 0.28148934 | 0.46943244 | 936 |
| PC.aa.C36.4 | 0.15782967 | 0.3326802 | 865 |
| PC.aa.C36.5 | 0.11741962 | 0.29354905 | 808 |
| PC.aa.C36.6 | 0.04924331 | 0.1652193 | 983 |
| PC.aa.C38.0 | 0.01368172 | 0.08171028 | 867 |
| PC.aa.C38.1 | 0.07421425 | 0.21562249 | 796 |
| PC.aa.C38.2 | 0.89584769 | 0.93046983 | 936 |
| PC.aa.C38.3 | 0.00032519 | 0.00474555 | 836 |
| PC.aa.C38.4 | 6.7979E-05 | 0.00146156 | 836 |
| PC.aa.C38.5 | 0.02669615 | 0.11713616 | 836 |
| PC.aa.C38.6 | 0.00117329 | 0.01327672 | 836 |
| PC.aa.C40.0 | 0.12281588 | 0.30006152 | 800 |
| PC.aa.C40.1 | 0.08531539 | 0.2292851 | 573 |
| PC.aa.C40.3 | 0.53746383 | 0.72221702 | 584 |
| PC.aa.C40.4 | 0.08353669 | 0.2273467 | 925 |
| PC.aa.C40.5 | 0.0211735 | 0.10116226 | 836 |
| PC.aa.C40.6 | 7.6632E-06 | 0.00037877 | 901 |
| PC.aa.C42.0 | 0.1552321 | 0.3326802 | 796 |
| PC.aa.C42.1 | 0.03048563 | 0.12851786 | 643 |
| PC.aa.C42.2 | 0.07195124 | 0.21485441 | 629 |
| PC.aa.C42.4 | 0.89427452 | 0.93046983 | 549 |
| PC.aa.C42.5 | 0.11151357 | 0.28542165 | 731 |
| PC.aa.C42.6 | 0.04092925 | 0.15602973 | 702 |
| PC.aa.C43.4 | 0.74035854 | 0.84220681 | 626 |
| PC.aa.C43.6 | 0.53336648 | 0.72221702 | 925 |
| PC.aa.C44.12 | 0.97811644 | 0.98923943 | 925 |
| PC.ae.C30.0 | 0.12676308 | 0.30622542 | 849 |
| PC.ae.C30.1 | 0.25088218 | 0.43878729 | 758 |
| PC.ae.C32.0 | 0.80806539 | 0.87378174 | 996 |
| PC.ae.C32.1 | 0.25264733 | 0.43878729 | 996 |
| PC.ae.C32.2 | 0.0442378 | 0.15851879 | 995 |
| PC.ae.C34.0 | 0.30519163 | 0.48604593 | 935 |
| PC.ae.C34.1 | 0.1457496 | 0.32305325 | 936 |
| PC.ae.C34.2 | 0.49068324 | 0.67626216 | 936 |
| PC.ae.C34.3 | 0.0100885 | 0.0699686 | 936 |
| PC.ae.C34.4 | 0.27957876 | 0.46943244 | 869 |
| PC.ae.C36.0 | 0.01506234 | 0.08303599 | 929 |
| PC.ae.C36.1 | 0.06650257 | 0.2042579 | 925 |
| PC.ae.C36.2 | 0.01362602 | 0.08171028 | 925 |
| PC.ae.C36.3 | 0.17413223 | 0.34433571 | 996 |
| PC.ae.C36.4 | 0.01479947 | 0.08303599 | 865 |
| PC.ae.C36.5 | 0.01870377 | 0.09437143 | 865 |
| PC.ae.C36.6 | 0.29069969 | 0.48077257 | 734 |
| PC.ae.C38.0 | 0.04138972 | 0.15602973 | 925 |
| PC.ae.C38.2 | 0.19824221 | 0.38055425 | 925 |
| PC.ae.C38.3 | 0.08184751 | 0.2273467 | 925 |
| PC.ae.C38.4 | 0.01807826 | 0.09437143 | 865 |
| PC.ae.C38.5 | 0.04841621 | 0.1652193 | 865 |
| PC.ae.C38.6 | 0.03990986 | 0.15601126 | 935 |
| PC.ae.C40.0 | 0.00571917 | 0.04833987 | 925 |
| PC.ae.C40.1 | 0.07077904 | 0.21433088 | 925 |
| PC.ae.C40.2 | 0.15635708 | 0.3326802 | 867 |
| PC.ae.C40.3 | 0.55940634 | 0.74162973 | 868 |
| PC.ae.C40.4 | 0.28165947 | 0.46943244 | 925 |
| PC.ae.C40.5 | 0.24275493 | 0.43641903 | 925 |
| PC.ae.C40.6 | 0.00033108 | 0.00474555 | 836 |
| PC.ae.C42.0 | 0.14132279 | 0.3221946 | 870 |
| PC.ae.C42.1 | 0.01430976 | 0.08303599 | 796 |
| PC.ae.C42.2 | 0.76410419 | 0.85563751 | 868 |
| PC.ae.C42.3 | 0.11112541 | 0.28542165 | 933 |
| PC.ae.C42.4 | 0.68940253 | 0.82345302 | 925 |
| PC.ae.C42.5 | 0.77002011 | 0.85779443 | 868 |
| PC.ae.C42.6 | 0.38859325 | 0.58424859 | 994 |
| SM.a.C30.1 | 0.44134153 | 0.64113803 | 859 |
| SM.a.C32.0 | 0.30232177 | 0.48514849 | 564 |
| SM.a.C32.1 | 0.1774492 | 0.34683253 | 925 |
| SM.a.C32.2 | 0.16840709 | 0.33838808 | 936 |
| SM.a.C33.1 | 0.99803328 | 0.99803328 | 925 |
| SM.a.C33.2 | 0.92402722 | 0.94672563 | 803 |
| SM.a.C34.0 | 0.62622194 | 0.77233203 | 628 |
| SM.a.C34.1 | 0.16792586 | 0.33838808 | 939 |
| SM.a.C34.2 | 0.62864235 | 0.77233203 | 996 |
| SM.a.C34.3 | 0.73039491 | 0.83975885 | 643 |
| SM.a.C35.0 | 0.80576053 | 0.87378174 | 735 |
| SM.a.C35.1 | 0.86442836 | 0.92005989 | 925 |
| SM.a.C36.0 | 0.00100442 | 0.01199726 | 643 |
| SM.a.C36.1 | 0.56302313 | 0.74162973 | 939 |
| SM.a.C36.2 | 0.7037088 | 0.8358972 | 996 |
| SM.a.C36.3 | 0.54136759 | 0.72294429 | 935 |
| SM.a.C37.1 | 0.86915256 | 0.92053104 | 868 |
| SM.a.C38.1 | 0.04281746 | 0.15602973 | 993 |
| SM.a.C38.2 | 0.13356045 | 0.31424072 | 996 |
| SM.a.C39.1 | 0.25286688 | 0.43878729 | 868 |
| SM.a.C39.2 | 0.88342096 | 0.92651466 | 935 |
| SM.a.C40.1 | 0.00878719 | 0.06747303 | 860 |
| SM.a.C40.2 | 0.18581481 | 0.35991156 | 808 |
| SM.a.C40.3 | 0.21543171 | 0.40276364 | 712 |
| SM.a.C40.4 | 0.05216853 | 0.16994294 | 995 |
| SM.a.C40.5 | 0.01288056 | 0.08145062 | 877 |
| SM.a.C41.1 | 0.08259372 | 0.2273467 | 925 |
| SM.a.C41.2 | 0.75338181 | 0.852511 | 925 |
| SM.a.C42.1 | 0.02565014 | 0.11713616 | 925 |
| SM.a.C42.2 | 0.07970459 | 0.22548008 | 925 |
| SM.a.C42.3 | 0.31120067 | 0.49192718 | 925 |
| SM.a.C42.4 | 0.00025198 | 0.00431655 | 865 |
| SM.a.C42.5 | 0.035858 | 0.14546168 | 643 |
| SM.a.C42.6 | 0.00012463 | 0.00243594 | 865 |
| SM.a.C43.1 | 0.20258239 | 0.38544437 | 925 |
| SM.a.C43.2 | 0.4583246 | 0.64828809 | 925 |
| SM.a.C44.2 | 0.79284699 | 0.87378174 | 732 |
| SM.a.C44.6 | 4.2721E-05 | 0.00104633 | 810 |
| SM.a.C31.1 | 0.80547934 | 0.87378174 | 867 |
| SM.a.C33.3 | 0.59159885 | 0.7463702 | 935 |
| SM.a.C35.2 | 0.72792101 | 0.83975885 | 939 |
| SM.a.C37.3 | 0.22896318 | 0.42001121 | 938 |
| SM.a.C39.5 | 0.17457019 | 0.34433571 | 773 |
| SM.a.C41.0 | 0.00041257 | 0.00532178 | 776 |
| SM.a.C41.3 | 0.06192302 | 0.19578602 | 722 |
| SM.a.C43.0 | 0.1641122 | 0.33603926 | 703 |
| SM.a.C43.3 | 0.98003721 | 0.98923943 | 804 |
| Ala | 0.97642131 | 0.98923943 | 993 |
| Arg | 0.5705069 | 0.74338778 | 991 |
| Asn | 0.25306802 | 0.43878729 | 919 |
| Asp | 0.21949214 | 0.40681732 | 993 |
| Cit | 0.58195593 | 0.74476503 | 993 |
| Gln | 0.30237162 | 0.48514849 | 919 |
| Glu | 0.36889398 | 0.56249791 | 993 |
| Gly | 0.04995002 | 0.1652193 | 993 |
| His | 0.28012995 | 0.46943244 | 981 |
| Ile | 0.45217674 | 0.64812 | 717 |
| Leu | 0.59709616 | 0.7463702 | 919 |
| Lys | 0.41786504 | 0.61959299 | 919 |
| Met | 0.39992268 | 0.59710677 | 919 |
| Orn | 0.02074036 | 0.10116226 | 919 |
| Phe | 0.91864026 | 0.94672563 | 919 |
| Pro | 0.76393817 | 0.85563751 | 993 |
| Trp | 0.66385533 | 0.80184773 | 993 |
| Ser | 0.00674615 | 0.05371932 | 993 |
| Thr | 0.03370955 | 0.13937601 | 936 |
| Tyr | 0.21517213 | 0.40276364 | 919 |
| Val | 0.72905045 | 0.83975885 | 919 |
| NEFA_12_0 | 0.80083712 | 0.87378174 | 788 |
| NEFA_14_0 | 0.15951085 | 0.33295953 | 964 |
| NEFA_14_1 | 0.45738038 | 0.64828809 | 989 |
| NEFA_15_0 | 0.14791088 | 0.32449835 | 986 |
| NEFA_15_1 | 0.0165769 | 0.08910083 | 681 |
| NEFA_16_0 | 0.14020763 | 0.3221946 | 851 |
| NEFA_16_1 | 0.64399976 | 0.78670425 | 989 |
| NEFA_17_0 | 0.06532522 | 0.2035496 | 917 |
| NEFA_17_1 | 0.2611189 | 0.44912451 | 902 |
| NEFA_18_0 | 0.01278717 | 0.08145062 | 839 |
| NEFA_18_1 | 0.59640892 | 0.7463702 | 921 |
| NEFA_18_2 | 0.29407254 | 0.48263814 | 850 |
| NEFA_18_3 | 0.59615959 | 0.7463702 | 988 |
| NEFA_20_1 | 0.92470876 | 0.94672563 | 884 |
| NEFA_20_2 | 0.56570826 | 0.74162973 | 846 |
| NEFA_20_3 | 0.01231655 | 0.08145062 | 850 |
| NEFA_20_4 | 0.01887429 | 0.09437143 | 850 |
| NEFA_22_6 | 0.025984 | 0.11713616 | 921 |
| NEFA_24_1 | 0.80875613 | 0.87378174 | 988 |
| NEFA_12_1 | 0.5359882 | 0.72221702 | 895 |
| NEFA_13_1 | 0.73795211 | 0.84220681 | 528 |
| NEFA_14_2 | 0.5785479 | 0.74476503 | 797 |
| NEFA_16_2 | 0.11607744 | 0.29354905 | 865 |
| NEFA_18_4 | 0.46778972 | 0.65735156 | 698 |
| NEFA_19_0 | 0.23051778 | 0.42001121 | 762 |
| NEFA_19_1 | 0.30008243 | 0.48514849 | 861 |
| NEFA_20_5 | 0.61034971 | 0.75852709 | 861 |
| NEFA_22_4 | 0.02882554 | 0.12394983 | 762 |
| NEFA_22_5 | 0.04633526 | 0.16331281 | 861 |
| NEFA_24_4 | 0.24358272 | 0.43641903 | 535 |
| NEFA_24_5 | 1.057E-05 | 0.00037877 | 602 |
| NEFA_26_1 | 0.14293069 | 0.3221946 | 714 |

### Triglycerides

| Analytes | P-VALUE | FDR P-Value | Number of observations |
| --- | --- | --- | --- |
| Carn | 0.01874481 | 0.09829596 | 996 |
| Carn.a.C10.0 | 0.01732255 | 0.09549612 | 622 |
| Carn.a.C10.1 | 0.30960322 | 0.50812742 | 620 |
| Carn.a.C12.0 | 0.40949425 | 0.58694177 | 616 |
| Carn.a.C12.1 | 0.43974209 | 0.60605481 | 549 |
| Carn.a.C14.0 | 0.73544913 | 0.82172871 | 823 |
| Carn.a.C14.1 | 0.37578033 | 0.58356624 | 896 |
| Carn.a.C14.2 | 0.67483522 | 0.78237493 | 887 |
| Carn.a.C16.0 | 0.13033494 | 0.31843195 | 991 |
| Carn.a.C16.1 | 0.54538702 | 0.67752908 | 894 |
| Carn.a.C18.0 | 0.16344971 | 0.37112977 | 796 |
| Carn.a.C18.1 | 0.07703438 | 0.2270739 | 995 |
| Carn.a.C2.0 | 0.68048424 | 0.78237493 | 925 |
| Carn.a.C3.0 | 0.00262166 | 0.04185712 | 994 |
| Carn.a.C4.0 | 0.01284157 | 0.07888392 | 861 |
| Carn.a.C4.0.DC | 0.97832597 | 0.98791317 | 546 |
| Carn.a.C5.0 | 0.06750425 | 0.2201648 | 666 |
| Carn.a.C6.0 | 0.07464465 | 0.2270739 | 846 |
| Carn.a.C8.1 | 0.86826424 | 0.92414263 | 748 |
| lyso.PC.a.C14.0 | 0.21611467 | 0.41860049 | 991 |
| lyso.PC.a.C16.0 | 0.00183248 | 0.03283196 | 925 |
| lyso.PC.a.C16.1 | 0.03026333 | 0.1275807 | 996 |
| lyso.PC.a.C18.0 | 3.9036E-06 | 0.00065519 | 996 |
| lyso.PC.a.C18.1 | 0.02469751 | 0.11062426 | 925 |
| lyso.PC.a.C18.2 | 0.40824183 | 0.58694177 | 925 |
| lyso.PC.a.C18.3 | 0.00447167 | 0.04704518 | 793 |
| lyso.PC.a.C18.6 | 0.01633446 | 0.09241865 | 707 |
| lyso.PC.a.C20.2 | 0.76258969 | 0.82806456 | 645 |
| lyso.PC.a.C20.3 | 0.18993923 | 0.4003621 | 853 |
| lyso.PC.a.C20.4 | 0.16447693 | 0.37112977 | 996 |
| lyso.PC.a.C20.5 | 0.42354091 | 0.59327914 | 920 |
| lyso.PC.a.C22.5 | 0.48984161 | 0.63063441 | 874 |
| lyso.PC.a.C22.6 | 0.40757922 | 0.58694177 | 939 |
| lyso.PC.e.C16.0 | 0.94632067 | 0.97348778 | 995 |
| lyso.PC.e.C18.0 | 0.37798277 | 0.58356624 | 967 |
| lyso.PC.e.C18.1 | 0.00672234 | 0.05181116 | 707 |
| PC.aa.C30.0 | 0.10454658 | 0.27750018 | 988 |
| PC.aa.C30.1 | 0.07604941 | 0.2270739 | 701 |
| PC.aa.C30.2 | 0.09998743 | 0.27120066 | 638 |
| PC.aa.C32.0 | 0.06847063 | 0.2201648 | 996 |
| PC.aa.C32.1 | 0.02434591 | 0.11062426 | 864 |
| PC.aa.C32.2 | 0.07775537 | 0.2270739 | 836 |
| PC.aa.C32.3 | 0.00100687 | 0.0216477 | 868 |
| PC.aa.C34.0 | 0.01051487 | 0.0706468 | 706 |
| PC.aa.C34.1 | 0.00045042 | 0.01795475 | 935 |
| PC.aa.C34.2 | 0.01109832 | 0.07230726 | 925 |
| PC.aa.C34.3 | 0.00146627 | 0.02865891 | 925 |
| PC.aa.C34.4 | 0.01455015 | 0.08689674 | 865 |
| PC.aa.C34.5 | 0.03157971 | 0.13056994 | 875 |
| PC.aa.C36.0 | 0.00589769 | 0.04876932 | 924 |
| PC.aa.C36.1 | 1.7406E-05 | 0.00124744 | 924 |
| PC.aa.C36.2 | 6.0948E-06 | 0.00065519 | 925 |
| PC.aa.C36.3 | 0.00070875 | 0.01795475 | 936 |
| PC.aa.C36.4 | 0.00468327 | 0.04704518 | 865 |
| PC.aa.C36.5 | 0.01172118 | 0.07411925 | 808 |
| PC.aa.C36.6 | 0.04412678 | 0.16357343 | 983 |
| PC.aa.C38.0 | 0.00527651 | 0.0483465 | 867 |
| PC.aa.C38.1 | 0.02017241 | 0.10086203 | 796 |
| PC.aa.C38.2 | 0.01874236 | 0.09829596 | 936 |
| PC.aa.C38.3 | 0.00051215 | 0.01795475 | 836 |
| PC.aa.C38.4 | 0.00292026 | 0.04185712 | 836 |
| PC.aa.C38.5 | 0.0031881 | 0.04284015 | 836 |
| PC.aa.C38.6 | 0.05736005 | 0.20217068 | 836 |
| PC.aa.C40.0 | 0.01552205 | 0.09019568 | 800 |
| PC.aa.C40.1 | 0.03634957 | 0.14209376 | 573 |
| PC.aa.C40.3 | 0.15895259 | 0.36999968 | 584 |
| PC.aa.C40.4 | 0.00562169 | 0.0483465 | 925 |
| PC.aa.C40.5 | 0.00065492 | 0.01795475 | 836 |
| PC.aa.C40.6 | 0.29489465 | 0.49149109 | 901 |
| PC.aa.C42.0 | 0.00757551 | 0.05429115 | 796 |
| PC.aa.C42.1 | 0.07750005 | 0.2270739 | 643 |
| PC.aa.C42.2 | 0.48590036 | 0.62932879 | 629 |
| PC.aa.C42.4 | 0.08090081 | 0.23191565 | 549 |
| PC.aa.C42.5 | 0.4957282 | 0.63066014 | 731 |
| PC.aa.C42.6 | 0.18151406 | 0.39025523 | 702 |
| PC.aa.C43.4 | 0.27286042 | 0.47310476 | 626 |
| PC.aa.C43.6 | 0.04508107 | 0.16427846 | 925 |
| PC.aa.C44.12 | 0.59560409 | 0.71860803 | 925 |
| PC.ae.C30.0 | 0.02289409 | 0.10800894 | 849 |
| PC.ae.C30.1 | 0.65709886 | 0.77200139 | 758 |
| PC.ae.C32.0 | 0.09946639 | 0.27120066 | 996 |
| PC.ae.C32.1 | 0.0686095 | 0.2201648 | 996 |
| PC.ae.C32.2 | 0.22503965 | 0.42817278 | 995 |
| PC.ae.C34.0 | 0.058679 | 0.20348364 | 935 |
| PC.ae.C34.1 | 0.0067475 | 0.05181116 | 936 |
| PC.ae.C34.2 | 0.49289936 | 0.63066014 | 936 |
| PC.ae.C34.3 | 0.74168531 | 0.82197084 | 936 |
| PC.ae.C34.4 | 0.03243762 | 0.13158658 | 869 |
| PC.ae.C36.0 | 0.01942353 | 0.09942996 | 929 |
| PC.ae.C36.1 | 0.00545587 | 0.0483465 | 925 |
| PC.ae.C36.2 | 0.02691501 | 0.11573454 | 925 |
| PC.ae.C36.3 | 0.10858099 | 0.28126402 | 996 |
| PC.ae.C36.4 | 0.16004637 | 0.36999968 | 865 |
| PC.ae.C36.5 | 0.31940028 | 0.51632376 | 865 |
| PC.ae.C36.6 | 0.07204092 | 0.2270739 | 734 |
| PC.ae.C38.0 | 0.00345506 | 0.04369629 | 925 |
| PC.ae.C38.2 | 0.07815567 | 0.2270739 | 925 |
| PC.ae.C38.3 | 0.11251316 | 0.28798012 | 925 |
| PC.ae.C38.4 | 0.17976755 | 0.39025523 | 865 |
| PC.ae.C38.5 | 0.33925272 | 0.54432339 | 865 |
| PC.ae.C38.6 | 0.04389214 | 0.16357343 | 935 |
| PC.ae.C40.0 | 0.2723 | 0.47310476 | 925 |
| PC.ae.C40.1 | 0.10091187 | 0.27120066 | 925 |
| PC.ae.C40.2 | 0.18369802 | 0.39104035 | 867 |
| PC.ae.C40.3 | 0.17566083 | 0.38537836 | 868 |
| PC.ae.C40.4 | 0.30442444 | 0.50347119 | 925 |
| PC.ae.C40.5 | 0.21994227 | 0.42221061 | 925 |
| PC.ae.C40.6 | 0.14024581 | 0.33503165 | 836 |
| PC.ae.C42.0 | 0.71419856 | 0.8124481 | 870 |
| PC.ae.C42.1 | 0.21580315 | 0.41860049 | 796 |
| PC.ae.C42.2 | 0.03403659 | 0.13551606 | 868 |
| PC.ae.C42.3 | 0.55079074 | 0.67752908 | 933 |
| PC.ae.C42.4 | 0.71994067 | 0.81466971 | 925 |
| PC.ae.C42.5 | 0.36126178 | 0.57111237 | 868 |
| PC.ae.C42.6 | 0.04085878 | 0.15686852 | 994 |
| SM.a.C30.1 | 0.38381718 | 0.58356624 | 859 |
| SM.a.C32.0 | 0.96647437 | 0.98541795 | 564 |
| SM.a.C32.1 | 0.25490313 | 0.44921454 | 925 |
| SM.a.C32.2 | 0.446449 | 0.60750972 | 936 |
| SM.a.C33.1 | 0.76894314 | 0.83076772 | 925 |
| SM.a.C33.2 | 0.93061923 | 0.96658519 | 803 |
| SM.a.C34.0 | 0.68008857 | 0.78237493 | 628 |
| SM.a.C34.1 | 0.41498234 | 0.5908689 | 939 |
| SM.a.C34.2 | 0.51364033 | 0.64503838 | 996 |
| SM.a.C34.3 | 0.22727303 | 0.42830481 | 643 |
| SM.a.C35.0 | 0.02665232 | 0.11573454 | 735 |
| SM.a.C35.1 | 0.20318632 | 0.4121232 | 925 |
| SM.a.C36.0 | 0.00056238 | 0.01795475 | 643 |
| SM.a.C36.1 | 0.38740072 | 0.58356624 | 939 |
| SM.a.C36.2 | 0.20578338 | 0.41348996 | 996 |
| SM.a.C36.3 | 0.36459439 | 0.57217369 | 935 |
| SM.a.C37.1 | 0.21092104 | 0.41603692 | 868 |
| SM.a.C38.1 | 0.00389523 | 0.04652634 | 993 |
| SM.a.C38.2 | 0.11857461 | 0.29992401 | 996 |
| SM.a.C39.1 | 0.19537363 | 0.40389741 | 868 |
| SM.a.C39.2 | 0.19343437 | 0.40377078 | 935 |
| SM.a.C40.1 | 0.85669688 | 0.91636731 | 860 |
| SM.a.C40.2 | 0.22909327 | 0.42830481 | 808 |
| SM.a.C40.3 | 0.00481393 | 0.04704518 | 712 |
| SM.a.C40.4 | 0.00705253 | 0.05228598 | 995 |
| SM.a.C40.5 | 0.00075159 | 0.01795475 | 877 |
| SM.a.C41.1 | 0.40531096 | 0.58694177 | 925 |
| SM.a.C41.2 | 0.23339573 | 0.42971922 | 925 |
| SM.a.C42.1 | 0.61377993 | 0.73020605 | 925 |
| SM.a.C42.2 | 0.4575654 | 0.6148535 | 925 |
| SM.a.C42.3 | 0.94383816 | 0.97348778 | 925 |
| SM.a.C42.4 | 0.002827 | 0.04185712 | 865 |
| SM.a.C42.5 | 0.06729215 | 0.2201648 | 643 |
| SM.a.C42.6 | 0.2787232 | 0.47559911 | 865 |
| SM.a.C43.1 | 0.35252207 | 0.56142404 | 925 |
| SM.a.C43.2 | 0.28173526 | 0.47695339 | 925 |
| SM.a.C44.2 | 0.16787432 | 0.37209258 | 732 |
| SM.a.C44.6 | 0.12826096 | 0.31696673 | 810 |
| SM.a.C31.1 | 0.47910884 | 0.62568599 | 867 |
| SM.a.C33.3 | 0.99532922 | 0.99532922 | 935 |
| SM.a.C35.2 | 0.75600211 | 0.82806456 | 939 |
| SM.a.C37.3 | 0.64880705 | 0.76644789 | 938 |
| SM.a.C39.5 | 0.39030327 | 0.58356624 | 773 |
| SM.a.C41.0 | 0.23944191 | 0.43260513 | 776 |
| SM.a.C41.3 | 0.87914102 | 0.93110995 | 722 |
| SM.a.C43.0 | 0.51273319 | 0.64503838 | 703 |
| SM.a.C43.3 | 0.89929466 | 0.94778604 | 804 |
| Ala | 0.31849315 | 0.51632376 | 993 |
| Arg | 0.58706814 | 0.71310537 | 991 |
| Asn | 0.24605865 | 0.43721164 | 919 |
| Asp | 0.61473161 | 0.73020605 | 993 |
| Cit | 0.39628219 | 0.58356624 | 993 |
| Gln | 0.59828297 | 0.71860803 | 919 |
| Glu | 0.09251456 | 0.25831986 | 993 |
| Gly | 0.83068253 | 0.89298372 | 993 |
| His | 0.47611411 | 0.62568599 | 981 |
| Ile | 0.20883297 | 0.41573231 | 717 |
| Leu | 0.99180961 | 0.99532922 | 919 |
| Lys | 0.75947537 | 0.82806456 | 919 |
| Met | 0.47830421 | 0.62568599 | 919 |
| Orn | 0.1466698 | 0.34652754 | 919 |
| Phe | 0.97872328 | 0.98791317 | 919 |
| Pro | 0.23460648 | 0.42971922 | 993 |
| Trp | 0.1083533 | 0.28126402 | 993 |
| Ser | 0.73764484 | 0.82172871 | 993 |
| Thr | 0.06567541 | 0.2201648 | 936 |
| Tyr | 0.13765639 | 0.33254071 | 919 |
| Val | 0.91890493 | 0.96372956 | 919 |
| NEFA_12_0 | 0.55147716 | 0.67752908 | 788 |
| NEFA_14_0 | 0.3956079 | 0.58356624 | 964 |
| NEFA_14_1 | 0.29228344 | 0.49094484 | 989 |
| NEFA_15_0 | 0.39050532 | 0.58356624 | 986 |
| NEFA_15_1 | 0.48017762 | 0.62568599 | 681 |
| NEFA_16_0 | 0.44324632 | 0.60699337 | 851 |
| NEFA_16_1 | 0.05065627 | 0.1815183 | 989 |
| NEFA_17_0 | 0.27527221 | 0.47346821 | 917 |
| NEFA_17_1 | 0.96708459 | 0.98541795 | 902 |
| NEFA_18_0 | 0.42495343 | 0.59327914 | 839 |
| NEFA_18_1 | 0.43798076 | 0.60605481 | 921 |
| NEFA_18_2 | 0.08313814 | 0.23519341 | 850 |
| NEFA_18_3 | 0.73564658 | 0.82172871 | 988 |
| NEFA_20_1 | 0.46549793 | 0.62162767 | 884 |
| NEFA_20_2 | 0.42213742 | 0.59327914 | 846 |
| NEFA_20_3 | 0.39498343 | 0.58356624 | 850 |
| NEFA_20_4 | 0.45490052 | 0.6148535 | 850 |
| NEFA_22_6 | 0.02310889 | 0.10800894 | 921 |
| NEFA_24_1 | 0.58591132 | 0.71310537 | 988 |
| NEFA_12_1 | 0.20079148 | 0.41114446 | 895 |
| NEFA_13_1 | 0.51603071 | 0.64503838 | 528 |
| NEFA_14_2 | 0.01044076 | 0.0706468 | 797 |
| NEFA_16_2 | 0.74589133 | 0.822393 | 865 |
| NEFA_18_4 | 0.70978383 | 0.81172087 | 698 |
| NEFA_19_0 | 0.02275243 | 0.10800894 | 762 |
| NEFA_19_1 | 0.92638575 | 0.96658519 | 861 |
| NEFA_20_5 | 0.24353594 | 0.43633523 | 861 |
| NEFA_22_4 | 0.67503673 | 0.78237493 | 762 |
| NEFA_22_5 | 0.2358459 | 0.42971922 | 861 |
| NEFA_24_4 | 0.12094549 | 0.30236373 | 535 |
| NEFA_24_5 | 0.16571376 | 0.37112977 | 602 |
| NEFA_26_1 | 0.00443211 | 0.04704518 | 714 |

### Glucose

| Analytes | P-VALUE | FDR P-Value | Number of observations |
| --- | --- | --- | --- |
| Carn | 0.54350026 | 0.78720801 | 996 |
| Carn.a.C10.0 | 0.19842684 | 0.63386123 | 622 |
| Carn.a.C10.1 | 0.46343015 | 0.78720801 | 620 |
| Carn.a.C12.0 | 0.52061536 | 0.78720801 | 616 |
| Carn.a.C12.1 | 0.96973745 | 0.99084667 | 549 |
| Carn.a.C14.0 | 0.60470174 | 0.8169584 | 823 |
| Carn.a.C14.1 | 0.43143657 | 0.78720801 | 896 |
| Carn.a.C14.2 | 0.8506203 | 0.97086412 | 887 |
| Carn.a.C16.0 | 0.3509449 | 0.76027691 | 991 |
| Carn.a.C16.1 | 0.97630415 | 0.99084667 | 894 |
| Carn.a.C18.0 | 0.24450899 | 0.66543587 | 796 |
| Carn.a.C18.1 | 0.1566306 | 0.59967778 | 995 |
| Carn.a.C2.0 | 0.22670442 | 0.649886 | 925 |
| Carn.a.C3.0 | 0.65816358 | 0.83238335 | 994 |
| Carn.a.C4.0 | 0.54009496 | 0.78720801 | 861 |
| Carn.a.C4.0.DC | 0.03747665 | 0.40287395 | 546 |
| Carn.a.C5.0 | 0.31693835 | 0.7425279 | 666 |
| Carn.a.C6.0 | 0.77594639 | 0.91663996 | 846 |
| Carn.a.C8.1 | 0.36422568 | 0.76027691 | 748 |
| lyso.PC.a.C14.0 | 0.35751856 | 0.76027691 | 991 |
| lyso.PC.a.C16.0 | 0.20958371 | 0.63386123 | 925 |
| lyso.PC.a.C16.1 | 0.41295868 | 0.78571783 | 996 |
| lyso.PC.a.C18.0 | 0.09381547 | 0.53317056 | 996 |
| lyso.PC.a.C18.1 | 0.21212326 | 0.63386123 | 925 |
| lyso.PC.a.C18.2 | 0.21163393 | 0.63386123 | 925 |
| lyso.PC.a.C18.3 | 0.38017889 | 0.76391085 | 793 |
| lyso.PC.a.C18.6 | 0.75209563 | 0.90662176 | 707 |
| lyso.PC.a.C20.2 | 0.98333293 | 0.99084667 | 645 |
| lyso.PC.a.C20.3 | 0.38802973 | 0.76537975 | 853 |
| lyso.PC.a.C20.4 | 0.18537311 | 0.63386123 | 996 |
| lyso.PC.a.C20.5 | 0.02084077 | 0.34467422 | 920 |
| lyso.PC.a.C22.5 | 0.07020489 | 0.50313505 | 874 |
| lyso.PC.a.C22.6 | 0.09618253 | 0.53317056 | 939 |
| lyso.PC.e.C16.0 | 0.92843666 | 0.97086412 | 995 |
| lyso.PC.e.C18.0 | 0.52684259 | 0.78720801 | 967 |
| lyso.PC.e.C18.1 | 0.8742311 | 0.97086412 | 707 |
| PC.aa.C30.0 | 0.05868508 | 0.46133036 | 988 |
| PC.aa.C30.1 | 0.11679595 | 0.54584272 | 701 |
| PC.aa.C30.2 | 0.62217238 | 0.82065682 | 638 |
| PC.aa.C32.0 | 0.36085123 | 0.76027691 | 996 |
| PC.aa.C32.1 | 0.237813 | 0.66402332 | 864 |
| PC.aa.C32.2 | 0.0120761 | 0.23603292 | 836 |
| PC.aa.C32.3 | 0.03148738 | 0.37609921 | 868 |
| PC.aa.C34.0 | 0.41174524 | 0.78571783 | 706 |
| PC.aa.C34.1 | 0.0859519 | 0.53317056 | 935 |
| PC.aa.C34.2 | 0.00033547 | 0.03606339 | 925 |
| PC.aa.C34.3 | 0.00092941 | 0.04739319 | 925 |
| PC.aa.C34.4 | 0.00837903 | 0.20016574 | 865 |
| PC.aa.C34.5 | 0.04165166 | 0.41024651 | 875 |
| PC.aa.C36.0 | 0.25157975 | 0.66777342 | 924 |
| PC.aa.C36.1 | 0.32423886 | 0.7425279 | 924 |
| PC.aa.C36.2 | 0.05896614 | 0.46133036 | 925 |
| PC.aa.C36.3 | 0.00284337 | 0.10188728 | 936 |
| PC.aa.C36.4 | 0.00110217 | 0.04739319 | 865 |
| PC.aa.C36.5 | 0.2688284 | 0.70000408 | 808 |
| PC.aa.C36.6 | 0.03560944 | 0.40287395 | 983 |
| PC.aa.C38.0 | 0.39707798 | 0.77610697 | 867 |
| PC.aa.C38.1 | 0.24941034 | 0.66777342 | 796 |
| PC.aa.C38.2 | 0.3765835 | 0.76382502 | 936 |
| PC.aa.C38.3 | 0.54152251 | 0.78720801 | 836 |
| PC.aa.C38.4 | 0.30546256 | 0.73791518 | 836 |
| PC.aa.C38.5 | 0.35970614 | 0.76027691 | 836 |
| PC.aa.C38.6 | 0.10090121 | 0.54234402 | 836 |
| PC.aa.C40.0 | 0.92199257 | 0.97086412 | 800 |
| PC.aa.C40.1 | 0.33559171 | 0.75885091 | 573 |
| PC.aa.C40.3 | 0.7734801 | 0.91663996 | 584 |
| PC.aa.C40.4 | 0.28740096 | 0.72597302 | 925 |
| PC.aa.C40.5 | 0.73653327 | 0.89974235 | 836 |
| PC.aa.C40.6 | 0.81341771 | 0.9460072 | 901 |
| PC.aa.C42.0 | 0.17350577 | 0.6322668 | 796 |
| PC.aa.C42.1 | 0.53987515 | 0.78720801 | 643 |
| PC.aa.C42.2 | 0.91502279 | 0.97086412 | 629 |
| PC.aa.C42.4 | 0.47935174 | 0.78720801 | 549 |
| PC.aa.C42.5 | 0.88060563 | 0.97086412 | 731 |
| PC.aa.C42.6 | 0.93403826 | 0.97086412 | 702 |
| PC.aa.C43.4 | 0.05712278 | 0.46133036 | 626 |
| PC.aa.C43.6 | 0.56529944 | 0.78777261 | 925 |
| PC.aa.C44.12 | 0.58796118 | 0.81033111 | 925 |
| PC.ae.C30.0 | 0.65071336 | 0.831506 | 849 |
| PC.ae.C30.1 | 0.38435445 | 0.76515007 | 758 |
| PC.ae.C32.0 | 0.4905426 | 0.78720801 | 996 |
| PC.ae.C32.1 | 0.29597368 | 0.72597302 | 996 |
| PC.ae.C32.2 | 0.94936035 | 0.97661471 | 995 |
| PC.ae.C34.0 | 0.02789512 | 0.35279118 | 935 |
| PC.ae.C34.1 | 0.44286619 | 0.78720801 | 936 |
| PC.ae.C34.2 | 0.09037291 | 0.53317056 | 936 |
| PC.ae.C34.3 | 0.09671466 | 0.53317056 | 936 |
| PC.ae.C34.4 | 0.06008023 | 0.46133036 | 869 |
| PC.ae.C36.0 | 0.85450393 | 0.97086412 | 929 |
| PC.ae.C36.1 | 0.33883576 | 0.75885091 | 925 |
| PC.ae.C36.2 | 0.02745126 | 0.35279118 | 925 |
| PC.ae.C36.3 | 0.04197871 | 0.41024651 | 996 |
| PC.ae.C36.4 | 0.05731184 | 0.46133036 | 865 |
| PC.ae.C36.5 | 0.0477755 | 0.44659711 | 865 |
| PC.ae.C36.6 | 0.14754653 | 0.59853783 | 734 |
| PC.ae.C38.0 | 0.02363131 | 0.35279118 | 925 |
| PC.ae.C38.2 | 0.19622267 | 0.63386123 | 925 |
| PC.ae.C38.3 | 0.11993245 | 0.54584272 | 925 |
| PC.ae.C38.4 | 0.12186256 | 0.54584272 | 865 |
| PC.ae.C38.5 | 0.15898434 | 0.59967778 | 865 |
| PC.ae.C38.6 | 0.1550457 | 0.59967778 | 935 |
| PC.ae.C40.0 | 0.3246401 | 0.7425279 | 925 |
| PC.ae.C40.1 | 0.21690081 | 0.63881746 | 925 |
| PC.ae.C40.2 | 0.37493499 | 0.76382502 | 867 |
| PC.ae.C40.3 | 0.48909159 | 0.78720801 | 868 |
| PC.ae.C40.4 | 0.4778674 | 0.78720801 | 925 |
| PC.ae.C40.5 | 0.93380227 | 0.97086412 | 925 |
| PC.ae.C40.6 | 0.48236 | 0.78720801 | 836 |
| PC.ae.C42.0 | 0.61071622 | 0.8169584 | 870 |
| PC.ae.C42.1 | 0.54553892 | 0.78720801 | 796 |
| PC.ae.C42.2 | 0.48931652 | 0.78720801 | 868 |
| PC.ae.C42.3 | 0.34741729 | 0.76027691 | 933 |
| PC.ae.C42.4 | 0.93851882 | 0.97086412 | 925 |
| PC.ae.C42.5 | 0.53350265 | 0.78720801 | 868 |
| PC.ae.C42.6 | 0.91259584 | 0.97086412 | 994 |
| SM.a.C30.1 | 0.08560011 | 0.53317056 | 859 |
| SM.a.C32.0 | 0.29654109 | 0.72597302 | 564 |
| SM.a.C32.1 | 0.24373563 | 0.66543587 | 925 |
| SM.a.C32.2 | 0.56792909 | 0.78777261 | 936 |
| SM.a.C33.1 | 0.20279503 | 0.63386123 | 925 |
| SM.a.C33.2 | 0.81840623 | 0.9460072 | 803 |
| SM.a.C34.0 | 0.27023413 | 0.70000408 | 628 |
| SM.a.C34.1 | 0.20392242 | 0.63386123 | 939 |
| SM.a.C34.2 | 0.01846357 | 0.33080559 | 996 |
| SM.a.C34.3 | 0.1671035 | 0.6194354 | 643 |
| SM.a.C35.0 | 0.986988 | 0.99084667 | 735 |
| SM.a.C35.1 | 0.91060299 | 0.97086412 | 925 |
| SM.a.C36.0 | 0.36911069 | 0.76306538 | 643 |
| SM.a.C36.1 | 0.53340662 | 0.78720801 | 939 |
| SM.a.C36.2 | 0.18934932 | 0.63386123 | 996 |
| SM.a.C36.3 | 0.10718406 | 0.54584272 | 935 |
| SM.a.C37.1 | 0.50557523 | 0.78720801 | 868 |
| SM.a.C38.1 | 0.00515269 | 0.1582612 | 993 |
| SM.a.C38.2 | 0.0010913 | 0.04739319 | 996 |
| SM.a.C39.1 | 0.11312355 | 0.54584272 | 868 |
| SM.a.C39.2 | 0.02544055 | 0.35279118 | 935 |
| SM.a.C40.1 | 0.55958018 | 0.78720801 | 860 |
| SM.a.C40.2 | 0.14310389 | 0.59853783 | 808 |
| SM.a.C40.3 | 0.12507565 | 0.54797586 | 712 |
| SM.a.C40.4 | 9.8432E-05 | 0.02116283 | 995 |
| SM.a.C40.5 | 0.18159952 | 0.63386123 | 877 |
| SM.a.C41.1 | 0.00934715 | 0.20096376 | 925 |
| SM.a.C41.2 | 0.35690969 | 0.76027691 | 925 |
| SM.a.C42.1 | 0.00670386 | 0.18016619 | 925 |
| SM.a.C42.2 | 0.11116544 | 0.54584272 | 925 |
| SM.a.C42.3 | 0.63931534 | 0.83059324 | 925 |
| SM.a.C42.4 | 0.21226981 | 0.63386123 | 865 |
| SM.a.C42.5 | 0.59866636 | 0.8169584 | 643 |
| SM.a.C42.6 | 0.31461999 | 0.7425279 | 865 |
| SM.a.C43.1 | 0.43262395 | 0.78720801 | 925 |
| SM.a.C43.2 | 0.74328155 | 0.90285613 | 925 |
| SM.a.C44.2 | 0.75481532 | 0.90662176 | 732 |
| SM.a.C44.6 | 0.89968469 | 0.97086412 | 810 |
| SM.a.C31.1 | 0.44636006 | 0.78720801 | 867 |
| SM.a.C33.3 | 0.62124972 | 0.82065682 | 935 |
| SM.a.C35.2 | 0.14674182 | 0.59853783 | 939 |
| SM.a.C37.3 | 0.15278316 | 0.59967778 | 938 |
| SM.a.C39.5 | 0.10342747 | 0.54236355 | 773 |
| SM.a.C41.0 | 0.07300624 | 0.50633363 | 776 |
| SM.a.C41.3 | 0.19667767 | 0.63386123 | 722 |
| SM.a.C43.0 | 0.32414748 | 0.7425279 | 703 |
| SM.a.C43.3 | 0.67271018 | 0.8458052 | 804 |
| Ala | 0.29714245 | 0.72597302 | 993 |
| Arg | 0.07862266 | 0.528246 | 991 |
| Asn | 0.56019919 | 0.78720801 | 919 |
| Asp | 0.46379091 | 0.78720801 | 993 |
| Cit | 0.55503406 | 0.78720801 | 993 |
| Gln | 0.89608969 | 0.97086412 | 919 |
| Glu | 0.51459353 | 0.78720801 | 993 |
| Gly | 0.52940115 | 0.78720801 | 993 |
| His | 0.55406102 | 0.78720801 | 981 |
| Ile | 0.1986045 | 0.63386123 | 717 |
| Leu | 0.71972373 | 0.89863821 | 919 |
| Lys | 0.64807844 | 0.831506 | 919 |
| Met | 0.98852141 | 0.99084667 | 919 |
| Orn | 0.12743625 | 0.54797586 | 919 |
| Phe | 0.99084667 | 0.99084667 | 919 |
| Pro | 0.48735998 | 0.78720801 | 993 |
| Trp | 0.40321011 | 0.78099255 | 993 |
| Ser | 0.77227108 | 0.91663996 | 993 |
| Thr | 0.27634248 | 0.70730516 | 936 |
| Tyr | 0.78115359 | 0.91774875 | 919 |
| Val | 0.91221811 | 0.97086412 | 919 |
| NEFA_12_0 | 0.21992454 | 0.63896994 | 788 |
| NEFA_14_0 | 0.73144971 | 0.89863821 | 964 |
| NEFA_14_1 | 0.54354517 | 0.78720801 | 989 |
| NEFA_15_0 | 0.45219323 | 0.78720801 | 986 |
| NEFA_15_1 | 0.47822192 | 0.78720801 | 681 |
| NEFA_16_0 | 0.61135583 | 0.8169584 | 851 |
| NEFA_16_1 | 0.54246767 | 0.78720801 | 989 |
| NEFA_17_0 | 0.93320653 | 0.97086412 | 917 |
| NEFA_17_1 | 0.65360239 | 0.831506 | 902 |
| NEFA_18_0 | 0.55215539 | 0.78720801 | 839 |
| NEFA_18_1 | 0.64129524 | 0.83059324 | 921 |
| NEFA_18_2 | 0.6335306 | 0.83054316 | 850 |
| NEFA_18_3 | 0.4543347 | 0.78720801 | 988 |
| NEFA_20_1 | 0.83081223 | 0.95521192 | 884 |
| NEFA_20_2 | 0.87234865 | 0.97086412 | 846 |
| NEFA_20_3 | 0.45750809 | 0.78720801 | 850 |
| NEFA_20_4 | 0.48619003 | 0.78720801 | 850 |
| NEFA_22_6 | 0.72695353 | 0.89863821 | 921 |
| NEFA_24_1 | 0.23184868 | 0.65588773 | 988 |
| NEFA_12_1 | 0.88851184 | 0.97086412 | 895 |
| NEFA_13_1 | 0.09278505 | 0.53317056 | 528 |
| NEFA_14_2 | 0.46134443 | 0.78720801 | 797 |
| NEFA_16_2 | 0.61176885 | 0.8169584 | 865 |
| NEFA_18_4 | 0.1149839 | 0.54584272 | 698 |
| NEFA_19_0 | 0.45301266 | 0.78720801 | 762 |
| NEFA_19_1 | 0.93925459 | 0.97086412 | 861 |
| NEFA_20_5 | 0.49221758 | 0.78720801 | 861 |
| NEFA_22_4 | 0.85811819 | 0.97086412 | 762 |
| NEFA_22_5 | 0.81786723 | 0.9460072 | 861 |
| NEFA_24_4 | 0.7310637 | 0.89863821 | 535 |
| NEFA_24_5 | 0.89760707 | 0.97086412 | 602 |
| NEFA_26_1 | 0.06941255 | 0.50313505 | 714 |

### Systolic Blood Pressure

| Analytes | P-VALUE | FDR P-Value | Number of observations |
| --- | --- | --- | --- |
| Carn | 0.84953876 | 0.99864195 | 996 |
| Carn.a.C10.0 | 0.21417809 | 0.99864195 | 622 |
| Carn.a.C10.1 | 0.0747109 | 0.99864195 | 620 |
| Carn.a.C12.0 | 0.99603114 | 0.99864195 | 616 |
| Carn.a.C12.1 | 0.11844498 | 0.99864195 | 549 |
| Carn.a.C14.0 | 0.09714141 | 0.99864195 | 823 |
| Carn.a.C14.1 | 0.24774401 | 0.99864195 | 896 |
| Carn.a.C14.2 | 0.04676774 | 0.99864195 | 887 |
| Carn.a.C16.0 | 0.09617131 | 0.99864195 | 991 |
| Carn.a.C16.1 | 0.03065515 | 0.99864195 | 894 |
| Carn.a.C18.0 | 0.97286654 | 0.99864195 | 796 |
| Carn.a.C18.1 | 0.07439615 | 0.99864195 | 995 |
| Carn.a.C2.0 | 0.065593 | 0.99864195 | 925 |
| Carn.a.C3.0 | 0.18278694 | 0.99864195 | 994 |
| Carn.a.C4.0 | 0.95160942 | 0.99864195 | 861 |
| Carn.a.C4.0.DC | 0.37743518 | 0.99864195 | 546 |
| Carn.a.C5.0 | 0.99286944 | 0.99864195 | 666 |
| Carn.a.C6.0 | 0.53004516 | 0.99864195 | 846 |
| Carn.a.C8.1 | 0.83759294 | 0.99864195 | 748 |
| lyso.PC.a.C14.0 | 0.40232874 | 0.99864195 | 991 |
| lyso.PC.a.C16.0 | 0.32619834 | 0.99864195 | 925 |
| lyso.PC.a.C16.1 | 0.98097008 | 0.99864195 | 996 |
| lyso.PC.a.C18.0 | 0.76074746 | 0.99864195 | 996 |
| lyso.PC.a.C18.1 | 0.28083313 | 0.99864195 | 925 |
| lyso.PC.a.C18.2 | 0.32425845 | 0.99864195 | 925 |
| lyso.PC.a.C18.3 | 0.06342959 | 0.99864195 | 793 |
| lyso.PC.a.C18.6 | 0.05897532 | 0.99864195 | 707 |
| lyso.PC.a.C20.2 | 0.47077329 | 0.99864195 | 645 |
| lyso.PC.a.C20.3 | 0.22860418 | 0.99864195 | 853 |
| lyso.PC.a.C20.4 | 0.37825113 | 0.99864195 | 996 |
| lyso.PC.a.C20.5 | 0.26789465 | 0.99864195 | 920 |
| lyso.PC.a.C22.5 | 0.9942954 | 0.99864195 | 874 |
| lyso.PC.a.C22.6 | 0.34379886 | 0.99864195 | 939 |
| lyso.PC.e.C16.0 | 0.90529676 | 0.99864195 | 995 |
| lyso.PC.e.C18.0 | 0.90115319 | 0.99864195 | 967 |
| lyso.PC.e.C18.1 | 0.85645916 | 0.99864195 | 707 |
| PC.aa.C30.0 | 0.95909051 | 0.99864195 | 988 |
| PC.aa.C30.1 | 0.23709886 | 0.99864195 | 701 |
| PC.aa.C30.2 | 0.30171697 | 0.99864195 | 638 |
| PC.aa.C32.0 | 0.97867993 | 0.99864195 | 996 |
| PC.aa.C32.1 | 0.61499848 | 0.99864195 | 864 |
| PC.aa.C32.2 | 0.14261119 | 0.99864195 | 836 |
| PC.aa.C32.3 | 0.32002119 | 0.99864195 | 868 |
| PC.aa.C34.0 | 0.94006793 | 0.99864195 | 706 |
| PC.aa.C34.1 | 0.90291239 | 0.99864195 | 935 |
| PC.aa.C34.2 | 0.85359242 | 0.99864195 | 925 |
| PC.aa.C34.3 | 0.26002522 | 0.99864195 | 925 |
| PC.aa.C34.4 | 0.13205092 | 0.99864195 | 865 |
| PC.aa.C34.5 | 0.63345491 | 0.99864195 | 875 |
| PC.aa.C36.0 | 0.67039954 | 0.99864195 | 924 |
| PC.aa.C36.1 | 0.78715446 | 0.99864195 | 924 |
| PC.aa.C36.2 | 0.79634125 | 0.99864195 | 925 |
| PC.aa.C36.3 | 0.60131406 | 0.99864195 | 936 |
| PC.aa.C36.4 | 0.51730409 | 0.99864195 | 865 |
| PC.aa.C36.5 | 0.42195463 | 0.99864195 | 808 |
| PC.aa.C36.6 | 0.07027361 | 0.99864195 | 983 |
| PC.aa.C38.0 | 0.73610674 | 0.99864195 | 867 |
| PC.aa.C38.1 | 0.49002277 | 0.99864195 | 796 |
| PC.aa.C38.2 | 0.42066793 | 0.99864195 | 936 |
| PC.aa.C38.3 | 0.53820375 | 0.99864195 | 836 |
| PC.aa.C38.4 | 0.87930177 | 0.99864195 | 836 |
| PC.aa.C38.5 | 0.45134404 | 0.99864195 | 836 |
| PC.aa.C38.6 | 0.83724182 | 0.99864195 | 836 |
| PC.aa.C40.0 | 0.82599724 | 0.99864195 | 800 |
| PC.aa.C40.1 | 0.16240932 | 0.99864195 | 573 |
| PC.aa.C40.3 | 0.99442967 | 0.99864195 | 584 |
| PC.aa.C40.4 | 0.40178008 | 0.99864195 | 925 |
| PC.aa.C40.5 | 0.38086863 | 0.99864195 | 836 |
| PC.aa.C40.6 | 0.8928542 | 0.99864195 | 901 |
| PC.aa.C42.0 | 0.66363288 | 0.99864195 | 796 |
| PC.aa.C42.1 | 0.68051823 | 0.99864195 | 643 |
| PC.aa.C42.2 | 0.61282437 | 0.99864195 | 629 |
| PC.aa.C42.4 | 0.54699278 | 0.99864195 | 549 |
| PC.aa.C42.5 | 0.88688393 | 0.99864195 | 731 |
| PC.aa.C42.6 | 0.98561937 | 0.99864195 | 702 |
| PC.aa.C43.4 | 0.56511962 | 0.99864195 | 626 |
| PC.aa.C43.6 | 0.41635186 | 0.99864195 | 925 |
| PC.aa.C44.12 | 0.21597913 | 0.99864195 | 925 |
| PC.ae.C30.0 | 0.3881719 | 0.99864195 | 849 |
| PC.ae.C30.1 | 0.99577597 | 0.99864195 | 758 |
| PC.ae.C32.0 | 0.79360045 | 0.99864195 | 996 |
| PC.ae.C32.1 | 0.95605362 | 0.99864195 | 996 |
| PC.ae.C32.2 | 0.45300922 | 0.99864195 | 995 |
| PC.ae.C34.0 | 0.67212484 | 0.99864195 | 935 |
| PC.ae.C34.1 | 0.62129945 | 0.99864195 | 936 |
| PC.ae.C34.2 | 0.81655634 | 0.99864195 | 936 |
| PC.ae.C34.3 | 0.74195303 | 0.99864195 | 936 |
| PC.ae.C34.4 | 0.68996806 | 0.99864195 | 869 |
| PC.ae.C36.0 | 0.71262107 | 0.99864195 | 929 |
| PC.ae.C36.1 | 0.6438978 | 0.99864195 | 925 |
| PC.ae.C36.2 | 0.65074373 | 0.99864195 | 925 |
| PC.ae.C36.3 | 0.84931177 | 0.99864195 | 996 |
| PC.ae.C36.4 | 0.55419165 | 0.99864195 | 865 |
| PC.ae.C36.5 | 0.41251169 | 0.99864195 | 865 |
| PC.ae.C36.6 | 0.59518769 | 0.99864195 | 734 |
| PC.ae.C38.0 | 0.80189081 | 0.99864195 | 925 |
| PC.ae.C38.2 | 0.82066283 | 0.99864195 | 925 |
| PC.ae.C38.3 | 0.52257244 | 0.99864195 | 925 |
| PC.ae.C38.4 | 0.48112819 | 0.99864195 | 865 |
| PC.ae.C38.5 | 0.80907855 | 0.99864195 | 865 |
| PC.ae.C38.6 | 0.61455678 | 0.99864195 | 935 |
| PC.ae.C40.0 | 0.9183163 | 0.99864195 | 925 |
| PC.ae.C40.1 | 0.93487689 | 0.99864195 | 925 |
| PC.ae.C40.2 | 0.39888535 | 0.99864195 | 867 |
| PC.ae.C40.3 | 0.72513992 | 0.99864195 | 868 |
| PC.ae.C40.4 | 0.5292463 | 0.99864195 | 925 |
| PC.ae.C40.5 | 0.33576689 | 0.99864195 | 925 |
| PC.ae.C40.6 | 0.89421952 | 0.99864195 | 836 |
| PC.ae.C42.0 | 0.80536068 | 0.99864195 | 870 |
| PC.ae.C42.1 | 0.68737304 | 0.99864195 | 796 |
| PC.ae.C42.2 | 0.64072035 | 0.99864195 | 868 |
| PC.ae.C42.3 | 0.65328192 | 0.99864195 | 933 |
| PC.ae.C42.4 | 0.56061093 | 0.99864195 | 925 |
| PC.ae.C42.5 | 0.31272508 | 0.99864195 | 868 |
| PC.ae.C42.6 | 0.36506338 | 0.99864195 | 994 |
| SM.a.C30.1 | 0.60534231 | 0.99864195 | 859 |
| SM.a.C32.0 | 0.37150691 | 0.99864195 | 564 |
| SM.a.C32.1 | 0.59055025 | 0.99864195 | 925 |
| SM.a.C32.2 | 0.79495047 | 0.99864195 | 936 |
| SM.a.C33.1 | 0.41950747 | 0.99864195 | 925 |
| SM.a.C33.2 | 0.02374247 | 0.99864195 | 803 |
| SM.a.C34.0 | 0.37255016 | 0.99864195 | 628 |
| SM.a.C34.1 | 0.97639408 | 0.99864195 | 939 |
| SM.a.C34.2 | 0.59050364 | 0.99864195 | 996 |
| SM.a.C34.3 | 0.18663346 | 0.99864195 | 643 |
| SM.a.C35.0 | 0.97414732 | 0.99864195 | 735 |
| SM.a.C35.1 | 0.56342451 | 0.99864195 | 925 |
| SM.a.C36.0 | 0.47012458 | 0.99864195 | 643 |
| SM.a.C36.1 | 0.98784778 | 0.99864195 | 939 |
| SM.a.C36.2 | 0.66346798 | 0.99864195 | 996 |
| SM.a.C36.3 | 0.60404112 | 0.99864195 | 935 |
| SM.a.C37.1 | 0.9194044 | 0.99864195 | 868 |
| SM.a.C38.1 | 0.72049171 | 0.99864195 | 993 |
| SM.a.C38.2 | 0.72899918 | 0.99864195 | 996 |
| SM.a.C39.1 | 0.60871334 | 0.99864195 | 868 |
| SM.a.C39.2 | 0.14909622 | 0.99864195 | 935 |
| SM.a.C40.1 | 0.66013911 | 0.99864195 | 860 |
| SM.a.C40.2 | 0.53250597 | 0.99864195 | 808 |
| SM.a.C40.3 | 0.62144185 | 0.99864195 | 712 |
| SM.a.C40.4 | 0.08040176 | 0.99864195 | 995 |
| SM.a.C40.5 | 0.61706484 | 0.99864195 | 877 |
| SM.a.C41.1 | 0.666486 | 0.99864195 | 925 |
| SM.a.C41.2 | 0.55730544 | 0.99864195 | 925 |
| SM.a.C42.1 | 0.69698938 | 0.99864195 | 925 |
| SM.a.C42.2 | 0.83552698 | 0.99864195 | 925 |
| SM.a.C42.3 | 0.47628929 | 0.99864195 | 925 |
| SM.a.C42.4 | 0.7801579 | 0.99864195 | 865 |
| SM.a.C42.5 | 0.80234972 | 0.99864195 | 643 |
| SM.a.C42.6 | 0.78231389 | 0.99864195 | 865 |
| SM.a.C43.1 | 0.50289655 | 0.99864195 | 925 |
| SM.a.C43.2 | 0.61983983 | 0.99864195 | 925 |
| SM.a.C44.2 | 0.92561593 | 0.99864195 | 732 |
| SM.a.C44.6 | 0.94259919 | 0.99864195 | 810 |
| SM.a.C31.1 | 0.30838612 | 0.99864195 | 867 |
| SM.a.C33.3 | 0.4006047 | 0.99864195 | 935 |
| SM.a.C35.2 | 0.29071252 | 0.99864195 | 939 |
| SM.a.C37.3 | 0.36872785 | 0.99864195 | 938 |
| SM.a.C39.5 | 0.74039236 | 0.99864195 | 773 |
| SM.a.C41.0 | 0.03548743 | 0.99864195 | 776 |
| SM.a.C41.3 | 0.66975815 | 0.99864195 | 722 |
| SM.a.C43.0 | 0.6048362 | 0.99864195 | 703 |
| SM.a.C43.3 | 0.78907771 | 0.99864195 | 804 |
| Ala | 0.07766432 | 0.99864195 | 993 |
| Arg | 0.24126232 | 0.99864195 | 991 |
| Asn | 0.0496627 | 0.99864195 | 919 |
| Asp | 0.057378 | 0.99864195 | 993 |
| Cit | 0.95817776 | 0.99864195 | 993 |
| Gln | 0.80058337 | 0.99864195 | 919 |
| Glu | 0.29707936 | 0.99864195 | 993 |
| Gly | 0.91390672 | 0.99864195 | 993 |
| His | 0.21149009 | 0.99864195 | 981 |
| Ile | 0.64889682 | 0.99864195 | 717 |
| Leu | 0.58603961 | 0.99864195 | 919 |
| Lys | 0.99864195 | 0.99864195 | 919 |
| Met | 0.59670826 | 0.99864195 | 919 |
| Orn | 0.69980676 | 0.99864195 | 919 |
| Phe | 0.36389404 | 0.99864195 | 919 |
| Pro | 0.25235692 | 0.99864195 | 993 |
| Trp | 0.48690395 | 0.99864195 | 993 |
| Ser | 0.77467523 | 0.99864195 | 993 |
| Thr | 0.84651017 | 0.99864195 | 936 |
| Tyr | 0.11197306 | 0.99864195 | 919 |
| Val | 0.53419548 | 0.99864195 | 919 |
| NEFA_12_0 | 0.26754836 | 0.99864195 | 788 |
| NEFA_14_0 | 0.69122159 | 0.99864195 | 964 |
| NEFA_14_1 | 0.12414599 | 0.99864195 | 989 |
| NEFA_15_0 | 0.23823326 | 0.99864195 | 986 |
| NEFA_15_1 | 0.53796864 | 0.99864195 | 681 |
| NEFA_16_0 | 0.32733051 | 0.99864195 | 851 |
| NEFA_16_1 | 0.11834472 | 0.99864195 | 989 |
| NEFA_17_0 | 0.60579247 | 0.99864195 | 917 |
| NEFA_17_1 | 0.33343989 | 0.99864195 | 902 |
| NEFA_18_0 | 0.71528025 | 0.99864195 | 839 |
| NEFA_18_1 | 0.27556411 | 0.99864195 | 921 |
| NEFA_18_2 | 0.12450875 | 0.99864195 | 850 |
| NEFA_18_3 | 0.54516137 | 0.99864195 | 988 |
| NEFA_20_1 | 0.39977262 | 0.99864195 | 884 |
| NEFA_20_2 | 0.86885784 | 0.99864195 | 846 |
| NEFA_20_3 | 0.23307172 | 0.99864195 | 850 |
| NEFA_20_4 | 0.36000999 | 0.99864195 | 850 |
| NEFA_22_6 | 0.27377108 | 0.99864195 | 921 |
| NEFA_24_1 | 0.57728438 | 0.99864195 | 988 |
| NEFA_12_1 | 0.09280011 | 0.99864195 | 895 |
| NEFA_13_1 | 0.13197041 | 0.99864195 | 528 |
| NEFA_14_2 | 0.21802899 | 0.99864195 | 797 |
| NEFA_16_2 | 0.13615573 | 0.99864195 | 865 |
| NEFA_18_4 | 0.2288098 | 0.99864195 | 698 |
| NEFA_19_0 | 0.79056296 | 0.99864195 | 762 |
| NEFA_19_1 | 0.62326712 | 0.99864195 | 861 |
| NEFA_20_5 | 0.74567523 | 0.99864195 | 861 |
| NEFA_22_4 | 0.38418449 | 0.99864195 | 762 |
| NEFA_22_5 | 0.35340633 | 0.99864195 | 861 |
| NEFA_24_4 | 0.97053472 | 0.99864195 | 535 |
| NEFA_24_5 | 0.56300254 | 0.99864195 | 602 |
| NEFA_26_1 | 0.99311744 | 0.99864195 | 714 |

## Supplemental Table 6: Results for the group testing after the Anova. MetS Factors as outcome and sex dummy (male versus *nh*female, male versus *h*female, *nh*female vs *h*female) and metabolite interaction as predictor. Adding two dummies at once allows to test for differences between two, rather than one category versus all the others. Metabolites are the 46 significant ones from the anova Models adjusted for ethnicity. P-Value, β-coefficient, FDR p-value and confidence interval reported.

### Supplemental Table 6.1: Results for the testing of *h*females versus *nh*females

**Waist circumference**

| **Analytes** | **P-Values** | **BETA** | **FDR P-Value** | **CI lower** | **CI upper** | **Number of**  **observations** |
| --- | --- | --- | --- | --- | --- | --- |
| lyso.PC.a.C18.1 | 0.0002633 | 4.10094985 | 0.00440216 | 1.76629501 | 6.4356047 | 925 |
| lyso.PC.a.C18.3 | 0.0002871 | 5.11737847 | 0.00440216 | 2.49972979 | 7.73502715 | 793 |
| SM.a.C41.0 | 0.00018425 | -3.73901041 | 0.00440216 | -6.07469747 | -1.40332334 | 776 |
| PC.aa.C34.2 | 0.30488143 | 0.66826293 | 0.51942763 | -1.67879499 | 3.01532086 | 925 |
| PC.aa.C34.3 | 0.75441892 | 0.31290237 | 0.82626835 | -2.05158577 | 2.67739051 | 925 |
| PC.aa.C36.4 | 0.18130063 | 0.67136479 | 0.34749288 | -1.94017223 | 3.28290182 | 865 |
| SM.a.C38.2 | 0.07890059 | -0.16747811 | 0.24196182 | -2.37494341 | 2.03998718 | 996 |
| SM.a.C40.4 | 0.57754374 | 1.79168136 | 0.72219274 | -0.48393876 | 4.06730147 | 995 |
| lyso.PC.a.C16.0 | 0.07001222 | 2.79848239 | 0.24196182 | 0.52925797 | 5.06770682 | 925 |
| lyso.PC.a.C16.1 | 0.12494594 | -0.65093366 | 0.28932364 | -2.68138995 | 1.37952264 | 996 |
| lyso.PC.a.C18.0 | 0.0474519 | 1.61095497 | 0.19843523 | -0.79110984 | 4.01301977 | 996 |
| lyso.PC.a.C20.3 | 0.01789519 | 2.91100056 | 0.12338838 | 0.42428088 | 5.39772024 | 853 |
| lyso.PC.a.C20.4 | 0.02688576 | 3.25753349 | 0.13741611 | 0.91554224 | 5.59952475 | 996 |
| lyso.PC.a.C20.5 | 0.00121832 | 2.44007936 | 0.01401068 | 0.19940711 | 4.68075162 | 920 |
| lyso.PC.a.C22.5 | 0.01877649 | 1.31969156 | 0.12338838 | -1.10018142 | 3.73956455 | 874 |
| lyso.PC.a.C22.6 | 0.05201866 | 3.13801926 | 0.19940485 | 1.04599957 | 5.23003895 | 939 |
| lyso.PC.e.C18.0 | 0.07568354 | 1.87395808 | 0.24196182 | -0.40323511 | 4.15115127 | 967 |
| lyso.PC.e.C18.1 | 0.02510979 | 2.98823771 | 0.13741611 | 0.16201171 | 5.8144637 | 707 |
| PC.aa.C36.0 | 0.51531451 | 1.19171 | 0.69719022 | -0.7147323 | 3.09815231 | 924 |
| PC.aa.C38.3 | 0.12012851 | -1.43781462 | 0.28932364 | -3.63893846 | 0.76330922 | 836 |
| PC.aa.C38.4 | 0.12579289 | -0.76779035 | 0.28932364 | -2.96828676 | 1.43270606 | 836 |
| PC.aa.C38.6 | 0.68608833 | 1.53104892 | 0.80923238 | -1.16973172 | 4.23182955 | 836 |
| PC.aa.C40.6 | 0.15213594 | -0.73523654 | 0.32243423 | -3.10455971 | 1.63408664 | 901 |
| PC.ae.C40.0 | 0.37390129 | 0.41611327 | 0.5657084 | -1.96555931 | 2.79778586 | 925 |
| PC.ae.C40.6 | 0.92572122 | 1.14697525 | 0.93199272 | -1.15738561 | 3.45133611 | 836 |
| SM.a.C36.0 | 0.00355835 | -1.90193128 | 0.03273685 | -4.39947395 | 0.59561139 | 643 |
| SM.a.C42.4 | 0.18967624 | -0.67671011 | 0.34900428 | -2.80060898 | 1.44718876 | 865 |
| SM.a.C42.6 | 0.48601513 | 1.44376663 | 0.67747564 | -1.24478351 | 4.13231677 | 865 |
| SM.a.C44.6 | 0.82515983 | 0.50675069 | 0.88272912 | -1.7519552 | 2.76545657 | 810 |
| NEFA_24_5 | 0.03034168 | -3.78640886 | 0.13957174 | -7.45453024 | -0.11828749 | 602 |
| Carn.a.C3.0 | 0.15420768 | -1.89587367 | 0.32243423 | -4.12336292 | 0.33161558 | 994 |
| PC.aa.C32.3 | 0.71716617 | 0.49218356 | 0.82065782 | -1.57440891 | 2.55877603 | 868 |
| PC.aa.C34.1 | 0.38123827 | 1.11764394 | 0.5657084 | -1.12073446 | 3.35602234 | 935 |
| PC.aa.C36.1 | 0.12292727 | 0.3050118 | 0.28932364 | -1.67305662 | 2.28308023 | 924 |
| PC.aa.C36.2 | 0.58089416 | 0.29135779 | 0.72219274 | -1.76318954 | 2.34590512 | 925 |
| PC.aa.C36.3 | 0.41885935 | 0.61250605 | 0.60211032 | -1.83048543 | 3.05549754 | 936 |
| PC.aa.C38.0 | 0.33431188 | 1.3558909 | 0.54922667 | -0.75285139 | 3.46463318 | 867 |
| PC.aa.C38.5 | 0.90651748 | 0.36109706 | 0.93199272 | -1.83121586 | 2.55340998 | 836 |
| PC.aa.C40.4 | 0.16885618 | -0.99362795 | 0.33771236 | -3.12325319 | 1.13599728 | 925 |
| PC.aa.C40.5 | 0.36477406 | -1.07653537 | 0.5657084 | -3.27398901 | 1.12091827 | 836 |
| PC.ae.C36.1 | 0.63109004 | 0.99178395 | 0.7639511 | -0.96661609 | 2.950184 | 925 |
| PC.ae.C38.0 | 0.93199272 | 0.75520871 | 0.93199272 | -1.31370581 | 2.82412323 | 925 |
| SM.a.C38.1 | 0.73145588 | 0.63592299 | 0.82065782 | -1.55173527 | 2.82358125 | 993 |
| SM.a.C40.3 | 0.55495586 | -0.40005556 | 0.72219274 | -3.44607983 | 2.64596872 | 712 |
| SM.a.C40.5 | 0.12379529 | 2.35996121 | 0.28932364 | 0.21375105 | 4.50617136 | 877 |
| NEFA_26_1 | 0.28516158 | 0.04830982 | 0.50451664 | -2.53966981 | 2.63628944 | 714 |

**HDL**

| **Analytes** | **P-Values** | **BETA** | **FDR P-Value** | **CI lower** | **CI upper** | **Number of observations** |
| --- | --- | --- | --- | --- | --- | --- |
| lyso.PC.a.C18.1 | 4.62E-11 | -0.0867251 | 2.13E-09 | -0.14133404 | -0.03211616 | 925 |
| lyso.PC.a.C18.3 | 5.53E-08 | -0.08893126 | 1.27E-06 | -0.14979094 | -0.02807158 | 793 |
| SM.a.C41.0 | 0.00039013 | 0.03390509 | 0.00224327 | -0.02209566 | 0.08990583 | 776 |
| PC.aa.C34.2 | 0.01816551 | -0.05515674 | 0.03798244 | -0.1059171 | -0.00439639 | 925 |
| PC.aa.C34.3 | 0.07834794 | -0.06064474 | 0.12871448 | -0.11276393 | -0.00852554 | 925 |
| PC.aa.C36.4 | 0.9865078 | -0.03790938 | 0.99332881 | -0.09568506 | 0.0198663 | 865 |
| SM.a.C38.2 | 0.05024917 | -0.04054764 | 0.09631092 | -0.08946336 | 0.00836809 | 996 |
| SM.a.C40.4 | 0.32522471 | -0.05825751 | 0.4400099 | -0.10891655 | -0.00759848 | 995 |
| lyso.PC.a.C16.0 | 0.00021702 | -0.03750629 | 0.00166381 | -0.09084134 | 0.01582875 | 925 |
| lyso.PC.a.C16.1 | 0.00077151 | -0.03889281 | 0.00394329 | -0.08632594 | 0.00854033 | 996 |
| lyso.PC.a.C18.0 | 0.00303523 | -0.02266926 | 0.00997291 | -0.07885516 | 0.03351663 | 996 |
| lyso.PC.a.C20.3 | 0.00029618 | -0.0662185 | 0.00194631 | -0.1238496 | -0.00858741 | 853 |
| lyso.PC.a.C20.4 | 7.74E-06 | -0.0787238 | 7.23E-05 | -0.13313348 | -0.02431413 | 996 |
| lyso.PC.a.C20.5 | 2.23E-06 | -0.0585652 | 3.42E-05 | -0.10993382 | -0.00719657 | 920 |
| lyso.PC.a.C22.5 | 0.00099093 | -0.04185394 | 0.00428743 | -0.09906793 | 0.01536005 | 874 |
| lyso.PC.a.C22.6 | 0.01159311 | -0.10055643 | 0.02715196 | -0.1504239 | -0.05068896 | 939 |
| lyso.PC.e.C18.0 | 0.00136289 | -0.05141913 | 0.00522442 | -0.1047597 | 0.00192144 | 967 |
| lyso.PC.e.C18.1 | 7.86E-06 | -0.09153214 | 7.23E-05 | -0.15892469 | -0.02413958 | 707 |
| PC.aa.C36.0 | 0.98073624 | -0.05627628 | 0.99332881 | -0.09871822 | -0.01383433 | 924 |
| PC.aa.C38.3 | 0.00332038 | 0.00184324 | 0.01018249 | -0.0510574 | 0.05474387 | 836 |
| PC.aa.C38.4 | 0.01232944 | -0.02569531 | 0.02715196 | -0.07618204 | 0.02479143 | 836 |
| PC.aa.C38.6 | 0.8377634 | -0.07162357 | 0.93992967 | -0.13242232 | -0.01082483 | 836 |
| PC.aa.C40.6 | 0.00619387 | -0.02174118 | 0.01780738 | -0.07663438 | 0.03315202 | 901 |
| PC.ae.C40.0 | 0.5168829 | -0.0722637 | 0.59441534 | -0.12602549 | -0.01850191 | 925 |
| PC.ae.C40.6 | 0.50396561 | -0.06278901 | 0.59441534 | -0.11473274 | -0.01084529 | 836 |
| SM.a.C36.0 | 0.00151924 | 0.02512649 | 0.00537579 | -0.03819402 | 0.08844699 | 643 |
| SM.a.C42.4 | 0.01229966 | -0.01953243 | 0.02715196 | -0.06830983 | 0.02924498 | 865 |
| SM.a.C42.6 | 0.90166446 | -0.08495953 | 0.98126784 | -0.1464062 | -0.02351286 | 865 |
| SM.a.C44.6 | 0.0110437 | -0.03019421 | 0.02715196 | -0.08303527 | 0.02264686 | 810 |
| NEFA_24_5 | 0.00102526 | 0.02362259 | 0.00428743 | -0.06510225 | 0.11234742 | 602 |
| Carn.a.C3.0 | 0.42070837 | 0.00712881 | 0.52357401 | -0.04577268 | 0.0600303 | 994 |
| PC.aa.C32.3 | 0.24444794 | -0.02347731 | 0.35139391 | -0.07207626 | 0.02512164 | 868 |
| PC.aa.C34.1 | 0.06045595 | -0.065512 | 0.11123895 | -0.11544851 | -0.01557549 | 935 |
| PC.aa.C36.1 | 0.42113562 | -0.02935705 | 0.52357401 | -0.07484038 | 0.01612627 | 924 |
| PC.aa.C36.2 | 0.22411483 | -0.03418045 | 0.33495558 | -0.07966009 | 0.0112992 | 925 |
| PC.aa.C36.3 | 0.45595756 | -0.04100754 | 0.55194863 | -0.09433915 | 0.01232407 | 936 |
| PC.aa.C38.0 | 0.91727211 | -0.05632118 | 0.98126784 | -0.10504602 | -0.00759635 | 867 |
| PC.aa.C38.5 | 0.99332881 | -0.04976004 | 0.99332881 | -0.09852537 | -0.00099471 | 836 |
| PC.aa.C40.4 | 0.3545582 | -0.02008291 | 0.46599078 | -0.06943685 | 0.02927102 | 925 |
| PC.aa.C40.5 | 0.07516514 | -0.00890155 | 0.12805912 | -0.0608115 | 0.04300839 | 836 |
| PC.ae.C36.1 | 0.22573094 | -0.05269837 | 0.33495558 | -0.09734657 | -0.00805017 | 925 |
| PC.ae.C38.0 | 0.09410905 | -0.05885532 | 0.14927642 | -0.10454477 | -0.01316587 | 925 |
| SM.a.C38.1 | 0.0483214 | -0.0612674 | 0.09631092 | -0.10965778 | -0.01287701 | 993 |
| SM.a.C40.3 | 0.30245137 | -0.05825458 | 0.42159887 | -0.12526761 | 0.00875846 | 712 |
| SM.a.C40.5 | 0.01239546 | -0.07342387 | 0.02715196 | -0.12381442 | -0.02303332 | 877 |
| NEFA_26_1 | 0.06434499 | -0.05438939 | 0.11384113 | -0.1163262 | 0.00754742 | 714 |

**Triacylglycerol**

| **Analytes** | **P-Values** | **BETA** | **FDR P-Value** | **CI lower** | **CI upper** | **Number of observations** |
| --- | --- | --- | --- | --- | --- | --- |
| lyso.PC.a.C18.1 | 0.63309671 | 0.08499625 | 0.72832952 | -0.02039728 | 0.19038977 | 925 |
| lyso.PC.a.C18.3 | 0.38450627 | 0.1689871 | 0.57055769 | 0.05309104 | 0.28488317 | 793 |
| SM.a.C41.0 | 0.84799975 | 0.07324598 | 0.91236842 | -0.03321496 | 0.17970692 | 776 |
| PC.aa.C34.2 | 0.12637914 | 0.14358522 | 0.2768305 | 0.04676306 | 0.24040739 | 925 |
| PC.aa.C34.3 | 0.00749347 | 0.17523792 | 0.04924283 | 0.08036503 | 0.2701108 | 925 |
| PC.aa.C36.4 | 0.02537304 | 0.18002399 | 0.08978152 | 0.07198256 | 0.28806542 | 865 |
| SM.a.C38.2 | 0.41016961 | 0.09266532 | 0.58961882 | 0.00018237 | 0.18514828 | 996 |
| SM.a.C40.4 | 0.0106752 | 0.14999429 | 0.06138238 | 0.05573408 | 0.2442545 | 995 |
| lyso.PC.a.C16.0 | 0.92107759 | 0.12341607 | 0.94990006 | 0.02578373 | 0.2210484 | 925 |
| lyso.PC.a.C16.1 | 0.81176993 | 0.09373688 | 0.91076626 | 0.00811 | 0.17936376 | 996 |
| lyso.PC.a.C18.0 | 0.14723308 | 0.11960055 | 0.29446615 | 0.01768821 | 0.2215129 | 996 |
| lyso.PC.a.C20.3 | 0.37823208 | 0.09846296 | 0.57055769 | -0.0099067 | 0.20683261 | 853 |
| lyso.PC.a.C20.4 | 0.92925006 | 0.07615229 | 0.94990006 | -0.02578392 | 0.1780885 | 996 |
| lyso.PC.a.C20.5 | 0.35843597 | 0.06430838 | 0.5685536 | -0.03250523 | 0.16112199 | 920 |
| lyso.PC.a.C22.5 | 0.99538609 | 0.05065964 | 0.99538609 | -0.05800486 | 0.15932415 | 874 |
| lyso.PC.a.C22.6 | 0.18065208 | 0.04057925 | 0.33239982 | -0.05248229 | 0.13364079 | 939 |
| lyso.PC.e.C18.0 | 0.42986524 | 0.01386012 | 0.59920609 | -0.08468232 | 0.11240256 | 967 |
| lyso.PC.e.C18.1 | 0.63333001 | 0.12203354 | 0.72832952 | -0.00536317 | 0.24943025 | 707 |
| PC.aa.C36.0 | 0.00249055 | 0.03992629 | 0.03892259 | -0.04286135 | 0.12271393 | 924 |
| PC.aa.C38.3 | 0.12597012 | 0.16671947 | 0.2768305 | 0.07713294 | 0.256306 | 836 |
| PC.aa.C38.4 | 0.05509302 | 0.15843523 | 0.18101991 | 0.06681013 | 0.25006033 | 836 |
| PC.aa.C38.6 | 0.0184081 | 0.08723885 | 0.07697935 | -0.02771922 | 0.20219693 | 836 |
| PC.aa.C40.6 | 0.14409537 | 0.0728372 | 0.29446615 | -0.02612446 | 0.17179886 | 901 |
| PC.ae.C40.0 | 0.10789078 | 0.0560332 | 0.27247184 | -0.04530149 | 0.15736788 | 925 |
| PC.ae.C40.6 | 0.08068975 | 0.028398 | 0.21833696 | -0.07297938 | 0.12977538 | 836 |
| SM.a.C36.0 | 0.59001758 | 0.18598155 | 0.7142318 | 0.07056963 | 0.30139347 | 643 |
| SM.a.C42.4 | 0.1597164 | 0.15084971 | 0.3061231 | 0.06082083 | 0.24087859 | 865 |
| SM.a.C42.6 | 0.11254271 | 0.07763277 | 0.27247184 | -0.03785628 | 0.19312182 | 865 |
| SM.a.C44.6 | 0.07267153 | 0.09539568 | 0.21641237 | -0.00349641 | 0.19428777 | 810 |
| NEFA_24_5 | 0.51624181 | -0.13232397 | 0.65964231 | -0.29578974 | 0.03114179 | 602 |
| Carn.a.C3.0 | 0.50778676 | 0.10386546 | 0.65964231 | 0.00898762 | 0.1987433 | 994 |
| PC.aa.C32.3 | 0.00487325 | 0.16747417 | 0.04111145 | 0.07828786 | 0.25666047 | 868 |
| PC.aa.C34.1 | 0.00536236 | 0.17650568 | 0.04111145 | 0.08862596 | 0.2643854 | 935 |
| PC.aa.C36.1 | 0.21986351 | 0.17643478 | 0.38898928 | 0.09618849 | 0.25668107 | 924 |
| PC.aa.C36.2 | 0.85286613 | 0.16443371 | 0.91236842 | 0.08030414 | 0.24856327 | 925 |
| PC.aa.C36.3 | 0.07527387 | 0.17868024 | 0.21641237 | 0.08175053 | 0.27560995 | 936 |
| PC.aa.C38.0 | 0.00338457 | 0.04366715 | 0.03892259 | -0.05076953 | 0.13810382 | 867 |
| PC.aa.C38.5 | 0.01363422 | 0.15635028 | 0.06271742 | 0.06584831 | 0.24685226 | 836 |
| PC.aa.C40.4 | 0.26116082 | 0.13249363 | 0.44494065 | 0.04424079 | 0.22074648 | 925 |
| PC.aa.C40.5 | 0.50896377 | 0.14826344 | 0.65964231 | 0.05767435 | 0.23885252 | 836 |
| PC.ae.C36.1 | 0.35449931 | 0.1279796 | 0.5685536 | 0.0447256 | 0.2112336 | 925 |
| PC.ae.C38.0 | 0.00258787 | 0.13864253 | 0.03892259 | 0.05151065 | 0.22577441 | 925 |
| SM.a.C38.1 | 0.02397894 | 0.15333515 | 0.08978152 | 0.06325509 | 0.24341521 | 993 |
| SM.a.C40.3 | 0.01261094 | 0.20637413 | 0.06271742 | 0.08281419 | 0.32993406 | 712 |
| SM.a.C40.5 | 0.00182137 | 0.17605855 | 0.03892259 | 0.08328367 | 0.26883343 | 877 |
| NEFA_26_1 | 0.54671013 | 0.1646378 | 0.67969367 | 0.05170729 | 0.27756831 | 714 |

**Glucose**

| **Analytes** | **P-Values** | **BETA** | **FDR P-Value** | **CI lower** | **CI upper** | **Number of observations** |
| --- | --- | --- | --- | --- | --- | --- |
| lyso.PC.a.C18.1 | 0.09790958 | 0.03054538 | 0.67994194 | -0.05155629 | 0.11264706 | 925 |
| lyso.PC.a.C18.3 | 0.17115073 | 0.03672946 | 0.67994194 | -0.05362421 | 0.12708314 | 793 |
| SM.a.C41.0 | 0.6649584 | 0.05535571 | 0.83266425 | -0.0262144 | 0.13692581 | 776 |
| PC.aa.C34.2 | 0.52769103 | 0.13088367 | 0.83266425 | 0.05171355 | 0.21005378 | 925 |
| PC.aa.C34.3 | 0.8632197 | 0.10889977 | 0.90245696 | 0.02940322 | 0.18839631 | 925 |
| PC.aa.C36.4 | 0.7074776 | 0.12321911 | 0.83266425 | 0.03446017 | 0.21197805 | 865 |
| SM.a.C38.2 | 0.25413701 | 0.13017382 | 0.81046694 | 0.05498814 | 0.2053595 | 996 |
| SM.a.C40.4 | 0.52876253 | 0.13527266 | 0.83266425 | 0.05778506 | 0.21276027 | 995 |
| lyso.PC.a.C16.0 | 0.16079619 | 0.06893584 | 0.67994194 | -0.00832156 | 0.14619324 | 925 |
| lyso.PC.a.C16.1 | 0.38178004 | -0.00649576 | 0.83266425 | -0.07624782 | 0.06325629 | 996 |
| lyso.PC.a.C18.0 | 0.02966496 | 0.06422868 | 0.45486265 | -0.01791823 | 0.14637559 | 996 |
| lyso.PC.a.C20.3 | 0.17145104 | 0.03403675 | 0.67994194 | -0.0508034 | 0.1188769 | 853 |
| lyso.PC.a.C20.4 | 0.09152517 | 0.02489558 | 0.67994194 | -0.05655073 | 0.10634189 | 996 |
| lyso.PC.a.C20.5 | 0.00847325 | 0.03519201 | 0.38976947 | -0.03955643 | 0.10994046 | 920 |
| lyso.PC.a.C22.5 | 0.17255104 | -0.02357982 | 0.67994194 | -0.10656555 | 0.0594059 | 874 |
| lyso.PC.a.C22.6 | 0.06652937 | 0.00823247 | 0.67994194 | -0.06531655 | 0.08178148 | 939 |
| lyso.PC.e.C18.0 | 0.2642827 | 0.0364627 | 0.81046694 | -0.04200899 | 0.1149344 | 967 |
| lyso.PC.e.C18.1 | 0.67132162 | 0.00452825 | 0.83266425 | -0.09446368 | 0.10352019 | 707 |
| PC.aa.C36.0 | 0.58958703 | 0.05214272 | 0.83266425 | -0.01238596 | 0.11667141 | 924 |
| PC.aa.C38.3 | 0.56793454 | 0.01137141 | 0.83266425 | -0.06657094 | 0.08931376 | 836 |
| PC.aa.C38.4 | 0.68976543 | 0.0530021 | 0.83266425 | -0.0228843 | 0.12888851 | 836 |
| PC.aa.C38.6 | 0.59204278 | 0.08360729 | 0.83266425 | -0.00817517 | 0.17538975 | 836 |
| PC.aa.C40.6 | 0.76025866 | 0.02570166 | 0.83266425 | -0.05697214 | 0.10837545 | 901 |
| PC.ae.C40.0 | 0.63422733 | 0.05444027 | 0.83266425 | -0.02631531 | 0.13519585 | 925 |
| PC.ae.C40.6 | 0.38939607 | 0.04842842 | 0.83266425 | -0.03027356 | 0.1271304 | 836 |
| SM.a.C36.0 | 0.41036519 | 0.06369863 | 0.83266425 | -0.02567543 | 0.15307269 | 643 |
| SM.a.C42.4 | 0.75407773 | 0.05608357 | 0.83266425 | -0.01667765 | 0.12884479 | 865 |
| SM.a.C42.6 | 0.57368172 | 0.06331098 | 0.83266425 | -0.0284052 | 0.15502716 | 865 |
| SM.a.C44.6 | 0.68849642 | -0.01710953 | 0.83266425 | -0.09456571 | 0.06034664 | 810 |
| NEFA_24_5 | 0.64221542 | 0.02248229 | 0.83266425 | -0.096753 | 0.14171758 | 602 |
| Carn.a.C3.0 | 0.7372967 | -0.01687137 | 0.83266425 | -0.09412826 | 0.06038552 | 994 |
| PC.aa.C32.3 | 0.49796739 | 0.05468669 | 0.83266425 | -0.01674485 | 0.12611822 | 868 |
| PC.aa.C34.1 | 0.68912696 | 0.07202816 | 0.83266425 | -0.00482785 | 0.14888418 | 935 |
| PC.aa.C36.1 | 0.1921575 | 0.04761617 | 0.67994194 | -0.01931839 | 0.11455074 | 924 |
| PC.aa.C36.2 | 0.14802523 | 0.0840374 | 0.67994194 | 0.0146849 | 0.1533899 | 925 |
| PC.aa.C36.3 | 0.91130229 | 0.09710197 | 0.91130229 | 0.01338842 | 0.18081553 | 936 |
| PC.aa.C38.0 | 0.18575322 | 0.04112896 | 0.67994194 | -0.03171078 | 0.11396869 | 867 |
| PC.aa.C38.5 | 0.48463523 | 0.05321174 | 0.83266425 | -0.02159305 | 0.12801652 | 836 |
| PC.aa.C40.4 | 0.90488901 | 0.04466213 | 0.91130229 | -0.02791855 | 0.11724281 | 925 |
| PC.aa.C40.5 | 0.43654999 | 0.02295544 | 0.83266425 | -0.0535008 | 0.09941168 | 836 |
| PC.ae.C36.1 | 0.6341794 | 0.04704814 | 0.83266425 | -0.01930392 | 0.1134002 | 925 |
| PC.ae.C38.0 | 0.4795929 | 0.08763475 | 0.83266425 | 0.01778846 | 0.15748103 | 925 |
| SM.a.C38.1 | 0.3625077 | 0.11048289 | 0.83266425 | 0.03587617 | 0.18508961 | 993 |
| SM.a.C40.3 | 0.48420088 | 0.03595853 | 0.83266425 | -0.06243644 | 0.13435351 | 712 |
| SM.a.C40.5 | 0.86116229 | 0.0565296 | 0.90245696 | -0.01931977 | 0.13237896 | 877 |
| NEFA_26_1 | 0.02719859 | 0.04039323 | 0.45486265 | -0.0454873 | 0.12627376 | 714 |

**Systolic blood pressure**

| **Analytes** | **P-Values** | **BETA** | **FDR P-Value** | **CI lower** | **CI upper** | **Number of observations** |
| --- | --- | --- | --- | --- | --- | --- |
| lyso.PC.a.C18.1 | 0.90418004 | 1.30517176 | 0.96194237 | -1.13079101 | 3.74113452 | 925 |
| lyso.PC.a.C18.3 | 0.16549837 | 3.12920467 | 0.96194237 | 0.4857732 | 5.77263614 | 793 |
| SM.a.C41.0 | 0.01064798 | -1.82382746 | 0.48980715 | -4.23841686 | 0.59076195 | 776 |
| PC.aa.C34.2 | 0.81414955 | 0.63429629 | 0.96194237 | -1.72722837 | 2.99582094 | 925 |
| PC.aa.C34.3 | 0.43671728 | 1.86435491 | 0.96194237 | -0.48558884 | 4.21429866 | 925 |
| PC.aa.C36.4 | 0.89888657 | 1.13287962 | 0.96194237 | -1.47718357 | 3.7429428 | 865 |
| SM.a.C38.2 | 0.52531568 | -0.06969687 | 0.96194237 | -2.2553358 | 2.11594206 | 996 |
| SM.a.C40.4 | 0.19101056 | 2.52474893 | 0.96194237 | 0.28514836 | 4.76434949 | 995 |
| lyso.PC.a.C16.0 | 0.50332768 | 1.66633968 | 0.96194237 | -0.62582349 | 3.95850285 | 925 |
| lyso.PC.a.C16.1 | 0.85774264 | 0.18184569 | 0.96194237 | -1.82947169 | 2.19316307 | 996 |
| lyso.PC.a.C18.0 | 0.83323454 | 0.41658764 | 0.96194237 | -1.97415782 | 2.80733311 | 996 |
| lyso.PC.a.C20.3 | 0.20712735 | 2.16746925 | 0.96194237 | -0.32100821 | 4.65594672 | 853 |
| lyso.PC.a.C20.4 | 0.48326772 | 1.64396042 | 0.96194237 | -0.72068501 | 4.00860586 | 996 |
| lyso.PC.a.C20.5 | 0.39822133 | 0.64312381 | 0.96194237 | -1.58478893 | 2.87103655 | 920 |
| lyso.PC.a.C22.5 | 0.92011879 | -0.11777006 | 0.96194237 | -2.61098256 | 2.37544244 | 874 |
| lyso.PC.a.C22.6 | 0.14410482 | 0.95573633 | 0.96194237 | -1.18480045 | 3.0962731 | 939 |
| lyso.PC.e.C18.0 | 0.75309797 | -0.02087754 | 0.96194237 | -2.28688653 | 2.24513146 | 967 |
| lyso.PC.e.C18.1 | 0.81023855 | 0.77127801 | 0.96194237 | -2.11342963 | 3.65598565 | 707 |
| PC.aa.C36.0 | 0.54578241 | 0.86807684 | 0.96194237 | -1.04732643 | 2.78348011 | 924 |
| PC.aa.C38.3 | 0.30398743 | -0.46221287 | 0.96194237 | -2.68993457 | 1.76550884 | 836 |
| PC.aa.C38.4 | 0.67492931 | 0.55147242 | 0.96194237 | -1.64406677 | 2.7470116 | 836 |
| PC.aa.C38.6 | 0.57104455 | 0.74787205 | 0.96194237 | -1.94637394 | 3.44211805 | 836 |
| PC.aa.C40.6 | 0.69610847 | 0.11703695 | 0.96194237 | -2.23739409 | 2.47146799 | 901 |
| PC.ae.C40.0 | 0.72763503 | 0.1239062 | 0.96194237 | -2.27445828 | 2.52227068 | 925 |
| PC.ae.C40.6 | 0.85344045 | -0.51812718 | 0.96194237 | -2.8261463 | 1.78989194 | 836 |
| SM.a.C36.0 | 0.56347864 | 1.61550479 | 0.96194237 | -1.06258011 | 4.29358969 | 643 |
| SM.a.C42.4 | 0.72880579 | -0.254393 | 0.96194237 | -2.38484456 | 1.87605856 | 865 |
| SM.a.C42.6 | 0.98216907 | -0.61278958 | 0.98729003 | -3.31803751 | 2.09245834 | 865 |
| SM.a.C44.6 | 0.91971132 | -0.36464343 | 0.96194237 | -2.64691377 | 1.91762691 | 810 |
| NEFA_24_5 | 0.42660494 | -2.04450443 | 0.96194237 | -5.80459525 | 1.71558639 | 602 |
| Carn.a.C3.0 | 0.11361822 | -0.35215863 | 0.96194237 | -2.58249531 | 1.87817804 | 994 |
| PC.aa.C32.3 | 0.4552316 | 1.60896718 | 0.96194237 | -0.51852531 | 3.73645967 | 868 |
| PC.aa.C34.1 | 0.82101206 | 0.1356842 | 0.96194237 | -2.05942674 | 2.33079515 | 935 |
| PC.aa.C36.1 | 0.51558006 | -0.22453545 | 0.96194237 | -2.2021036 | 1.7530327 | 924 |
| PC.aa.C36.2 | 0.68459733 | 0.14567464 | 0.96194237 | -1.91290593 | 2.20425521 | 925 |
| PC.aa.C36.3 | 0.37468938 | -0.38792461 | 0.96194237 | -2.78880049 | 2.01295128 | 936 |
| PC.aa.C38.0 | 0.74304574 | -0.34993121 | 0.96194237 | -2.52508467 | 1.82522226 | 867 |
| PC.aa.C38.5 | 0.39791913 | 0.14593887 | 0.96194237 | -2.01441301 | 2.30629076 | 836 |
| PC.aa.C40.4 | 0.24954747 | -0.32959146 | 0.96194237 | -2.45565213 | 1.79646921 | 925 |
| PC.aa.C40.5 | 0.27838198 | -0.12309462 | 0.96194237 | -2.32485959 | 2.07867035 | 836 |
| PC.ae.C36.1 | 0.72489604 | -0.47154102 | 0.96194237 | -2.43952681 | 1.49644477 | 925 |
| PC.ae.C38.0 | 0.88944774 | 0.6112458 | 0.96194237 | -1.45232037 | 2.67481196 | 925 |
| SM.a.C38.1 | 0.98729003 | 0.64125766 | 0.98729003 | -1.52416934 | 2.80668466 | 993 |
| SM.a.C40.3 | 0.39132128 | 1.45030051 | 0.96194237 | -1.53475635 | 4.43535736 | 712 |
| SM.a.C40.5 | 0.78926717 | 0.56071994 | 0.96194237 | -1.60821495 | 2.72965483 | 877 |
| NEFA_26_1 | 0.90753311 | -0.11750057 | 0.96194237 | -2.71244687 | 2.47744574 | 714 |

### Supplemental Table 6.2: Results for the testing of males versus *h*females

**Waist Circumference**

| **Analytes** | **P-Values** | **BETA** | **FDR P-Value** | **CI lower** | **CI upper** | **Number of observations** |
| --- | --- | --- | --- | --- | --- | --- |
| lyso.PC.a.C18.1 | 0.45863292 | -0.72527478 | 0.8403022 | -2.64510871 | 1.19455916 | 925 |
| lyso.PC.a.C18.3 | 0.56829962 | -0.6483716 | 0.8403022 | -2.87813498 | 1.58139179 | 793 |
| SM.a.C41.0 | 0.30939315 | 0.98300338 | 0.7144239 | -0.91411418 | 2.88012095 | 776 |
| PC.aa.C34.2 | 0.0369097 | 1.9490703 | 0.24319358 | 0.11868708 | 3.77945353 | 925 |
| PC.aa.C34.3 | 0.92792393 | -0.08225636 | 0.97346202 | -1.86639387 | 1.70188115 | 925 |
| PC.aa.C36.4 | 0.00944622 | 2.4593435 | 0.14484206 | 0.60374198 | 4.31494502 | 865 |
| SM.a.C38.2 | 0.04271622 | 1.94363433 | 0.24561829 | 0.06391739 | 3.82335127 | 996 |
| SM.a.C40.4 | 0.00565453 | 2.4593741 | 0.14484206 | 0.71910283 | 4.19964538 | 995 |
| lyso.PC.a.C16.0 | 0.55623371 | 0.54989212 | 0.8403022 | -1.28340779 | 2.38319203 | 925 |
| lyso.PC.a.C16.1 | 0.0080272 | -2.52067725 | 0.14484206 | -4.38284121 | -0.65851329 | 996 |
| lyso.PC.a.C18.0 | 0.3328171 | -0.90729784 | 0.72670149 | -2.74484067 | 0.93024498 | 996 |
| lyso.PC.a.C20.3 | 0.55146484 | -0.70209946 | 0.8403022 | -3.01503927 | 1.61084036 | 853 |
| lyso.PC.a.C20.4 | 0.87478356 | 0.16761196 | 0.97346202 | -1.91907173 | 2.25429565 | 996 |
| lyso.PC.a.C20.5 | 0.06386121 | -2.10398427 | 0.32640176 | -4.32949182 | 0.12152328 | 920 |
| lyso.PC.a.C22.5 | 0.07168696 | -2.07621696 | 0.32716414 | -4.33595279 | 0.18351886 | 874 |
| lyso.PC.a.C22.6 | 0.63843532 | 0.51316869 | 0.8403022 | -1.62940803 | 2.65574541 | 939 |
| lyso.PC.e.C18.0 | 0.67474165 | -0.40183402 | 0.8403022 | -2.28038565 | 1.4767176 | 967 |
| lyso.PC.e.C18.1 | 0.51276818 | -0.7971967 | 0.8403022 | -3.18727267 | 1.59287926 | 707 |
| PC.aa.C36.0 | 0.63987481 | 0.45062336 | 0.8403022 | -1.43894363 | 2.34019034 | 924 |
| PC.aa.C38.3 | 0.64685072 | 0.41530875 | 0.8403022 | -1.3633618 | 2.1939793 | 836 |
| PC.aa.C38.4 | 0.23431731 | 1.15451741 | 0.65517887 | -0.74950185 | 3.05853667 | 836 |
| PC.aa.C38.6 | 0.03423738 | 2.08237479 | 0.24319358 | 0.15508196 | 4.00966762 | 836 |
| PC.aa.C40.6 | 0.24225472 | 1.06533715 | 0.65517887 | -0.72149633 | 2.85217063 | 901 |
| PC.ae.C40.0 | 0.0941817 | 1.51551428 | 0.36102985 | -0.25966924 | 3.29069779 | 925 |
| PC.ae.C40.6 | 0.18612555 | 1.26608977 | 0.57078501 | -0.611988 | 3.14416754 | 836 |
| SM.a.C36.0 | 0.03700772 | 2.22733301 | 0.24319358 | 0.13467229 | 4.31999373 | 643 |
| SM.a.C42.4 | 0.34755289 | 0.89076116 | 0.72670149 | -0.96943336 | 2.75095569 | 865 |
| SM.a.C42.6 | 0.01291552 | 2.3846538 | 0.14852843 | 0.50594237 | 4.26336523 | 865 |
| SM.a.C44.6 | 0.42941383 | 0.77830048 | 0.8403022 | -1.15409697 | 2.71069793 | 810 |
| NEFA_24_5 | 0.92509482 | 0.10905364 | 0.97346202 | -2.1680211 | 2.38612838 | 602 |
| Carn.a.C3.0 | 0.91890849 | 0.11119126 | 0.97346202 | -2.03147938 | 2.25386189 | 994 |
| PC.aa.C32.3 | 0.93228296 | 0.07863257 | 0.97346202 | -1.73710966 | 1.89437481 | 868 |
| PC.aa.C34.1 | 0.97346202 | 0.03086682 | 0.97346202 | -1.78959554 | 1.85132918 | 935 |
| PC.aa.C36.1 | 0.11081767 | -1.55164633 | 0.39212405 | -3.45957635 | 0.35628369 | 924 |
| PC.aa.C36.2 | 0.31061909 | 0.93824725 | 0.7144239 | -0.87682157 | 2.75331607 | 925 |
| PC.aa.C36.3 | 0.0782349 | 1.63231914 | 0.32716414 | -0.18476617 | 3.44940445 | 936 |
| PC.aa.C38.0 | 0.82047012 | 0.21199086 | 0.9715329 | -1.62088225 | 2.04486397 | 867 |
| PC.aa.C38.5 | 0.82369094 | 0.21551513 | 0.9715329 | -1.68253267 | 2.11356293 | 836 |
| PC.aa.C40.4 | 0.48358837 | 0.61245438 | 0.8403022 | -1.10262321 | 2.32753197 | 925 |
| PC.aa.C40.5 | 0.96792676 | 0.03768935 | 0.97346202 | -1.80160074 | 1.87697944 | 836 |
| PC.ae.C36.1 | 0.67589525 | 0.41231328 | 0.8403022 | -1.52259083 | 2.34721739 | 925 |
| PC.ae.C38.0 | 0.46661847 | 0.65914579 | 0.8403022 | -1.11706681 | 2.43535839 | 925 |
| SM.a.C38.1 | 0.25637434 | 1.040961 | 0.65517887 | -0.757774 | 2.839696 | 993 |
| SM.a.C40.3 | 0.6266538 | 0.52730644 | 0.8403022 | -1.60002835 | 2.65464123 | 712 |
| SM.a.C40.5 | 0.5498992 | 0.54272687 | 0.8403022 | -1.23812549 | 2.32357922 | 877 |
| NEFA_26_1 | 0.18139025 | -1.69042628 | 0.57078501 | -4.17124892 | 0.79039635 | 714 |

**HDL**

| **Analytes** | **P-Values** | **BETA** | **FDR P-Value** | **CI lower** | **CI upper** | **Number of observations** |
| --- | --- | --- | --- | --- | --- | --- |
| lyso.PC.a.C18.1 | 2.68E-07 | 0.11860338 | 1.23E-05 | 0.07369734 | 0.16350942 | 925 |
| lyso.PC.a.C18.3 | 2.13E-05 | 0.11295438 | 0.0001783 | 0.06111294 | 0.16479583 | 793 |
| SM.a.C41.0 | 0.00159261 | -0.07341792 | 0.004884 | -0.11890347 | -0.02793238 | 776 |
| PC.aa.C34.2 | 0.66644942 | 0.00869692 | 0.71294589 | -0.03088919 | 0.04828304 | 925 |
| PC.aa.C34.3 | 0.56320228 | -0.01158837 | 0.64768262 | -0.05091519 | 0.02773846 | 925 |
| PC.aa.C36.4 | 0.07403654 | -0.03740923 | 0.1135227 | -0.07846116 | 0.0036427 | 865 |
| SM.a.C38.2 | 0.58526067 | 0.01158716 | 0.65663392 | -0.03006592 | 0.05324023 | 996 |
| SM.a.C40.4 | 0.10536667 | -0.03199921 | 0.15635055 | -0.07074051 | 0.00674208 | 995 |
| lyso.PC.a.C16.0 | 0.0013309 | 0.07067841 | 0.00437297 | 0.02758918 | 0.11376765 | 925 |
| lyso.PC.a.C16.1 | 0.01020435 | 0.05705529 | 0.02040869 | 0.0135536 | 0.10055698 | 996 |
| lyso.PC.a.C18.0 | 0.00284569 | 0.06552056 | 0.00770011 | 0.02253921 | 0.10850191 | 996 |
| lyso.PC.a.C20.3 | 0.02336344 | 0.06203673 | 0.03980438 | 0.00843308 | 0.11564038 | 853 |
| lyso.PC.a.C20.4 | 0.00688891 | 0.06689474 | 0.01667842 | 0.01841643 | 0.11537305 | 996 |
| lyso.PC.a.C20.5 | 0.00030176 | 0.09431066 | 0.00126192 | 0.04328969 | 0.14533163 | 920 |
| lyso.PC.a.C22.5 | 0.00942191 | 0.07083481 | 0.01998153 | 0.017407 | 0.12426261 | 874 |
| lyso.PC.a.C22.6 | 0.46056782 | -0.01921145 | 0.57259783 | -0.07028405 | 0.03186115 | 939 |
| lyso.PC.e.C18.0 | 0.04568692 | 0.04486473 | 0.07246891 | 0.00086187 | 0.08886759 | 967 |
| lyso.PC.e.C18.1 | 0.00211603 | 0.08954994 | 0.00608357 | 0.03255757 | 0.14654231 | 707 |
| PC.aa.C36.0 | 0.00955639 | -0.05566402 | 0.01998153 | -0.09773028 | -0.01359777 | 924 |
| PC.aa.C38.3 | 0.000164 | -0.08245816 | 0.00075439 | -0.12520578 | -0.03971054 | 836 |
| PC.aa.C38.4 | 1.24E-05 | -0.09787781 | 0.00014211 | -0.14156236 | -0.05419325 | 836 |
| PC.aa.C38.6 | 0.00044711 | -0.07791095 | 0.00171393 | -0.12129729 | -0.03452461 | 836 |
| PC.aa.C40.6 | 1.71E-06 | -0.10160387 | 3.94E-05 | -0.14300177 | -0.06020597 | 901 |
| PC.ae.C40.0 | 0.00810798 | -0.0541757 | 0.01864836 | -0.09424714 | -0.01410426 | 925 |
| PC.ae.C40.6 | 0.00015305 | -0.0820376 | 0.00075439 | -0.12437226 | -0.03970293 | 836 |
| SM.a.C36.0 | 0.00106515 | -0.08883169 | 0.00376898 | -0.14188717 | -0.03577621 | 643 |
| SM.a.C42.4 | 5.39E-05 | -0.08833664 | 0.0003107 | -0.13105782 | -0.04561546 | 865 |
| SM.a.C42.6 | 5.40E-05 | -0.08877292 | 0.0003107 | -0.13171077 | -0.04583507 | 865 |
| SM.a.C44.6 | 8.14E-06 | -0.10342017 | 0.00012488 | -0.14862744 | -0.05821289 | 810 |
| NEFA_24_5 | 2.33E-05 | -0.11960751 | 0.0001783 | -0.17468559 | -0.06452943 | 602 |
| Carn.a.C3.0 | 0.18937696 | 0.03405655 | 0.27222938 | -0.01683055 | 0.08494365 | 994 |
| PC.aa.C32.3 | 0.01205035 | -0.05473663 | 0.02309651 | -0.09743647 | -0.01203679 | 868 |
| PC.aa.C34.1 | 0.5147071 | -0.01348807 | 0.62306649 | -0.05410119 | 0.02712505 | 935 |
| PC.aa.C36.1 | 0.7507007 | -0.00710429 | 0.78482345 | -0.05097486 | 0.03676629 | 924 |
| PC.aa.C36.2 | 0.89745806 | -0.0026391 | 0.89745806 | -0.04281762 | 0.03753943 | 925 |
| PC.aa.C36.3 | 0.31129098 | -0.02047684 | 0.43392076 | -0.06014463 | 0.01919096 | 936 |
| PC.aa.C38.0 | 0.01338671 | -0.05347833 | 0.02463155 | -0.0958289 | -0.01112776 | 867 |
| PC.aa.C38.5 | 0.02036088 | -0.0499906 | 0.03602309 | -0.09221037 | -0.00777084 | 836 |
| PC.aa.C40.4 | 0.02613031 | -0.04512038 | 0.04292836 | -0.0848672 | -0.00537355 | 925 |
| PC.aa.C40.5 | 0.0063061 | -0.06061737 | 0.01611559 | -0.1040665 | -0.01716824 | 836 |
| PC.ae.C36.1 | 0.38932088 | -0.01935861 | 0.49772418 | -0.06347114 | 0.02475392 | 925 |
| PC.ae.C38.0 | 0.38952327 | -0.0172066 | 0.49772418 | -0.05643208 | 0.02201888 | 925 |
| SM.a.C38.1 | 0.63348813 | -0.00967069 | 0.69382033 | -0.0494582 | 0.03011682 | 993 |
| SM.a.C40.3 | 0.34324178 | -0.02260826 | 0.46438594 | -0.06940997 | 0.02419345 | 712 |
| SM.a.C40.5 | 0.85036474 | -0.00402018 | 0.86926173 | -0.04583255 | 0.03779219 | 877 |
| NEFA_26_1 | 0.55880116 | 0.01768779 | 0.64768262 | -0.04168449 | 0.07706007 | 714 |

**Triacylglycerol**

| **Analytes** | **P-Values** | **BETA** | **FDR P-Value** | **CI lower** | **CI upper** | **Number of observations** |
| --- | --- | --- | --- | --- | --- | --- |
| lyso.PC.a.C18.1 | 0.01039044 | 0.11339743 | 0.04345092 | 0.0267302 | 0.20006465 | 925 |
| lyso.PC.a.C18.3 | 0.03203564 | 0.10801654 | 0.09210247 | 0.00929405 | 0.20673903 | 793 |
| SM.a.C41.0 | 0.15790516 | 0.06226474 | 0.27937067 | -0.02420614 | 0.14873562 | 776 |
| PC.aa.C34.2 | 0.09225892 | 0.06484324 | 0.21219551 | -0.01066476 | 0.14035125 | 925 |
| PC.aa.C34.3 | 0.27987541 | 0.03943968 | 0.39645784 | -0.03214718 | 0.11102653 | 925 |
| PC.aa.C36.4 | 0.1511463 | 0.05619616 | 0.2781092 | -0.0205716 | 0.13296391 | 865 |
| SM.a.C38.2 | 0.20202158 | 0.05123373 | 0.33189259 | -0.02751803 | 0.1299855 | 996 |
| SM.a.C40.4 | 0.53108713 | 0.02301619 | 0.6107502 | -0.04906892 | 0.0951013 | 995 |
| lyso.PC.a.C16.0 | 0.0014105 | 0.12870164 | 0.02103072 | 0.04982475 | 0.20757852 | 925 |
| lyso.PC.a.C16.1 | 0.04194262 | 0.08150586 | 0.11349179 | 0.00297607 | 0.16003564 | 996 |
| lyso.PC.a.C18.0 | 7.66E-07 | 0.19768487 | 3.52E-05 | 0.11972349 | 0.27564626 | 996 |
| lyso.PC.a.C20.3 | 0.43682826 | 0.03994987 | 0.55419549 | -0.06084657 | 0.14074631 | 853 |
| lyso.PC.a.C20.4 | 0.12660023 | 0.07076346 | 0.25320046 | -0.02006038 | 0.16158729 | 996 |
| lyso.PC.a.C20.5 | 0.85891951 | 0.00871153 | 0.91884413 | -0.08744685 | 0.10486991 | 920 |
| lyso.PC.a.C22.5 | 0.32386565 | 0.0510343 | 0.43817117 | -0.05043924 | 0.15250784 | 874 |
| lyso.PC.a.C22.6 | 0.4123458 | -0.03983057 | 0.5419402 | -0.1351411 | 0.05547995 | 939 |
| lyso.PC.e.C18.0 | 0.16470954 | 0.05759849 | 0.28061626 | -0.02369328 | 0.13889025 | 967 |
| lyso.PC.e.C18.1 | 0.00403328 | 0.15831553 | 0.02319136 | 0.05057899 | 0.26605208 | 707 |
| PC.aa.C36.0 | 0.00863388 | -0.11003974 | 0.03971586 | -0.19209457 | -0.02798491 | 924 |
| PC.aa.C38.3 | 0.01236375 | 0.09246524 | 0.04739438 | 0.02007271 | 0.16485777 | 836 |
| PC.aa.C38.4 | 0.15063777 | 0.05810672 | 0.2781092 | -0.02117356 | 0.13738699 | 836 |
| PC.aa.C38.6 | 0.2332601 | -0.04985512 | 0.36999878 | -0.13188987 | 0.03217963 | 836 |
| PC.aa.C40.6 | 0.9188152 | -0.00387703 | 0.93923331 | -0.07850932 | 0.07075526 | 901 |
| PC.ae.C40.0 | 0.45781366 | -0.02858578 | 0.55419549 | -0.10411575 | 0.04694419 | 925 |
| PC.ae.C40.6 | 0.09731572 | -0.06987143 | 0.21316776 | -0.15249503 | 0.01275218 | 836 |
| SM.a.C36.0 | 0.00228595 | 0.15082358 | 0.02103072 | 0.05412133 | 0.24752583 | 643 |
| SM.a.C42.4 | 0.04782608 | 0.07961482 | 0.12222221 | 0.00076397 | 0.15846567 | 865 |
| SM.a.C42.6 | 0.72473455 | -0.01448362 | 0.79375689 | -0.09518533 | 0.06621809 | 865 |
| SM.a.C44.6 | 0.97599168 | -0.00129753 | 0.97599168 | -0.08590298 | 0.08330792 | 810 |
| NEFA_24_5 | 0.12031239 | -0.08038081 | 0.25156228 | -0.18185613 | 0.0210945 | 602 |
| Carn.a.C3.0 | 0.00207568 | 0.14358649 | 0.02103072 | 0.05232142 | 0.23485156 | 994 |
| PC.aa.C32.3 | 0.47620575 | 0.02845534 | 0.56167858 | -0.04990522 | 0.1068159 | 868 |
| PC.aa.C34.1 | 0.2655737 | 0.04056968 | 0.3940771 | -0.03090247 | 0.11204184 | 935 |
| PC.aa.C36.1 | 0.00320626 | 0.11654344 | 0.02106969 | 0.03914253 | 0.19394436 | 924 |
| PC.aa.C36.2 | 4.37E-05 | 0.15553584 | 0.00100412 | 0.08121244 | 0.22985924 | 925 |
| PC.aa.C36.3 | 0.01492929 | 0.08958651 | 0.0528267 | 0.01749066 | 0.16168237 | 936 |
| PC.aa.C38.0 | 0.00745451 | -0.11217063 | 0.03810082 | -0.19425293 | -0.03008832 | 867 |
| PC.aa.C38.5 | 0.45440389 | 0.02987721 | 0.55419549 | -0.04847706 | 0.10823149 | 836 |
| PC.aa.C40.4 | 0.03119571 | 0.07814768 | 0.09210247 | 0.00707392 | 0.14922144 | 925 |
| PC.aa.C40.5 | 0.00304775 | 0.11479426 | 0.02106969 | 0.03897031 | 0.1906182 | 836 |
| PC.ae.C36.1 | 0.05516441 | 0.08047275 | 0.13355595 | -0.00178241 | 0.16272791 | 925 |
| PC.ae.C38.0 | 0.9051254 | -0.00454429 | 0.93923331 | -0.07934909 | 0.07026052 | 925 |
| SM.a.C38.1 | 0.24948914 | 0.04348999 | 0.38255001 | -0.03057557 | 0.11755556 | 993 |
| SM.a.C40.3 | 0.2844154 | 0.04708516 | 0.39645784 | -0.03920875 | 0.13337908 | 712 |
| SM.a.C40.5 | 0.67261088 | 0.01657969 | 0.75463659 | -0.06040175 | 0.09356114 | 877 |
| NEFA_26_1 | 0.02741329 | 0.12186489 | 0.09007225 | 0.01361034 | 0.23011945 | 714 |

**Glucose**

| **Analytes** | **P-Values** | **BETA** | **FDR P-Value** | **CI lower** | **CI upper** | **Number of observations** |
| --- | --- | --- | --- | --- | --- | --- |
| lyso.PC.a.C18.1 | 0.17947831 | -0.04621399 | 0.37296471 | -0.11372786 | 0.02129988 | 925 |
| lyso.PC.a.C18.3 | 0.33148511 | -0.03809959 | 0.52875353 | -0.11506459 | 0.0388654 | 793 |
| SM.a.C41.0 | 0.02785832 | 0.07436746 | 0.11649844 | 0.00811369 | 0.14062124 | 776 |
| PC.aa.C34.2 | 0.0009517 | 0.10429573 | 0.01141005 | 0.0425539 | 0.16603756 | 925 |
| PC.aa.C34.3 | 0.00092392 | 0.10158315 | 0.01141005 | 0.0415986 | 0.1615677 | 925 |
| PC.aa.C36.4 | 0.00099218 | 0.10616881 | 0.01141005 | 0.04310203 | 0.16923559 | 865 |
| SM.a.C38.2 | 0.01060683 | 0.08352927 | 0.07262176 | 0.01950661 | 0.14755193 | 996 |
| SM.a.C40.4 | 0.0003001 | 0.10955977 | 0.01141005 | 0.05030144 | 0.1688181 | 995 |
| lyso.PC.a.C16.0 | 0.76039229 | 0.00970187 | 0.81344292 | -0.05271416 | 0.0721179 | 925 |
| lyso.PC.a.C16.1 | 0.18648235 | -0.04309476 | 0.37296471 | -0.10706549 | 0.02087597 | 996 |
| lyso.PC.a.C18.0 | 0.34483926 | -0.03026504 | 0.52875353 | -0.09310617 | 0.03257608 | 996 |
| lyso.PC.a.C20.3 | 0.35662298 | -0.03708106 | 0.52918249 | -0.11599232 | 0.04183019 | 853 |
| lyso.PC.a.C20.4 | 0.12360145 | -0.056991 | 0.30273264 | -0.1295586 | 0.0155766 | 996 |
| lyso.PC.a.C20.5 | 0.02012936 | -0.08806698 | 0.09780506 | -0.16230954 | -0.01382443 | 920 |
| lyso.PC.a.C22.5 | 0.02126197 | -0.09110757 | 0.09780506 | -0.16860164 | -0.0136135 | 874 |
| lyso.PC.a.C22.6 | 0.04008854 | -0.07890363 | 0.14706616 | -0.15423008 | -0.00357718 | 939 |
| lyso.PC.e.C18.0 | 0.69811213 | -0.01279843 | 0.80294968 | -0.07753301 | 0.05193614 | 967 |
| lyso.PC.e.C18.1 | 0.63001005 | -0.02054869 | 0.78325573 | -0.10426395 | 0.06316657 | 707 |
| PC.aa.C36.0 | 0.33638938 | 0.03134493 | 0.52875353 | -0.03261256 | 0.09530242 | 924 |
| PC.aa.C38.3 | 0.26933675 | 0.03546781 | 0.47651886 | -0.02751536 | 0.09845098 | 836 |
| PC.aa.C38.4 | 0.28582422 | 0.03572809 | 0.48695978 | -0.02993399 | 0.10139017 | 836 |
| PC.aa.C38.6 | 0.07859245 | 0.05876464 | 0.22595329 | -0.00673186 | 0.12426114 | 836 |
| PC.aa.C40.6 | 0.69821712 | 0.01232135 | 0.80294968 | -0.05002738 | 0.07467009 | 901 |
| PC.ae.C40.0 | 0.2609753 | 0.03449699 | 0.47651886 | -0.02569432 | 0.09468829 | 925 |
| PC.ae.C40.6 | 0.73968328 | 0.01086191 | 0.81344292 | -0.05328101 | 0.07500484 | 836 |
| SM.a.C36.0 | 0.56255706 | 0.02209349 | 0.71882291 | -0.05279195 | 0.09697893 | 643 |
| SM.a.C42.4 | 0.18305994 | 0.04326338 | 0.37296471 | -0.02046378 | 0.10699053 | 865 |
| SM.a.C42.6 | 0.25250307 | 0.03739 | 0.47651886 | -0.02669965 | 0.10147965 | 865 |
| SM.a.C44.6 | 0.99503492 | -0.00021014 | 0.99503492 | -0.06647646 | 0.06605617 | 810 |
| NEFA_24_5 | 0.90221438 | -0.00463252 | 0.92226359 | -0.07865071 | 0.06938566 | 602 |
| Carn.a.C3.0 | 0.38013372 | -0.03325157 | 0.54496533 | -0.10756666 | 0.04106352 | 994 |
| PC.aa.C32.3 | 0.01105114 | 0.08143095 | 0.07262176 | 0.01867003 | 0.14419188 | 868 |
| PC.aa.C34.1 | 0.08462601 | 0.05498292 | 0.22898804 | -0.0075237 | 0.11748955 | 935 |
| PC.aa.C36.1 | 0.86745613 | -0.00549166 | 0.90688595 | -0.07005285 | 0.05906954 | 924 |
| PC.aa.C36.2 | 0.39095339 | 0.02679506 | 0.54496533 | -0.03447369 | 0.08806382 | 925 |
| PC.aa.C36.3 | 0.00136227 | 0.10191659 | 0.01253292 | 0.03965085 | 0.16418233 | 936 |
| PC.aa.C38.0 | 0.68615029 | -0.01303878 | 0.80294968 | -0.0763495 | 0.05027194 | 867 |
| PC.aa.C38.5 | 0.47381137 | 0.023645 | 0.62272351 | -0.04111905 | 0.08840905 | 836 |
| PC.aa.C40.4 | 0.18057037 | 0.03991093 | 0.37296471 | -0.01854137 | 0.09836323 | 925 |
| PC.aa.C40.5 | 0.75161218 | -0.01032282 | 0.81344292 | -0.07431743 | 0.0536718 | 836 |
| PC.ae.C36.1 | 0.40899374 | 0.02759272 | 0.55334447 | -0.03796328 | 0.09314872 | 925 |
| PC.ae.C38.0 | 0.04702653 | 0.06076485 | 0.15451573 | 0.00080015 | 0.12072956 | 925 |
| SM.a.C38.1 | 0.01838605 | 0.07382522 | 0.09780506 | 0.01248213 | 0.13516831 | 993 |
| SM.a.C40.3 | 0.04156217 | 0.07145842 | 0.14706616 | 0.00273965 | 0.1401772 | 712 |
| SM.a.C40.5 | 0.12504174 | 0.04923626 | 0.30273264 | -0.01370097 | 0.11217349 | 877 |
| NEFA_26_1 | 0.0599739 | -0.07899876 | 0.18391996 | -0.16132336 | 0.00332583 | 714 |

**Systolic blood pressure**

| **Analytes** | **P-Values** | **BETA** | **FDR P-Value** | **CI lower** | **CI upper** | **Number of observations** |
| --- | --- | --- | --- | --- | --- | --- |
| lyso.PC.a.C18.1 | 0.149954 | 1.47070077 | 0.91474584 | -0.53244072 | 3.47384226 | 925 |
| lyso.PC.a.C18.3 | 0.42750109 | 0.91066062 | 0.91474584 | -1.34106504 | 3.16238628 | 793 |
| SM.a.C41.0 | 0.13315607 | 1.50194665 | 0.91474584 | -0.45925785 | 3.46315115 | 776 |
| PC.aa.C34.2 | 0.71790162 | 0.33911642 | 0.96161725 | -1.50254887 | 2.18078171 | 925 |
| PC.aa.C34.3 | 0.32605448 | 0.88779401 | 0.91474584 | -0.88536895 | 2.66095697 | 925 |
| PC.aa.C36.4 | 0.30835594 | 0.96310398 | 0.91474584 | -0.89145031 | 2.81765827 | 865 |
| SM.a.C38.2 | 0.47005359 | 0.68538883 | 0.91474584 | -1.17574236 | 2.54652003 | 996 |
| SM.a.C40.4 | 0.26105205 | 0.98149221 | 0.91474584 | -0.73123325 | 2.69421768 | 995 |
| lyso.PC.a.C16.0 | 0.38044385 | 0.82799638 | 0.91474584 | -1.02383569 | 2.67982844 | 925 |
| lyso.PC.a.C16.1 | 0.97081448 | -0.03440006 | 0.98121166 | -1.87901142 | 1.8102113 | 996 |
| lyso.PC.a.C18.0 | 0.46411488 | 0.6825557 | 0.91474584 | -1.146328 | 2.5114394 | 996 |
| lyso.PC.a.C20.3 | 0.83651749 | 0.24341074 | 0.98121166 | -2.07116403 | 2.55798551 | 853 |
| lyso.PC.a.C20.4 | 0.54083392 | 0.65681345 | 0.92142074 | -1.45005483 | 2.76368172 | 996 |
| lyso.PC.a.C20.5 | 0.10683152 | 1.82003462 | 0.91474584 | -0.39279977 | 4.03286901 | 920 |
| lyso.PC.a.C22.5 | 0.97895161 | 0.03130575 | 0.98121166 | -2.29691628 | 2.35952779 | 874 |
| lyso.PC.a.C22.6 | 0.34181111 | -1.06242238 | 0.91474584 | -3.25468867 | 1.12984392 | 939 |
| lyso.PC.e.C18.0 | 0.65816299 | -0.42158892 | 0.9461093 | -2.29091423 | 1.4477364 | 967 |
| lyso.PC.e.C18.1 | 0.77347474 | 0.35777928 | 0.96161725 | -2.08175331 | 2.79731186 | 707 |
| PC.aa.C36.0 | 0.855078 | 0.17672703 | 0.98121166 | -1.7217216 | 2.07517566 | 924 |
| PC.aa.C38.3 | 0.39670686 | 0.77768372 | 0.91474584 | -1.02247986 | 2.57784731 | 836 |
| PC.aa.C38.4 | 0.97825796 | 0.02638483 | 0.98121166 | -1.8733451 | 1.92611477 | 836 |
| PC.aa.C38.6 | 0.98121166 | -0.02307452 | 0.98121166 | -1.94570419 | 1.89955516 | 836 |
| PC.aa.C40.6 | 0.68207894 | -0.37071541 | 0.95077671 | -2.14631793 | 1.40488712 | 901 |
| PC.ae.C40.0 | 0.73397944 | -0.30964042 | 0.96161725 | -2.09726527 | 1.47798443 | 925 |
| PC.ae.C40.6 | 0.76884902 | -0.28173014 | 0.96161725 | -2.16278943 | 1.59932916 | 836 |
| SM.a.C36.0 | 0.51703026 | 0.74080752 | 0.91474584 | -1.50312734 | 2.98474239 | 643 |
| SM.a.C42.4 | 0.48125046 | -0.66985009 | 0.91474584 | -2.53578373 | 1.19608355 | 865 |
| SM.a.C42.6 | 0.50445751 | -0.64315743 | 0.91474584 | -2.53353697 | 1.24722212 | 865 |
| SM.a.C44.6 | 0.80983209 | -0.23945082 | 0.98032306 | -2.19200844 | 1.7131068 | 810 |
| NEFA_24_5 | 0.6250767 | -0.58108488 | 0.9461093 | -2.91525187 | 1.75308212 | 602 |
| Carn.a.C3.0 | 0.08588135 | 1.87961857 | 0.91474584 | -0.26579107 | 4.02502821 | 994 |
| PC.aa.C32.3 | 0.44279823 | 0.73126351 | 0.91474584 | -1.13798649 | 2.6005135 | 868 |
| PC.aa.C34.1 | 0.65150906 | 0.41100661 | 0.9461093 | -1.37426654 | 2.19627976 | 935 |
| PC.aa.C36.1 | 0.56645112 | 0.55739043 | 0.93059827 | -1.35005706 | 2.46483791 | 924 |
| PC.aa.C36.2 | 0.50187234 | 0.6225462 | 0.91474584 | -1.19608575 | 2.44117815 | 925 |
| PC.aa.C36.3 | 0.43368998 | 0.71268845 | 0.91474584 | -1.07307149 | 2.49844838 | 936 |
| PC.aa.C38.0 | 0.43612944 | -0.75047747 | 0.91474584 | -2.64107374 | 1.14011879 | 867 |
| PC.aa.C38.5 | 0.21638903 | 1.17885916 | 0.91474584 | -0.6915176 | 3.04923593 | 836 |
| PC.aa.C40.4 | 0.24634337 | 1.01204457 | 0.91474584 | -0.70016233 | 2.72425146 | 925 |
| PC.aa.C40.5 | 0.19699683 | 1.21229933 | 0.91474584 | -0.63059938 | 3.05519805 | 836 |
| PC.ae.C36.1 | 0.36481077 | -0.89828854 | 0.91474584 | -2.84266338 | 1.04608631 | 925 |
| PC.ae.C38.0 | 0.61421895 | 0.45517733 | 0.9461093 | -1.31644358 | 2.22679823 | 925 |
| SM.a.C38.1 | 0.46722179 | 0.6598672 | 0.91474584 | -1.12058882 | 2.44032323 | 993 |
| SM.a.C40.3 | 0.90223131 | 0.13048724 | 0.98121166 | -1.95426808 | 2.21524256 | 712 |
| SM.a.C40.5 | 0.33779179 | 0.87942361 | 0.91474584 | -0.92028496 | 2.67913218 | 877 |
| NEFA_26_1 | 0.95476849 | 0.07188841 | 0.98121166 | -2.41561245 | 2.55938927 | 714 |

### Supplemental Table 6.3: Results for the testing of males versus *nh*females

**Waist Circumference**

| **Analytes** | **P-Values** | **BETA** | **FDR P-Value** | **CI lower** | **CI upper** | **Number of observations** |
| --- | --- | --- | --- | --- | --- | --- |
| lyso.PC.a.C18.1 | 0.00059186 | 4.10094985 | 0.01361287 | 1.76629501 | 6.4356047 | 925 |
| lyso.PC.a.C18.3 | 0.00013425 | 5.11737847 | 0.00617528 | 2.49972979 | 7.73502715 | 793 |
| SM.a.C41.0 | 0.00173942 | -3.73901041 | 0.0266711 | -6.07469747 | -1.40332334 | 776 |
| PC.aa.C34.2 | 0.57644483 | 0.66826293 | 0.77989595 | -1.67879499 | 3.01532086 | 925 |
| PC.aa.C34.3 | 0.79514382 | 0.31290237 | 0.83280015 | -2.05158577 | 2.67739051 | 925 |
| PC.aa.C36.4 | 0.61398872 | 0.67136479 | 0.79580699 | -1.94017223 | 3.28290182 | 865 |
| SM.a.C38.2 | 0.88167659 | -0.16747811 | 0.9012694 | -2.37494341 | 2.03998718 | 996 |
| SM.a.C40.4 | 0.12265533 | 1.79168136 | 0.40301037 | -0.48393876 | 4.06730147 | 995 |
| lyso.PC.a.C16.0 | 0.01570163 | 2.79848239 | 0.12037919 | 0.52925797 | 5.06770682 | 925 |
| lyso.PC.a.C16.1 | 0.52942574 | -0.65093366 | 0.77989595 | -2.68138995 | 1.37952264 | 996 |
| lyso.PC.a.C18.0 | 0.18845591 | 1.61095497 | 0.52973918 | -0.79110984 | 4.01301977 | 996 |
| lyso.PC.a.C20.3 | 0.02182454 | 2.91100056 | 0.1434184 | 0.42428088 | 5.39772024 | 853 |
| lyso.PC.a.C20.4 | 0.00645561 | 3.25753349 | 0.0593916 | 0.91554224 | 5.59952475 | 996 |
| lyso.PC.a.C20.5 | 0.032845 | 2.44007936 | 0.16787447 | 0.19940711 | 4.68075162 | 920 |
| lyso.PC.a.C22.5 | 0.28474987 | 1.31969156 | 0.59540733 | -1.10018142 | 3.73956455 | 874 |
| lyso.PC.a.C22.6 | 0.00332299 | 3.13801926 | 0.03821438 | 1.04599957 | 5.23003895 | 939 |
| lyso.PC.e.C18.0 | 0.10665261 | 1.87395808 | 0.37738617 | -0.40323511 | 4.15115127 | 967 |
| lyso.PC.e.C18.1 | 0.03826703 | 2.98823771 | 0.17602833 | 0.16201171 | 5.8144637 | 707 |
| PC.aa.C36.0 | 0.22021814 | 1.19171 | 0.5331597 | -0.7147323 | 3.09815231 | 924 |
| PC.aa.C38.3 | 0.20014618 | -1.43781462 | 0.52973918 | -3.63893846 | 0.76330922 | 836 |
| PC.aa.C38.4 | 0.49362091 | -0.76779035 | 0.77989595 | -2.96828676 | 1.43270606 | 836 |
| PC.aa.C38.6 | 0.26615513 | 1.53104892 | 0.59540733 | -1.16973172 | 4.23182955 | 836 |
| PC.aa.C40.6 | 0.54265877 | -0.73523654 | 0.77989595 | -3.10455971 | 1.63408664 | 901 |
| PC.ae.C40.0 | 0.73176224 | 0.41611327 | 0.83280015 | -1.96555931 | 2.79778586 | 925 |
| PC.ae.C40.6 | 0.3288635 | 1.14697525 | 0.59540733 | -1.15738561 | 3.45133611 | 836 |
| SM.a.C36.0 | 0.1353062 | -1.90193128 | 0.41493901 | -4.39947395 | 0.59561139 | 643 |
| SM.a.C42.4 | 0.5319012 | -0.67671011 | 0.77989595 | -2.80060898 | 1.44718876 | 865 |
| SM.a.C42.6 | 0.29218029 | 1.44376663 | 0.59540733 | -1.24478351 | 4.13231677 | 865 |
| SM.a.C44.6 | 0.6597729 | 0.50675069 | 0.79867245 | -1.7519552 | 2.76545657 | 810 |
| NEFA_24_5 | 0.04307788 | -3.78640886 | 0.18014384 | -7.45453024 | -0.11828749 | 602 |
| Carn.a.C3.0 | 0.09519208 | -1.89587367 | 0.36490298 | -4.12336292 | 0.33161558 | 994 |
| PC.aa.C32.3 | 0.64029923 | 0.49218356 | 0.79604769 | -1.57440891 | 2.55877603 | 868 |
| PC.aa.C34.1 | 0.32738743 | 1.11764394 | 0.59540733 | -1.12073446 | 3.35602234 | 935 |
| PC.aa.C36.1 | 0.76224842 | 0.3050118 | 0.83280015 | -1.67305662 | 2.28308023 | 924 |
| PC.aa.C36.2 | 0.78083573 | 0.29135779 | 0.83280015 | -1.76318954 | 2.34590512 | 925 |
| PC.aa.C36.3 | 0.62280547 | 0.61250605 | 0.79580699 | -1.83048543 | 3.05549754 | 936 |
| PC.aa.C38.0 | 0.20728924 | 1.3558909 | 0.52973918 | -0.75285139 | 3.46463318 | 867 |
| PC.aa.C38.5 | 0.74655053 | 0.36109706 | 0.83280015 | -1.83121586 | 2.55340998 | 836 |
| PC.aa.C40.4 | 0.36007749 | -0.99362795 | 0.61346535 | -3.12325319 | 1.13599728 | 925 |
| PC.aa.C40.5 | 0.33653458 | -1.07653537 | 0.59540733 | -3.27398901 | 1.12091827 | 836 |
| PC.ae.C36.1 | 0.32054009 | 0.99178395 | 0.59540733 | -0.96661609 | 2.950184 | 925 |
| PC.ae.C38.0 | 0.47393685 | 0.75520871 | 0.77861054 | -1.31370581 | 2.82412323 | 925 |
| SM.a.C38.1 | 0.5685123 | 0.63592299 | 0.77989595 | -1.55173527 | 2.82358125 | 993 |
| SM.a.C40.3 | 0.79659145 | -0.40005556 | 0.83280015 | -3.44607983 | 2.64596872 | 712 |
| SM.a.C40.5 | 0.03118773 | 2.35996121 | 0.16787447 | 0.21375105 | 4.50617136 | 877 |
| NEFA_26_1 | 0.97077491 | 0.04830982 | 0.97077491 | -2.53966981 | 2.63628944 | 714 |

**HDL**

| **Analytes** | **P-Values** | **BETA** | **FDR P-Value** | **CI lower** | **CI upper** | **Number of observations** |
| --- | --- | --- | --- | --- | --- | --- |
| lyso.PC.a.C18.1 | 0.00188545 | -0.0867251 | 0.04244896 | -0.14133404 | -0.03211616 | 925 |
| lyso.PC.a.C18.3 | 0.00423584 | -0.08893126 | 0.04244896 | -0.14979094 | -0.02807158 | 793 |
| SM.a.C41.0 | 0.23499906 | 0.03390509 | 0.3179399 | -0.02209566 | 0.08990583 | 776 |
| PC.aa.C34.2 | 0.03322815 | -0.05515674 | 0.07278546 | -0.1059171 | -0.00439639 | 925 |
| PC.aa.C34.3 | 0.02262385 | -0.06064474 | 0.05863456 | -0.11276393 | -0.00852554 | 925 |
| PC.aa.C36.4 | 0.19814806 | -0.03790938 | 0.28483783 | -0.09568506 | 0.0198663 | 865 |
| SM.a.C38.2 | 0.10412774 | -0.04054764 | 0.18387445 | -0.08946336 | 0.00836809 | 996 |
| SM.a.C40.4 | 0.02424412 | -0.05825751 | 0.05863456 | -0.10891655 | -0.00759848 | 995 |
| lyso.PC.a.C16.0 | 0.16788992 | -0.03750629 | 0.24912697 | -0.09084134 | 0.01582875 | 925 |
| lyso.PC.a.C16.1 | 0.10792631 | -0.03889281 | 0.18387445 | -0.08632594 | 0.00854033 | 996 |
| lyso.PC.a.C18.0 | 0.42869425 | -0.02266926 | 0.47881011 | -0.07885516 | 0.03351663 | 996 |
| lyso.PC.a.C20.3 | 0.02437324 | -0.0662185 | 0.05863456 | -0.1238496 | -0.00858741 | 853 |
| lyso.PC.a.C20.4 | 0.00461402 | -0.0787238 | 0.04244896 | -0.13313348 | -0.02431413 | 996 |
| lyso.PC.a.C20.5 | 0.02549329 | -0.0585652 | 0.05863456 | -0.10993382 | -0.00719657 | 920 |
| lyso.PC.a.C22.5 | 0.15142468 | -0.04185394 | 0.23218451 | -0.09906793 | 0.01536005 | 874 |
| lyso.PC.a.C22.6 | 8.15E-05 | -0.10055643 | 0.0037509 | -0.1504239 | -0.05068896 | 939 |
| lyso.PC.e.C18.0 | 0.05882544 | -0.05141913 | 0.11765088 | -0.1047597 | 0.00192144 | 967 |
| lyso.PC.e.C18.1 | 0.00783876 | -0.09153214 | 0.04686588 | -0.15892469 | -0.02413958 | 707 |
| PC.aa.C36.0 | 0.00941039 | -0.05627628 | 0.04686588 | -0.09871822 | -0.01383433 | 924 |
| PC.aa.C38.3 | 0.94549041 | 0.00184324 | 0.94549041 | -0.0510574 | 0.05474387 | 836 |
| PC.aa.C38.4 | 0.31809328 | -0.02569531 | 0.40645252 | -0.07618204 | 0.02479143 | 836 |
| PC.aa.C38.6 | 0.02100537 | -0.07162357 | 0.05863456 | -0.13242232 | -0.01082483 | 836 |
| PC.aa.C40.6 | 0.43717445 | -0.02174118 | 0.47881011 | -0.07663438 | 0.03315202 | 901 |
| PC.ae.C40.0 | 0.00848188 | -0.0722637 | 0.04686588 | -0.12602549 | -0.01850191 | 925 |
| PC.ae.C40.6 | 0.01788838 | -0.06278901 | 0.05863456 | -0.11473274 | -0.01084529 | 836 |
| SM.a.C36.0 | 0.43613719 | 0.02512649 | 0.47881011 | -0.03819402 | 0.08844699 | 643 |
| SM.a.C42.4 | 0.43210987 | -0.01953243 | 0.47881011 | -0.06830983 | 0.02924498 | 865 |
| SM.a.C42.6 | 0.0067854 | -0.08495953 | 0.04686588 | -0.1464062 | -0.02351286 | 865 |
| SM.a.C44.6 | 0.26234891 | -0.03019421 | 0.34480143 | -0.08303527 | 0.02264686 | 810 |
| NEFA_24_5 | 0.60124187 | 0.02362259 | 0.64318898 | -0.06510225 | 0.11234742 | 602 |
| Carn.a.C3.0 | 0.79149475 | 0.00712881 | 0.80908352 | -0.04577268 | 0.0600303 | 994 |
| PC.aa.C32.3 | 0.34331538 | -0.02347731 | 0.42682452 | -0.07207626 | 0.02512164 | 868 |
| PC.aa.C34.1 | 0.01018823 | -0.065512 | 0.04686588 | -0.11544851 | -0.01557549 | 935 |
| PC.aa.C36.1 | 0.20557567 | -0.02935705 | 0.28656003 | -0.07484038 | 0.01612627 | 924 |
| PC.aa.C36.2 | 0.14056478 | -0.03418045 | 0.22296483 | -0.07966009 | 0.0112992 | 925 |
| PC.aa.C36.3 | 0.13163521 | -0.04100754 | 0.21625785 | -0.09433915 | 0.01232407 | 936 |
| PC.aa.C38.0 | 0.02353235 | -0.05632118 | 0.05863456 | -0.10504602 | -0.00759635 | 867 |
| PC.aa.C38.5 | 0.04551673 | -0.04976004 | 0.09517134 | -0.09852537 | -0.00099471 | 836 |
| PC.aa.C40.4 | 0.42473249 | -0.02008291 | 0.47881011 | -0.06943685 | 0.02927102 | 925 |
| PC.aa.C40.5 | 0.73651314 | -0.00890155 | 0.76999101 | -0.0608115 | 0.04300839 | 836 |
| PC.ae.C36.1 | 0.02075564 | -0.05269837 | 0.05863456 | -0.09734657 | -0.00805017 | 925 |
| PC.ae.C38.0 | 0.01163551 | -0.05885532 | 0.04865757 | -0.10454477 | -0.01316587 | 925 |
| SM.a.C38.1 | 0.01313568 | -0.0612674 | 0.05035343 | -0.10965778 | -0.01287701 | 993 |
| SM.a.C40.3 | 0.08831241 | -0.05825458 | 0.16249483 | -0.12526761 | 0.00875846 | 712 |
| SM.a.C40.5 | 0.00434003 | -0.07342387 | 0.04244896 | -0.12381442 | -0.02303332 | 877 |
| NEFA_26_1 | 0.08513043 | -0.05438939 | 0.16249483 | -0.1163262 | 0.00754742 | 714 |

**Triacylglycerol**

| **Analytes** | **P-Values** | **BETA** | **FDR P-Value** | **CI lower** | **CI upper** | **Number of observations** |
| --- | --- | --- | --- | --- | --- | --- |
| lyso.PC.a.C18.1 | 0.1138274 | 0.08499625 | 0.1586685 | -0.02039728 | 0.19038977 | 925 |
| lyso.PC.a.C18.3 | 0.00431873 | 0.1689871 | 0.00866116 | 0.05309104 | 0.28488317 | 793 |
| SM.a.C41.0 | 0.17722199 | 0.07324598 | 0.22033004 | -0.03321496 | 0.17970692 | 776 |
| PC.aa.C34.2 | 0.00369663 | 0.14358522 | 0.00809739 | 0.04676306 | 0.24040739 | 925 |
| PC.aa.C34.3 | 0.00030486 | 0.17523792 | 0.00180113 | 0.08036503 | 0.2701108 | 925 |
| PC.aa.C36.4 | 0.00111676 | 0.18002399 | 0.00366934 | 0.07198256 | 0.28806542 | 865 |
| SM.a.C38.2 | 0.04955082 | 0.09266532 | 0.08140491 | 0.00018237 | 0.18514828 | 996 |
| SM.a.C40.4 | 0.0018443 | 0.14999429 | 0.00472189 | 0.05573408 | 0.2442545 | 995 |
| lyso.PC.a.C16.0 | 0.01328543 | 0.12341607 | 0.02546375 | 0.02578373 | 0.2210484 | 925 |
| lyso.PC.a.C16.1 | 0.031938 | 0.09373688 | 0.0544129 | 0.00811 | 0.17936376 | 996 |
| lyso.PC.a.C18.0 | 0.02148791 | 0.11960055 | 0.03953776 | 0.01768821 | 0.2215129 | 996 |
| lyso.PC.a.C20.3 | 0.0748888 | 0.09846296 | 0.11112532 | -0.0099067 | 0.20683261 | 853 |
| lyso.PC.a.C20.4 | 0.14296577 | 0.07615229 | 0.18789787 | -0.02578392 | 0.1780885 | 996 |
| lyso.PC.a.C20.5 | 0.19268733 | 0.06430838 | 0.22727223 | -0.03250523 | 0.16112199 | 920 |
| lyso.PC.a.C22.5 | 0.36043705 | 0.05065964 | 0.38978751 | -0.05800486 | 0.15932415 | 874 |
| lyso.PC.a.C22.6 | 0.39235672 | 0.04057925 | 0.41019112 | -0.05248229 | 0.13364079 | 939 |
| lyso.PC.e.C18.0 | 0.78259255 | 0.01386012 | 0.78259255 | -0.08468232 | 0.11240256 | 967 |
| lyso.PC.e.C18.1 | 0.06042699 | 0.12203354 | 0.09265472 | -0.00536317 | 0.24943025 | 707 |
| PC.aa.C36.0 | 0.34414901 | 0.03992629 | 0.3861184 | -0.04286135 | 0.12271393 | 924 |
| PC.aa.C38.3 | 0.00027569 | 0.16671947 | 0.00180113 | 0.07713294 | 0.256306 | 836 |
| PC.aa.C38.4 | 0.00072133 | 0.15843523 | 0.00335533 | 0.06681013 | 0.25006033 | 836 |
| PC.aa.C38.6 | 0.13672474 | 0.08723885 | 0.18498053 | -0.02771922 | 0.20219693 | 836 |
| PC.aa.C40.6 | 0.14894392 | 0.0728372 | 0.19031723 | -0.02612446 | 0.17179886 | 901 |
| PC.ae.C40.0 | 0.27811957 | 0.0560332 | 0.31983751 | -0.04530149 | 0.15736788 | 925 |
| PC.ae.C40.6 | 0.58258333 | 0.028398 | 0.59552962 | -0.07297938 | 0.12977538 | 836 |
| SM.a.C36.0 | 0.00162804 | 0.18598155 | 0.00468062 | 0.07056963 | 0.30139347 | 643 |
| SM.a.C42.4 | 0.0010475 | 0.15084971 | 0.00366934 | 0.06082083 | 0.24087859 | 865 |
| SM.a.C42.6 | 0.1873986 | 0.07763277 | 0.22685093 | -0.03785628 | 0.19312182 | 865 |
| SM.a.C44.6 | 0.05864775 | 0.09539568 | 0.09265472 | -0.00349641 | 0.19428777 | 810 |
| NEFA_24_5 | 0.11240971 | -0.13232397 | 0.1586685 | -0.29578974 | 0.03114179 | 602 |
| Carn.a.C3.0 | 0.03193572 | 0.10386546 | 0.0544129 | 0.00898762 | 0.1987433 | 994 |
| PC.aa.C32.3 | 0.00024243 | 0.16747417 | 0.00180113 | 0.07828786 | 0.25666047 | 868 |
| PC.aa.C34.1 | 8.70E-05 | 0.17650568 | 0.00180113 | 0.08862596 | 0.2643854 | 935 |
| PC.aa.C36.1 | 1.77E-05 | 0.17643478 | 0.00081391 | 0.09618849 | 0.25668107 | 924 |
| PC.aa.C36.2 | 0.00013368 | 0.16443371 | 0.00180113 | 0.08030414 | 0.24856327 | 925 |
| PC.aa.C36.3 | 0.00031324 | 0.17868024 | 0.00180113 | 0.08175053 | 0.27560995 | 936 |
| PC.aa.C38.0 | 0.36436659 | 0.04366715 | 0.38978751 | -0.05076953 | 0.13810382 | 867 |
| PC.aa.C38.5 | 0.00072942 | 0.15635028 | 0.00335533 | 0.06584831 | 0.24685226 | 836 |
| PC.aa.C40.4 | 0.00329655 | 0.13249363 | 0.00758207 | 0.04424079 | 0.22074648 | 925 |
| PC.aa.C40.5 | 0.00136673 | 0.14826344 | 0.0041913 | 0.05767435 | 0.23885252 | 836 |
| PC.ae.C36.1 | 0.00262442 | 0.1279796 | 0.00635386 | 0.0447256 | 0.2112336 | 925 |
| PC.ae.C38.0 | 0.0018477 | 0.13864253 | 0.00472189 | 0.05151065 | 0.22577441 | 925 |
| SM.a.C38.1 | 0.00086804 | 0.15333515 | 0.00363 | 0.06325509 | 0.24341521 | 993 |
| SM.a.C40.3 | 0.00109205 | 0.20637413 | 0.00366934 | 0.08281419 | 0.32993406 | 712 |
| SM.a.C40.5 | 0.00020823 | 0.17605855 | 0.00180113 | 0.08328367 | 0.26883343 | 877 |
| NEFA_26_1 | 0.00433058 | 0.1646378 | 0.00866116 | 0.05170729 | 0.27756831 | 714 |

**Glucose**

| **Analytes** | **P-Values** | **BETA** | **FDR P-Value** | **CI lower** | **CI upper** | **Number of observations** |
| --- | --- | --- | --- | --- | --- | --- |
| lyso.PC.a.C18.1 | 0.46548217 | 0.03054538 | 0.62205378 | -0.05155629 | 0.11264706 | 925 |
| lyso.PC.a.C18.3 | 0.42512938 | 0.03672946 | 0.60113072 | -0.05362421 | 0.12708314 | 793 |
| SM.a.C41.0 | 0.18319508 | 0.05535571 | 0.34252665 | -0.0262144 | 0.13692581 | 776 |
| PC.aa.C34.2 | 0.00121917 | 0.13088367 | 0.01869393 | 0.05171355 | 0.21005378 | 925 |
| PC.aa.C34.3 | 0.00730879 | 0.10889977 | 0.05603406 | 0.02940322 | 0.18839631 | 925 |
| PC.aa.C36.4 | 0.00656591 | 0.12321911 | 0.05603406 | 0.03446017 | 0.21197805 | 865 |
| SM.a.C38.2 | 0.00070697 | 0.13017382 | 0.01626025 | 0.05498814 | 0.2053595 | 996 |
| SM.a.C40.4 | 0.00063829 | 0.13527266 | 0.01626025 | 0.05778506 | 0.21276027 | 995 |
| lyso.PC.a.C16.0 | 0.08025216 | 0.06893584 | 0.30763326 | -0.00832156 | 0.14619324 | 925 |
| lyso.PC.a.C16.1 | 0.85503307 | -0.00649576 | 0.8740338 | -0.07624782 | 0.06325629 | 996 |
| lyso.PC.a.C18.0 | 0.12526925 | 0.06422868 | 0.34252665 | -0.01791823 | 0.14637559 | 996 |
| lyso.PC.a.C20.3 | 0.43124595 | 0.03403675 | 0.60113072 | -0.0508034 | 0.1188769 | 853 |
| lyso.PC.a.C20.4 | 0.54875457 | 0.02489558 | 0.67281469 | -0.05655073 | 0.10634189 | 996 |
| lyso.PC.a.C20.5 | 0.35573689 | 0.03519201 | 0.53726122 | -0.03955643 | 0.10994046 | 920 |
| lyso.PC.a.C22.5 | 0.57720082 | -0.02357982 | 0.68080097 | -0.10656555 | 0.0594059 | 874 |
| lyso.PC.a.C22.6 | 0.82617829 | 0.00823247 | 0.86373185 | -0.06531655 | 0.08178148 | 939 |
| lyso.PC.e.C18.0 | 0.36206735 | 0.0364627 | 0.53726122 | -0.04200899 | 0.1149344 | 967 |
| lyso.PC.e.C18.1 | 0.92846294 | 0.00452825 | 0.92846294 | -0.09446368 | 0.10352019 | 707 |
| PC.aa.C36.0 | 0.11311768 | 0.05214272 | 0.34252665 | -0.01238596 | 0.11667141 | 924 |
| PC.aa.C38.3 | 0.7746683 | 0.01137141 | 0.82871492 | -0.06657094 | 0.08931376 | 836 |
| PC.aa.C38.4 | 0.17077128 | 0.0530021 | 0.34252665 | -0.0228843 | 0.12888851 | 836 |
| PC.aa.C38.6 | 0.07414145 | 0.08360729 | 0.30763326 | -0.00817517 | 0.17538975 | 836 |
| PC.aa.C40.6 | 0.54192395 | 0.02570166 | 0.67281469 | -0.05697214 | 0.10837545 | 901 |
| PC.ae.C40.0 | 0.18615579 | 0.05444027 | 0.34252665 | -0.02631531 | 0.13519585 | 925 |
| PC.ae.C40.6 | 0.22746627 | 0.04842842 | 0.38758351 | -0.03027356 | 0.1271304 | 836 |
| SM.a.C36.0 | 0.16213033 | 0.06369863 | 0.34252665 | -0.02567543 | 0.15307269 | 643 |
| SM.a.C42.4 | 0.13068517 | 0.05608357 | 0.34252665 | -0.01667765 | 0.12884479 | 865 |
| SM.a.C42.6 | 0.17581899 | 0.06331098 | 0.34252665 | -0.0284052 | 0.15502716 | 865 |
| SM.a.C44.6 | 0.66469833 | -0.01710953 | 0.74985556 | -0.09456571 | 0.06034664 | 810 |
| NEFA_24_5 | 0.71128159 | 0.02248229 | 0.7790227 | -0.096753 | 0.14171758 | 602 |
| Carn.a.C3.0 | 0.66834952 | -0.01687137 | 0.74985556 | -0.09412826 | 0.06038552 | 994 |
| PC.aa.C32.3 | 0.13330242 | 0.05468669 | 0.34252665 | -0.01674485 | 0.12611822 | 868 |
| PC.aa.C34.1 | 0.06619861 | 0.07202816 | 0.30451361 | -0.00482785 | 0.14888418 | 935 |
| PC.aa.C36.1 | 0.16301335 | 0.04761617 | 0.34252665 | -0.01931839 | 0.11455074 | 924 |
| PC.aa.C36.2 | 0.01760555 | 0.0840374 | 0.10123189 | 0.0146849 | 0.1533899 | 925 |
| PC.aa.C36.3 | 0.02304951 | 0.09710197 | 0.11780858 | 0.01338842 | 0.18081553 | 936 |
| PC.aa.C38.0 | 0.26806221 | 0.04112896 | 0.44038792 | -0.03171078 | 0.11396869 | 867 |
| PC.aa.C38.5 | 0.16301541 | 0.05321174 | 0.34252665 | -0.02159305 | 0.12801652 | 836 |
| PC.aa.C40.4 | 0.22749467 | 0.04466213 | 0.38758351 | -0.02791855 | 0.11724281 | 925 |
| PC.aa.C40.5 | 0.55580344 | 0.02295544 | 0.67281469 | -0.0535008 | 0.09941168 | 836 |
| PC.ae.C36.1 | 0.16438584 | 0.04704814 | 0.34252665 | -0.01930392 | 0.1134002 | 925 |
| PC.ae.C38.0 | 0.01398479 | 0.08763475 | 0.09190004 | 0.01778846 | 0.15748103 | 925 |
| SM.a.C38.1 | 0.00374245 | 0.11048289 | 0.04303822 | 0.03587617 | 0.18508961 | 993 |
| SM.a.C40.3 | 0.47330179 | 0.03595853 | 0.62205378 | -0.06243644 | 0.13435351 | 712 |
| SM.a.C40.5 | 0.14389093 | 0.0565296 | 0.34252665 | -0.01931977 | 0.13237896 | 877 |
| NEFA_26_1 | 0.3560959 | 0.04039323 | 0.53726122 | -0.0454873 | 0.12627376 | 714 |

**Systolic blood pressure**

| **Analytes** | **P-Values** | **BETA** | **FDR P-Value** | **CI lower** | **CI upper** | **Number of observations** |
| --- | --- | --- | --- | --- | --- | --- |
| lyso.PC.a.C18.1 | 0.29329593 | 1.30517176 | 0.97122968 | -1.13079101 | 3.74113452 | 925 |
| lyso.PC.a.C18.3 | 0.02039483 | 3.12920467 | 0.62513218 | 0.4857732 | 5.77263614 | 793 |
| SM.a.C41.0 | 0.13854627 | -1.82382746 | 0.97122968 | -4.23841686 | 0.59076195 | 776 |
| PC.aa.C34.2 | 0.59822813 | 0.63429629 | 0.97122968 | -1.72722837 | 2.99582094 | 925 |
| PC.aa.C34.3 | 0.11981211 | 1.86435491 | 0.97122968 | -0.48558884 | 4.21429866 | 925 |
| PC.aa.C36.4 | 0.39450156 | 1.13287962 | 0.97122968 | -1.47718357 | 3.7429428 | 865 |
| SM.a.C38.2 | 0.95011599 | -0.06969687 | 0.97122968 | -2.2553358 | 2.11594206 | 996 |
| SM.a.C40.4 | 0.02717966 | 2.52474893 | 0.62513218 | 0.28514836 | 4.76434949 | 995 |
| lyso.PC.a.C16.0 | 0.15400032 | 1.66633968 | 0.97122968 | -0.62582349 | 3.95850285 | 925 |
| lyso.PC.a.C16.1 | 0.85921491 | 0.18184569 | 0.97122968 | -1.82947169 | 2.19316307 | 996 |
| lyso.PC.a.C18.0 | 0.73246689 | 0.41658764 | 0.97122968 | -1.97415782 | 2.80733311 | 996 |
| lyso.PC.a.C20.3 | 0.08771052 | 2.16746925 | 0.97122968 | -0.32100821 | 4.65594672 | 853 |
| lyso.PC.a.C20.4 | 0.17278812 | 1.64396042 | 0.97122968 | -0.72068501 | 4.00860586 | 996 |
| lyso.PC.a.C20.5 | 0.57117481 | 0.64312381 | 0.97122968 | -1.58478893 | 2.87103655 | 920 |
| lyso.PC.a.C22.5 | 0.92615472 | -0.11777006 | 0.97122968 | -2.61098256 | 2.37544244 | 874 |
| lyso.PC.a.C22.6 | 0.38112012 | 0.95573633 | 0.97122968 | -1.18480045 | 3.0962731 | 939 |
| lyso.PC.e.C18.0 | 0.98557829 | -0.02087754 | 0.98557829 | -2.28688653 | 2.24513146 | 967 |
| lyso.PC.e.C18.1 | 0.59979184 | 0.77127801 | 0.97122968 | -2.11342963 | 3.65598565 | 707 |
| PC.aa.C36.0 | 0.37399665 | 0.86807684 | 0.97122968 | -1.04732643 | 2.78348011 | 924 |
| PC.aa.C38.3 | 0.68392771 | -0.46221287 | 0.97122968 | -2.68993457 | 1.76550884 | 836 |
| PC.aa.C38.4 | 0.62212847 | 0.55147242 | 0.97122968 | -1.64406677 | 2.7470116 | 836 |
| PC.aa.C38.6 | 0.58600691 | 0.74787205 | 0.97122968 | -1.94637394 | 3.44211805 | 836 |
| PC.aa.C40.6 | 0.92230325 | 0.11703695 | 0.97122968 | -2.23739409 | 2.47146799 | 901 |
| PC.ae.C40.0 | 0.91926231 | 0.1239062 | 0.97122968 | -2.27445828 | 2.52227068 | 925 |
| PC.ae.C40.6 | 0.65959169 | -0.51812718 | 0.97122968 | -2.8261463 | 1.78989194 | 836 |
| SM.a.C36.0 | 0.23663156 | 1.61550479 | 0.97122968 | -1.06258011 | 4.29358969 | 643 |
| SM.a.C42.4 | 0.81475675 | -0.254393 | 0.97122968 | -2.38484456 | 1.87605856 | 865 |
| SM.a.C42.6 | 0.65672393 | -0.61278958 | 0.97122968 | -3.31803751 | 2.09245834 | 865 |
| SM.a.C44.6 | 0.75389071 | -0.36464343 | 0.97122968 | -2.64691377 | 1.91762691 | 810 |
| NEFA_24_5 | 0.28600807 | -2.04450443 | 0.97122968 | -5.80459525 | 1.71558639 | 602 |
| Carn.a.C3.0 | 0.75674185 | -0.35215863 | 0.97122968 | -2.58249531 | 1.87817804 | 994 |
| PC.aa.C32.3 | 0.1380804 | 1.60896718 | 0.97122968 | -0.51852531 | 3.73645967 | 868 |
| PC.aa.C34.1 | 0.90347373 | 0.1356842 | 0.97122968 | -2.05942674 | 2.33079515 | 935 |
| PC.aa.C36.1 | 0.82371685 | -0.22453545 | 0.97122968 | -2.2021036 | 1.7530327 | 924 |
| PC.aa.C36.2 | 0.88957608 | 0.14567464 | 0.97122968 | -1.91290593 | 2.20425521 | 925 |
| PC.aa.C36.3 | 0.75124138 | -0.38792461 | 0.97122968 | -2.78880049 | 2.01295128 | 936 |
| PC.aa.C38.0 | 0.75226384 | -0.34993121 | 0.97122968 | -2.52508467 | 1.82522226 | 867 |
| PC.aa.C38.5 | 0.89454539 | 0.14593887 | 0.97122968 | -2.01441301 | 2.30629076 | 836 |
| PC.aa.C40.4 | 0.76101131 | -0.32959146 | 0.97122968 | -2.45565213 | 1.79646921 | 925 |
| PC.aa.C40.5 | 0.91264499 | -0.12309462 | 0.97122968 | -2.32485959 | 2.07867035 | 836 |
| PC.ae.C36.1 | 0.63829601 | -0.47154102 | 0.97122968 | -2.43952681 | 1.49644477 | 925 |
| PC.ae.C38.0 | 0.56116454 | 0.6112458 | 0.97122968 | -1.45232037 | 2.67481196 | 925 |
| SM.a.C38.1 | 0.56128813 | 0.64125766 | 0.97122968 | -1.52416934 | 2.80668466 | 993 |
| SM.a.C40.3 | 0.34046469 | 1.45030051 | 0.97122968 | -1.53475635 | 4.43535736 | 712 |
| SM.a.C40.5 | 0.61200137 | 0.56071994 | 0.97122968 | -1.60821495 | 2.72965483 | 877 |
| NEFA_26_1 | 0.92918623 | -0.11750057 | 0.97122968 | -2.71244687 | 2.47744574 | 714 |

## Supplemental Table 7: Median, 25% and 75 % quartile for every metabolite (215) of the Raine Study metabolomics data set stratified by males, non-hormonal and hormonal contraceptive taking females.

### Supplemental Table 7.1: Non-hormonal taking females.

| **Metabolites** | **Median** | **25% Quartile** | **75% Quartile** |
| --- | --- | --- | --- |
| Carn | 39.034 | 33.576 | 43.977 |
| Carn.a.C10.0 | 0.273 | 0.185 | 0.372 |
| Carn.a.C10.1 | 0.307 | 0.235 | 0.49 |
| Carn.a.C12.0 | 0.092 | 0.058 | 0.126 |
| Carn.a.C12.1 | 0.357 | 0.251 | 0.519 |
| Carn.a.C14.0 | 0.034 | 0.025 | 0.052 |
| Carn.a.C14.1 | 0.111 | 0.09 | 0.149 |
| Carn.a.C14.2 | 0.02 | 0.014 | 0.03 |
| Carn.a.C16.0 | 0.081 | 0.064 | 0.095 |
| Carn.a.C16.1 | 0.037 | 0.026 | 0.049 |
| Carn.a.C18.0 | 0.034 | 0.028 | 0.042 |
| Carn.a.C18.1 | 0.099 | 0.08 | 0.119 |
| Carn.a.C2.0 | 4.778 | 3.879 | 5.941 |
| Carn.a.C3.0 | 0.348 | 0.28 | 0.429 |
| Carn.a.C4.0 | 0.207 | 0.165 | 0.259 |
| Carn.a.C4.0.DC | 0.001 | 0.001 | 0.002 |
| Carn.a.C5.0 | 0.145 | 0.107 | 0.203 |
| Carn.a.C6.0 | 0.054 | 0.039 | 0.07 |
| Carn.a.C8.1 | 0.122 | 0.083 | 0.162 |
| lyso.PC.a.C14.0 | 1.921 | 1.477 | 2.508 |
| lyso.PC.a.C16.0 | 74.599 | 65.999 | 92.594 |
| lyso.PC.a.C16.1 | 3.073 | 2.532 | 3.832 |
| lyso.PC.a.C18.0 | 21.564 | 17.859 | 24.943 |
| lyso.PC.a.C18.1 | 21.751 | 17.529 | 25.747 |
| lyso.PC.a.C18.2 | 39.504 | 30.028 | 47.332 |
| lyso.PC.a.C18.3 | 0.789 | 0.586 | 1.013 |
| lyso.PC.a.C18.6 | 0.291 | 0.253 | 0.36 |
| lyso.PC.a.C20.2 | 0.215 | 0.17 | 0.278 |
| lyso.PC.a.C20.3 | 2.174 | 1.714 | 2.695 |
| lyso.PC.a.C20.4 | 6.523 | 5.331 | 8.002 |
| lyso.PC.a.C20.5 | 0.75 | 0.54 | 1.001 |
| lyso.PC.a.C22.5 | 0.56 | 0.478 | 0.694 |
| lyso.PC.a.C22.6 | 1.554 | 1.279 | 2.017 |
| lyso.PC.e.C16.0 | 0.938 | 0.747 | 1.106 |
| lyso.PC.e.C18.0 | 1.581 | 1.299 | 1.916 |
| lyso.PC.e.C18.1 | 0.361 | 0.302 | 0.455 |
| PC.aa.C30.0 | 3.391 | 2.499 | 4.426 |
| PC.aa.C30.1 | 2.195 | 1.73 | 2.894 |
| PC.aa.C30.2 | 0.541 | 0.424 | 0.633 |
| PC.aa.C32.0 | 12.81 | 10.873 | 14.735 |
| PC.aa.C32.1 | 15.143 | 12.006 | 20.189 |
| PC.aa.C32.2 | 3.586 | 2.637 | 4.398 |
| PC.aa.C32.3 | 0.485 | 0.388 | 0.607 |
| PC.aa.C34.0 | 2.786 | 2.255 | 3.499 |
| PC.aa.C34.1 | 213.892 | 178.873 | 251.871 |
| PC.aa.C34.2 | 390.29 | 346.152 | 448.067 |
| PC.aa.C34.3 | 13.759 | 11.888 | 16.375 |
| PC.aa.C34.4 | 1.765 | 1.321 | 2.14 |
| PC.aa.C34.5 | 0.251 | 0.176 | 0.337 |
| PC.aa.C36.0 | 1.477 | 1.196 | 1.887 |
| PC.aa.C36.1 | 47.764 | 40.296 | 57.972 |
| PC.aa.C36.2 | 254.507 | 226.537 | 306.406 |
| PC.aa.C36.3 | 140.328 | 119.083 | 167.77 |
| PC.aa.C36.4 | 164.72 | 141.652 | 192.815 |
| PC.aa.C36.5 | 21.095 | 16.296 | 25.778 |
| PC.aa.C36.6 | 0.778 | 0.598 | 1.015 |
| PC.aa.C38.0 | 1.956 | 1.548 | 2.347 |
| PC.aa.C38.1 | 0.633 | 0.507 | 0.77 |
| PC.aa.C38.2 | 4.369 | 3.425 | 5.256 |
| PC.aa.C38.3 | 41.905 | 34.038 | 52.764 |
| PC.aa.C38.4 | 106.402 | 89.831 | 126.018 |
| PC.aa.C38.5 | 57.518 | 49.288 | 67.362 |
| PC.aa.C38.6 | 75.84 | 61.699 | 88.202 |
| PC.aa.C40.0 | 0.418 | 0.332 | 0.497 |
| PC.aa.C40.1 | 0.202 | 0.155 | 0.244 |
| PC.aa.C40.3 | 0.238 | 0.182 | 0.302 |
| PC.aa.C40.4 | 3.09 | 2.545 | 3.607 |
| PC.aa.C40.5 | 11.84 | 10.015 | 14.339 |
| PC.aa.C40.6 | 27.719 | 23.004 | 33.168 |
| PC.aa.C42.0 | 0.417 | 0.35 | 0.488 |
| PC.aa.C42.1 | 0.145 | 0.112 | 0.179 |
| PC.aa.C42.2 | 0.077 | 0.064 | 0.102 |
| PC.aa.C42.4 | 0.105 | 0.09 | 0.132 |
| PC.aa.C42.5 | 0.241 | 0.188 | 0.279 |
| PC.aa.C42.6 | 0.572 | 0.446 | 0.691 |
| PC.aa.C43.4 | 0.188 | 0.154 | 0.231 |
| PC.aa.C43.6 | 1.01 | 0.864 | 1.208 |
| PC.aa.C44.12 | 1.129 | 0.896 | 1.381 |
| PC.ae.C30.0 | 0.272 | 0.187 | 0.354 |
| PC.ae.C30.1 | 0.682 | 0.516 | 0.837 |
| PC.ae.C32.0 | 2.959 | 2.54 | 3.46 |
| PC.ae.C32.1 | 2.953 | 2.484 | 3.497 |
| PC.ae.C32.2 | 0.925 | 0.748 | 1.141 |
| PC.ae.C34.0 | 1.551 | 1.257 | 1.875 |
| PC.ae.C34.1 | 10.303 | 9.055 | 11.885 |
| PC.ae.C34.2 | 11.819 | 10.255 | 13.833 |
| PC.ae.C34.3 | 9.111 | 7.846 | 11.427 |
| PC.ae.C34.4 | 0.226 | 0.172 | 0.308 |
| PC.ae.C36.0 | 0.356 | 0.289 | 0.429 |
| PC.ae.C36.1 | 3.654 | 2.987 | 4.38 |
| PC.ae.C36.2 | 11.428 | 9.924 | 13.819 |
| PC.ae.C36.3 | 8.295 | 7.122 | 10.035 |
| PC.ae.C36.4 | 16.582 | 14.09 | 19.687 |
| PC.ae.C36.5 | 11.427 | 9.679 | 13.821 |
| PC.ae.C36.6 | 0.678 | 0.478 | 0.959 |
| PC.ae.C38.0 | 1.807 | 1.435 | 2.182 |
| PC.ae.C38.2 | 1.079 | 0.893 | 1.305 |
| PC.ae.C38.3 | 3.366 | 2.895 | 4.084 |
| PC.ae.C38.4 | 13.323 | 11.636 | 15.493 |
| PC.ae.C38.5 | 17.863 | 15.092 | 19.977 |
| PC.ae.C38.6 | 7.644 | 6.487 | 9.436 |
| PC.ae.C40.0 | 11.237 | 8.88 | 13.177 |
| PC.ae.C40.1 | 1.226 | 0.986 | 1.463 |
| PC.ae.C40.2 | 0.97 | 0.811 | 1.186 |
| PC.ae.C40.3 | 0.625 | 0.493 | 0.769 |
| PC.ae.C40.4 | 1.743 | 1.518 | 2.105 |
| PC.ae.C40.5 | 3.713 | 3.224 | 4.4 |
| PC.ae.C40.6 | 4.818 | 4.196 | 5.715 |
| PC.ae.C42.0 | 0.304 | 0.242 | 0.392 |
| PC.ae.C42.1 | 0.202 | 0.166 | 0.25 |
| PC.ae.C42.2 | 0.413 | 0.334 | 0.504 |
| PC.ae.C42.3 | 0.71 | 0.548 | 0.874 |
| PC.ae.C42.4 | 0.858 | 0.731 | 1.068 |
| PC.ae.C42.5 | 2.294 | 1.918 | 2.692 |
| PC.ae.C42.6 | 1.119 | 0.928 | 1.379 |
| SM.a.C30.1 | 0.407 | 0.312 | 0.506 |
| SM.a.C32.0 | 0.363 | 0.269 | 0.493 |
| SM.a.C32.1 | 8.426 | 6.983 | 9.742 |
| SM.a.C32.2 | 0.753 | 0.587 | 0.968 |
| SM.a.C33.1 | 6.024 | 5.145 | 6.826 |
| SM.a.C33.2 | 0.204 | 0.161 | 0.25 |
| SM.a.C34.0 | 1.836 | 1.254 | 2.588 |
| SM.a.C34.1 | 100.21 | 87.118 | 112.885 |
| SM.a.C34.2 | 14.972 | 13.007 | 16.844 |
| SM.a.C34.3 | 0.103 | 0.083 | 0.126 |
| SM.a.C35.0 | 0.584 | 0.489 | 0.694 |
| SM.a.C35.1 | 2.891 | 2.482 | 3.418 |
| SM.a.C36.0 | 1.369 | 1.094 | 1.718 |
| SM.a.C36.1 | 18.989 | 16.078 | 21.781 |
| SM.a.C36.2 | 10.415 | 8.991 | 12.502 |
| SM.a.C36.3 | 0.798 | 0.649 | 0.934 |
| SM.a.C37.1 | 2.064 | 1.728 | 2.494 |
| SM.a.C38.1 | 31.022 | 26.045 | 37.64 |
| SM.a.C38.2 | 18.306 | 15.637 | 21.173 |
| SM.a.C39.1 | 4.703 | 3.98 | 5.697 |
| SM.a.C39.2 | 1.412 | 1.177 | 1.741 |
| SM.a.C40.1 | 15.88 | 11.954 | 19.882 |
| SM.a.C40.2 | 30.736 | 27.074 | 35.557 |
| SM.a.C40.3 | 9.529 | 8.076 | 12.019 |
| SM.a.C40.4 | 2.345 | 1.963 | 2.783 |
| SM.a.C40.5 | 0.947 | 0.738 | 1.202 |
| SM.a.C41.1 | 10.32 | 8.784 | 12.303 |
| SM.a.C41.2 | 8.635 | 7.412 | 10.224 |
| SM.a.C42.1 | 16.653 | 14.133 | 19.755 |
| SM.a.C42.2 | 41.458 | 35.349 | 47.694 |
| SM.a.C42.3 | 24.171 | 21.083 | 27.828 |
| SM.a.C42.4 | 7.212 | 6.377 | 8.467 |
| SM.a.C42.5 | 2.549 | 2.141 | 3.291 |
| SM.a.C42.6 | 3.032 | 2.394 | 3.423 |
| SM.a.C43.1 | 1.063 | 0.886 | 1.3 |
| SM.a.C43.2 | 2.419 | 1.841 | 2.902 |
| SM.a.C44.2 | 0.256 | 0.184 | 0.332 |
| SM.a.C44.6 | 1.222 | 0.931 | 1.495 |
| SM.a.C31.1 | 0.255 | 0.188 | 0.319 |
| SM.a.C33.3 | 0.113 | 0.096 | 0.137 |
| SM.a.C35.2 | 0.435 | 0.356 | 0.509 |
| SM.a.C37.3 | 0.339 | 0.255 | 0.414 |
| SM.a.C39.5 | 0.397 | 0.298 | 0.493 |
| SM.a.C41.0 | 0.471 | 0.343 | 0.637 |
| SM.a.C41.3 | 1.383 | 1.077 | 1.664 |
| SM.a.C43.0 | 0.636 | 0.517 | 0.787 |
| SM.a.C43.3 | 0.578 | 0.478 | 0.71 |
| Ala | 313 | 258 | 391 |
| Arg | 68.2 | 53.75 | 80.7 |
| Asn | 40.4 | 34.5 | 46.8 |
| Asp | 7.64 | 6.35 | 8.945 |
| Cit | 23.7 | 19.1 | 29.35 |
| Gln | 541 | 459 | 637 |
| Glu | 53.9 | 43.65 | 66.65 |
| Gly | 214 | 177 | 271 |
| His | 80.55 | 66.8 | 105 |
| Ile | 51.8 | 43.825 | 60.625 |
| Leu | 104 | 93.5 | 119 |
| Lys | 162 | 130 | 219 |
| Met | 23.6 | 19.9 | 26.8 |
| Orn | 46.6 | 38.1 | 59.8 |
| Phe | 51.1 | 45.4 | 57.2 |
| Pro | 150 | 116 | 194 |
| Trp | 97.6 | 61.05 | 118.5 |
| Ser | 108 | 82.45 | 131 |
| Thr | 133 | 101 | 193 |
| Tyr | 54 | 46.5 | 63.7 |
| Val | 188 | 162 | 222 |
| NEFA_12_0 | 1.45 | 0.878 | 2.345 |
| NEFA_14_0 | 4.965 | 2.542 | 9.323 |
| NEFA_14_1 | 0.942 | 0.607 | 1.575 |
| NEFA_15_0 | 0.736 | 0.424 | 1.17 |
| NEFA_15_1 | 0.07 | 0.031 | 0.111 |
| NEFA_16_0 | 43.45 | 28.375 | 69.625 |
| NEFA_16_1 | 7.905 | 4.51 | 13.9 |
| NEFA_17_0 | 1.275 | 0.81 | 1.865 |
| NEFA_17_1 | 0.682 | 0.402 | 1.073 |
| NEFA_18_0 | 14.85 | 9.547 | 20.925 |
| NEFA_18_1 | 73.4 | 46.3 | 107 |
| NEFA_18_2 | 22.2 | 13.4 | 31 |
| NEFA_18_3 | 2.505 | 1.593 | 3.98 |
| NEFA_20_1 | 0.626 | 0.377 | 0.894 |
| NEFA_20_2 | 0.362 | 0.215 | 0.521 |
| NEFA_20_3 | 0.439 | 0.304 | 0.588 |
| NEFA_20_4 | 1.37 | 1.06 | 1.69 |
| NEFA_22_6 | 0.876 | 0.627 | 1.21 |
| NEFA_24_1 | 0.135 | 0.086 | 0.196 |
| NEFA_12_1 | 0.19 | 0.102 | 0.348 |
| NEFA_13_1 | 0.028 | 0.005 | 0.045 |
| NEFA_14_2 | 0.02 | -0.003 | 0.064 |
| NEFA_16_2 | 0.111 | 0.063 | 0.174 |
| NEFA_18_4 | 0.04 | 0.023 | 0.068 |
| NEFA_19_0 | 0.133 | 0.092 | 0.192 |
| NEFA_19_1 | 0.236 | 0.15 | 0.368 |
| NEFA_20_5 | 0.24 | 0.152 | 0.317 |
| NEFA_22_4 | 0.208 | 0.139 | 0.281 |
| NEFA_22_5 | 0.364 | 0.248 | 0.522 |
| NEFA_24_4 | 0.013 | 0.008 | 0.018 |
| NEFA_24_5 | 0.015 | 0.009 | 0.022 |
| NEFA_26_1 | 0.09 | 0.059 | 0.136 |

### Supplemental Table 7.2: Hormonal taking females.

| **Metabolites** | **Median** | **25% Quartile** | **75% Quartile** |
| --- | --- | --- | --- |
| Carn | 32.328 | 26.801 | 38.32 |
| Carn.a.C10.0 | 0.258 | 0.179 | 0.411 |
| Carn.a.C10.1 | 0.296 | 0.22 | 0.392 |
| Carn.a.C12.0 | 0.085 | 0.054 | 0.125 |
| Carn.a.C12.1 | 0.331 | 0.207 | 0.536 |
| Carn.a.C14.0 | 0.033 | 0.024 | 0.047 |
| Carn.a.C14.1 | 0.114 | 0.086 | 0.135 |
| Carn.a.C14.2 | 0.017 | 0.011 | 0.027 |
| Carn.a.C16.0 | 0.072 | 0.057 | 0.087 |
| Carn.a.C16.1 | 0.031 | 0.022 | 0.043 |
| Carn.a.C18.0 | 0.028 | 0.02 | 0.036 |
| Carn.a.C18.1 | 0.082 | 0.064 | 0.101 |
| Carn.a.C2.0 | 4.224 | 3.44 | 5.176 |
| Carn.a.C3.0 | 0.316 | 0.24 | 0.375 |
| Carn.a.C4.0 | 0.224 | 0.168 | 0.287 |
| Carn.a.C4.0.DC | 0.001 | 0.001 | 0.002 |
| Carn.a.C5.0 | 0.127 | 0.095 | 0.171 |
| Carn.a.C6.0 | 0.046 | 0.032 | 0.067 |
| Carn.a.C8.1 | 0.101 | 0.075 | 0.139 |
| lyso.PC.a.C14.0 | 2.104 | 1.705 | 2.67 |
| lyso.PC.a.C16.0 | 70.591 | 60.417 | 87.32 |
| lyso.PC.a.C16.1 | 2.865 | 2.344 | 3.554 |
| lyso.PC.a.C18.0 | 16.113 | 11.973 | 20.091 |
| lyso.PC.a.C18.1 | 16.038 | 12.697 | 20.741 |
| lyso.PC.a.C18.2 | 27.228 | 19.218 | 35.905 |
| lyso.PC.a.C18.3 | 0.574 | 0.404 | 0.845 |
| lyso.PC.a.C18.6 | 0.251 | 0.187 | 0.306 |
| lyso.PC.a.C20.2 | 0.199 | 0.149 | 0.252 |
| lyso.PC.a.C20.3 | 1.891 | 1.49 | 2.46 |
| lyso.PC.a.C20.4 | 5.391 | 4.034 | 6.732 |
| lyso.PC.a.C20.5 | 0.503 | 0.306 | 0.742 |
| lyso.PC.a.C22.5 | 0.412 | 0.274 | 0.538 |
| lyso.PC.a.C22.6 | 1.345 | 1.036 | 1.614 |
| lyso.PC.e.C16.0 | 0.814 | 0.668 | 0.998 |
| lyso.PC.e.C18.0 | 1.315 | 1.045 | 1.636 |
| lyso.PC.e.C18.1 | 0.293 | 0.218 | 0.378 |
| PC.aa.C30.0 | 4.352 | 3.259 | 6.117 |
| PC.aa.C30.1 | 2.391 | 1.965 | 2.883 |
| PC.aa.C30.2 | 0.533 | 0.438 | 0.653 |
| PC.aa.C32.0 | 14.493 | 12.827 | 17.164 |
| PC.aa.C32.1 | 20.586 | 15.726 | 29.224 |
| PC.aa.C32.2 | 4.683 | 3.526 | 6.073 |
| PC.aa.C32.3 | 0.52 | 0.415 | 0.65 |
| PC.aa.C34.0 | 2.931 | 2.266 | 3.691 |
| PC.aa.C34.1 | 254.683 | 215.828 | 300.232 |
| PC.aa.C34.2 | 449.935 | 396.944 | 513.619 |
| PC.aa.C34.3 | 16.061 | 13.242 | 19.586 |
| PC.aa.C34.4 | 2.264 | 1.75 | 2.978 |
| PC.aa.C34.5 | 0.243 | 0.177 | 0.314 |
| PC.aa.C36.0 | 1.48 | 1.234 | 1.789 |
| PC.aa.C36.1 | 46.121 | 38.677 | 54.367 |
| PC.aa.C36.2 | 244.564 | 207.457 | 271.574 |
| PC.aa.C36.3 | 167.813 | 143.826 | 202.318 |
| PC.aa.C36.4 | 204.04 | 178.551 | 249.944 |
| PC.aa.C36.5 | 22.275 | 17.085 | 27.09 |
| PC.aa.C36.6 | 0.948 | 0.64 | 1.248 |
| PC.aa.C38.0 | 1.977 | 1.583 | 2.375 |
| PC.aa.C38.1 | 0.649 | 0.489 | 0.79 |
| PC.aa.C38.2 | 5.064 | 3.984 | 6.109 |
| PC.aa.C38.3 | 46.422 | 37.022 | 57.778 |
| PC.aa.C38.4 | 111.528 | 94.41 | 127.453 |
| PC.aa.C38.5 | 57.957 | 48.738 | 66.312 |
| PC.aa.C38.6 | 90.391 | 72.298 | 113.238 |
| PC.aa.C40.0 | 0.397 | 0.322 | 0.509 |
| PC.aa.C40.1 | 0.198 | 0.157 | 0.247 |
| PC.aa.C40.3 | 0.257 | 0.2 | 0.317 |
| PC.aa.C40.4 | 3.166 | 2.624 | 3.739 |
| PC.aa.C40.5 | 11.214 | 9.1 | 12.872 |
| PC.aa.C40.6 | 28.409 | 22.266 | 35.958 |
| PC.aa.C42.0 | 0.427 | 0.315 | 0.536 |
| PC.aa.C42.1 | 0.152 | 0.121 | 0.198 |
| PC.aa.C42.2 | 0.079 | 0.06 | 0.101 |
| PC.aa.C42.4 | 0.119 | 0.095 | 0.145 |
| PC.aa.C42.5 | 0.292 | 0.222 | 0.36 |
| PC.aa.C42.6 | 0.608 | 0.467 | 0.79 |
| PC.aa.C43.4 | 0.181 | 0.147 | 0.231 |
| PC.aa.C43.6 | 0.995 | 0.806 | 1.231 |
| PC.aa.C44.12 | 1.126 | 0.938 | 1.375 |
| PC.ae.C30.0 | 0.303 | 0.233 | 0.394 |
| PC.ae.C30.1 | 0.683 | 0.502 | 0.853 |
| PC.ae.C32.0 | 3.053 | 2.641 | 3.626 |
| PC.ae.C32.1 | 2.978 | 2.579 | 3.45 |
| PC.ae.C32.2 | 0.945 | 0.751 | 1.14 |
| PC.ae.C34.0 | 1.627 | 1.344 | 1.994 |
| PC.ae.C34.1 | 10.917 | 9.672 | 12.497 |
| PC.ae.C34.2 | 11.68 | 10.061 | 13.573 |
| PC.ae.C34.3 | 9.01 | 7.589 | 10.398 |
| PC.ae.C34.4 | 0.234 | 0.18 | 0.286 |
| PC.ae.C36.0 | 0.362 | 0.284 | 0.433 |
| PC.ae.C36.1 | 3.776 | 3.218 | 4.516 |
| PC.ae.C36.2 | 11.355 | 9.875 | 13.649 |
| PC.ae.C36.3 | 8.361 | 7.22 | 9.751 |
| PC.ae.C36.4 | 16.85 | 14.603 | 19.189 |
| PC.ae.C36.5 | 11.268 | 9.62 | 13.038 |
| PC.ae.C36.6 | 0.559 | 0.388 | 0.778 |
| PC.ae.C38.0 | 1.919 | 1.517 | 2.38 |
| PC.ae.C38.2 | 1.09 | 0.882 | 1.253 |
| PC.ae.C38.3 | 3.803 | 3.16 | 4.419 |
| PC.ae.C38.4 | 14.22 | 12.042 | 15.752 |
| PC.ae.C38.5 | 16.791 | 14.45 | 18.911 |
| PC.ae.C38.6 | 7.517 | 6.256 | 9.186 |
| PC.ae.C40.0 | 12.157 | 10.126 | 15.135 |
| PC.ae.C40.1 | 1.217 | 0.98 | 1.445 |
| PC.ae.C40.2 | 1.129 | 0.891 | 1.338 |
| PC.ae.C40.3 | 0.64 | 0.539 | 0.759 |
| PC.ae.C40.4 | 1.81 | 1.556 | 2.085 |
| PC.ae.C40.5 | 3.605 | 3.074 | 4.116 |
| PC.ae.C40.6 | 4.884 | 4.031 | 5.661 |
| PC.ae.C42.0 | 0.422 | 0.306 | 0.534 |
| PC.ae.C42.1 | 0.213 | 0.16 | 0.261 |
| PC.ae.C42.2 | 0.395 | 0.326 | 0.468 |
| PC.ae.C42.3 | 0.703 | 0.569 | 0.851 |
| PC.ae.C42.4 | 0.884 | 0.706 | 1.019 |
| PC.ae.C42.5 | 2.309 | 1.955 | 2.803 |
| PC.ae.C42.6 | 1.11 | 0.867 | 1.327 |
| SM.a.C30.1 | 0.442 | 0.333 | 0.533 |
| SM.a.C32.0 | 0.39 | 0.292 | 0.536 |
| SM.a.C32.1 | 8.664 | 7.467 | 10.048 |
| SM.a.C32.2 | 0.735 | 0.629 | 0.859 |
| SM.a.C33.1 | 5.755 | 4.958 | 6.541 |
| SM.a.C33.2 | 0.183 | 0.155 | 0.221 |
| SM.a.C34.0 | 2.001 | 1.573 | 2.588 |
| SM.a.C34.1 | 97.688 | 86.858 | 107.891 |
| SM.a.C34.2 | 14.279 | 12.709 | 15.836 |
| SM.a.C34.3 | 0.097 | 0.081 | 0.119 |
| SM.a.C35.0 | 0.555 | 0.454 | 0.647 |
| SM.a.C35.1 | 2.726 | 2.348 | 3.126 |
| SM.a.C36.0 | 1.688 | 1.306 | 2.015 |
| SM.a.C36.1 | 19.116 | 16.406 | 21.758 |
| SM.a.C36.2 | 9.737 | 8.485 | 11.269 |
| SM.a.C36.3 | 0.751 | 0.643 | 0.868 |
| SM.a.C37.1 | 2.031 | 1.694 | 2.492 |
| SM.a.C38.1 | 35.666 | 30.281 | 43.117 |
| SM.a.C38.2 | 19.953 | 17.281 | 22.729 |
| SM.a.C39.1 | 4.951 | 4.16 | 5.673 |
| SM.a.C39.2 | 1.382 | 1.109 | 1.731 |
| SM.a.C40.1 | 18.037 | 13.793 | 21.835 |
| SM.a.C40.2 | 32.113 | 27.967 | 36.507 |
| SM.a.C40.3 | 12.407 | 9.837 | 14.673 |
| SM.a.C40.4 | 2.854 | 2.465 | 3.519 |
| SM.a.C40.5 | 1.055 | 0.813 | 1.281 |
| SM.a.C41.1 | 11.22 | 9.116 | 12.915 |
| SM.a.C41.2 | 8.842 | 7.547 | 9.989 |
| SM.a.C42.1 | 18.824 | 15.972 | 21.711 |
| SM.a.C42.2 | 46.702 | 38.496 | 52.929 |
| SM.a.C42.3 | 25.776 | 22.031 | 29.425 |
| SM.a.C42.4 | 7.547 | 6.377 | 8.885 |
| SM.a.C42.5 | 3.133 | 2.432 | 3.913 |
| SM.a.C42.6 | 3.723 | 2.952 | 4.689 |
| SM.a.C43.1 | 1.072 | 0.9 | 1.264 |
| SM.a.C43.2 | 2.551 | 2.079 | 3.109 |
| SM.a.C44.2 | 0.258 | 0.206 | 0.316 |
| SM.a.C44.6 | 1.275 | 0.987 | 1.677 |
| SM.a.C31.1 | 0.252 | 0.208 | 0.306 |
| SM.a.C33.3 | 0.115 | 0.092 | 0.138 |
| SM.a.C35.2 | 0.384 | 0.329 | 0.453 |
| SM.a.C37.3 | 0.32 | 0.251 | 0.395 |
| SM.a.C39.5 | 0.396 | 0.286 | 0.503 |
| SM.a.C41.0 | 0.533 | 0.402 | 0.707 |
| SM.a.C41.3 | 1.346 | 1.132 | 1.682 |
| SM.a.C43.0 | 0.674 | 0.53 | 0.865 |
| SM.a.C43.3 | 0.608 | 0.497 | 0.718 |
| Ala | 307 | 242 | 387 |
| Arg | 57.9 | 47.4 | 70.35 |
| Asn | 41.9 | 36.45 | 49.15 |
| Asp | 7.98 | 6.78 | 9.52 |
| Cit | 19.3 | 14.7 | 24.4 |
| Gln | 470 | 393.5 | 560 |
| Glu | 51.2 | 38.8 | 64.1 |
| Gly | 160 | 133 | 206 |
| His | 86 | 70.675 | 112 |
| Ile | 48.55 | 40.1 | 55.45 |
| Leu | 98.6 | 86.4 | 115.5 |
| Lys | 156 | 124 | 196.5 |
| Met | 21.8 | 18.8 | 25.75 |
| Orn | 36.8 | 27.65 | 44.95 |
| Phe | 48.8 | 42.75 | 55.1 |
| Pro | 124 | 101 | 161 |
| Trp | 95 | 56.9 | 122 |
| Ser | 91.3 | 72.2 | 118 |
| Thr | 141 | 95.175 | 203.5 |
| Tyr | 45.3 | 36.4 | 53.9 |
| Val | 180 | 155.5 | 207.5 |
| NEFA_12_0 | 1.65 | 0.975 | 2.8 |
| NEFA_14_0 | 5.145 | 2.777 | 9.575 |
| NEFA_14_1 | 1.115 | 0.608 | 1.688 |
| NEFA_15_0 | 0.824 | 0.511 | 1.19 |
| NEFA_15_1 | 0.069 | 0.04 | 0.119 |
| NEFA_16_0 | 50.85 | 33.55 | 69.5 |
| NEFA_16_1 | 9.18 | 5.35 | 14.9 |
| NEFA_17_0 | 1.27 | 0.881 | 1.71 |
| NEFA_17_1 | 0.711 | 0.455 | 1.04 |
| NEFA_18_0 | 14.55 | 9.498 | 20.5 |
| NEFA_18_1 | 79.4 | 49.475 | 115.5 |
| NEFA_18_2 | 23.3 | 15.6 | 35.35 |
| NEFA_18_3 | 2.745 | 1.835 | 4.15 |
| NEFA_20_1 | 0.676 | 0.457 | 1.07 |
| NEFA_20_2 | 0.43 | 0.265 | 0.618 |
| NEFA_20_3 | 0.529 | 0.358 | 0.696 |
| NEFA_20_4 | 1.47 | 1.11 | 1.97 |
| NEFA_22_6 | 1.05 | 0.692 | 1.455 |
| NEFA_24_1 | 0.142 | 0.088 | 0.196 |
| NEFA_12_1 | 0.191 | 0.09 | 0.361 |
| NEFA_13_1 | 0.02 | 0.003 | 0.041 |
| NEFA_14_2 | 0.027 | -0.001 | 0.071 |
| NEFA_16_2 | 0.114 | 0.069 | 0.18 |
| NEFA_18_4 | 0.038 | 0.02 | 0.063 |
| NEFA_19_0 | 0.14 | 0.098 | 0.185 |
| NEFA_19_1 | 0.254 | 0.165 | 0.376 |
| NEFA_20_5 | 0.217 | 0.149 | 0.318 |
| NEFA_22_4 | 0.221 | 0.156 | 0.301 |
| NEFA_22_5 | 0.361 | 0.231 | 0.493 |
| NEFA_24_4 | 0.015 | 0.011 | 0.025 |
| NEFA_24_5 | 0.028 | 0.014 | 0.042 |
| NEFA_26_1 | 0.082 | 0.05 | 0.138 |

### Supplemental Table 7.3: Male subset.

| **Metabolites** | **Median** | **25% Quartile** | **75% Quartile** |
| --- | --- | --- | --- |
| Carn | 43.332 | 37.223 | 50.219 |
| Carn.a.C10.0 | 0.269 | 0.204 | 0.38 |
| Carn.a.C10.1 | 0.338 | 0.262 | 0.456 |
| Carn.a.C12.0 | 0.104 | 0.081 | 0.145 |
| Carn.a.C12.1 | 0.433 | 0.272 | 0.694 |
| Carn.a.C14.0 | 0.04 | 0.031 | 0.052 |
| Carn.a.C14.1 | 0.113 | 0.087 | 0.146 |
| Carn.a.C14.2 | 0.022 | 0.015 | 0.033 |
| Carn.a.C16.0 | 0.088 | 0.074 | 0.105 |
| Carn.a.C16.1 | 0.034 | 0.025 | 0.046 |
| Carn.a.C18.0 | 0.039 | 0.031 | 0.048 |
| Carn.a.C18.1 | 0.102 | 0.082 | 0.127 |
| Carn.a.C2.0 | 4.787 | 3.931 | 5.812 |
| Carn.a.C3.0 | 0.42 | 0.344 | 0.528 |
| Carn.a.C4.0 | 0.254 | 0.189 | 0.337 |
| Carn.a.C4.0.DC | 0.001 | 0.001 | 0.002 |
| Carn.a.C5.0 | 0.189 | 0.138 | 0.26 |
| Carn.a.C6.0 | 0.054 | 0.04 | 0.073 |
| Carn.a.C8.1 | 0.106 | 0.074 | 0.154 |
| lyso.PC.a.C14.0 | 2.111 | 1.594 | 2.754 |
| lyso.PC.a.C16.0 | 82.756 | 71.75 | 99.261 |
| lyso.PC.a.C16.1 | 3.229 | 2.686 | 3.929 |
| lyso.PC.a.C18.0 | 21.782 | 18.213 | 25.75 |
| lyso.PC.a.C18.1 | 23.796 | 19.589 | 28.722 |
| lyso.PC.a.C18.2 | 43.987 | 35.956 | 53.638 |
| lyso.PC.a.C18.3 | 0.91 | 0.707 | 1.184 |
| lyso.PC.a.C18.6 | 0.298 | 0.249 | 0.35 |
| lyso.PC.a.C20.2 | 0.24 | 0.184 | 0.296 |
| lyso.PC.a.C20.3 | 2.802 | 2.334 | 3.454 |
| lyso.PC.a.C20.4 | 8.364 | 6.769 | 9.91 |
| lyso.PC.a.C20.5 | 0.9 | 0.708 | 1.233 |
| lyso.PC.a.C22.5 | 0.717 | 0.587 | 0.865 |
| lyso.PC.a.C22.6 | 1.744 | 1.381 | 2.201 |
| lyso.PC.e.C16.0 | 0.98 | 0.803 | 1.191 |
| lyso.PC.e.C18.0 | 1.705 | 1.447 | 2.107 |
| lyso.PC.e.C18.1 | 0.422 | 0.345 | 0.515 |
| PC.aa.C30.0 | 3.378 | 2.477 | 4.4 |
| PC.aa.C30.1 | 2.014 | 1.555 | 2.522 |
| PC.aa.C30.2 | 0.483 | 0.391 | 0.572 |
| PC.aa.C32.0 | 12.461 | 10.865 | 14.091 |
| PC.aa.C32.1 | 15.505 | 11.721 | 20.186 |
| PC.aa.C32.2 | 3.165 | 2.388 | 4.173 |
| PC.aa.C32.3 | 0.446 | 0.352 | 0.562 |
| PC.aa.C34.0 | 2.726 | 2.034 | 3.416 |
| PC.aa.C34.1 | 218.642 | 188.479 | 255.634 |
| PC.aa.C34.2 | 363.064 | 319.581 | 420.708 |
| PC.aa.C34.3 | 12.82 | 10.432 | 15.494 |
| PC.aa.C34.4 | 1.815 | 1.36 | 2.215 |
| PC.aa.C34.5 | 0.255 | 0.189 | 0.349 |
| PC.aa.C36.0 | 1.467 | 1.157 | 1.785 |
| PC.aa.C36.1 | 45.241 | 37.498 | 54.409 |
| PC.aa.C36.2 | 235.602 | 201.663 | 265.509 |
| PC.aa.C36.3 | 139.382 | 117.45 | 158.438 |
| PC.aa.C36.4 | 170.961 | 147.072 | 200.908 |
| PC.aa.C36.5 | 22.054 | 16.925 | 26.548 |
| PC.aa.C36.6 | 0.774 | 0.559 | 0.957 |
| PC.aa.C38.0 | 1.821 | 1.429 | 2.138 |
| PC.aa.C38.1 | 0.59 | 0.47 | 0.727 |
| PC.aa.C38.2 | 3.925 | 3.206 | 4.722 |
| PC.aa.C38.3 | 41.645 | 33.132 | 50.582 |
| PC.aa.C38.4 | 103.458 | 87.464 | 121.751 |
| PC.aa.C38.5 | 56.335 | 48.648 | 65.04 |
| PC.aa.C38.6 | 68.124 | 54.913 | 82.699 |
| PC.aa.C40.0 | 0.374 | 0.29 | 0.449 |
| PC.aa.C40.1 | 0.186 | 0.151 | 0.226 |
| PC.aa.C40.3 | 0.231 | 0.18 | 0.283 |
| PC.aa.C40.4 | 3.106 | 2.549 | 3.661 |
| PC.aa.C40.5 | 11.544 | 9.489 | 13.765 |
| PC.aa.C40.6 | 24.075 | 18.82 | 29.075 |
| PC.aa.C42.0 | 0.351 | 0.278 | 0.444 |
| PC.aa.C42.1 | 0.133 | 0.106 | 0.164 |
| PC.aa.C42.2 | 0.075 | 0.059 | 0.093 |
| PC.aa.C42.4 | 0.101 | 0.08 | 0.122 |
| PC.aa.C42.5 | 0.207 | 0.168 | 0.255 |
| PC.aa.C42.6 | 0.509 | 0.409 | 0.637 |
| PC.aa.C43.4 | 0.163 | 0.132 | 0.202 |
| PC.aa.C43.6 | 0.927 | 0.746 | 1.115 |
| PC.aa.C44.12 | 1.044 | 0.852 | 1.257 |
| PC.ae.C30.0 | 0.258 | 0.188 | 0.346 |
| PC.ae.C30.1 | 0.576 | 0.45 | 0.77 |
| PC.ae.C32.0 | 2.829 | 2.42 | 3.261 |
| PC.ae.C32.1 | 2.76 | 2.321 | 3.172 |
| PC.ae.C32.2 | 0.805 | 0.654 | 0.97 |
| PC.ae.C34.0 | 1.569 | 1.262 | 1.852 |
| PC.ae.C34.1 | 9.823 | 8.526 | 11.117 |
| PC.ae.C34.2 | 10.747 | 9.083 | 12.499 |
| PC.ae.C34.3 | 8.979 | 7.183 | 10.683 |
| PC.ae.C34.4 | 0.229 | 0.175 | 0.303 |
| PC.ae.C36.0 | 0.353 | 0.285 | 0.421 |
| PC.ae.C36.1 | 3.769 | 3.068 | 4.509 |
| PC.ae.C36.2 | 10.767 | 9.16 | 12.914 |
| PC.ae.C36.3 | 7.943 | 6.704 | 9.33 |
| PC.ae.C36.4 | 17.522 | 15.01 | 19.959 |
| PC.ae.C36.5 | 12.594 | 10.58 | 14.58 |
| PC.ae.C36.6 | 0.744 | 0.546 | 1.036 |
| PC.ae.C38.0 | 1.689 | 1.366 | 2.032 |
| PC.ae.C38.2 | 0.972 | 0.81 | 1.177 |
| PC.ae.C38.3 | 3.319 | 2.801 | 3.897 |
| PC.ae.C38.4 | 13.74 | 11.643 | 15.633 |
| PC.ae.C38.5 | 17.729 | 15.481 | 20.221 |
| PC.ae.C38.6 | 7.542 | 6.305 | 8.913 |
| PC.ae.C40.0 | 9.901 | 7.971 | 11.624 |
| PC.ae.C40.1 | 1.185 | 0.972 | 1.418 |
| PC.ae.C40.2 | 0.876 | 0.709 | 1.052 |
| PC.ae.C40.3 | 0.554 | 0.469 | 0.652 |
| PC.ae.C40.4 | 1.711 | 1.44 | 1.955 |
| PC.ae.C40.5 | 3.626 | 3.091 | 4.14 |
| PC.ae.C40.6 | 4.535 | 3.821 | 5.248 |
| PC.ae.C42.0 | 0.274 | 0.208 | 0.351 |
| PC.ae.C42.1 | 0.2 | 0.158 | 0.247 |
| PC.ae.C42.2 | 0.396 | 0.326 | 0.471 |
| PC.ae.C42.3 | 0.612 | 0.504 | 0.735 |
| PC.ae.C42.4 | 0.785 | 0.623 | 0.931 |
| PC.ae.C42.5 | 2.09 | 1.721 | 2.473 |
| PC.ae.C42.6 | 1.028 | 0.839 | 1.245 |
| SM.a.C30.1 | 0.325 | 0.237 | 0.419 |
| SM.a.C32.0 | 0.301 | 0.215 | 0.411 |
| SM.a.C32.1 | 7.564 | 6.178 | 9.069 |
| SM.a.C32.2 | 0.531 | 0.413 | 0.641 |
| SM.a.C33.1 | 5.42 | 4.639 | 6.451 |
| SM.a.C33.2 | 0.158 | 0.128 | 0.191 |
| SM.a.C34.0 | 1.518 | 1.187 | 2.033 |
| SM.a.C34.1 | 89.313 | 79.086 | 99.798 |
| SM.a.C34.2 | 12.046 | 10.623 | 13.591 |
| SM.a.C34.3 | 0.081 | 0.064 | 0.102 |
| SM.a.C35.0 | 0.525 | 0.442 | 0.607 |
| SM.a.C35.1 | 2.684 | 2.25 | 3.103 |
| SM.a.C36.0 | 1.241 | 0.935 | 1.595 |
| SM.a.C36.1 | 16.034 | 13.607 | 18.671 |
| SM.a.C36.2 | 8.344 | 7.061 | 9.681 |
| SM.a.C36.3 | 0.639 | 0.52 | 0.758 |
| SM.a.C37.1 | 1.803 | 1.483 | 2.112 |
| SM.a.C38.1 | 29.262 | 24.673 | 34.792 |
| SM.a.C38.2 | 15.737 | 14.092 | 18.256 |
| SM.a.C39.1 | 4.249 | 3.458 | 5.162 |
| SM.a.C39.2 | 1.149 | 0.937 | 1.41 |
| SM.a.C40.1 | 14.427 | 10.786 | 19.628 |
| SM.a.C40.2 | 27.179 | 23.58 | 31.002 |
| SM.a.C40.3 | 9.469 | 7.878 | 11.552 |
| SM.a.C40.4 | 2.395 | 2.008 | 2.802 |
| SM.a.C40.5 | 0.978 | 0.758 | 1.242 |
| SM.a.C41.1 | 9.879 | 8.184 | 11.699 |
| SM.a.C41.2 | 7.379 | 6.221 | 8.663 |
| SM.a.C42.1 | 16.65 | 14.343 | 20.042 |
| SM.a.C42.2 | 37.61 | 32.486 | 43.575 |
| SM.a.C42.3 | 20.457 | 17.334 | 23.285 |
| SM.a.C42.4 | 6.884 | 5.731 | 7.914 |
| SM.a.C42.5 | 2.477 | 1.93 | 3.085 |
| SM.a.C42.6 | 2.669 | 2.142 | 3.265 |
| SM.a.C43.1 | 1.05 | 0.822 | 1.269 |
| SM.a.C43.2 | 2.226 | 1.81 | 2.779 |
| SM.a.C44.2 | 0.235 | 0.188 | 0.287 |
| SM.a.C44.6 | 1.041 | 0.818 | 1.336 |
| SM.a.C31.1 | 0.209 | 0.156 | 0.266 |
| SM.a.C33.3 | 0.105 | 0.085 | 0.127 |
| SM.a.C35.2 | 0.372 | 0.3 | 0.44 |
| SM.a.C37.3 | 0.301 | 0.247 | 0.371 |
| SM.a.C39.5 | 0.421 | 0.325 | 0.539 |
| SM.a.C41.0 | 0.411 | 0.315 | 0.544 |
| SM.a.C41.3 | 1.114 | 0.897 | 1.39 |
| SM.a.C43.0 | 0.53 | 0.436 | 0.685 |
| SM.a.C43.3 | 0.497 | 0.401 | 0.601 |
| Ala | 320.5 | 249 | 396.75 |
| Arg | 68.7 | 55.9 | 83.8 |
| Asn | 42.4 | 36.675 | 48.7 |
| Asp | 7.745 | 6.433 | 9.218 |
| Cit | 25.2 | 20.025 | 29.9 |
| Gln | 565.5 | 489 | 657 |
| Glu | 59.85 | 46.95 | 75.275 |
| Gly | 205 | 170 | 240 |
| His | 85.1 | 67.8 | 111 |
| Ile | 60.65 | 49.925 | 73.1 |
| Leu | 132 | 113 | 148 |
| Lys | 183 | 145.75 | 228 |
| Met | 25.9 | 22.2 | 30.3 |
| Orn | 51.7 | 42.075 | 61.8 |
| Phe | 55.65 | 49.55 | 62 |
| Pro | 170 | 129.25 | 224 |
| Trp | 115 | 72.15 | 150.75 |
| Ser | 93.95 | 71.625 | 117 |
| Thr | 135 | 89.45 | 192 |
| Tyr | 56.4 | 48.9 | 64.95 |
| Val | 223 | 191 | 256 |
| NEFA_12_0 | 1.24 | 0.683 | 2.2 |
| NEFA_14_0 | 5.05 | 2.43 | 9.055 |
| NEFA_14_1 | 0.681 | 0.387 | 1.15 |
| NEFA_15_0 | 0.719 | 0.428 | 1.14 |
| NEFA_15_1 | 0.058 | 0.029 | 0.094 |
| NEFA_16_0 | 44.05 | 31.525 | 67.725 |
| NEFA_16_1 | 5.775 | 3.353 | 10.5 |
| NEFA_17_0 | 1.3 | 0.935 | 1.902 |
| NEFA_17_1 | 0.598 | 0.391 | 0.959 |
| NEFA_18_0 | 14.8 | 9.898 | 23.125 |
| NEFA_18_1 | 66.55 | 43.875 | 97.6 |
| NEFA_18_2 | 19.8 | 13.725 | 28.5 |
| NEFA_18_3 | 2.27 | 1.47 | 3.53 |
| NEFA_20_1 | 0.579 | 0.378 | 0.833 |
| NEFA_20_2 | 0.34 | 0.22 | 0.494 |
| NEFA_20_3 | 0.458 | 0.34 | 0.634 |
| NEFA_20_4 | 1.495 | 1.18 | 1.89 |
| NEFA_22_6 | 0.829 | 0.612 | 1.21 |
| NEFA_24_1 | 0.157 | 0.111 | 0.223 |
| NEFA_12_1 | 0.121 | 0.053 | 0.261 |
| NEFA_13_1 | 0.023 | 0.002 | 0.047 |
| NEFA_14_2 | 0.017 | -0.004 | 0.057 |
| NEFA_16_2 | 0.089 | 0.051 | 0.133 |
| NEFA_18_4 | 0.032 | 0.018 | 0.055 |
| NEFA_19_0 | 0.152 | 0.101 | 0.2 |
| NEFA_19_1 | 0.23 | 0.153 | 0.341 |
| NEFA_20_5 | 0.243 | 0.164 | 0.342 |
| NEFA_22_4 | 0.206 | 0.155 | 0.274 |
| NEFA_22_5 | 0.362 | 0.261 | 0.497 |
| NEFA_24_4 | 0.013 | 0.008 | 0.019 |
| NEFA_24_5 | 0.012 | 0.007 | 0.022 |
| NEFA_26_1 | 0.106 | 0.07 | 0.187 |
